# Candidate chemosensory genes identified in *Colaphellus bowringi* by antennal transcriptome analysis

**DOI:** 10.1186/s12864-015-2236-3

**Published:** 2015-12-02

**Authors:** Xiao-Ming Li, Xiu-Yun Zhu, Zhi-Qiang Wang, Yi Wang, Peng He, Geng Chen, Liang Sun, Dao-Gui Deng, Ya-Nan Zhang

**Affiliations:** College of Life Sciences, Huaibei Normal University, Huaibei, 235000 China; State Key Laboratory Breeding Base of Green Pesticide and Agricultural Bioengineering, Key Laboratory of Green Pesticide and Agricultural Bioengineering, Ministry of Education, Guizhou University, Guiyang, 550025 China; Tea Research Institute, Chinese Academy of Agricultural Sciences, Hangzhou, 310008 China

**Keywords:** Transcriptome analysis, Chemosensory gene, Antenna, Tissue expression, Cabbage beetle

## Abstract

**Background:**

Since chemosensory genes play key roles in insect behaviour, they can potentially be used as new targets for pest control. The cabbage beetle, *Colaphellus bowringi*, is a serious insect pest of cruciferous vegetables in China and other Asian countries. However, a systematic identification of the chemosensory genes expressed in the antennae has not been reported.

**Results:**

We assembled the antennal transcriptome of *C. bowringi* by using Illumina sequencing technology and identified 104 candidate chemosensory genes by analyzing transcriptomic data, which included transcripts encoding 26 odorant-binding proteins (OBPs), 12 chemosensory proteins (CSPs), four sensory neuron membrane proteins (SNMPs), 43 odorant receptors (ORs), nine ionotropic receptors (IRs), and ten gustatory receptors (GRs). The data obtained are similar to those found in other coleopteran species, suggesting that our approach successfully identified the chemosensory genes of *C. bowringi*. The expression patterns of 43 OR genes, some of which were predominately found in the antenna or associated with sex-biased expression, were analyzed using quantitative real time RT-PCR (qPCR).

**Conclusions:**

Our study revealed that a large number of chemosensory genes are expressed in *C. bowringi*. These candidate chemosensory genes and their expression profiles in various tissues provide further information on understanding their function in *C. bowringi* as well as other insects, and identifying potential targets to disrupt the odorant system in *C. bowringi* so that new methods for pest management can be developed.

**Electronic supplementary material:**

The online version of this article (doi:10.1186/s12864-015-2236-3) contains supplementary material, which is available to authorized users.

## Background

The olfactory system plays a key role in various insect behaviours, such as those related to locating suitable hosts, avoiding predators, identifying oviposition sites, and finding sexual partners [1]. The antennae are crucial olfactory organs in this system, and many studies have demonstrated that the system generally involves two main steps. Firstly, odorants penetrate the sensillar lymph through pores, wherein they are recognised and bound by odorant-binding proteins (OBPs) [2–4] or chemosensory proteins (CSPs) [5, 6]. Secondly, it was speculated that the OBPs or CSPs were the transporters that transferred odorants through the sensillar lymph to a family of integral membrane protein, the olfactory receptors (ORs), located on the dendrites of olfactory receptor neurons (ORNs) [7–10]. Additionally, sensory neuron membrane proteins (SNMPs) [11, 12] and ionotropic receptors (IRs) [2, 13–15] have also been proposed to play a role in insect olfaction.

To thoroughly explore the mechanisms of insect olfaction, tissue or sex expression profiling as well as functional analyses of candidate chemosensory genes are the primary important steps that should be performed. Compared with initial techniques such as gene cloning with degenerate primers and Rapid Amplification of cDNA Ends (RACE) [16–19], RNA-seq is considered to be a timesaving, cost effective, and highly efficient method. Therefore, large-scale studies identifying chemosensory genes have been undertaken with distinct insects whose genomes have not been sequenced in recent years, such as *Ips typographus* (european spruce bark beetle) [20], *Dendroctonus ponderosae* (mountain pine beetle) [20], *Dendroctonus valens* (red turpentine beetle) [21], *Anomala corpulenta* (metallic green beetle) [22], *Sesamia inferens* (purple stem borer) [23], and *Helicoverpa armigera* (cotton bollworm) [24].

To date, many chemosensory genes have been identified from insects of almost every insect order. However, their exact functions are largely unknown, as these genes were identified based on sequence similarity to previously reported genes. Examination of gene expression profiles, particularly the tissue or sex distribution, and phylogenetic analyses could potentially provide important information concerning the function of chemosensory genes [25–30].

The cabbage beetle, *Colaphellus bowringi* Baly (Coleoptera: Chrysomelidae), is a serious insect pest and widely distributed in China as well as some other Asian countries. It primarily feeds on the developing leaves of cruciferous vegetables such as *Raphanus sativus*, *Brassica chinensis*, *B. pekinensis* and *B. campestris*, and aestivates and hibernates in the soil during the adult stage [31, 32]. There are two distinct infestation peaks annually: one in spring with a single generation and a second in autumn involving three generations. Both sexes copulate an average of five times per day [33–35], and 15-dayold partners have significantly greater mating success in mate choice than other developmental stages [36, 37]. However, highly effective sex attractants and pesticides to control the pest are not available [38, 39].

In this study, we performed a transcriptome analysis of adult antennae of *C. bowringi*, and identified 104 candidate chemosensory genes comprising 26 OBPs, 12 CSPs, 4 SNMPs, 43 ORs, 9 IRs, and 10 GRs. Furthermore, we conducted a comprehensive and comparative phylogenetic analysis and examined OR gene transcription patterns using quantitative real-time RT-PCR (qPCR). The results clearly revealed a unique feature of sex-biased expression of some ORs, and ultimately allowed us to identify potential targets to disrupt odorant perception in *C. bowringi* that could lead to new pest management techniques.

## Results

### Transcriptome sequencing and sequence assembly

We carried out next-generation sequencing on a cDNA library constructed from the adult antennae of *C. bowringi* using the Illumina HiSeq™ 2500 platform. The transcriptome sequence consisted of approximately 50 million clean reads (5.0 Gb). After clustering and redundancy filtering, we identified 41,761 unigenes with an N50 length of 1510 bp (Table 1). We called these 41,761sequences unigenses, although each might not necessarily represent a unique gene. Of the 41,761 unigenes, those with a sequence length greater than 500 bp accounted for 39.55 % of the transcriptome assembly (Additional file 1: Figure S1).

**Table 1:** Summary of *C. bowringi* transcriptome assembly

Statistics project	Number
Total clean reads	50,737,524
GC percentage	41.13 %
Q20 percentage	96.84 %
Total unigene nucleotides	33,080,006
Total unigene	41,761
N50 of unigenes (nt)	1510
Min length of unigenes (nt)	201
Median length of unigenes (nt)	379
Max length of unigenes (nt)	21,193
Unigenes with homolog in NR	18,903

### Homology analysis and Gene Ontology (GO) annotation

Among the 41,761 unigenes, 18,903 were matched by a Blastx similarity of the entries in the NCBI non-redundant (nr) protein database, with a cut-off E-value of 10^−5^. The highest match percentage (54.60 %) was to *Tribolium castaneum* (red flour beetle) sequences followed by *Dendroctonus ponderosae* (16.70 %), *Acyrthosiphon pisum* (pea aphid) (2.50 %), *Diaphorina citri* (asian citrus psyllid) (1.90 %) and *Bombyx mori* (silkworm) (1.30 %) (Additional file 2: Figure S2).

Gene Ontology (GO) annotation was used to classify transcripts into functional groups according to the GO category. Of the 41,761 unigenes, 14,147 (33.87 %) could be annotated based on sequence similarity. In the molecular function category, the genes expressed in the antenna were mostly associated with binding, catalytic, and transporter activities. In the biological process category, cellular, metabolic, and single-organism processes were the most represented. In the cellular component category, cell, cell part, and organelle were the most abundant groups (Additional file 3: Figure S3).

### Identification of candidate chemosensory genes

**Fig. 1:**
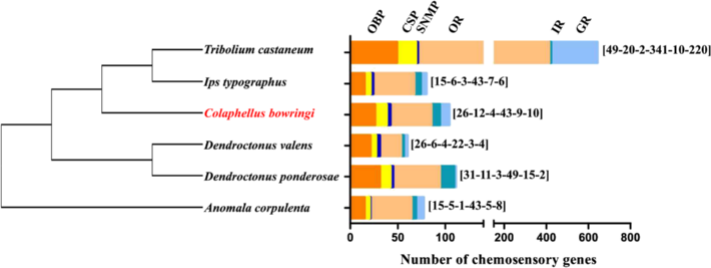
The number of chemosensory genes in different insect species. The digits by the histogram bars represent number of chemosensory genes in different subfamilies. A phylogenetic tree showing the phylogenetic relationships between these species is illustrated on the left. The data are obtained from the current study for *C. bowringi* and from the references [9, 10, 12, 15] for *Tribolium castaneum*, [20] for *Ips typographus* and *Dendroctonus ponderosae*, [21] for *D. valens* and [22] for *Anomala corpulenta*

By similarity analysis, a total of 104 transcripts belonging to gene families putatively involved in insect chemoreception were identified, including OBPs (26 transcripts), CSPs (12 transcripts), SNMPs (four transcripts), ORs (43 transcripts), IRs (9 transcripts) and GRs (10 transcripts) (Tables 2 and 3). Compared with insects where the chemosensory genes had been identified by analyzing either the genome or transcriptome, the number of candidate chemosensory genes identified here in *C. bowringi* was similar to those in *D. ponderosae* (111) and more than *I. typographus* (80), but less than in *T. castaneum* (642) (Fig. 1).

**Table 2:** The Blastx Match of *C. bowringi* candidate OBPs, CSPs and SNMPs genes

Gene	Acc.	ORF	Signal	Complete	Best Blastx Match					
Name	No.	(aa)	Peptide	ORF	Name	Acc. No.	Species	E value	Identity (%)	Group
*Odorant Binding Protein (OBP)*										
OBP1	KT381483	142	1–19	Y	odorant-binding protein 1	AHA33382.1	*Batocera horsfieldi*	2.00E-61	65	Classic
OBP2	KT381484	142	1–21	Y	odorant binding protein 23	EFA10803.1	*Tribolium castaneum*	1.00E-43	59	Classic
OBP3	KT381485	89	N	N	odorant-binding protein 5	AHA39270.1	*Monochamus alternatus*	1.00E-20	47	--
OBP4	KT381486	113	N	N	odorant-binding protein 26	AGI05179.1	*Dendroctonus ponderosae*	5.00E-19	35	--
OBP5	KT381487	131	1–18	Y	odorant-binding protein 5	AHA39270.1	*Monochamus alternatus*	5.00E-10	35	Minus-C
OBP6	KT381488	133	1–16	Y	odorant-binding protein 2	AHA39267.1	*Monochamus alternatus*	1.00E-37	58	Minus-C
OBP7	KT381489	136	1–21	Y	pheromone binding protein PBP1	AIV43008.1	*Batocera horsfieldi*	4.00E-48	63	Classic
OBP8	KT381490	134	1–24	Y	odorant-binding protein 3	AHA33381.1	*Batocera horsfieldi*	1.00E-07	35	Classic
OBP9	KT381491	151	1–19	Y	odorant-binding protein 1	AJM71475.1	*Tenebrio molitor*	1.00E-27	37	Classic
OBP10	KT381492	134	1–27	Y	odorant binding protein 05	EFA05677.1	*Tribolium castaneum*	2.00E-06	37	Classic
OBP11	KT381493	140	1–18	Y	odorant binding protein 1	ABR53888.1	*Monochamus alternatus*	2.00E-10	33	Minus-C
OBP12	KT381494	248	1–18	Y	odorant binding protein 2	AKK25130.1	*Dendroctonus valens*	1.00E-31	62	Classic
OBP13	KT381495	133	1–18	Y	odorant binding protein	AHE13799.1	*Lissorhoptrus oryzophilus*	1.00E-22	37	Minus-C
OBP14	KT381496	123	1–23	Y	odorant-binding protein 16	AGI05186.1	*Dendroctonus ponderosae*	3.00E-06	29	Classic
OBP15	KT381497	140	1–19	Y	minus-C odorant binding protein 3	ADD82416.1	*Batocera horsfieldi*	8.00E-14	40	Minus-C
OBP16	KT381498	141	1–18	Y	minus-C odorant binding protein 2	ADD70031.1	*Batocera horsfieldi*	3.00E-18	32	Minus-C
OBP17	KT381499	136	1–18	Y	odorant-binding protein 2	AHA33380.1	*Batocera horsfieldi*	4.00E-48	57	Classic
OBP18	KT381500	179	1–22	Y	odorant-binding protein 28	AHF71059.1	*Lygus lineolaris*	2.00E-50	50	Plus-C
OBP19	KT381501	130	1–22	Y	odorant-binding protein 5	AHA39270.1	*Monochamus alternatus*	8.00E-14	34	Minus-C
OBP20	KT381502	130	1–15	Y	odorant binding protein 4	AKK25132.1	*Dendroctonus valens*	2.00E-34	44	Minus-C
OBP21	KT381503	142	1–20	Y	odorant binding protein	AHE13800.1	*Lissorhoptrus oryzophilus*	1.00E-07	25	Minus-C
OBP22	KT381504	132	1–16	Y	odorant binding protein 10	AKK25136.1	*Dendroctonus valens*	4.00E-13	30	Minus-C
OBP23	KT381505	130	1–19	Y	odorant binding protein C03	EFA07546.1	*Tribolium castaneum*	9.00E-18	37	Minus-C
OBP24	KT381506	103	N	N	minus-C odorant binding protein 4	ADD82417.1	*Batocera horsfieldi*	7.00E-12	36	--
OBP25	KT381507	248	1–21	Y	odorant-binding protein 2	AGI05158.1	*Dendroctonus ponderosae*	2.00E-51	41	Plus-C
OBP26	KT381508	150	1–16	Y	odorant binding protein 12	EFA02857.1	*Tribolium castaneum*	1.00E-22	36	Classic
*Chemosensory Protein (CSP)*										
CSP1	KT381509	126	1–18	Y	chemosensory protein 12	NP_001039280.1	*Tribolium castaneum*	1.00E-40	56	
CSP2	KT381510	138	1–19	Y	chemosensory protein 11 precursor	NP_001039279.1	*Tribolium castaneum*	2.00E-31	52	
CSP3	KT381511	130	1–19	Y	chemosensory protein 6	AGI05162.1	*Dendroctonus ponderosae*	4.00E-34	55	
CSP4	KT381512	124	1–18	Y	CSP11	AKI84394.1	*Holotrichia parallela*	1.00E-37	53	
CSP5	KT381513	126	1–17	Y	chemosensory protein 8	AHE13803.1	*Lissorhoptrus oryzophilus*	7.00E-45	80	
CSP6	KT381514	123	1–19	Y	chemosensory protein 2	AGI05172.1	*Dendroctonus ponderosae*	7.00E-36	45	
CSP7	KT381515	125	1–18	Y	chemosensory protein 7 precursor	NP_001039289.1	*Tribolium castaneum*	1.00E-54	69	
CSP8	KT381516	97	1–19	N	chemosensory protein CSP3	AJO62209.1	*Tenebrio molitor*	7.00E-33	59	
CSP9	KT381517	148	N	Y	chemosensory protein CSP7	AJO62213.1	*Tenebrio molitor*	9.00E-31	46	
CSP10	KT381518	138	1–16	Y	chemosensory protein 8	AHE13803.1	*Lissorhoptrus oryzophilus*	3.00E-30	45	
CSP11	KT381519	113	N	Y	chemosensory protein 1 isoform X1	XP_008200934.1	*Tribolium castaneum*	1.00E-42	69	
CSP12	KT381520	162	N	Y	chemosensory protein	AFI45003.1	*Dendroctonus ponderosae*	9.00E-65	72	
*Sensory Neuron Membrane Protein (SNMP)*										
SNMP1a	KT381536	514		Y	SNMP-1	AJO62245.1	*Tenebrio molitor*	0.00E + 00	59	
SNMP1b	KT381537	534		Y	sensory neuron membrane protein	AFI45066.1	*Dendroctonus ponderosae*	0.00E + 00	52	
SNMP2	KT381538	522		Y	SNMP-2	AJO62246.1	*Tenebrio molitor*	9.00E-119	42	
SNMP3	KT381539	520		Y	sensory neuron membrane protein 2-like	XP_008198962.1	*Tribolium castaneum*	3.00E-162	46

**Table 3:** The Blastx Match of *C. bowringi* candidate ORs, IRs and GR genes

Gene	Acc.	ORF	Signal	Complete	Best Blastx Match				
Name	No.	(aa)	Peptide	ORF	Name	Acc. No.	Species	E value	Identity (%)
*Odorant Receptor (OR)*									
OR1	KT381540	344	4	N	odorant receptor 127	EEZ97733.1	*Tribolium castaneum*	1.00E-24	26
ORco(OR2)	KT381541	479	7	Y	odorant receptor co-receptor	AJF94638.2	*Ambrostoma quadriimpressum*	0.00E + 00	92
OR3	KT381542	330	5	N	odorant receptor 43	EEZ99411.1	*Tribolium castaneum*	6.00E-101	44
OR4	KT381543	378	6	Y	odorant receptor 14	AKC58549.1	*Anomala corpulenta*	3.00E-44	30
OR5	KT381544	254	4	N	odorant receptor 18	AKC58553.1	*Anomala corpulenta*	1.00E-16	28
OR6	KT381545	384	7	Y	odorant receptor 89	EFA10702.1	*Tribolium castaneum*	3.00E-43	32
OR7	KT381546	318	4	N	olfactory receptor OR16	AJO62235.1	*Tenebrio molitor*	2.00E-47	32
OR8	KT381547	174	3	N	odorant receptor 44	EEZ99412.1	*Tribolium castaneum*	3.00E-43	40
OR9	KT381548	336	5	N	odorant receptor 59	EEZ99171.1	*Tribolium castaneum*	1.00E-91	44
OR10	KT381549	363	5	Y	odorant receptor 14	AKC58549.1	*Anomala corpulenta*	1.00E-41	27
OR11	KT381550	336	5	N	odorant receptor 64	EFA10800.1	*Tribolium castaneum*	5.00E-146	56
OR12	KT381551	156	2	N	odorant receptor 47	EFA02940.1	*Tribolium castaneum*	9.00E-24	36
OR13	KT381552	420	8	Y	odorant receptor 14	AKC58549.1	*Anomala corpulenta*	1.00E-33	27
OR14	KT381553	356	4	N	odorant receptor 58	EEZ99414.1	*Tribolium castaneum*	1.00E-49	33
OR15	KT381554	389	7	Y	odorant receptor 3	EFA01310.1	*Tribolium castaneum*	1.00E-66	36
OR16	KT381555	196	0	N	odorant receptor 128	EFA02867.1	*Tribolium castaneum*	9.00E-10	21
OR17	KT381556	387	7	Y	odorant receptor 80	EFA10776.1	*Tribolium castaneum*	3.00E-55	30
OR18	KT381557	174	0	N	odorant receptor 49b-like	XP_001812261.1	*Tribolium castaneum*	4.00E-18	34
OR19	KT381558	379	6	Y	odorant receptor 89	EFA10702.1	*Tribolium castaneum*	2.00E-45	32
OR20	KT381559	248	3	N	odorant receptor 23	AGI05173.1	*Dendroctonus ponderosae*	3.00E-12	24
OR21	KT381560	355	4	N	odorant receptor 58	EEZ99414.1	*Tribolium castaneum*	1.00E-48	32
OR22	KT381561	390	3	Y	odorant receptor 89	EFA10702.1	*Tribolium castaneum*	3.00E-41	29
OR23	--	42	0	N	odorant receptor 82a	XP_966790.1	*Tribolium castaneum*	3.00E-57	45
OR24	KT381562	395	4	Y	odorant receptor 89	EFA10702.1	*Tribolium castaneum*	2.00E-51	29
OR25	--	58	0	N	odorant receptor 35	EEZ99408.1	*Tribolium castaneum*	2.00E-21	73
OR26	KT381563	380	6	Y	odorant receptor 41	EEZ99227.1	*Tribolium castaneum*	2.00E-46	29
OR27	KT381564	398	6	Y	odorant receptor 167	EFA02801.1	*Tribolium castaneum*	9.00E-70	35
OR28	KT381565	368	4	Y	odorant receptor 120	EEZ99330.1	*Tribolium castaneum*	3.00E-14	24
OR29	KT381566	250	3	N	odorant receptor 14	AKC58549.1	*Anomala corpulenta*	7.00E-29	29
OR30	KT381567	194	4	N	odorant receptor 37	EEZ99229.1	*Tribolium castaneum*	3.00E-29	30
OR31	KT381568	418	6	Y	odorant receptor 14	AKC58549.1	*Anomala corpulenta*	1.00E-36	27
OR32	KT381569	393	6	Y	odorant receptor 94a-like	XP_011629601.1	*Pogonomyrmex barbatus*	5.00E-13	27
OR33	KT381570	185	2	N	olfactory receptor OR10	AJO62229.1	*Tenebrio molitor*	3.00E-51	37
OR34	KT381571	436	5	Y	odorant receptor Or1-like	XP_008560066.1	*Microplitis demolitor*	3.00E-16	27
OR35	KT381572	388	4	Y	odorant receptor 92	EFA02873.1	*Tribolium castaneum*	6.00E-49	30
OR36	KT381573	383	7	Y	odorant receptor 14	AKC58549.1	*Anomala corpulenta*	7.00E-58	32
OR37	KT381574	371	4	Y	odorant receptor 123	EEZ99420.1	*Tribolium castaneum*	2.00E-14	24
OR38	KT381575	271	2	N	odorant receptor 37	EEZ99229.1	*Tribolium castaneum*	2.00E-82	47
OR39	KT381576	121	2	N	olfactory receptor OR60	AJE25900.1	*Planotortrix excessana*	6.00E-07	39
OR40	KT381577	369	6	Y	putative olfactory receptor 10	BAR43452.1	*Ostrinia furnacalis*	1.00E-19	23
OR41	KT884514	79	0	N	odorant receptor 184	EFA01394.1	*Tribolium castaneum*	1.00E-05	33
OR42	KT884515	83	0	N	olfactory receptor 17	CAM84015.1	*Tribolium castaneum*	3.00E-04	27
OR43	KT884516	73	0	N	odorant receptor 3	EFA01310.1	*Tribolium castaneum*	1.00E-04	38
*Ionotropic Receptor (IR)*									
IR6	KT381529	920	3	Y	chemosensory ionotropic receptor IR6	AJO62244.1	*Tenebrio molitor*	0.00E + 00	80
IR21a	KT381530	158	1	N	chemosensory ionotropic receptor 21a	AKC58586.1	*Anomala corpulenta*	1.00E-82	58
IR75q	KT381531	483	3	N	chemosensory ionotropic receptor 75q	AKC58589.1	*Anomala corpulenta*	3.00E-110	46
IR8a	KT381532	866	3	Y	ionotropic receptor 8a	AGI05169.1	*Dendroctonus ponderosae*	0.00E + 00	60
IR41a	KT381533	347	2	N	chemosensory ionotropic receptor 41a	AKC58587.1	*Anomala corpulenta*	5.00E-65	40
IR5	KT381534	552	3	Y	chemosensory ionotropic receptor IR5	AJO62243.1	*Tenebrio molitor*	4.00E-180	52
IR2	KT381535	607	3	Y	chemosensory ionotropic receptor IR2	AJO62240.1	*Tenebrio molitor*	0.00E + 00	60
IR64a	KT884510	213	1	N	ionotropic receptor	BAR64801.1	*Ostrinia furnacalis*	6.00E-57	38
IR68a	KT884511	95	0	N	ionotropic receptor	BAR64802.1	*Ostrinia furnacalis*	3.00E-44	66
*Gustatory Receptor (GR)*									
GR1	KT381521	134	0	N	gustatory receptor 99	EFA02933.1	*Tribolium castaneum*	5.00E-06	35
GR2	KT381522	287	5	N	gustatory receptor	ABY40623.1	*Tribolium castaneum*	9.00E-71	52
GR3	KT381523	120	2	N	gustatory receptor 6	EFA04712.1	*Tribolium castaneum*	1.00E-30	48
GR4	KT381524	216	5	N	gustatory receptor	ABY40595.1	*Tribolium castaneum*	2.00E-06	33
GR5	KT381525	77	0	N	gustatory receptor for sugar taste 43a-like	XP_001813898.1	*Tribolium castaneum*	3.00E-17	49
GR6	KT381526	183	3	N	putative gustatory receptor 28b	XP_001813096.2	*Tribolium castaneum*	5.00E-08	38
GR7	KT381527	113	1	N	gustatory receptor candidate 10	CAL23143.2	*Tribolium castaneum*	2.00E-59	82
GR8	KT381528	118	2	N	gustatory receptor 2 isoform X1	XP_008191523.1	*Tribolium castaneum*	2.00E-68	82
GR9	KT884512	81	1	N	gustatory receptor 102	EFA02935.1	*Tribolium castaneum*	2.00E-04	29
GR10	KT884513	79	1	N	gustatory receptor	ABY40593.1	*Tribolium castaneum*	4.00E-04	33

Genes without accession number represent that the gene fragments obtained in this study were less than 200 bp in length. Gene fragments less than 200 bp are unable to be deposited in the GenBank, and thus no accession numbers were provided for these genes

### OBPs

We identified 26 different transcripts encoding candidate OBPs in *C. bowringi*, which is less than that in *D. ponderosa*e (31), but more than that in *I. typographus* (15), *A. corpulenta* (15), and *D. valens* (21). The results of the sequence analysis revealed 23 transcripts with a full-length open reading frame (ORF) with predicted signal peptide sequences, and CbowOBP3, 4, 24 corresponded to a partial sequence that encoded amino acids from 89 to 113. Except for CbowOBP18, the other 25 CbowOBPs identified were similar to known coleopteran OBPs (Table 2). Among the 26 CbowOBPs, CbowOBP5 showed the highest expression level (RPKM = 18323.68) (Additional file 4: Table S1)

**Fig. 2:**
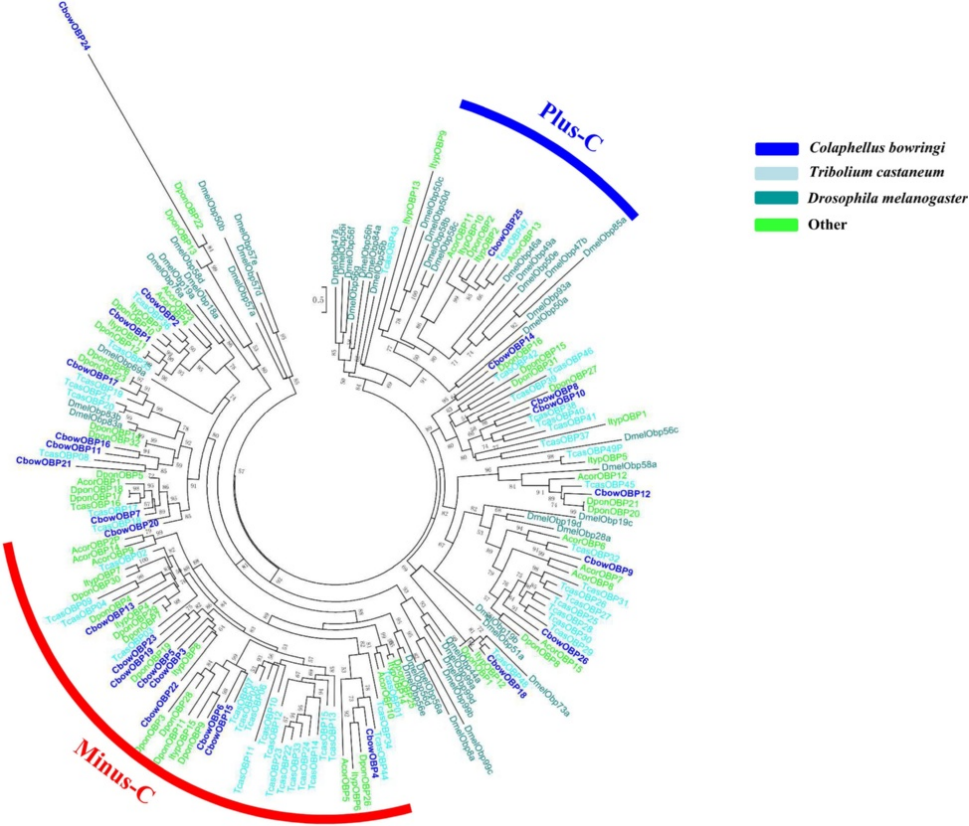
Phylogenetic tree of insect OBP. The *C. bowringi* translated genes are shown in blue. Amino acid sequences used for the tree are given in Additional file 6: Table S2. Bootstrap values greater than 50 % are shown. The Plus-C subfamily is marked in blue, and the Minus-C subfamily is marked in red

A phylogenetic tree of the OBPs was constructed using the protein sequences from *C. bowringi*, *T. castaneum*, *D. ponderosae*, *I. typographus*, *A. corpulenta,* and *Drosophila melanogaster* (fruit fly) (Fig. 2). As previous reports [4, 40–42] and our results, 23 full-length CbowOBPs could be divided into three groups: Minus-C OBPs (CbowOBP5, 6, 11, 13, 15, 16, 19, 20, 21, 22 and 23), Plus-C OBPs (CbowOBP18 and 25), and the remainder Classic OBPs.

### CSPs

**Fig. 3:**
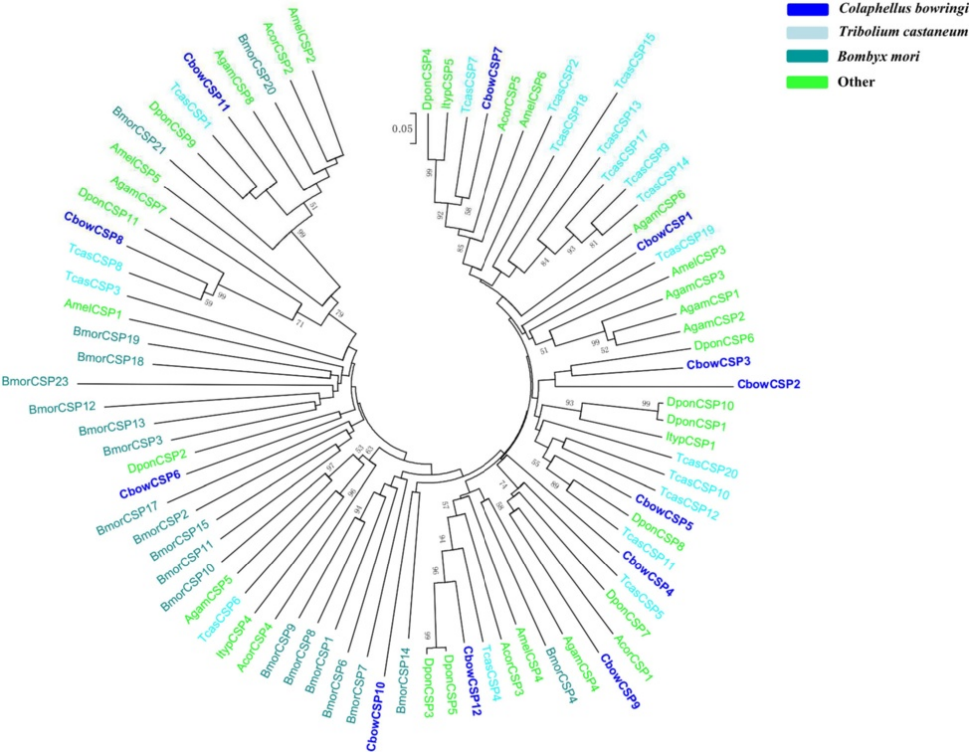
Phylogenetic tree of insect CSP. The *C. bowringi* translated genes are shown in blue. Amino acid sequences used for the tree are given in Additional file 6: Table S2. Bootstrap values greater than 50 % are shown

In total, 12 different transcripts encoding candidate CSPs with four conserved cysteine profiles were obtained in *C. bowringi* through bioinformatic analysis, which included 11 sequences predicted to be full length and 8 with a signal peptide (Table 2), with CbowCSP3 harbouring the highest expression level (RPKM = 4155.27) (Additional file 4: Table S1). The phylogenetic tree revealed two branches with high bootstrap values: CbowCSP8 with TcasCSP8 and DponCSP11, and finally CbowCSP11 with AgamCSP8, BmorCSP20, and BmorCSP21 (Fig. 3).

### SNMPs

Four SNMP homologs with full-length ORFs were also obtained from the *C. bowringi* transcriptome. This number is consistent with *D. valens*, but is greater than that in other previously studied coleoptera insects (Fig. 1). The Blastx results demonstrated that CbowSNMPs encoding proteins harboured a 42–59 % identity to those of other reported insects (Table 2). The RPKM results showed that CbowSNMPs displayed the highest expression level (RPKM = 90.28) (Additional file 4: Table S1). Based on the phylogenetic analysis, we found that CbowSNMP1a and CbowSNMP1b clustered with the coleoptera SNMP1 group, while CbowSNMP2 and CbowSNMP3 clustered with high support with DponSNMP2 and ItypSNMP2Fix, respectively (Additional file 5: Figure S4).

### ORs

Forty-three different transcripts for candidate ORs were identified based on the antennal transcriptome data for *C. bowringi*, among which 20 sequences contained a full-length ORF that encoded 363 to 479 amino acids. We identified one OR sequence that shared a high level of identity with the conserved ORco proteins of other insect species and labelled it CbowORco. The amino-acid sequence of CbowORco shared 92 % identity with the co-receptor of *Ambrostoma quadriimpressum* (leaf beetle) (AJF94638.2). More than 80 % of the CbowORs were highly divergent, and had low levels of identity (21–40 %) with other reported insect ORs. Based on prediction and comparison with other insect ORs [20, 22], we found full-length CbowORs had 3 to 8 TMD (transmembrane domains) (Table 3).

**Fig. 4:**
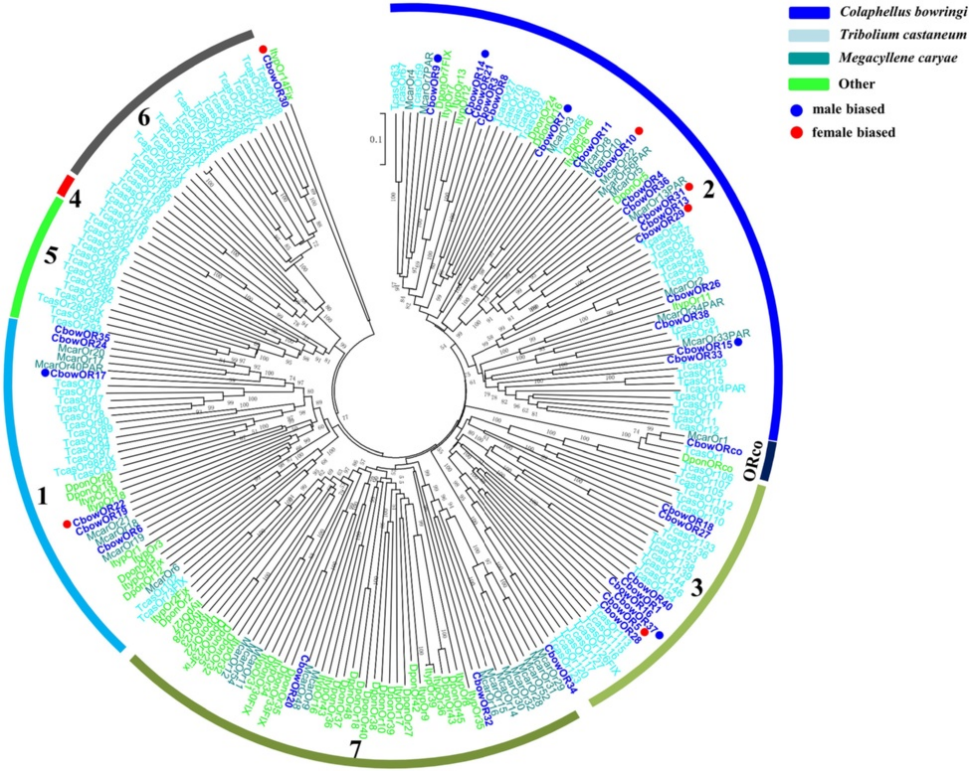
Phylogenetic tree of insect OR. The *C. bowringi* translated genes are shown in blue. Amino acid sequences used for the tree are given in Additional file 6: Table S2. Bootstrap values greater than 50 % are shown

A phylogenetic analysis was conducted using a data set containing the sequences of the 36 ORs longer than 160 amino acids in *C. bowringi* and 192 ORs from four other coleopteran species (Fig. 4). The OR sequences were clustered into several subgroups according to previous studies. CbowORs were only present within the previously defined coleopteran OR subgroups 1, 2, 3, and 7 as well as the ORco subgroup. We found that 6 CbowORs (CbowOR6, 17, 19, 22, 24 and 35) and a functionally characterized McarOR20 [43] were clustered in subgroup 1. A total of17 CbowORs (OR3, 4, 7–11, 13–15, 21, 26, 29, 31, 33, 36 and 38) and 2 functionally characterized McarORs (OR3 and 5) [43] belong to subgroup 2 (Fig. 4).

**Fig. 5:**
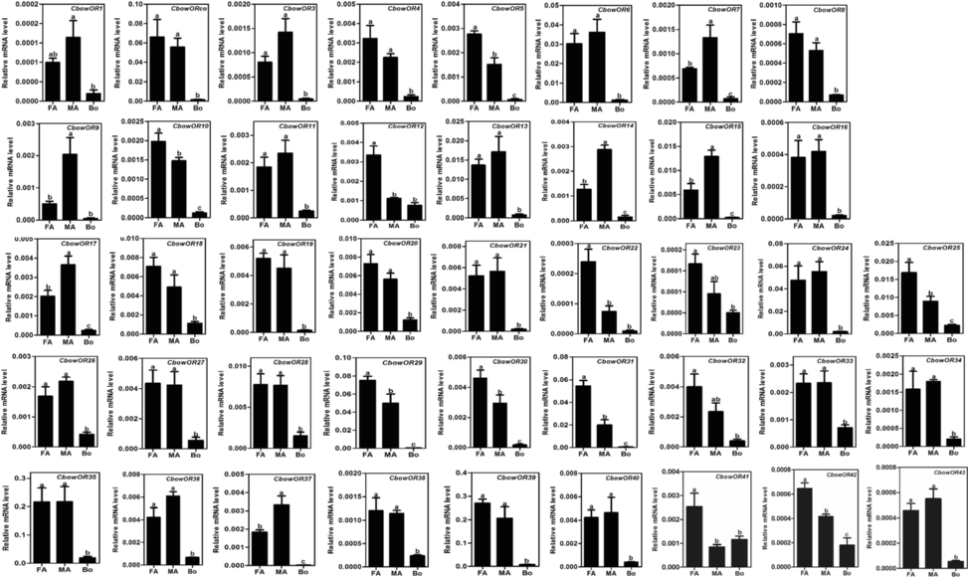
Relative expression levels of all ORs in adult antennae and whole body, using qPCR. FA, female antennae; MA, male antennae; Bo, whole insect body (without antennae). The relative expression level is indicated as mean ± SE (*N* = 3). Different capital letters mean significant difference between tissues (*P* <0.05, ANOVA, LSD)

The transcriptional profiles of CbowOR genes were characterized using qPCR, and the results revealed that all of the 43 CbowORs displayed predominately antenna linked or otherwise biased expression levels. Although we did not identify apparent sex-specific genes in these *C. bowringi* olfactory receptors, there were six (*CbowOR7*, *9*, *14*, *15*, *17* and *37*) and10 (*CbowOR5 10*, *12*, *22*, *25*, *29*, *30*,*31*, *41* and *42*) with significantly higher expression in the male and female antennae, respectively (Fig. 5).

### IRs and GRs

**Fig. 6:**
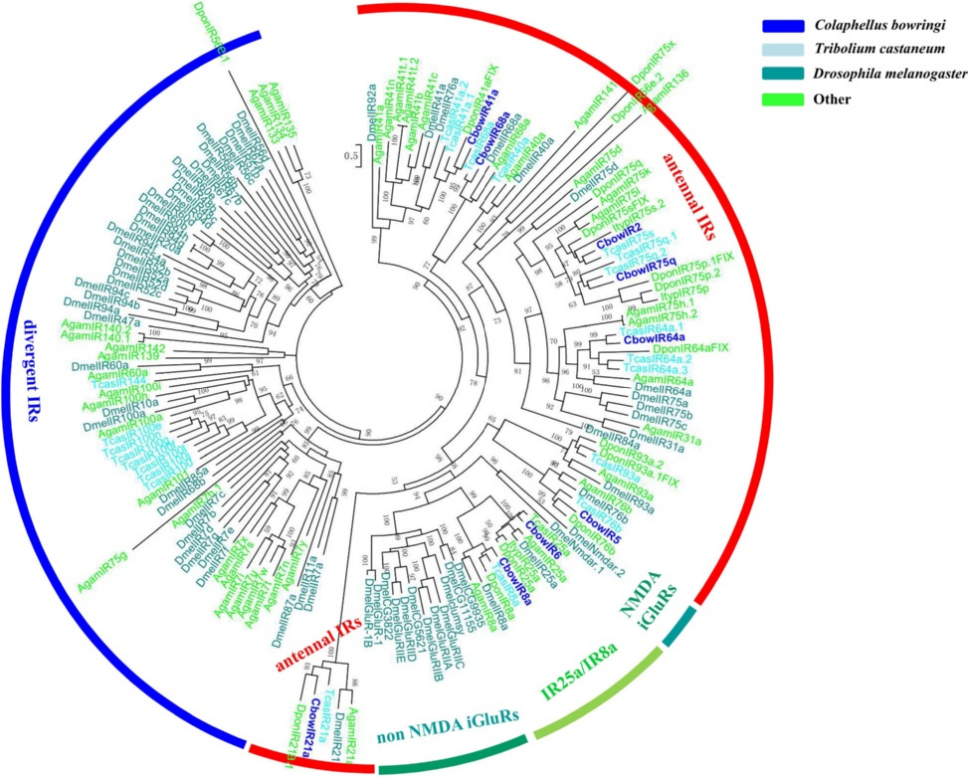
Phylogenetic tree of insect IR. The *C. bowringi* translated genes are shown in blue. Amino acid sequences used for the tree are given in Additional file 6: Table S2. Bootstrap values greater than 50 % are shown

In total, we identified nine IR and ten GR candidates in *C. bowringi*, which is similar to that reported in other recent antennal transcriptomic studies of coleoptera insects [20, 22] (Fig. 1). Only four of these likely represented a full-length ORF (CbowIR2, 5, 6 and 8a), among which we also found three TMDs. The RPKM results showed that CbowIR6 (RPKM = 74.73) and CbowGR1 (RPKM = 53.07) displayed the highest expression levels (Additional file 4: Table S1). According to the phylogenetic tree of the IRs from *D. melanogaster* and various coleopterans, we observed all nine CbowIRs were clustered into antennal IRs and IR25a/IR8a clades (Fig. 6).

## Discussion

Compared to dipterans and lepidopterans, the molecular basis of chemoreception in coleopterans is relatively poorly understood. In the current study, we sequenced and analyzed the transcriptome of antennae from *C. bowringi*. Among the 41,761 unigenes identified, only 45.26 % gene translations shared significant similarity with entries in the NCBI non-redundant (nr) protein database, and only 33.87 % could be annotated to one or more GO term, which is similar to that reported in other coleopteran species [20–22], indicating that a large number of *C. bowringi* genes are non-coding or homologous to genes that do not have any GO term, or perhaps some are *C. bowringi*-specific or fast-evolving genes. Importantly, we identified 104 novel chemosensory genes in *C. bowringi*. Our results not only establish a means to further elucidate the molecular mechanisms of chemosensation, but also provide insight into insect physiology and the development of additional pest control strategies [44].

The total number (104) of chemosensory transcripts identified in *C. bowringi* is different from what has been reported in *D. ponderosae* (111) and *I. typographus* (80), This phenomenon may be due to the evolution of divergent physiological behaviours (such as: herbivory, mating, and oviposition) of different insects during the process of adaptation to various environments [45–47]. Specific environments might lead to divergent evolutionary trajectories of the same ancestral chemosensory genes, resulting in different functional genes among species.

In total, 26 OBPs were identified in the antennal transcriptome of *C. bowringi*. This is close to the number of OBPs in the antennae of *D. ponderosae* (31) and *D. valens* (21), however less than in *T. castaneum* (49). The number of CbowCSPs (12) is similar to *D. ponderosae* (11) while less than *T. castaneum* (20). Previous studies showed that some insect OBPs and CSPs are expressed primarily or exclusively in non-antennae tissues or in larvae [23, 48–50], thus we may not have obtained these types of genes.

Currently, the general mechanism of insect SNMP function is still poorly understood. While DmelSNMP1 is essential for the detection of the pheromone (Z)-11-octadecenyl acetate (a volatile male-specific fatty-acid-derived pheromone) in *D. melanogaster*, and it is thought that SNMP acts in concert with odorant receptors to capture pheromone molecules on the surface of olfactory dendrites [51, 52]. In this study, SNMP transcripts were identified in *C. bowringi* (4) and were found to be more numerous than those in the *T. castaneum* genome (2). The expression of antennal SNMPs in *C. bowringi*, similar to what was previously reported for other known coleopteran insects, suggests that SNMPs in coleopteran insects may have same role as in *D. melanogaster*.

In comparison with the lepidopterans, although the coleopterans ORs have been focused on in recent years [20–22, 43, 53], species richness and function analyses are still lacking. For this reason, it is necessary to identify additional coleopteran ORs to further elucidate the mechanisms of coleopteran chemosensation. In insects, gene duplications and deletion events may be the major contributors to high levels of diversity in OR genes and variability in gene number among species. Forty-three ORs were first identified in the antennal transcriptome of *C. bowringi*, which is less than the number of ORs in the complete genome of *T. castaneum* (341). However, it is same as the number of ORs identified in the antennal transcriptome of *I. typographus* (43) and *A. corpulenta* (43), suggesting we may missed some larvae-biased ORs or those with lower expression levels. Remarkably, similar to what has been observed in *T. castaneum*, *M. caryae,* and *A. corpulenta*, a species-specific expansion of ORs (CbowOR1/5/16/28/37/40 and 3/8/14/21) was also found in *C. bowringi*, which may reflect that these distinct species inhabit different ecological niches. *M. caryae* was the first beetle in which the function of the ORs was characterized [43]. For this reason, we are only able to speculate on the possible functions of CbowORs by examining those of the orthologous McarORs. McarOR3 can bind the pheromone component (S)-2-methyl-1-butanol and additional structurally related chemicals using functional analysis in vitro. CbowOR7 displayed a male-biased transcriptional profile characteristic and could be clustered into the same subgroup with McarOR3, indicating that it may have a similar function to McarOR3 as well as other lepidopteran pheromone receptors (PRs) [54–57]. In total, we identified 6 (*CbowOR7*, *9*, *14*, *15*, *17* and *37*) and 10 (*CbowOR5*, *10*, *12*, *22*, *25*, *29*, *30*, *31*, *41* and *42*) genes with significantly higher expression levels in male and female antennae, respectively. Based on previous studies of the insect OR functions [57–60], the male-biased CbowORs may be involved in the detection of the sex pheromone or other male-specific behaviours, while female-biased CbowORs may detect odours critical to female behaviour, such as oviposition cues or male-produced courtship pheromones. The sex-specific functions of these CbowORs need to be further investigated in the future.

Furthermore, we identified 9 IRs from the antennal transcriptome assembly in *C. bowringi*, which is fewer than that in *T. castaneum* (10) and *D. ponderosae* (15). This may be due to the possibility that some transcripts were missing from our antennal transcriptome. Like ORco, both IR8a and IR25a were thought to act as co-receptors since they are co-expressed along with other IRs [61]. Sequence alignments and the phylogenetic tree revealed that CbowIR8a and CbowIR6 (25a) belong to the co-expression IR group. To date, multiple GRs have also been identified in different insect species [20, 62–65]. While only ten CbowGRs were found in *C. bowringi*, this was expected since GRs are primarily expressed in gustatory organs, such as the proboscis and maxillary palps, rather than the antennae [65–67].

## Conclusions

In conclusion, we identified an extensive set of candidate genes that may be related to odorant perception of *C. bowringi* by analyzing transcriptomic sequence data. As the first step towards understanding gene functions, we conducted a comprehensive and comparative phylogenetic analysis and examined OR gene transcription patterns, some of which were sex-biased. Further analysis is needed to explore the function of these genes using integrated functional studies.

## Methods

### Insect rearing and collection

*C. bowringi* were collected in April 2015 from a *B. campestris* field in the Pollution-Free Planting Base of Huaibei City, Huaibei, China. The field studies did not involve endangered or protected species, and no specific permissions were required for these research activities at these locations. Specimens were separated into females and males, and were reared on fresh leaves of *B. campestris*. The rearing conditions were 25 °C ± 1 °C, a 12 h light : 12 h dark photoperiod, and 70 ± 10 % relative humidity [68]. For transcriptome sequencing, the antennae of 800 adults (400 males and 400 females) were collected. For the expression study of different tissues, 150–200 female antennae (FA), 150–200 male antennae (MA), and 10–15 whole insect body without antennae (Bo) were also collected. All samples were immediately frozen in liquid nitrogen and stored at −80 °C until use.

### cDNA library construction

Total RNA was extracted using TRIzol reagent (Invitrogen, Carlsbad, CA, USA), cDNA library construction and Illumina sequencing of the samples were performed at Novogene Bioinformatics Technology Co., Ltd., Beijing, China. The mRNA was purified from 3 μg of total RNA using oligo (dT) magnetic beads and fragmented into short sequences in the presence of divalent cations at 94 °C for 5 min. Then, the first-strand cDNA was generated using random hexamer-primed reverse transcription, followed by synthesis of the second-strand cDNA using RNaseH and DNA polymerase I. After the end repair and ligation of adaptors, the products were amplified by PCR and purified using the QIAquick PCR Purification Kit (Qiagen, Valencia, CA, USA) to create a cDNA library, which was assessed on the Agilent Bioanalyzer 2100 system.

### Clustering and sequencing

Clustering of the index-coded samples was performed on a cBot Cluster Generation System using TruSeq PE Cluster Kit v3-cBot-HS (Illumina) according to the manufacturer’s instructions. After cluster generation, the libraries were sequenced on an Illumina HiSeq™ 2500 platform and paired-end reads were generated.

### *De novo* assembly of short reads and gene annotation

Clean short reads were obtained by removing those containing an adapter or poly-N and of low quality from the raw reads. Transcriptome *de novo* assembly was carried out with the short read assembling program Trinity (r20140413p1) [69, 70] with min_kmer_cov set to two by default and all other parameters also set as default. The resulting sequences were the unigenes. The unigenes larger than 150 bp were first aligned by Blastx to protein databases, including Nr, Swiss-Prot, KEGG, and COG (E-value < 10^−5^), retrieving proteins with the highest sequence similarity with the given unigenes along with their protein functional annotations. Then, we used the Blast2GO program [71] to obtain a GO annotation of the unigenes, and GO functional classification with the WEGO software [72].

### Expression abundance analysis of the Unigenes

The expression abundance of these unigenes were calculated based on the reads per kilobase per million mapped reads (RPKM) method [73], using the formula: RPKM (A) = (10,00,000 × C × 1000)/(N × L), where RPKM (A) is the abundance of gene A, C is the number of reads that uniquely aligned to gene A, N is the total number of reads that uniquely aligned to all genes, and L is the number of bases in gene A. The RPKM method was able to eliminate the influence of different gene lengths and sequencing discrepancies in the calculation of expression abundance.

### RNA isolation and cDNA synthesis

Total RNA was extracted with the SV 96 Total RNA Isolation System (Promega, Madison, WI, USA) following the manufacturer’s instructions, in which a DNaseI digestion was included to avoid contamination of genomic DNA. RNA quality was checked with a spectrophotometer (NanoDrop^TM^ 1000, Thermo Fisher Scientific, USA). The single-stranded cDNA templates were synthesized from 1 μg of total RNA from the various tissue samples using the PrimeScript™ RT Master Mix (TaKaRa, Dalian, China).

### Sequence analysis

The open reading frames (ORFs) of the chemosensory genes were predicted using ORF finder (http://www.ncbi.nlm.nih.gov/gorf/gorf.html). The similarity searches were performed with the NCBI-BLAST network server (http://blast.ncbi.nlm.nih.gov/). Transmembrane domains of both CbowORs and CbowIRs were predicted with the TMHMM Server Version 2.0 (http://www.cbs.dtu.dk/services/TMHMM). Putative N-terminal signal peptides of CbowOBPs and CbowCSPs were predicted by Signal IP 4.1 (http://www.cbs.dtu.dk/services/SignalP/) [74].

### Nomenclature of all genes

We adopted nomenclature for the CbowORco, CbowIRs and CbowSNMPs that are analogous to those deposited in GenBank (http://www.ncbi.nlm.nih.gov/genbank/). Based on previous studies, CbowOBPs were divided into three groups [3, 4]: Classic OBPs, characterized by 6 cysteine residues at conserved positions; Plus-C OBPs, which have 4–6 additional cysteines and one characteristic proline; and Minus-C OBPs, which are missing cysteine residues, generally C2 and C5. The rest of the chemosensory genes of *C. bowringi* were named based on their order in the antennal transcriptome data.

### Phylogenetic analysis

The phylogenetic trees were reconstructed for the analyses of CbowOBPs, CbowCSPs, CbowSNMP, CbowORs, and CbowIRs, using these genes (the signal peptides of sequences were removed from OBPs and CSPs) as well as the sequences in other insects. The OBP data set contained 26 sequences from *C. bowringi* and 150 from other insects. The CSP data set contained 12 sequences from *C. bowringi* and 72 from other insects. The SNMP data set contained 4 sequences from *C. bowringi* and 17 from other insects. The OR data set contained 36 sequences from *C. bowringi* (amino acids > 160 aa), and 192 from other insects. The IR data set contained 9 sequences from *C. bowringi* and 108 from other insects. The amino acid sequences of the genes used for phylogenetic tree construction are listed in Additional file 6: Table S2. Amino acid sequences were aligned with ClustalX 1.83 [75] and unrooted trees were constructed with MEGA5.0 [76] using the neighbour-joining method, with Poisson correction of distances (CSP, SNMP, and OR) and FastTree 2.1.7 [77] using maximum-likelihood method (OBP and IR). The species phylogenetic tree was constructed based on the alignment result of cytochrome oxidase subunit I (*COI*) genes, from different species (*T. castaneum*: KJ003352.1, *I. typographus*: KF846151.1, *D. ponderosae*: JQ308497.1, *D. valens*: EU404100.1 and *A. corpulenta*: the reference [19]) using MEGA5.0.

### Quantitative real time-PCR validation

The expression profiles of 43 OR genes were analyzed using quantitative real time-PCR (qPCR) experiments. The qPCR was performed on an ABI 7300 (Applied Biosystems, Foster City, CA, USA) using a mixture of 10 μl 2 × TransStart Top Green qPCR SuperMix (TransGen Biotech, Beijing, China), 0.4 μl of each primer (10 μM), 2.5 ng of sample cDNA, and 6.8 μl sterilized ultrapure H_2_O. The reaction programs were 30 s at 94 °C, 40 cycles of 94 °C for 5 s and 60 °C for 31 s. This was followed by the measurement of fluorescence during 55–95 °C melting curve in order to detect a single gene-specific peak and to check the absence of primer dimer peaks. A single and discrete peak was detected for all primers tested. Negative controls were non-template reactions (replacing cDNA with H_2_O). The results were analyzed using the ABI 7300 analysis software SDS 1.4. The qPCR primers (Additional file 7: Table S3) were designed using Beacon Designer 7.9 (PREMIER Biosoft International, CA, USA).

According to a previous study [68], expression levels of these genes were calculated relative to the two most stable reference genes *CbowEF1α* and *CbowACT1* using the Q-Gene method in Microsoft Excel-based software of Visual Basic [78, 79]. For each sample, three biological replications were performed with each biological replication measured in three technique replications.

### Statistical analysis

Data (mean ± SE) form various samples were subjected to a one-way nested analysis of variance (ANOVA) followed by the least significant difference test (LSD) for mean comparison using SPSS Statistics 17.0 (SPSS Inc., Chicago, IL, USA).

### Supporting information

All the Illumina sequencing data are available from the SRA database (accession number: SRX1309381), and all of the chemosensory genes of *Colaphellus bowringi* were submitted to the GenBank (accession numbers: KT381483 - KT381577 and KT884510 - KT884516).

## Additional files

Additional file 1: Figure S1. Distribution of unigene size in the *C. bowringi* transcriptome assembly. (TIF 190 kb) Additional file 2: Figure S2. Percentage of homologous hits of the *C. bowringi* transcripts to other insect species. The *C. bowringi* transcripts were searched by Blastx against the non-redundancy protein database with a cutoff E-value 10^−5^. Species that have more than 1 % matching hits to the *C. bowringi* transcripts are shown. (TIF 221 kb) Additional file 3: Figure S3. Gene ontology (GO) classification of the *C. bowringi* transcripts with Blast2GO program. (TIF 1678 kb) Additional file 4: Table S1. The Reads Per Kilobase per Million mapped reads (RPKM) values of CbowOBPs, CSPs, SNMPs, IRs and GRs. (XLSX 10 kb) Additional file 5: Figure S4. Phylogenetic tree of insect SNMP. The *C. bowringi* translated genes are shown in blue. Amino acid sequences used for the tree are given in Additional file 6: Table S2. Values at the nodes are results of bootstrap with 1000 replicates and values greater than 50 % are shown. (TIF 141 kb) Additional file 6: Table S2. Amino acid sequences of *C. bowringi* and other insect used in phylogenetic analyses. (PDF 814 kb) Additional file 7: Table S3. Primers used for qPCR. (XLS 29 kb)

## Abbreviations

RNA-seq: High-throughput mRNA sequencing
RPKM: Reads per kilobase per million mapped reads
GO: Gene ontology
KEGG: Kyoto encyclopedia of genes and genomes
COG: Clusters of orthologous groups
OR: Odorant receptor
GR: Gustatory receptor
IR: Ionotropic receptor
OBP: Odorant-binding protein
CSP: Chemosensory protein
SNMP: Sensory neuron membrane protein
ORF: Open reading frame
TMD: Transmembrane domain
PCR: Polymerase chain reaction
qPCR: Quantitative real time RT-PCR
cDNA: Complementary DNA
ANOVA: Analysis of variance
SE: Standard error
