# Supplementary figures and images for: Candidate chemosensory genes identified in Colaphellus bowringi by antennal transcriptome analysis
Source: BMC Genomics. 2015 Dec 2;16:1028. doi: 10.1186/s12864-015-2236-3 (PMC4667470; doi:10.1186/s12864-015-2236-3)

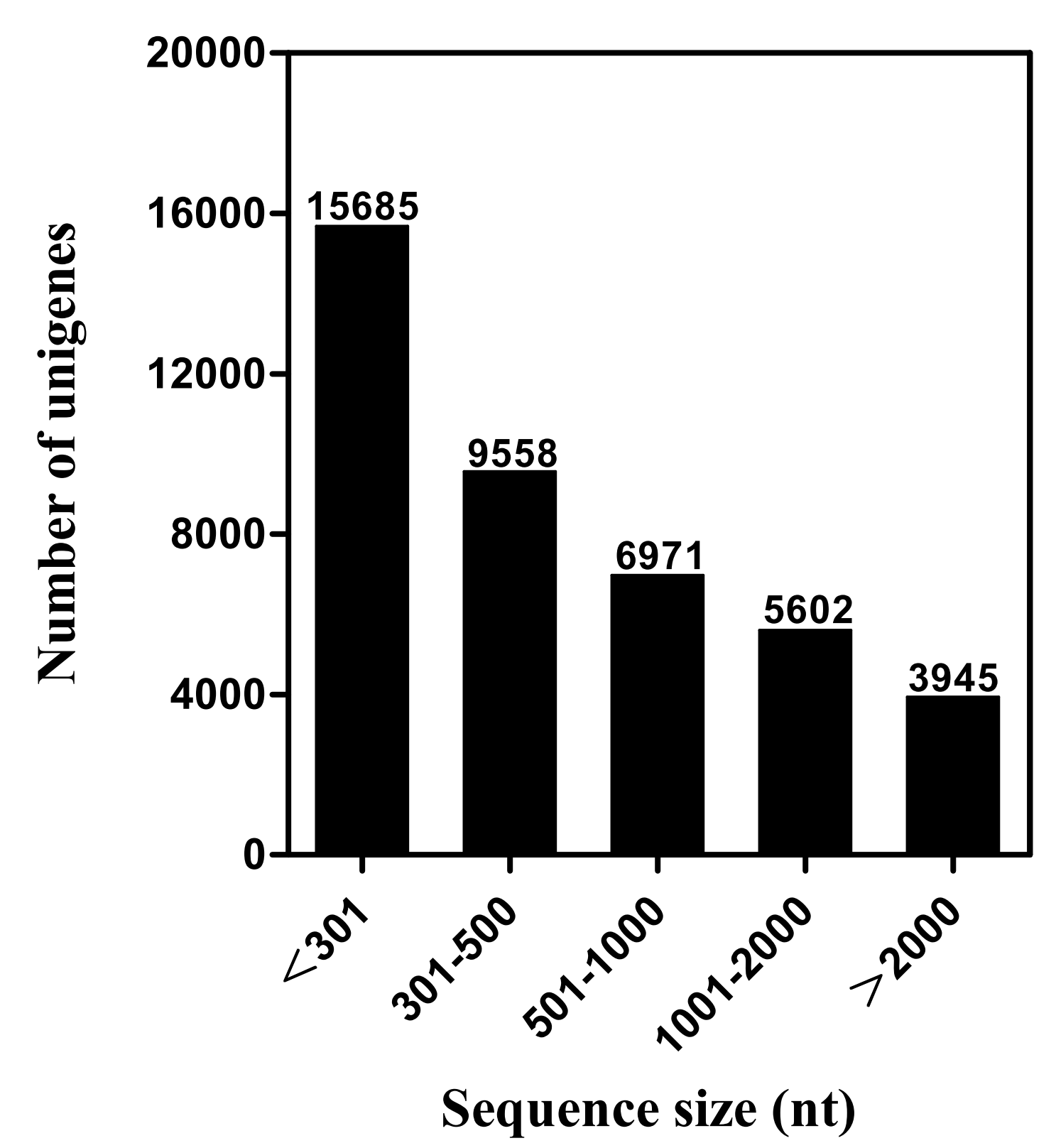

Supplement: Additional file 1: Figure S1. — Distribution of unigene size in the C. bowringi transcriptome assembly. (TIF 190 kb) [file 12864_2015_2236_MOESM1_ESM.tif]

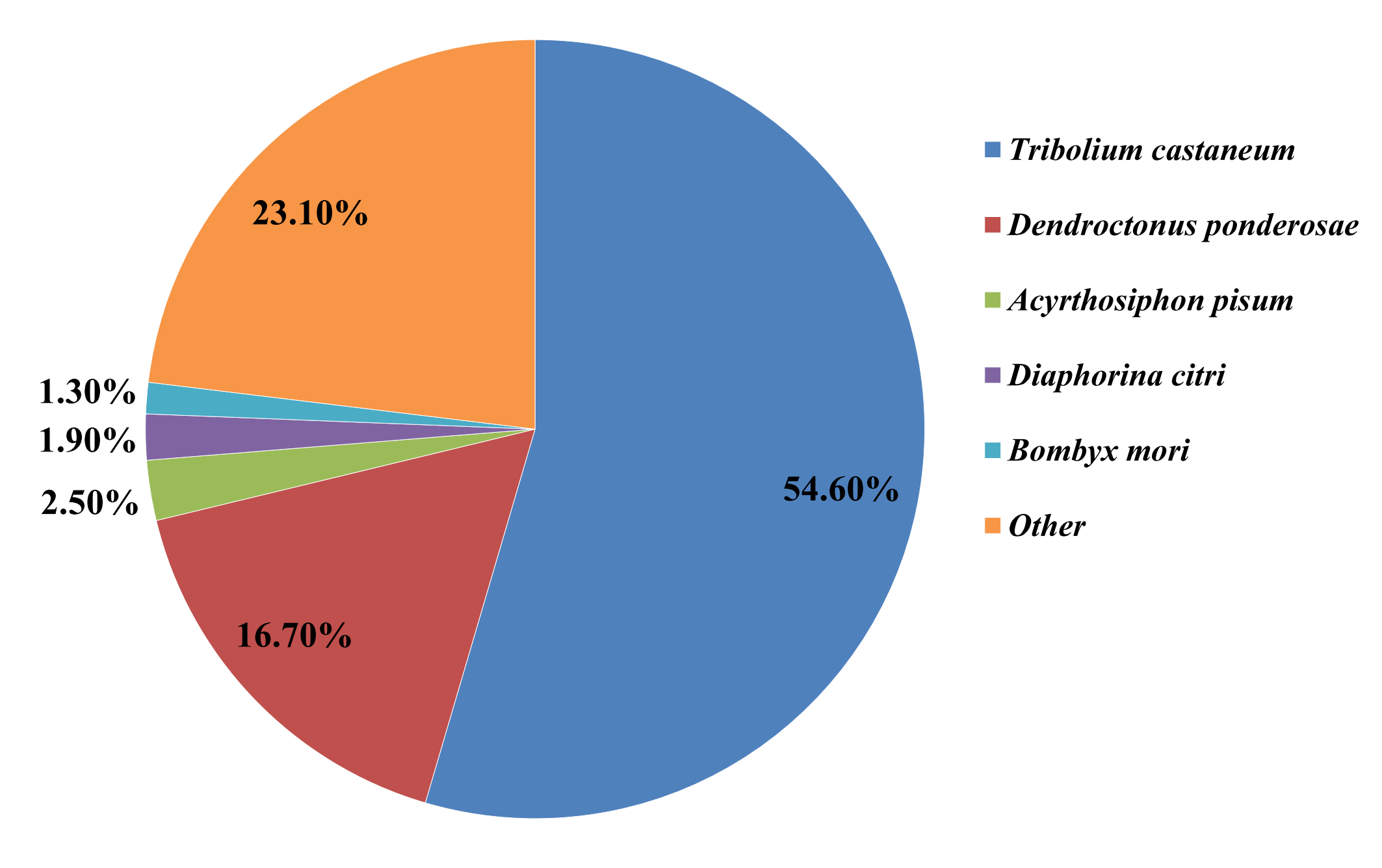

Supplement: Additional file 2: Figure S2. — Percentage of homologous hits of the C. bowringi transcripts to other insect species. The C. bowringi transcripts were searched by Blastx against the non-redundancy protein database with a cutoff E-value 10−5. Species that have more than 1 % matching hits to the C. bowringi transcripts are shown. (TIF 221 kb) [file 12864_2015_2236_MOESM2_ESM.tif]

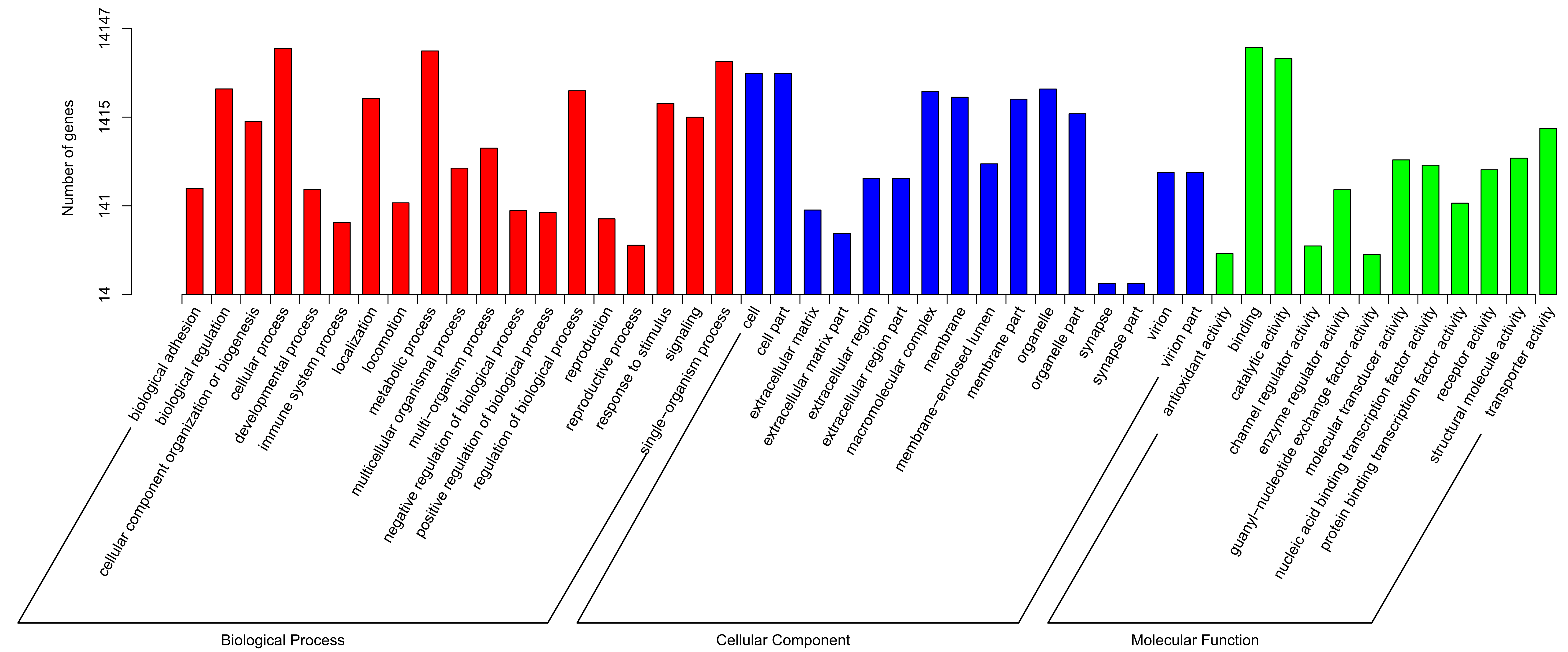

Supplement: Additional file 3: Figure S3. — Gene ontology (GO) classification of the C. bowringi transcripts with Blast2GO program. (TIF 1678 kb) [file 12864_2015_2236_MOESM3_ESM.tif]

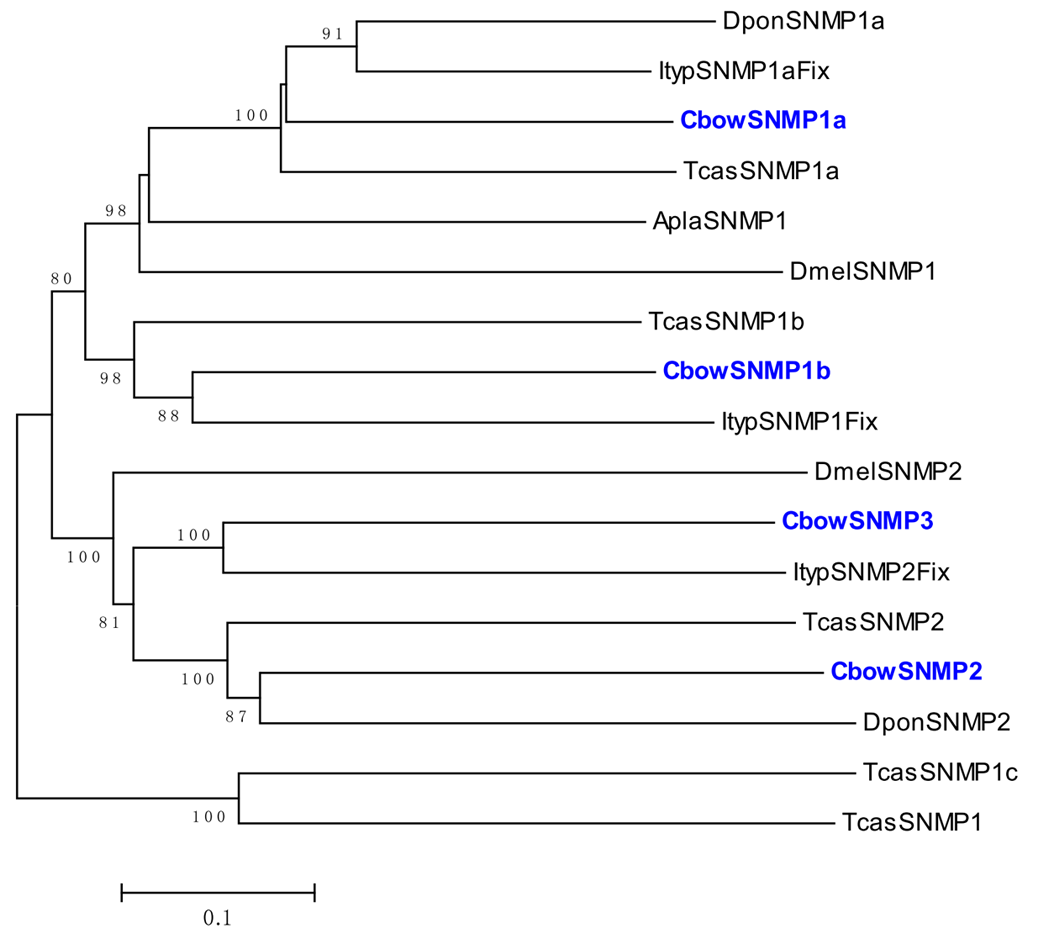

Supplement: Additional file 5: Figure S4. — Phylogenetic tree of insect SNMP. The C. bowringi translated genes are shown in blue. Amino acid sequences used for the tree are given in Additional file 6: Table S2. Values at the nodes are results of bootstrap with 1000 replicates and values greater than 50 % are shown. (TIF 141 kb) [file 12864_2015_2236_MOESM5_ESM.tif]
