# Supplementary material for: Candidate chemosensory genes identified in Colaphellus bowringi by antennal transcriptome analysis
Source: BMC Genomics. 2015 Dec 2;16:1028. doi: 10.1186/s12864-015-2236-3 (PMC4667470; doi:10.1186/s12864-015-2236-3)
Supplement: Additional file 6: Table S2. — Amino acid sequences of C. bowringi and other insect used in phylogenetic analyses. (PDF 814 kb) [file 12864_2015_2236_MOESM6_ESM.pdf]

Additional file 6: Table S2 Amino acid sequences of *C. bowringi* and other insect used in phylogenetic analyses

OBP sequences

>CbowOBP1
ELTEKQMKATKKLIRNTCQNKAKATTEELDAMVKGNFNQGKNAQCYQLCILNTYKLLKS
DNTFDWQAGVNALKANAPERIAGPGSASIKNCKDALKTKDDKCKGATEIAQCIYEDNPE
NYFLP
>CbowOBP2
LNEKQMKAAVKMVRNVCQPKFKATDVDIDKMHKGDWNIDHTAMCYMHCAMNMYKL
MNTDNSFNYQSALAQLNQLPDSYKKATEICMEQCKDSAVTLSDKCISAYELAKCMYFCN
PEKYFLP
>CbowOBP3
MRAAKGDYQDDMKLKKQILCFNKKVGLQDENGDIVLDVAKSKLFDIVKDEKKTMDILK
KCAVKKDTPENTAFESAKCLHKLAPEEKLVI
>CbowOBP4
MKHREEIGLECLRQVNIQRDTIENAKATLNFPEDRKYKDFLACSYKKQGFQSQDGVILYN
SIKDFLSRYYKRNDLKVMDNCKENIREDHGEMALNALRCIMDNLKNMEEKSRR
>CbowOBP5
KPLSEEGREKAQKINEECAKESGIEEDNLEKILADEFPEDDKMKEHSFCFLTTLGVMDKDG
KIDKDVMTDTLKLFAPEGKEVEIMEKCAVETDDAKETAFAIGKCVHEQVKSV
>CbowOBP6
GQLPEDEKERLRQVHLSCQADSKTYCDEDLLRKLGDNVNNPQVGIHMLCMSVKAGLQE
RNGDLNRSFIKSRIALVTEQAKVDGYVQKCAVKKETPEKTAAMLWLCFVQNGINYYHKL
>CbowOBP7
LSEEMQELADMLHATCVHETGARQDDIENARKGIFAEDEKFKCYIKCIMAQMACIDEDGII
DEDATIAVLPEEYRNQAEPVIKKCGTKKGSNPCENAWLTHKCYQNEAPEDYFLV
>CbowOBP8
VGKFPDGRPYPDGFEDCLKSSNAKLEEVLNKPKANISEEVYCFFKCLSERVGFIDQQGNV
HIDKMDVTQIFQGAVEEVPDELKSCLGGVNKVESCQDMSKICECFLKMAP
>CbowOBP9
VRNTAGKITQNESAKKTLGNCKTETGATMADIESLKEKKIPKTKTGRCFMECLFSKAKIM
DNGRFNKKGMVVAFTPALKGDLTKMGKLRELSEVCEKEIGLNKLENCEGGKKIVECVAK
HGKSYGMSFSTTK
>CbowOBP10
EKKLPIEAEECLKITNTDLKDMMAHPKEMSESHYCFFKCIFEKRGIINKVDGTVVPDVLDD
IKDVAVLQVASEEKLAELKKCMADVEKIEKCTDMENFRVCFDKLMS
>CbowOBP11
YITEEGWGEPLIALANSLHNKCVPITGVTQASIDQVKEGNFIEDEKMKRYVLCLWLVSEVI
SEKFELNTEIFKLLPKKLQDGHNIIGCTKKINGTDVSELYEKTYSVTKCIQKANPDEFIMF
>CbowOBP12
LECGIAKANRNEIRQALSMCVKNNDTLEDILEMSSLSSSTTSSPTEDSDDEDSQEDTIKSTS
STTKSPRRIKSSRIKRARSFSNTKQYASKATERNREESNNSNNTNNKIDNLKDAQKDDSED
DNEVSQETPKKRQDMSENCIVHCVLEHLNLTDETGLPDHSKLSEELLKTASGRELRNFLQ

ESTDECFQEVNEENDLDSCSYTTKLITCLADKGKSNCADWPAGALPF
>CbowOBP13
GVTEEQKKKIESYHKECSKQTGIDEDLVNKARNGQYTDTQILKDYLFCTSKLAGFINDNN
ELQKDVILQKTSVSTKDSAAAQKMFEACAVPQKNGPETSYHVLKCYYEKSGLSLV
>CbowOBP14
APSDSSNSTIKDYCIKEVEISEEKVNKFEKNPDDTPDEDIMCYTHCILVTLGVVDDDGKIIIE
KFTKIFDKYDMECVKKIPKILECTDLINLNKCAATDE
>CbowOBP15
EETEKEKMKRIHEECQSDPATKVEESVLKAAEEGDVDVTKIGPHTLCMNVKVGLQKENG
DIVKDELRAGLRRVPGVDESKIESIVEECGQREGGTAEEAAIKLFQCLQKRSKITHHHHHH
E
>CbowOBP16
YLSEEDYGPKLSAVANKVHNACIKKHAVNEDTIMQVRKGNFVEDELIKKYISCIWLLSTVL
DESGNLNIKIINDLCPPKGKDTLPKIYHDCHAENAGVSQLDEKVYNIMKCWYEKDPELFF
VL
>CbowOBP17
IELPSELQEYVEDLHKICVTKSGISEDDHAAYDVKTNPHDPKLQCYMKCLMLEAKWMNP
QGDIQYDFIIDTSHPQIKDLLVAAINKCRAIDNGANLCEKASNFNFCMYDADPVNWFLI
>CbowOBP18
GKNQTNNCDIPASAPKRIEETINSCQEEIKLAILTEALEALNVNEHIHSRAKRSAFSKDEKRI
AGCLLQCVYRKMNAVNERGFPTAEGLISLYTEGIRHKDYVLATMQAVNHCLYHVQKNHL
TTPQSIDEHGKTCDIAYDVFDCVSEEIGKYCGQTP
>CbowOBP19
TDREDKMKIHNVCVAETGVSQEFIDKMIAGEFCDDTNFKNYLVCFLKNDGVFLDNGELK
ADGANRQIREFADDEDTVSGFMANCAVQMATVEESAFHYSKCMYNTLYG
>CbowOBP20
LTEEQQQIMESLHAECISQTGATEDMIVNARNGDFSEDNKLKCYMKCVFEELGVLDDDG
KVDIDGILAMLPDEYKDVATTVFNKCGTQAGTDVCDAIFQTHKCYYAANSEHYFLP
>CbowOBP21
WMSDDKFGDKLEKMYRLWHDDCMKKTGAPENTVELIRSGIFPDDPRMKAYNRCLYTDV
MDKNARLLPEKLDYYIYPAFGKTGLKMYLDCEEKVKDEANYDDRVYKMQQCIYEANPD
VSFNFF
>CbowOBP22
QMVSPSQLNTILQYHTECREKTKLPNSLVTGLIAGQFPNDPVLKSHLLCVHQKLGVQDAD
GNLRKEFISETLGAVLPASVDSKELLNKCAVQKSSPEDTALDLDRCLYQTVQPMRG
>CbowOBP23
LSEENMKEIEEFQKSCVAEVHTSPDVLSQIMSGDVSDDPKIKAHLLCFAKKAGVMTESGE
TIMDKLKQKLNQYLGSKADGFFEKCNIEITTPEDTAFNVYKCLSEMLQEGK
>CbowOBP24
MNVHAGLQKPNGDIDKDDLRRALSEGIRDVITVDDIVDDCGQRVGSTAEEASVNLFRCIF
GHSNAYVHEWKPSMLRQTSGAEGFFTSSLVLSVILATLSVRLI
>CbowOBP25
YNFEDTEFNQILANDLEDVYSFTYSHPRSRRDDKAVEEDKCHPPRRGRPLCCAEETMRKL
HDDKKEIKRACFKEITGKEKPERPDRHHGPPFDPFSCEKIEQHRRDMMCIQQCVGEKLDY

LDADGKPKPEQFEKYVEGIFEKEDYLLPLKDKIVSVCLDEAKNATEKVSSDPCKSTGLVLE
HCIFINTQLNCPEDQIKDKKMCSKFQDRLRQGFDKRSSPSPEAEDE
>CbowOBP26
KVDPKVIEEIIEEFTETVAKCSDEINPNADDIAALTEMKHIPDSHEGKCMIYCIYRSFDAVEE
DGHVKFEGGMAFLSKIKESDPDMFDKMSAIYKKCTETDYFDKDPCISSANFVSCNIKAGK
EANISSDISSW

>TcasOBP01
EDDDRQETIRQYRDDCIAETKVDPALIDRADNGDFTDDAKLQCFSKCFYQKAGFVSETGD
LLFDVIKDKIPKEANREKALAIIDKCKELKGADSCETVYLVHKCYFLHSYGTDKKTE
>TcasOBP02
LTDEQKEKIKNYHKECSAVSGVSQDVITKARKGEFIEDPKFKEHLFCFSKKAGFQNEAGDF
QEEVIRKKLNAELNDLDATNKLIAKCAVKKDSPQQTAFETIKCYYENTPTHVSLA
>TcasOBP03
LTDEQKSKLEEYSKECLKESKVDESVLKEAEKGVYLDDPKLMNHVYCLVKKINSQKDKG
ELEVTQIKEKLMMQINDEKEVDKLIQLCLVQEKSARYSLGKCEVSS
>TcasOBP04
FNNPEDELRRSAACLEQSKVSSESIKNLQIGNFDDDERLKEYLFCVSKNAGYQDPAGHLQ
HEMIRLRFKGGRYSDDTINEVLQQCGHQKDTPQETAFQFMKCAYQNAFPRNYK
>TcasOBP05
LTKEQIDKLEPISKECRELNGISEDTILKVRRGEAVNEPKLKNHVLCVSKKTGLASETGETN
VEVLRTKLRKVSENDDEVNSIIQKCVVKKSTPEETAFEIFVCLRKVKPNFSPAN
>TcasOBP06
LSEQQTEKLNQLSKECRALTGVSQETITNARNGNFEEDPKLK
LQVLCIGKKVGIMNESSQIDENVLKAKLRKVSDNDEEVNKIYNKCAVKKPAPEETAFETI
KCVMKNKPKFSPVE
>TcasOBP07
LTKEQKEKLDKISKECKNQSGVSQELIDKARTGELINDPKLKAQIYCVSKKAGLATEAGEI
NMDNLKTKLKKVAANDDEVNKIIQKCVVKKPTPEETAFEVYKCLHANKPNFSVVD
>TcasOBP08
KQDFHKKCLASSGANADTIAKVRNGKFSNDPQTQKYFGCMLRSVGVVNQAGQLQVAAL
RKQVPKDMKRDEAMKIYMSCKDKKGANNDETAYLLYKCFWEASPRHVKIDGQ
>TcasOBP09
FSLSNREQAIFLSTYSTCLETSKVDSERALRTASGIIDDEPKLKEFLFCINKQNGVQDDAGN
FVKDAVRKRIEHPLLTDKTMEIIVNKCTRKRETGEETAYQFLKCSYFTIMNEKHQ
>TcasOBP10
QHLTEEQKNNWRKWSNECKVLIGVSQEAINKIRNNEFDSVDDKIKKHGLCFAKKASLAD
SSGNIIINQIKIKLKRVIEDDEEVDRIVTKCTIRKNTPEETTFETFRCLRENSSKFVPV
>TcasOBP11
LENEGQNPDTANCVALGGQRIKDSEIAKMAHCILTKTNLMTDKGTFNSNLLKERLRQSVH
SDELVDKVVMMCTVEKETPLKSAFSGYKCLRYLVPWFPLD
>TcasOBP12
KKCFLAEDTDKLEVMINECKTKTGVPDDILQKARNGEKIDDPKLREHALCMMKKSEMM
NDAGEMQMDKIRARIKHAVSNEAEGTRIMNECAVKKDTPLATAYEMICCLIRNKNSVDE

>TcasOBP13
ETAKEKLRKYSDECKSVSGVSEELLNKVRNHEDVHDPKLDEHGFCILKKAGFMNEAGDI
LADTIKTKLKENSEHPDTVDALVEKCNEKKDTPQHTASHLFTCLVDKKVHSH
>TcasOBP14
RKWFDKDPQDVAKWQKECFEASGVSMESMNKLPNITLSEDPKLGENAFCLLKKLGFISE
DGTLLIEKLRTSLKNQWGDEIANKLVNECARQKSTPQETAHEMFLCIPAKLK
>TcasOBP15
KQQKQDTLDEEKEKMKKWTQECIQESGVTSEILQQLRNQKRVEDPKLKEYTFCTFKKNG
FMNEDGKLQYDVIKSTLMKVSGSEEEANKVVKDCVVEKSTPQDTAFETVDCWYRYKKN
>TcasOBP16
EEMQELVNQLHSTCVAETGVSEDLINKVNSDKVMIDDEKLKCYIKCLLTETGCISDDGVV
DVEATIALLPEDMKAKTTPVIRSCGAKMGANPCESAWLTHKCYLETSPADYVLI
>TcasOBP17
EEMQELANTLHATCVDETGVSEDAIESARKGNFAPDDKLKCYMKCIMEQMACIDDEGIID
VEATIAVLPEEYQAKAEPIVRKCGTKIGANACDNAFLTNKCWYEEDPEVSLQLN
>TcasOBP18
IEMDDDMKELINNLHNTCTGETGATDDQIENARKGNFAEDDSFKCYFKCVFDQMGCMTD
DGKVDSEAVIAVMPPELADKIASTVRGCTEVGANPCETAWLANKCYQKSNPDVSKVSSN
VRSD
>TcasOBP19
EIVVPDDLKDYINELHDHCLKEMGLTEGDHKNYNIHVKDPKMMCYMKCLMTTSKWMN
MDESIQYDFILSSVHPAVKNILLPALDKCRDIPKGTMECEKAYNFNMCLFNADPENWFFI
>TcasOBP20
EIDGYYDICYKQIGLTKDDLKAYKIGDRDPKIMCFMKCVFVEAKWMDENENLQYDYIKN
TIHHSIRHITLPELENCGKKAEGDKCEKSFSFFNCMNKAEPEDWVLIQ
>TcasOBP21
EIDEYFEQCFEPNGVTMDDIKAYKMGDKDPKIMCFMRCLFVSGKWMDENENMQYDYIK
ETIHHAIRHITIPELENCGKEAQTGDKCEKSFNFFMCMNRAEPEV
>TcasOBP22
EEDNVGKIESVEKKCQEKTGVSEESLQKIMRLEEVDDPLVKENALCTLKAYGVMDDDGN
IFPDKFEEKLKPEIGADEAKRVAEKCAVKKDSPEETAHQTLWC ATEENALTDTSQEQ
>TcasOBP23
DEDNLNTENVQSIEEDCQKETGVSDESLQELSETGDSDDPLVKKNALCILKAYGVIDDQG
EISEDKLEEKLEPDRGKEEAEKVAKSCAVKKDSPEETAHEALLCMQQKSQK
>TcasOBP24
TPSLDDFKKVQKDCQKKTGVSDESINKVNNLEPVCDDLLLQENALCILKTYEVMDEEGKI
CPDKLMEVLEPKFGKEKAEKLIEKCTLEKDTPQLLAHATLFCLSVQKYVV
>TcasOBP25
QFLQKIKKVSEDCIAETQATKNDIKTLLEHKIPDSHEGKCMIFCFHKHFQIQNEDGSLNKVA
AISLLEPIKDHSQDIYDKVVKIFNTCFDSAERDDDSCIYASNLAECAIRESKSVSVQ
>TcasOBP26
VDQEFVEKFLQKMEKIGEECAEETHATSDDIADLIEQRDPKTHEGKCLIFCYHKKFNTMK
EDGSLDKVGSVLALEEVRDADFELYKNILTIFVTCGDKAKIYDDPCETATALTMCGRDEAK
AVSWAYFA

>TcasOBP27
FLEKMQEFGAQCAEETDATSDDIAELIARKLPPSTHEGKCMIFCMQKKFNMMKENGGIDR
AGAIAALKPLQKADPELHQKVLKIFVTCGMRVKPSPDPCDTATELALCGKKEAEAVFCRL
GWKTLS
>TcasOBP28
QDFIDKFVAKVKSIGETCVPETNASKDDISSLLAHKMPDSHEGKCLIFCFHKQFQIQNDDG
SINREGAIKALEPLKADDAELYEKVISIFKKCESTPVDGDSCLYAASLAECAVKEGRAVSFW
KNTNFKLI
>TcasOBP29
MDESFLQQTRDRVKAIVKECVTEEKATDSDFDDIMALKIPTSHEGKCVFFCSHKKFNMQH
PDGSINKEGALDTFEVVKDVDAEFHDKVITVYNHCLSTPVDPDPCVYSVNLFQCFMKEA
KAVRK
>TcasOBP30
IDKEFVQELRQKLRSHVEACAKEVNAGPDDVSAIFAHKLPATHEGKCIFFCMHKLYNAQN
EDGSLNMAGALANLELIKDMDPDVYTKVSTSFKNCESAPFDSDPCLYAANLVTCIVKEGR
AVSNNFSGFFF
>TcasOBP31
GVSIILDPKFLEKLTQEVQAVGTSCGEKEHATADDMIEIMEEKFPPTSHEAKCVVACFYKH
YKMMKEDGTFDKDAAVKAFDEIKAQDAEIHAKILKVIDACDAKKQMSDDHCVSAASMA
GCVKTEAIAV
>TcasOBP32
LSLSATVFGQSLSEDEMRENARKLMTSCKDKVGASDADVEALKMHQMPESREGFCMLE
CVFDSAKIMQDGKFSKSGMIEGFKPLIGDDKAKLESLEKLSATCESELGDGEDKCETAKRL
VECVIKNGKTHGFEVPPPRE
>TcasOBP33
EENDINEIRSVEENCQKQTGVSVEKVNNFELVDDPLVKENALCILKAYGIMDEDGNIYEDK
LKEQITSELGEKNAEQVAKKCTIKKESPQETAHESLWCVGEQKPIPGASPDEKN
>TcasOBP34
DESVYLSNHEACVKLSGVDETLLETIYEGDVFEDMKFKTYIHCFFKKSGFQDENGVMHF
DAIKSSFHKDFSQTENIDKTITECEEKKLNGESALETAFLHFKCFMGEL
>TcasOBP35
QMKAALKLVRNVCQPKTKATNEQIEAMHTGNWDLDKNGKCYMWCILNMYKLIGKDNS
FDWEAGIATLKAQAPESVRDPAIASVNNCKDAVKTTSDKCEAAYEIAHCMYLDNPEKYFL
P
>TcasOBP36
AMSEAQLKAAVKLVRNMCQPKSKATNEDIEKMHHGDWNIDRTAMCYMHCALNSNKLIT
KENVFNRDYAITLAEKNLPTALKTASIEAANLCKDSAKTLDDKCVAAYEISKCLYESNPEK
YFLP
>TcasOBP37
AILEDSELMKVVENCVKKTNANESEFSSPNFLETTPSQPALCTAKCLLESLEIVNSEGNINM
ETLKEYAQPFESPAREAVATCGEEIKSVTTCDDMEKYRKCVEPLIKNS
>TcasOBP38
EVREECLSENSMTMDELHEGWKMENLPESHLCFLKCLLEKREVIDENGVPQKEKIDEILT
VKQLSDEKREEISTCITNVEKIENCETMSEIMRCFPKKRRD

>TcasOBP39
LDLMADKNFVELRNKCLDKLGLKEEDLRDLKFDGDVSEDLMCFGKCIQEEDGLLDSEGN
LNEEKLEKKIETMPFLSRVSDDTKNNIMECLKEIGKIETCQDFGKQRDCIHKYV
>TcasOBP40
EKESEEAQIFTELDGPAAELRDQCLEKNSMKVTDLKTYNTSNDIPEKELCFYKCFYEGVEF
IDANGNLNVNNMKEIPAISELGDEVLNEITACVEKIGKIRCCGDLRKIEQCYQNITM
>TcasOBP41
QGKYWTTISECLTEHSMGVEDMKKFDLPAEKMSEEMLCFNKCFYDKLLITDENGEINTDN
LMSIPLVNAIDASKHDDLVTCLKKVGKIEECDGVKKIEQCFVEFI
>TcasOBP42
LCMNETGVSEETARNYKPAEDPASEEILCMVKCIFEKIGCLKDDGSFCVDTMKKKNYIMD
VINEENEEKIYECLRGVGKITNCRDMAAVEECFVKNDSK
>TcasOBP43
RSFSHDELDTDLSFIKTCNRTSPISMSKFGLFLTEFNLTEPGTMNECFLHCLFMKYGWMDS
DGGFLLHDIKQTLEESDVEIASLEFILYKCTATESNNRCERAFVFTQCFWDKMAEQQPSED
QFFYNIEDKK
>TcasOBP44
QPEDRHQIALQCIDIVGIDQKVVEDAINIEIPKNNPKYKEFLACSYKKQGYQNENGEILME
NIKKFLQKFYHPSDLQELNSCSGHNGTNHAENAYQALQCIYNRLSNMTVVGN
>TcasOBP45
AIRPWRTCGTWPPCPPNGKMLQNFRIKRASVRLTNTETNETTPEPKAVSSEAQATENCIIQC
IFDNLQMTDSTGYPVHTKILDGLLKNTTNRELRDFLQDTTDECFQVMDKEDTMDPCSYS
NKLVTCLAEKGRSNCADWPVGELPFKP
>TcasOBP46
MLWRRGRFLYGENLDMFDPAGLQACMKKLSVGETELAKALEDKSKDPPEKIMCLFKCAL
EDSGFLQDGVVDKSKWPMPECVQDVVKITNCNDMVALKHCFD
>TcasOBP47
YEFNDPLFMELESSAYPHHRSRRDEDAVTEKCRPFRKKKLCCAEETFDELHDKDRDFKRE
CFKQVVGSKDGPREFDPFRCDKVDKHRRDMTCVSQCVGQKKDVLDKDGNVKEAEFGEF
VKETMAKESWFVSIQDKVVSTCLAEARNATANRDTSDTESCNPAGVKLMHCMFREIQLG
CPTEQIKDQKACARARDKIKRHNEFLPPPPQFLNDE
>TcasOBP48
EDAKEKKCDIPPTAPKKIEDVINQCQDEIKLAILTEALEALNINEHTKSRAKRDTFSDDEKRI
AGCLLQCVYRKMKAVNEKGFPTVEGLVALYSEGVTQKEYIIATLQAVNVCLNKAQKKHLT
KPQSLEAEHGKTCDIAYDVFDCVSERIGEYCGQTP
>TcasOBP49P
MRRGYGRNLDMNLRAKRSNDNDDSQCVSQCVFGYLEVLDDNRVPSETLVIKWLQDHLS
NDMKRIRALREARRCFARLSTSDTEDGCEFSQSLSKCLNLELE

>AcorOBP15
ETLKEHGQKVMEKIIDYATSCADSLGVSPEDIKLLMEKKLPESKEGQCIPSCVNKKFGLQK
ADGTIDKEYRNSDMEEVKAIDEEVYNKMNSIWDKCVLNGAEGSDECDTGIKLVSCMKEE
SEKVGLNKEAMGF
>AcorOBP14

LTIEEKATVTKIGKKCIEETKVDVKLVEKGERGEFADDPKLKEFVFCFLKASDIINADGYPK
PDEIKVRLANDAPVSEIDDVLSQCESKAATPVDRAADLWKCYWKKSPVHIPLQ
>AcorOBP1
MSEEMEELAKQLHNDCVAQTGVDEAHITTVKDQKGFPDDEKFKCYLKCLMTEMAIVGD
DGVVDVEAAVGVLPDEYKAKAEPVIRKCGVKPGANPCDNVYQTHKCYYDTDPQSYMIV
>AcorOBP2P
SLQCLTETNADLTLVHKGQKGEFVDDPKVKAFVFCLLKKSQIVDDDGYPRPDVIKEKLSK
DIPPDVITKVLAKCNPT
>AcorOBP5
SGMDSGVVKNIISLDTFPKPSDKYFKYLECMYFDQGYLDSDGLISYETIEDFILDFYDVDT
VKQALEPCVVLQEGQNGGERAYNAAKCLIQNLEALEKRYEKQNKNADNTT
>AcorOBP4
YLTEAQIKATQKLIRRTCKTKAKITNEEELDRLPKGNWDDVSHTSRCYLHCCLSMLKVINS
DGFIDLEAGMRQSAILPPERRASSEIAIDTCKDKGEGLTDKCDIAYEIAKCLYDFEPKFYLIP
>AcorOBP3
LNEAQMKAGIKLIKNTCRTKTKITDEQIAKMHEGVWDDADDVTKCYCHCALGMMKMQ
AKNGAFEYELFEKQKPMIPETIRETLIASVDNCINAGEGLTKKCDLSYAFFKCVYLYDPEH
YMFP
>AcorOBP6
DAEYEAEKQKLRQKAVGLMTECKDKVGASAEDVQALTNKQLPTTDKGFCLIECIFTNGN
VMKNGKLDVQGTLQVLDTALSKNPDAKKKTTAVLQTCEKEVGAGGANGCETAKLIAEC
FKKEAKK
>AcorOBP7
TTKLLAIFQEHALKTGLDCLSEVDATMDDLKSIINHDMPTTRAKMCLITCIHEKFGIQDAN
GKMLKDQTIAFLDVLKDDPPYHNLAKDHFLHCLETVSETDEKCTIGANLMRCIVVGGNE
KGIF
>AcorOBP8
VTKLQRMIKDAIAHLGTECLSETSDATMSDVQDLVDHVRPTTRKALCLITCIHTKAGMQD
EHGKLKEEGGLNFVEPLKQEDMDYYEISKEHFINCINTVPDDAEACIVGGRFNDCIIIGGK
TKGILD
>AcorOBP9
LTDEQKAKLKVVSDKCIASSGADPSSVEKGRKGEFGDDPKLKEFIFCLLKATEMLDDNAD
VRLDKIKAKISKDLTEAEIDTLLGKCKPTATVPVEKAAEFWKCYWANTPKRIELV
>AcorOBP10
KEAKVDPALIDKADAGEFADTKELKCFAKCFYVKAGFITEQGELLMDVVKAKLPPEHERE
KALAIIELCKDLKGADACETAYAIHKCYFQNAHAANLHKN
>AcorOBP11
HYRGARDTTQKCIDQNSCCSGPPISNFHASDKEASQQCSKEVNFNRGSIRGPLTAEQKDQI
KCIAECIGKKKGYLTADGELIKDKLLSSMKERLQSVAWLAPKLDSMFEDCLPQNENTAKQ
PKKCNDVGLTVGHCIWKQIQLQCPLNEQQNPQNCKNLQEYLTTHNQFPPAPPVKC
>AcorOBP12
ICMKKNLPINGDQIIEN
TSMGQQGYDSSYEDDSKPSSSEDSMSSKEHAMNSLREDSINDSMNRNGDNNLRNNTEIT
DDCVIRCVLKQLGMVDPSGYPDHSKISQNLIKGAENRELKDFLQDSTDDCFQMMEQDEH

MDSCSFSTQLIKCLAEKGKSNCADWPMSDVPFSHLF
>AcorOBP13
ASGESVFLHPRVRRDDEASKCHHRHKFCCGDELMSKLHDKYRDTKRECFKQVTGKEFGG
GPPFTCEELEERKKEMTCVAECAGKKKGAVDDKGNIKEDEVKKLVAECTAELEWFKPML
DEVTTKCIAEAKAAAEKYDKKGCNPSDIKFSFCIFKEIQLNCPADQIKDQERCDAMRASLK
KHDHPPVH

>DmelObp8a
vpmrsspqslallrardqcgreltaaqrlqldrmqfedaahvrhylhcfwsrlqlwldetgfqaqrivqsfggerrlnveqalpaingcnak
tssrgsgaqtvvdwcfrafvcvlatpvgewykrhmsdvingna
>DmelObp19a
Gvteeqmwsagklmrdvclpkypkvsvevadnirngdipnskdtncyincilemmqaikkgkfqlestlkqmdimlpdsykdeyr
kginlckdstvglknapncdpahallsclknnikvfvfp
>DmelObp19b
Deeegsmtvdevveliepfgdactpkpsrenivemvlnkedakhetkcfrhcmleqfelmpedqlqynedktvdminmmfpdred
dgrrivktcneelkaeqdkceaahgiamcmlremrssgfkipeike
>DmelObp19c
Qtqafdlakllpktgtepiwavidrnlpqvqelvtaarmeciqklqlprdqrplgkvtnpsekekclvecvlkkiklmdadnklnvgqve
kltslvtqdnkmaiavsssmaqacsrgissknpcevahlfnqcisrqlernnvklvw
>DmelObp19d
Kpheeinrdhllelaneckaetgatdedveqlmshdlperheakclracvmkklqimdesgklnkehaielvkvmskhdaekedapa
evvakceaietpedhcdaafayeeciyeqmrehgleleeh
>DmelObp28a
Fdekealaklmesaescmpevgatdadlqemvkkqpastyagkclracvmknigildangkldteaghekakqytgndpaklkiald
igetcaaitvpddhceaaeaygtcfrgeakkhgll
>DmelObp44a
Sdyklrtaedlqsarkecaasskvtealiakyktfdypddditrnyiqcifvkfdlfdeakgfkvenlvaqlgqgkedkaalkadiekcad
kneqkspanewafrgfkcflgknlplvqaavqkn
>DmelObp46a
Rstppaldedcelnsvdtmhdfccdlhdespqfsdcqmewhekipyetdeeeqtymfctaecsfnstnflgrdrrslnlnevkehlesdl
vndadikllydtyvkcdkhalslmphkgvkqlskrlsrlgchpypglvlecvanemilhcptkrfrqtaqceetrnhlkqcmqylkyks
>DmelObp47b
Qatidcqrppqlvdpalcckdggrdqvaeqcaqrilgtangqkaggppsldtaaclaeciltsskyidepqklnlanirsdlsakfsndtly
vetmtmafskcepqsqrrlamimqqqqqvqqqktqqqqprcspfsaivlgctymeyfkncpdhrwtpnaqctlakayvtqcglga
>DmelObp49a
Dvdcskrpsfvnpktccpmpdfvtaelkqkcikfdmtpppppdgeasgsfeskrrhhhphpppcffscifnetgiyqnrkldeaklna
ylqevfedssdlqttatqafttcatkvadfeanlpprpapspppgfpmcphdaghlmgcvfrnmmkncpdsirndsqqctdmkefftk
ckpprgpppsaedm
>DmelObp50a
Akcraapksvqnvhvccsaplpnwgvfnrechksaiqasvsinriskskvnlanflikcrldcdfnassvlqgnrliqakvrpmlerafs
neptidayesnfakcstvvrskyqelsplsrqsdacdrhalfyslcayarliftcpdkmwqrnnrmcqeakayakkcpwpalkmfmrn
t
>DmelObp50b
Vsndmgglqkctellnthklvyccgksfldkfpfvgsnctpfwddygpcryeclyrhwdlldqdnkikkpelylmitslysplngydk

ygaafkaahetcealgsrhadflllysnqvadkmgmasstclpyamlhaqctmvyltancprenwiddpkcnslqkllssctkkldekt
nalkgkdeeltdngcghidsegsnllmacfltlmiakfisdh
>DmelObp50c
Dpidvdctrrqdfnivkdccvyptfrfdqfksqcgkympvgaprispclyecifnktntvvdgaihpdnarlmleklfgnqdfeeayfn
glmgcsdsvqemisnrrsrpqrkteqcspfslfygicaqryvfnhcpssswsgtescemarlqnmncskpsrgsshrl
>DmelObp50d
Adpicsqrpdvtalrnccklpnldfssfnskcsqylvngvhispcsfecifraanalngthlvmeniekmmktilgsdefvhvyldgfrsc
gnqekvlikamkrrrvpitgkcgsmaimyglcahryvyrncpesvwsksatcneareysircddm
>DmelObp50e
Sfncsappnfnnfdintccrtpeldmgdvpqkchkyvsglksanskypsyahlcypdciyretgamvngkikvnrvkqyleehvhrr
dqeivshivqsfesclsnvkghmkslniesykvlphgcspfagiiyscvnaetflncpqqmwknekpcnlakqfaeqcnplphvplps
s
>DmelObp51a
Lfeseanecakklgitpdyfenfphssrvkcfyhcqmekleiiangvvtpfdlkvlnispesydkygvkvkpclklshrdkcelgylvfq
clkrefnl
>DmelObp56a
Sslnlsdeqkdlakqhreqcaeevklteeekakvnakdfnnptenikcfancffekvgtlkdgelqesvvleklgaligeektkaalekcrt
ikgenkcdtasklydcfesfkpapeaka
>DmelObp56e
Vgltdsqkaeakqrakacvkqegitkeqaialrsgnfadsdpkvkcfancfleqtglvangqikpdvvlaklgpiageanvkevqakcd
stkgadkcdtsyllykcyyenhaqf
>DmelObp56f
Mkssekikaclkrqlgytitentkfdakedslqskcfyhcllevkgviandaisseqprkvlekkygitdtdelekaeekchsikasgkcel
gyeilkcyqsitkh
>DmelObp56g
Qqanidssvskelvtdclkengvtpqdladlqsgkvkaedakdnvkcssqcilvksgfmdstgklltdkiksyyansnfkdviekdldr
csavkganacdtafkilscfqaan
>DmelObp56h
Npdfrqimqqcmetnqvteadlkefmasgmqssakenlkcytkclmekqghltngqfnaqamldtlknvpqikdkmdeissgvna
ckdikgtndcdtafkvtmclkehkaipghh
>DmelObp56i
Gpikdqcmaaagitaqdvanrhetddpghsvkcffrcflenigiiadnqiipgafdrvlghivtaeavermeatcnmiksetshdescef
awqisecyegvrlsdvkkgqrtrnhrg
>DmelObp57a
Iqslslleetnyvsdclasnnisqaefqelidrnsseeddlentdrrykcfihclaekgnlldtngyldvdkidqiepvsdelreilydckkiyd
eeedhceyafkmvtcltesfeqsdevteagkntnklne
>DmelObp57d
Ntsvfnpcvsqnelseyeahqvmenwpvppidraykcfltcvlldlglidergnvqidkymksgvvdwqwvaielvtcriefsderdl
celsygifncfkdvklaaekyvsisnak
>DmelObp57e
Ndpcphnqgidediaesilgdwpanvdltsvkrshkcyvtcilqyynivtasgeifldkyydtgvidelavapkinrcryefrmetdycs
rifaifnclrqeiltks
>DmelObp58a
Lkcrsqeglseaelkrtvrncmhrqdededrgrggqgrqgngyeygygmdhdqeeqdrnpgnrggygnrrqrglrqsdgrnhtsnd

ggqcvaqcffeemnmvdgngmpdrrkvsylltkdlrdrelrnfftdtvqqcfrylesngrgrhhkcsaarelvkcmseyakaqcedwe
ehgnmlfn
>DmelObp58b
Vrvhcrhmeriheenihhcckhqdghddvtescakqtnfrlpspneeaivdvtvdqamvgtcwakcvfdhynlmenntldmdkvr
syykryhqtdpeyatemlnayekchtqseeatekflslpivrafstakfckptssiimscviynffhncpasrwsnttecvetlafarkckd
vltt
>DmelObp58c
Ikidcenteainedhihycckhpdghndliegcaretnftlpnqneealvditadrairgtcfgkcvfsklnlmkdnnldmdavrslfterf
pddpeyakeminafdhchgkseentsmflskplfkqmskqfcdpkssvvlacvirqffhncpadrwsktkecedtlafskkcqdslatl
>DmelObp58d
Qdneettavaissgdltedkcntsragccselyigeeedlvkcfvihspklpvdgdadigktlrflscfveclykqkkyigksdtinmkmv
kldaektfvdrpkekdyhiamfefcrkdavgvynllkaspgakvllkgacrpyllmvfmcisdyhqkhecpyfrwegtakagtkdmc
enakaecyqidgitlptkspa
>DmelObp69a
Veinptiikqvrklrmrclnqtgasvdvidksvknrilptdpeikcflycmfdmfglidsqnimhleallevlpeeiyktinglvsscgtqk
gkdgcdtayetvkcyiavngkfiweeiivllg
>DmelObp76a
Mtmeqfltsldmirsgcapkfklktedldrlrvgdfnfppsqdlmcytkcvslmagtvnkkgefnapkalaqlphlvppemmemsrk
sveacrdthkqfkescervyqtakcfsenadgqfmwp
>DmelObp83a
Qrdenypppgilkmakpfhdacvektgvteaaikefsdgeihedeklkcymncffheievvddngdvhleklfatvplsmrdklme
mskgcvhpegdtlchkawwfhqcwkkadpkhyflp
>DmelObp83b
Qeprrdgewpppailklgkhfhdicapktgvtdeaikefsdgqihedealkcymnclfhefevvddngdvhmekvlnaipgeklrni
mmeaskgcihpegdtlchkawwfhqcwkkadpvhyflv
>DmelObp84a
Lqdhakdngdifiinydsfdgdvddistttsapreadyvdfdevnrncnasfitsmtnvlqfnntgdlpddkdkvtsmcyfhcffeksgl
mtdyklntdlvrkyvwpatgdsveaceaegkdetnacmrgyaivkcvftraltdarnkptv
>DmelObp85a
Mspgsvvfsmfltrpsldkgnsecrkslnlpahrkfnfaelytinmcieecnfigcgyieidppfrldlanirtnlqtiapqpqnesipflvd
ayrkcelfrsshgrrftlhlpdiefieepcnpfalqiticvrihamqkcpsefyvdsdecrlareyftqcvgdietnla
>DmelObp93a
Cdvqkndkainscrksllgnnstnsngevrnlksdkvalhaciaecsfrtngfllsngtvntqalqksyqqryndpnmsqlmlkslnsc
tdyarkrvqefqwmpkkgdcdfypatllacvmekvyincptskwkntsdctamwkylvacddvasnkkk
>DmelObp99a
Dyvvknrhdmlayrdecvkelavpvdlvekyqkweypndaktqcyikcvftkwglfdvqsgfnvenihqqlvgnhadhneafhas
laacvdkneqgsnacewayrgatcllkenlaqiqkslapka
>DmelObp99b
Ddwtpktgeeirkirvdclkenplsndqisqlknlifpnepdvrqyltcsaiklgifcdqqgyhadrlakqfkmdlseeealqiaqscvdd
naqknptdvwafrghqcmmaskigdkvrafvkakaeeakkkaa
>DmelObp99c
Asvwklptaqmvyedlekcrqesqeedaatlrclvkklglwtdesgynarriakifaghnqmeelmlvvehcnrmeqdtshlddwaf
layrcatsgqfghwvkdfmsqkever
>DmelObp99d

Dhhhhhhdyvvkthedltnyrtqcvekvhaseelvekykkwqypddavthcylecifqkfgfydtehgfdvhkihiqlagpgvevhe
sdevhqkiahcaethskegdscskayhagmcfmnsnlqlvqhsvkv
>DmelObp73a
VEYLIRFETKKAKCLNPPRTARKVESVIRECQDEVRNKLVNEAYEILKEQVSQNQPPIDPN
DDSIDFIWPSVPEAPSLDHSPNISQYEYIVYDEPEPQRHVARLMRNIRRLDVASSGIYHPTLV
PLEDKRIAGCLLHCVYAKNNAIDQRGWPTLDGLVHFYSEGVHEHGFFMATLRSVNLCLRT
MTARYGVNRKELPKKGESCDLAFDVCSHMNTNIFKDLQFWAPFISYVNESRAINYI

>DponOBP25
QDFTEEQRKKIIENRQQCIEETKVNPDLIEKADLGDFAEDQALKCFTKCFYQKAGFVNDK
GEVQKDVVEAKLPPQADKKRALEIVDKCALKGKDACETVYLIHKCYFEHTHPEADEKTA
KDGKSEEKKA
>DponOBP1
KNNKCDIPLSAPKRIEEVINTCQDEIKIAILSEALEAFKVNEHKVVSRAKRSAFNEDEKKIA
GCLLQCVYRKLNAVNEYGFPTVEGLVSLYTEGVTQKEYVVATRQAVTKCLENAQKTHEIS
TKTVEASKSCEVAYEVFDCVSLEVAKYCGQTP
>DponOBP17
LSDEMKELAQMLHNTCVAETGVNEDFIRKVNAEKIFADDENLKCYIKCLMAQMACIDDD
GIIDEEATIAILPEEYQALAAPVIRACGTKHGANPCENAWLSHRCYAEMEPSVSG
>DponOBP32
ELDQTSLPPETKELMAALHKNCIEQIGVSEADVDQLRAANFEEDAKLKCYTRCLMAESG
VMDENGAIDVEAFAEILPEDIRGNIQTIFRRCSLTNKDIEDQCVKAYEMVKCWHKEDPESY
FMI
>DponOBP7
DLTEEQKQKIVANGKACVADTGADPELIKAARQGKFADDAKLKAFALCMSKKIGFQNEA
GEIQSDVVQQKLGSAIGDNEAAKKLVEKCLVSKGSGEETAIQSFKCYYENTPTHIAVF
>DponOBP14
ELDQTSLPPETKELMAALHKNCIEQIGVSEADVDQLRAANFEEDAKLKCYTRCLMAESG
VMDENGAIDVEAFAEILPEAVRGNIQTIFRRCSLTNKDIEDQCVKAYEMVKCWHKEDPES
YFMI
>DponOBP12
AMTEAQMKAALKLIRNVCQPKNKATDAQIAAMHNGDWNQDKNGMCYMNCVLNYYKL
QLPDNSFDWETGLKVVESQAPPSMAGFIMETITGCKDAVKTRDDKCKAAVEITKCLYDQN
PEKYFLP
>DponOBP5
MSDEMQELANQLHTTCIGETGAAEDAITNARNGDFSEADSFKCYIKCLLSQMAIIDDNDG
TIDVDAMVAVLPEEIQEATEPIIRKCGSIIGANPCDSAWLTHKCYYKEGPEHYFLI
>DponOBP15
HPPRGPPGPPPFLGHPDPESANECRTEVGLTSEDRETKKNGELTEKELCFIRCLGQKNGALS
DAGALNIETIKNDLPDHLEDSEAVIACLKKVGTVTTCQHIKKVAKCYPEPKEPMDRT
>DponOBP23
LKITLPPELQEYVDDLHKLCLEKGGLTENDHQTYNINDKNEKMMCYMKCLMLESKWMK
SGGEIDYDFIETQAYPEVRDLLLSALNKCRTIEEGADLCEKSYNFNKCMYEADPVNWFFV

>DponOBP18
LSDEMKELAQMLHNTCVAETGVNEDFIRKVNAEKIFADDENLKCYIKCLMAQMACIDDN
GIIDEEATIAILPEEYQALAAPVIRACGTKHGANPCENAWLSHRCYAEMEPSAYMLI
>DponOBP10
RLTEKQVAAAVKLVRNMCMGKSKVNPEDIDKMHQGNWDVDYEAQCYMWCGFNMYK
MLDKENHFDKKSALQQMEQLPTDLQDYVIKCMGQCENAVTNFDDKCVVAFEYSKCLYF
CDPEKYFLP
>DponOBP2
YDFQDATFNEILSSDFEDIFDTLDNTYLHPRAKRNEEAVNSDEKCRRRHHRKPKLCCGED
VLDSLQEKEKEIVRLCFKDITGGVKESKPDRGFGNHRNFDLFSCEAVEKRKSDMICVEQC
KLQKQGLVSDDGSPKPEQISTYLKEAFTTQTWFEKVSQGIVEKCVNEAINATKNPVKFYTE
GNKLCSRSGIVLKHCLFNSIQLSCPAGQIKDKNACERFQERAKKGKDLFDQPPGPPPFDDN
REEQI
>DponOBP21
LECGLSKISSEHFRKIASECVKDNETLNRIWELTSEASMDDESASSDEEVPITQGKEAPNLD
LGSSAQKSMKMSRASRTKRSRKSFNNESPMSQRKPSPTSTTTEQTTTIQSEENEDNADAN
NVEESGEVCILQCIFEKLEMTDTNGLPDHKKFAAALVESATGRETRDFLQDSVDECFQETE
EGDFENSCEYSTKLVTCLAGRGKSNCADWPVGDLPF
>DponOBP31
GNSDDLFARIAPADVEMCGKDTGVDRKEFEDAREKRALNHSMLCFLKCAMEKVGFLKD
GHLEIDQAKGSLPDKMMEPVVECFKAVGPISTCDDIQKVEDCLPSS
>DponOBP4
MGKQAGFINEAGDVLKDVLKEKSLKLFNDPALVQKLIDQCIVKKETPQETSYHAHVCLYK
NSPGHLALTQFGAISQEKKEKKVQIIKECAEESGVSRSAVLSARKGDFQDEPLLKQYFFCIN
KKSQIQNEAGEYKTDVIRKGLTELFNAEEANRIIEKCARIQDSALNTAFQSFKCFYNEAPEI
TGVF
>DponOBP20
LECGLSKISSEHFRKIASECVKDNETLNRIWELTSEASMDDESASSDEEVPITQGKEAPNLD
LGSSPHKSMKMSRASRTKRSRKIFNNESPMSQRKPSPASTTTEQTTTVQSEENEDIADANN
VEESGEVCLLQCIFEKLEMTDTNGLPDHKKFAAALVESATGRETRDFLKDSVDECFQETEE
GDFEDSCEYSTKLVTCLAGRGKSNCADWPVGDLPF
>DponOBP13
DRQQVVDFHRPCLDHHEIEDDDLHFALDKIKMRDDDEFYLHFFCVAKQGQLMTEDGTVN
TDNFETNMKGIIDEDNMENVAAIVRLCLIQKDTVLQTIRNAVDCFMGKDHKL
>DponOBP3
DAEINQSTFEAGRNRIMEMSRTCDENPATAVDQKALENYLESNGPAPANAGVHALCITKN
LGWQNEDGSVNKPLITEKVKAIFGSVDAKIERYIEDCTEAKAKPEDTAEQLLNCYRKHSP
KTE
>DponOBP8
LDQAWRDHMKEKLTEFGLECAESEQATSEDIEALHNHKPPVTHAGRCVIFCVSKKLNLM
NADGTLNVTPQSDWIEKVKETDSEAFEKMKTVYHHCADTVEVEADACDTSLSYAHCIKE
EGHKVGLYTVSAD
>DponOBP30
LTDKQKELLTQHYNQCVAISKVDQAVLQKARAGDFANDPNLKTHIKCISEKIGFQGTDGK

FRRDVIEKKLKETIPGDNAKNAKLIETCVVANKDPKLQAFNAFKCLYTNAKINLL
>DponOBP6
KITLPPELQEYVDDLHKLCLEKGGLTENDHQTYNINDKNEKMMCYMKCLMLESKWMKS
GGEIDYDFIETQAYPEVRDLLLSALNKCRTIEEGADLCEKSYNFNKCMYEADPVNWFFV
>DponOBP28
AALTKEEIKERLKAAHDKCQADPQTAIDEAALKAFKDSKGKGQLPANMGPHDLCISKAL
KWQNADGKVNKELIKERITDNVADASKVDAIVNECAVDKENEIATAENLFKCLLKHHATA
VHGH
>DponOBP26
NPVRTHKLLSKSELHEIATSCLEEVQLSGSIVYNILKTEIFPRDNNKYRDFLACSYKKQGFL
SEDGTKLLYDNLFHFISHFYGPTEVQALKHCNLIRREDPGFLCFDTMKCIIDALKQLEFDA
NADIGIETNQVV
>DponOBP22
DQREKAVEFQRGCMEAHGLLEDELHEIMDGKPIQNEAFYFHFFCVVKKAKLISDNGIVNT
DHFEENLKDVIDEEHMAHVAALTRKCLIQRDDIFTTIKMAIDCFYSSEHKL
>DponOBP11
HDPHGLDSVHKECHNEVASQHYLCMAKGLHLVTPEGKVNVNGVKTHAGHVVSESAKID
QIAKECAVDHASTEETVNHLFKCLEEKHVLSLAGHVAPQHHH
>DponOBP29
DLTEEQKQKIVANGKACVADTGADPELIKAARQGKFADDAKLKAFALCMSKKIGFQNEA
GEIQSDVVQQKLGSAIGDNEAAKKLVEKCLVSKGSGEETAIQSFKCYYENTPTHIAVF
>DponOBP19
DFDFSNYKEFEHLAGDQREKAISIFKECMAETGATHEMMEKSVEGDIPDDIVFKNHLVCIG
KKSGFIDENGLHSKEKLKEKLTLLLGDEGLVDKILDKCFMEKGTPQDTAFELAKCCHKEY
HN
>DponOBP9
KKNKSNDEEKPKSYKKVFKECQKKDETRVDASIIRKLKKHKQVDLPANFGDHKLCVFKGI
GLLKADNTVDEDKLKKKISSAKPQKDNVDSIFTECKSSKSTLQETALNLDRCLTTNSIEF
>DponOBP16
YVPNVNNKIRDFCIDDSGVSIEMVENLLANPEKQLIDVESCYLHCIFTEMGLLSENGNVEV
EKFKSLKASEAPYIDLTCLEEIKSIDHCSEMMILRACHV
>DponOBP27
FLTVPKCLISTGARIKDLHNLATGDSLPESSRCFVKCVGEESGLILDGTLHSEHFEALPMVS
RLKADVFVDARRCIESVQGIKIESCKDIDNLNDCMKIVYRQKYSDSK

>ItypOBP10
YDFSDSIFNDHLNQIYYTLDNWQHERIRRNAEDVELKCRKPPPPMPKPCCAQDSFRDLMD
KEREVLRDCFKEVVGEEHHPGRSNHPNKFDMFSCEAVEKRKNDIICIKQCLGSKLGLVNK
DGKLDQAQIGNYVKSTFKNEAWLSPLADQIIGKCLVEAESVAPPKFHIEKLKPCKPSVITFK
HCLDREIQLNCPADQIHNQESCERFRNHLNHKNDFDEDQPMMGPPDDD
>ItypOBP11
STGDSTMKFILLVIVVGQMGCVFGAMTESQMKAAFKLIRNVCQPKNKATDAQIEAMHKG
DWNQNKNGMCYMNCVLNYYKLQLPDNSFDW
>ItypOBP12

LQKTNNKCEIPTAAPKKIEDVINTCQDEIKIAILSEALEALNINEHKVSRKRRSTFNDDEKKI
AGCLLQCVYRKMNAVNQYGFPTVDGLVSLYTEGITQKEYVLATLQSVTKCLGKAQKTYD
IPAQNGTASTACDVAYGVFDCVSEEVAKYCGQTP
>ItypOBP13
KVEMLTDKNEIIKCAMECMQAEIDRLTEERNEHLKKLFESHNAQISETKKKQWCYNCEQ
DAIYHCCWNTAYCSQTCQQQHWQAEHKKVCRRKRQT
>ItypOBP14
QDFTEEQRKKIIQNRQDCIQETKVNPELIEKADQGEFIDDQALKCFTKCFYLKAGFVNDEG
EVQKDVVEAKLPPQADKKKALEIVDKCAVKGKDACETVYLIHKCYFEHTHPDLPAKAEE
EKKA
>ItypOBP15
MIQHHRTNSDLTAVKMKILAVLFVICVLFQFTIARNGGNLHYSKISMKKVQKRCQKNEESR
IDPDVLKKLRKGEEVVQLPDNFPDHVTCLMKGMEYLNDDNTVNEEKVRNMVQRRVTDD
QDVDAIVGECKAVKTALKETALNLINCLRKHELLWNHNFHD
>ItypOBP2
YNFQDEDFXSAVVVRDGRIVDSIDSGPVHPRVRRDQEAATVAEEKCPKRHRRPKLCCAEE
TLDALHAKKKEITKACFKEVTGLEKQDRHDHGPHFKRFDLFNCKEVEKRKSDMICIDQC
VGQKKGLLDDSGAPIRDQLIQHLKQHFSNESWFDQTVVEKITSNCLAAAKNATETPIKFST
EGLKACNPSGITLKHCLFREIQLSCPADQIKDKTACDRFQDRIQKEIEIDDLRLAPDDQQ
>ItypOBP3
RMTEKQLAAAVKLVRNMCLSKEKAKLEEVDKMHEGNWDIDHKTQCYMWCVLSQYKLI
GKPNHFDRESANIQVDTLLPESMHDYVVGCLDKCENAATNFDDKCVAAYEYAKCLYFCN
PKEYFLP
>ItypOBP4
DLTQQQKDKLLADGKACVAETGVSTDLIQAARQGKFTEDDKLKAFSFCMSKRLGFQNDA
GDIQTEVVKQKLGGALGDLGVAAQLVTKCLVPKATPQETAFESFRCYYQNTPTHLTVF
>ItypOBP5
MRQPGGNNKNTQQDYEMWTPSTGYQPSGSNNDFNVRTRYDGNTRFNRPSSEECRDQGN
GNIPRSPFGSSNLPRRQRSSYFNREDDDNDNDCISQCVLGYMQLLDTDRSPSETLIIKWLQ
EHVTRNEMDRIKALRDTRKCFGKLVTTDIEDGCEYAKELSKCLELDLE
>ItypOBP6
EEVTTSTKRQLTREKKKKIGKTCMLETGVRIETILRAIKEDIPKNDEKYKSYLVCSYKKQG
YLSEDGGTMLYDNLYSFLQESAGYAKEDLHYIDDCKTITAETPGDLCLKKLVGILDGLHK
VEKNREIDTNTIES
>ItypOBP7
QTDKQKELLAQHYKECLAKSKVNEATLQKARIGQFADDDKLKEHILCVAQKIGFQNSAG
QFQNQVIETKLREALKGDAAKTKKLISDCAITNPDPKLQAFNAFKCVYQKASINLL
>ItypOBP8
EVSKEELEKLKEIHDTCLTESGVDQSMPEKAFKGEFTDDPKFKEHLLCFHKK
>ItypOBP9
EKKCNSSNCMYDRMLETVGKEFIEQCFKETGVTPEDIRSVMEQNGYGEKQIVFPKMLDK
ENWYFGKRWSNQYRLY

CSP Sequences

>CbowCSP1
EKYTTKYDNVDIDSILNSERLIKNYMDCLMERGPCTPEGKELRDNLPDALKTECHKCSDK
QKEVSKKVLRHLVKNKRKEFDELTGKYDPEGVYKNKYKEDLAKEGIIV
>CbowCSP2
DEKKSRANVPKYTTKYDNVDLDAIINNDRIFRNYIECCLGKRKCTPDGLELRNHIRDAMD
NECDKCSETQKKAMKKVGRKLYKEKPEWWKELCDHFDPDMKYRTKYQKFIDEALAEE
DK
>CbowCSP3
RPEDKYTTKYDKVDLDAILQNERLLRSYIDCLLDKKKCSKDGEELKKILPEALKSKCAKC
NENQKKGAKKVIRYLLKEKRAWWDELEAVYDPEGIYRKTYEKELKEEGIQI
>CbowCSP4
GEKYTAKFDNIDYEEILRSERLLKNYIFCLLDKGPCSPDGLGIKNILADALETECSKCSDRQ
KEGSTKVIRFLIENHAGWWKELTEKYDPDGIFMQKYRDQWNSNN
>CbowCSP5
KPAEKYTTKYDNVDLDTILKSDRLLKNYVNCLLDKGNCTPDGAELKKVLPDALQTDCSK
CSDVQKRGSKKIIRYLIDNKAEWYKELEAKYDKNGVYKKKYDKELEDAKV
>CbowCSP6
QTYNTKYDHVDIDSILANKRVLSSYIKCIMDEGPCTSEGREFRKHIPEAITNNCAKCSDAQ
KRIIRKTSRFIRRERPQDWDRVSRKYDPQQKYTANFNKFLSEN
>CbowCSP7
QNSYSRKYDNVDVDKILKNERVLSNYIKCLLEEGPCTAEGRELKKTLPDALANECEKCNP
NQKNTAEKVMKHLMSKRARDWERLSKKYDPQGNYKKRYQHLVEKVAN
>CbowCSP8
QILSRNNYIQKQLLCTLDRAPCDHLGSQIRDALPEIIGNNCKSCDQRQTANAKRIAVFVQS
KYPDVWNALVKKYSRLE
>CbowCSP9
MEVSYHLIVCIVVFSYVSEDRFVSTTSLNRVERAVEKYSNKYDKFDVAGVLASARLVKRY
GDCLMDRGPCPPEGRFLKDIVPDAIATECSKCNNIQKKQAGLILQHLLLHYRPLFLELCDK
YDPTGKARKQYGIDTNEADEYEDYDEA
>CbowCSP10
YNTAFDRVNVEDVLKNKRLLKRYVDCLLGVPKTCTKDGQLLKDTLPNALKTKCEDCSEP
QRKGAKRVANYLIDCKPKWWSDLAKIYDSDGIYTKQYHDELLAEGINIDGSSKDTEHKTQ
CYN
>CbowCSP11
MQIKYQDAILVTVVLCAIVTIVNALPQSQAISDEALESTLKDKRYLLRQLKCALGEAPCDP
VGRRLKSLAPLVIQGSCSQCTPQEQRQVRKVLSYMQVNFPKEWNKVLKQYSG
>CbowCSP12
MKEGKARKLSQCYSFHGHEQCKYISLKMFLPFVVLSCLITLSISAVPEKSRYTTKYDNINLE
EIIHNDRLLKNYVDCLLDKGRCTPDGLELKKNMPDAIETDCSKCSDKQKEGSEIMMRYLI
DNKPEYWNPLQEKYDPSGSYKKRYLDAKKTEVSVEPIVKS

>DponCSP10

EEYTSKFDNVDLDQILSSDRLLRNYINCLLDKGKCTPDGIELKKNLPDALENECSKCTPKQ
RDGAKKVIRYLIENKRDYWDEVAAKYDPEGTYYKKYQEQAKKENIKL
>DponCSP9
ETTERPAISDEALEKTLSDKRYLQRQLKCAVGEAPCDPVGRRLKSLAPLVLRGSCPQCTEQ
EKKQIKKVLAYVQVNFPKEWNKMLQTYAG
>DponCSP4
QSSPYTSKYDNVDVDKILKNDRVLTNYIKCLMEEGPCTPEGRELRKTLPDALASGCSKCN
EKQKSTTEKVIRHLQTRRAKDWDRLSKKYDPEGVYKQKYTAELKTETTA
>DponCSP7
RRVKRSAQTYTTKYDNIDIDQILASNRLLKNYVNCLLDKGGCTQEGKELKKYLPDAIATE
CSKCSQTQKKIAGRVFQALLLNHRDDWELLTNKYDPEGNFQKKYLQEDEDYSDLEEA
>DponCSP5
EVTEKSQYTTKYDNVDINEVVHNERLLKNYVNCLLDRGPCSPDGLELKKNMPDAIETDC
SKCSDKQREGSEAMMRFLIDNKPEYWNPLQEKYDPTGSYKKRYLDAKKAEVAIQPAEKT
P
>DponCSP3
EVTEKSQYTTKYDNVDINEVVHNERLLKNYVNCLLDRGPCSPDGLELKKNMPDAIETDC
SKCSDKQREGLEAMMRFLIDNKPEYWNPLQEKYDPTGSYKKRYLDAKRAEVAIQPAEKT
P
>DponCSP1
EEYTSKFDNVDLDQILSSDRLLRNYINCLLEKGKCTPDGTELKKNLPDALENECSKCTPKQ
RDGAKKVIRYLIENKRDYWDEVAAKYDPEGTYYKKYQEQAKKENIKL
>DponCSP6
KPQEKYTTKYDNIDLDAIIRNDRLLRNYIDCVLGKKKCTKDGEELKVHLPDALQSDCSKC
SEAQRNGSRKIITHLLKNKRGWFNELQAKYDPAGNYLSKYSEELRKEGIVI
>DponCSP11
QILNGNVYVEKQLLCALDRAPCDNLGRQIKDALPEIIGKNCKACDNKQLSNAKRIARFVQ
NKYPNVWNDLVRKYGNPTN
>DponCSP8
DTPKYTTKYDNVDLEEIIKSDRLMKNYVNCLLEKGKCTPDGAELKRVLPDALHTECSKCS
DSQKKGSRKIMRHLIDNKPEWWTELENKYDKEGAYKKQYREELKKDGIKL
>DponCSP2
QTYTSRFDNINIDEILSNKRVLNNYVRCVLDEGPCTAEGRELRTHIPEALRTSCAKCTPSQQ
KFVRKGANFLIKNDPDQWKRIAKKFDPEGKFAPQFRQFLNA

>ItypCSP1
DKYTSKYDDVDIDQILQSERLLRNYLNCLLDKGRCTPDGAELKKNLPDALENECSKCNES
QXKGASKVIRYLIDNKRQYWDELAAKYDPEGVFFKKYEAEAKKDLLDQIGRA
>ItypCSP4
KPAVKHYASKYDHIDVETILNNPRMVKYYSACLLSQGPCPPEGVEFKRILPEALHTNCHRC
TEKQATVTLRAIKRLKKEYPKIWSQLSQMWDPDDVYVRKFESTFGNRNKIPSVVVNNGW
DLGSSTTSNADEPRPDTTTHQIITSPNIMSFTTSKTSSTPITTSSTANPSTKTSTTTVGTTTKPP
SRPAPIPGLLP
>ItypCSP5

QSPYTSKYDNVDVDKILKNERVLTNYIKCLMEEGPCTPEGRELRKTLPDALASGCSKCNE
KQKDTTEKVIRHLMDKRTKDWDRLSKKYDPQGVYKQRFEKELSARKLA

>AcorCSP1
QDNSQGLFLWKYKVDVNTVISSKRLLINYINCLLDKGPCTTEANELKKILPNAISTQCKDC
SITEKQAVGKIFAHLLQYHRDLWNELLDKYDPDGTFRKQYELDEDEDYDDEKESN
>AcorCSP2
MPQNRPQVSEEAIDRALKDTRYLMRQLKCAVGEAPCDQVGRRLKSLAPLVLRGACPQCS
PGEVKQIQKVLGYVQKNYPREWNKILQQYAG
>AcorCSP3
GDEKYTTKYDNMDIEHILSNDRLLSKYVQCLLDLAPCTVDGLELKKNMPDALETNCSKC
SDTQKVSSEKIISYLIDNRPDYWTPLQKKYDPTEEYTKKFIEARKVKAKVST
>AcorCSP4
ADPPGGYYTTRYDHLDIENILNQKRLVHYYAACLLEKGPCTPQGTEFKNILPEAIKTNCLR
CTEKQRIVTTRTIKRLTKEYPDIWGQLEQKWDPTGANVKRLLASVNRPRPISGIPSLADRF
GNEDQNNLGEITTRSTTSSSTIGGGISSTTSSSVSVPSSTGSSSSSSATTTTTTTTTTTTTTRP
PTTTFRTIYKPVTARPFNSIGPNLMILNPKVIIDKVLYTADAVLNTVSGVLKG
>AcorCSP5
QNRYTTRYDSIDVDSILSNRRILTNYLKCLMDEGPCTNEGRELKKTLPDALANGCSKCNE
KQKSSAEKVIRHLIKNRSNDWKRLTAKYDPSGQYRKKYEAQYNIKA

>AgamCSP1
QDKYTSKYDNINVDEILKSDRLFGNYYKCLLDQGRCTPDGNELKRILPDALQTNCEKCSE
KQRDGAIKVINYLIQNRKDQWDVLQKKFDPENKYLEKYRGQAQKEGIKLD
>AgamCSP2
QEQYTTKYDGIDLDEILKSDRLFNNYFKCLMDEGRCTPDGNELKKILPEALQTNCEKCSE
KQRSGAIKVINYVIENRKEQWDALQKKYDPENLYVEKYREEAKKEGIKLE
>AgamCSP3
QDKYTTKYDGVDLDEILKSDRLFNNYYKCLMDTGRCTPDGNELKRILPDALKTDCAKCS
EKQKSGTEKVINYLIDNRKDQWENLQKKYDPENIYVNKYREDAKKKGINL
>AgamCSP4
ETANETYVTKYDNIDLEEIFSSKRLMDNYMNCLKNVGPCTPDGRELKDNLPDALMSDCV
KCSEKQRIGSDKVIKFIVANRPDDFAILEQLYDPTGEYRRKYMQSDALAEHVKQEDRDLSS
SGDGDADTETEAHATEHNSQDHDHREGQSDAE
>AgamCSP5
QEVARTLYSTRYDNLDIDTILASNRLVTNYVDCLLSRKPCPPEGKDLKRILPEALRTKCARC
SPIQKENALKIITRLYYDYPDQYRALRERWDPSGEYHRRFEEYLRGLQFNQIGGSNGGSGV
GNTVLSNL
>AgamCSP6
QKYTDKFDNIDVDRVLSNDRILNNYLKCLLDKGPCTQEGRELKKTLPDALKTNCEKCSEK
QRTSSRKVIAHLEERKPQEWKKLLDKYDPEGIYKSKFEKINKRS
>AgamCSP7
NDSQNINRLLNNQVIVSRQIMCVLEKSPCDQLGRQLKAALPEVIQRNCRNCSPQQAQNAQ
KLTNFLQTRYPEVWAMLIRKYGAV

>AgamCSP8
DPSGSTCAAEATTARTQVSDEALDKALSDKRYLMRQLKCALGEVACDPVGKRLKSLAPF
VLRGACPQCTPAEMNQIKKTLAHLQRNFPSEWNKLVQTYAG

>BmorCSP1
DDKYTDKYDKINLQEILENKRLLESYMDCVLGKGKCTPEGKELKDHLQEALETGCEKCT
EAQEKGAETSIDYLIKNELEIWKELTAHFDPDGKWRKKYEDRAKAKGIVIPE
>BmorCSP2
QDKYEPIDDSFDASEVLSNERLLKSYTKCLLNQGPCTAELKKIKDKIPEALETHCAKCTDK
QKQMAKQLAQGIKKTHPELWDEFITFYDPQGKYQTSFKDFLES
>BmorCSP3
ADLSKYENFDVEPIVTSDRLLKAYINCFLDKGRCTPEASDFKKALPDTIATNCGKCTEKQK
ANVRKVIKVIQQKHSTEWEKLVKKHDPSGKHRADFDKFLLGS
>BmorCSP4
TETSTYTTQYDEVDIKEIMGNERLLVAYIGCLLDKNPCTPEGKELKRNIPDALQSDCSKCS
DKQRENADAWIEFMIDNRPEDWTKLEERYNPDGSYRTKYLEGKHNATSNVDESK
>BmorCSP6
EKYTDKYDNIDVDEILENRKLLVPYIKCVLDEGRCTPDGKELKAHIKDGMQTACAKCTDK
QKVSARKIVKHIKQHEADYWEQMKAKYDPKDEFKEIYEGFLAGQN
>BmorCSP7
RPKTPFDNINIEEIFENRRLLLGYINCILERGNCTRAGKDLKSSLKNVLEENCDKCSEDQRK
SIIKVINYLVSSEPESWNQLKSKYDPEGKYLIKYEAKMESN
>BmorCSP8
RPDDKYTDRYDNVNLDEVLSNSRLLQPYIKCILDKDRCAPDAKELKEHIREALETECAKC
TEAQKKGTRRVIGHLINNESKSWNELTAKYDPENKFTAKYEKELREIKA
>BmorCSP9
RPEQYTDKYDTVDLDQLISNRRLLIPYVHCILEKGQCTAEGKELKSHIKEALETNCAKCTK
AQKGGTEKMIGHLINHEAEFWEELKAKYDPTNEFTKKYETELKRVTA
>BmorCSP10
DYLDVDDIFRNKRLVRNYVDCLINAQRCTPEGKALKRILPEALRTKCIRCTERQKRTSVKV
IRRLKNEYPEEWAKLASRWDPTGDFTRYFEDYLAKEHFNTIPGSGL
>BmorCSP11
QLVGNLRLLKNYAKCFLDQGPCTAEGTEFKKRIPEALRTKCAKCNPKQRHLIRTVVKAFQ
TKLPDLWEELAIKEDPKGQYKHEFTAFINAMD
>BmorCSP12
SLKAFIGCFLETSPCDAVSGDFKKDIPEAVAEACGKCTPAQKHLFKRFLEVVKDKLPQEYE
AFKTKYDPQGKHFDALLSAVANS
>BmorCSP13
VDEIIDNPRLLKAYTFCFNDKGKCTAEGNDFKKWIPESLQTSCGKCSEKQKYLVAKFVHAI
KDKMPDEFDILRKLHDPKGEYTENLDKFLETYGH
>BmorCSP14
EILESNRLLKGYVDCLLGKGRCTPDGKALKETLPDALEHECVKCTGKQKSGADKVIRHLV
NKRPDLWKELAVKYDPDNIYQARYKDKIDAVKGSA
>BmorCSP15

ENDRILQSYTNCFLDKGPCTPDAKEFKKVIPEALETTCGKCSPKQKQLIKTVIKAVIERHPE
AWEELVNKYDKDRKFRPSFDKFINEDD
>BmorCSP17
NDRILLGYFKCVMDRGPCTKDGKTFKRALPEALPTACARCSNKQKAAFRTLLLAIRARSE
PSFLELLDKYDPSRSNRELLYTFLATGL
>BmorCSP18
DKLFTDYINCMLDKGPCEVEYSSEFKELLPEVIATSCAKCTPIQKTGLRKTVKALSVKRPD
DFSQFRAKYDPKGEYEKQFAAFVVATD
>BmorCSP19
FLCLIFLPYALNQKYYDSRYDYYDIDHLVQNPRLLKKYLDCFLGKGPCTPIGRLFKQVMPE
VITTACAKCTPTQKRFARKTFNAFRRYFPETLMELRRKFDPESKYYDAFEKVITNA
>BmorCSP20
NDKRFIQRQLKCALGEAPCDPIGKRLKTLAPLVLRGACPQCSPQETKQIQKTLSYVQRNFP
QHWAKLVRQYAG
>BmorCSP21
GTMQRHLRCALGEGPCDMVGRRLRTLAPFVLRGACPQCSVQESRHIRRTLAYIQRNYPW
EWARIVRQYG
>BmorCSP23
GECAPDAKELKIKLSTAKHLSESIETVCAKCTEAQKKGARRVVITVGSGLSLPLALMKSM
GDGNHSPSGGPSVVSES

>TcasCSP1
QLTRISDEAIESTLNDRRYLLRQLKCATGEAPCDPVGRRLKSLAPLVLRGSCPQCTPQEMK
QIQKVLAFVQKNYPKEWNKILHQYAG
>TcasCSP2
ATYDVYPTKYDNVDIDAILHNKRLFDNYLQCLLKKGKCNEEAAILRDVIPDALITGCRKC
NDHQKVSVEKVIRFLIKERNSDWQQLISVYDPKGEYQTQYAHYLEKI
>TcasCSP3
ASVPYETVDIDKLLADDKMVTEYMACLRGEGPCNPAEKDLEEHIPLVLGNYCADCNDKQ
KNFVIKLATFVIKNRFDEWRQVQKRFDPDLSHADDFNKFILGS
>TcasCSP4
EDTTHKYTTKYDNIDLENVVKNERLLKSYVDCLLEKGRCSPDGLELKKNMPDAIETDCS
KCSEKQKEGSDFIMRYLIDNKPDYWKALEAKYDPDGTYKKRYFESQKDEVSKVEA
>TcasCSP5
KTLHRSTRDDKYTTRYDNVDVDRILHSKRLLLNYINCLLEKGPCSPEGRELKKILPDALVT
NCSKCSEVQKKQAGKILTFVLLNYRNEWNQLVAKYDPDGIYRKQYEIDDDYDYSELDSA
KK
>TcasCSP6
APAEFYESRYDHLDVESILNNRRMVNYYAACLLSKGPCPPQGVDLKRVLPEALQTNCAKC
TEKQRTAAYRSIKRLKKEYPKIWEQLRAVWDPDDVFIRKFETSFESGKPSGVISTNTSPPSPI
LSNRFGENEEADAASNVISSTPLPPTTSTTTRTTLTTKFTTKPSTKPTNKPVVVTKPPQAPPF
ATVGANLQATVSFGTNLVGGIVRSLGTLGSRVVESGTKLANMVISAAIRP
>TcasCSP7
AENKYTNKYDNVDVDKILNNDRVLTNYIKCLMDEGPCTSEGRELKKTLPDALSSGCTKC

NQKQKETAEKVIRHLTQKRARDWERLSKKYDPQGQYKKRYEEHVATSRAA
>TcasCSP8
QLGLAGNNYIEKQLLCALDKAPCDALGNQIKGALPEIIGKNCERCDSRQVANARRIARYV
QTKHPDVWNALVKKYSV
>TcasCSP9
QEYLVPQNIDVDEILKNDRLTRNYLDCVLGKGKCTPEGEELKKDIPEALQNGCAKCNEKH
KEGVRKVIHHLIENKPNWWQELESKFDPQGEYKKKYDELLKKEGLAN
>TcasCSP10
KNYFNCLMERGTCSPDGEELKKALPDALHSGCSKCTEKQKEGSRKIIHYLIDNKRDWWN
ELEAKYDKDGVYRQKYKDVIEKEGIKL
>TcasCSP11
NYVNCLLEKGKCTPDGAELKRHLPDALHTECSKCSETQKNGSKKIMRHLIDHKRDWWN
ELEEKYDKEGEYRKKYEAEIKGKKD
>TcasCSP12
YVNCLLDRGKCSPDGQELKNNLADALQTSCSKCSQRQKDGSRTIIRYLIKNKRDWWNEL
EAKYDPTGIYKNKYADELKAEGIVL
>TcasCSP13
ECLLGTGKCTPSGEELKKDIPDALKNECAKCNDKHKEGIRKVIHYLVKQKPEWWEQLQK
KFDPQGIYKKRYQNYLDKEGLKA
>TcasCSP14
DCILGKGKCTPEGEELKRDIPEALQNECAKCNEKHKEGVRKVLHHLIKNKPNWWQELEA
KFDPKGEYKQKYNKLLEKEGLQA
>TcasCSP15
VLDKGKCTKEAEKLKKGITETMKNGCVKCEQKQKEDVHKVFQHLMIHRPNWWHELET
KFNPHHEIKLQHLHQSKFNPHEEVKLQHLHQFPHHDFLEREGFIR
>TcasCSP17
DCILEKGKCTPEGEELKKDIPDALQNECAKCNEKHKEGVRKVIRHLIKNKPSWWQELQEK
YDPKGEYKSRYNHFLEEEGLN
>TcasCSP18
NCLLDKGRCTPEGKKLKSTIPEALSTDCAKCNEKVKANVRKVLHHLIDNKPDMWKQLEA
KYDPSGEYRSKYKDELEKNGIHV
>TcasCSP19
FNCIMDRGACTPDADELKRVLPDALKSDCAKCSEKQKEMTKKVIHFLSHNKQQMWKELT
AKYDPDGIYFEKYKDKFDS
>TcasCSP20
KEILQSDRLTENYVNCLLEKKPCTPDGEELKRVLPDALKTSCAKCTDKQKQGAKTVIQHL
YKNKQDWWKQLEAKYDPEHTYVKAHEDELKAL

>AmelCSP1
EELYSDKYDYVNIDEILANDRLRNQYYDCFIDAGSCLTPDSVFFKSHITEAFQTQCKKCTEI
QKQNLDKLAEWFTTNEPEKWNHFVEIMIKKKDEGA
>AmelCSP2
ETEEGQSGRSRVSDEQLNMALSDQRYLRRQLKCALGEAPCDPVGRRLKSLAPLVLRGACP
QCSPEETRQIKKVLSHIQRTYPKEWSKIVQQYAGVS

>AmelCSP3
RPDESYTSKFDNINVDEILHSDRLLNNYFKCLMDEGRCTAEGNELKRVLPDALATDCKKC
TDKQREVIKKVIKFLVENKPELWDSLANKYDPDKKYRVKFEEEAKKLGINV
>AmelCSP4
EDKYTTKYDNVDIDVVLNTERLLNAYVNCLLDQGPCTPDAAELKRNLPDALENECSPCSE
KQKKIADKVVQFLIDNKPEIWVLLEAKYDPTGAYKQHYLQNRVKEESY
>AmelCSP5
QDDISKFLKDRPYVQKQLHCILDRGHCDVIGKKIKELLPEVLNNHCNRCTSRQIGIANTLIP
FMQQNYPYEWQLILRRYKIMKYY
>AmelCSP6
EDYTTKYDDMDIDRILQNGRILTNYIKCMLDEGPCTNEGRELKKILPDALSTGCNKCNEK
QKHTANKVVNYLKTKRPKDWERLSAKYDSTGEYKKRYEHGLQFAKNN

OR sequences

>CbowOR1
MIGFLKRQTLVDKLKIHVTFFFILILDVLILVKISTTHNKTLEDIMTSYEAVGSYLQMTTKIL
TLIIYNGDLKQILAMTNQFWKYDKFGPVISNKQQKYPRMMPSFITAYFFFCICTLTVLMLK
PVLFHELPRSCCIPEGEVWFYVVSAIQNETLFYCTFTVFAFDAMFALLYTEAAMQFCLLNE
AFSRMKNHGDLKECVDYHVFLYNFVKKLNDVYWMFLLVQSFDCLSETCFQLLTMVHTQ
ENLTLRVKAVLYAIALYMQLSFFCFPVGFLQDESQASSTAISACPWYLKDAKFKRSVFIVMI
RAQKKISVRAGGFFEMDRQAFIYLCKSSFSVYTLLKSIN
>CbowORco
MMKFKVSGLVADLMPNIRLIQASGHFMFNYHADNSGALHALRLGYSCMHLVFCLFQFGC
TFGNLVVERDNVNDLAANTITVLFFTHCITKFVYFAVRSKLFYRTLGIWNQANSHPLFVES
NNRYHALALKKMRTLLVCVMATTVLSASAWTGITFVGDSIHHIKDPDNENETIIEEIPRLLV
KSWYPWDAMSGTAYYASLIFQIYYVFFSLAHANLMDSLFCSWLIFACEQLQHLKEIMKPL
MELSASLDTYVPKSADLFRAPSANSQDNLIENDYNAKNEEINLKGIYNTRQELGINFRSGA
LQTFGQGGGGVGPNGLSKKQELMVRSAIKYWVERHKHVVRLVTAIGDAYGVALLLHML
TSTVMLTLLAYQATQIGGVNKYAATVIGYLVYSLAQVFHFCIFGNRLIEESSSVMEAAYSC
HWYDGSEEAKTFVQIVCQQCQKAMSISGAKFFTISLDLFASVLGAVVTYFMVLVQLK
>CbowOR3
MATITNSLFVILEIGICIIKFLPFKNDPKKIRKTLFALNQDMFNRATESQRRFIEETEAACRNI
FAIFMTFCLLSLFSWPIKVLFYEQRRFPIDVWLPFDPFENVSIYLGVFAYLFIATGNAPIGNA
AIDTLIAGLIIHAACQFRILKDNLRCLSQRADEKLNGLPQELKEMKRNEIVYRNIRECILHY
DAIYDFVKEVEKTFSVVIFSQFAVSILVICISCFQLSIAEPLTITFFAMVIYVVSLLLEIFLYCYY
GTVLYEESNTLIAAIFDSEWYDLDEKSKKALFILMERAKRPMMLTTGKLLSVSLETWTMII
RRSYSLLAVLKNHQ
>CbowOR4
MLFYVCGFAVVICEYMMFKESIKDIGKFVSHIGMVLTHLAGIVKFCLLTIGHGKILKLMHV
LQNKDYQYCSLEDSKPGEVLRKGQTVNNVIAYSTFVMYTLVGITGHISSVRNLNEQIKGD
NFEGTNKTCYDFLPYMFYIPIPSETKWQCEMVFNLMDIGFALHAFVIAAHDGIFAGLLICL
KSQLLIVCDVYKTIRQRSLKNMHLPENYTITNDMENPALENEMYRLLVHSMEHLKILLWV
RDELEYIFTMVVLTQTVASLFILASNFYVASTILTASLEFFAKLEYTFCIFFQLSLICWFGDDI

TRASDLIKLSLYESDWLSSSPRFKHAMVLTMIRMQRPVFLSIGKFTPITLSTLVAVCRGSFSY
FALFKSIQK
>CbowOR5
MIKMIQIRFHTLGFLACMKIFMLSFGQLLSKTLPLKCYSAKWIPFHVMWFYQSFLTSVCII
MPIIAMDLLLMTFISLTHIQFKMLNLEIDRVFRRSERAKKSEIARLVDHHNFLIDFSNRINNT
FSTMLLAYIFVFVISMCVEMYKSSANPSFSVFMNAVTYLSAAVFGLIFLFCIPGQNLTDEAN
NIPNAVYFTDWYRDSKQSTSVLMMISNGQRDISIKAGEVIKINLATSLSTIKTLLSYFMFLR
TVVLDE
>CbowOR6
MYTIKKSQPFYSSLRTLRFFLVYREFVQKSTFMLLSFFSSFMSFSAFLVFVCGCILHAVMSIR
ENIGGDISEDLSVSIGGLAMMVNVAMFKYHQDKWSNFFKDVTNFEKFGKPTDFDATKDR
ANLLSTLYMIYCITGTIVYSCVGVIESSCDELSEETKQKVICGTLAPIWLPFEDVSLTVRNTI
LLVQYVLANYIITPSAVICFLPFETTELLICHINFLKDKLLKVFGNEDGMIRNDKLRFCVAYH
THILGMADQLKYVVKFSVGHMSLVCALVFGCIGNQIFRAKPVGAVIFLLGYMVSLFLLCY
AGQRIMNESLSIVDVIYNSKWYKGNTQIKKNVRFMMARCQIPVTLDAWPFGIFSFPLFMM
IVKTSYSYLTLLRQST
>CbowOR7
MISPTSYLIKLIIFKSKSVHVLEMLSFLELTEFNNYPKGLSGIVERTVKFSRYLGYAYQFMCC
LVITLYSTIPLFTKADLPIRFSHDVGKLKPAVYIFQVIGLSSAASNNSCLDVLAMSLMGICSA
QIDILNKKLITLGKNEDEDETDGSNNSYLRLKKCAKHHVEIIRFQKALERVFSSIFLAQFATS
VMVICNIGFQLVHVQPASVQFALMLFYFIAMNTQLVMYCWYGNEIIVKVYHCLSILSSAIR
DACYKFEWFDSNMETKKLLLIIMEHSKKHLYLTAGKISVLSLESFTSVMRTSYSYFALLQT
LYRNNQD
>CbowOR8
LPFDPFQNVWTYYGVFLFIFLGVASAGVGTPPVDTLIVGLINHAAGQIKIIKNTLEHLDHDT
NKVLNEYRYISANQREILKNKMIYKRITNCVIHYDAVYYMVKDLEDTYSSVIFAQLSASVL
ILCITCLQIINVEPLSVPFFAMCTYVFSMTAQIFLYCYFGTILFEESDSVIK
>CbowOR9
MLTFSLIHITSLVQVSETLSFNLTQLAYLCKLLNFQIHSKRLLELEDFLRKTTLTNVTVEEEA
IIRNTMKGSRRLATVYRSLCVIIVFLYALFPLIDENSGDEKKLPLPMWFPFDTNNHFGKVWF
FEIFSIAIGAWTNSNLDVICVTMITLTTCQFNIMNSRLSNLRKSTDDVEEEDTVQKALKECV
IHYNDIISFQILVETTFSLSIFGQFVFSVLVICMTGFQMLVISFKSVQFVLLLSYLLGQTCQIV
MYCWYGQSILDSSEAINDACYSSEWFNCSKETQKMFLIIMERSKRPVKMRAGKFFFLNLD
TLMSILKSSYSYFAVLRHIYSSKFT
>CbowOR10
MMVKQTKNEAKRFFRYIGMLFTPTQALGCYVLLRIKQHGIEKVREELLDEQFHYKSCGSF
RPGKIFNDAKSFCDKFVVITIILYSLVVASAHISAYVTLNLAFEGEYFPANITCYDFMPNYFV
IPFPTPTKSSCKNALTYMDVSLNVYATLLASYDTTFCSVLICFKTKLQILSGAMRSMRERVT
TEMNLPLNSSLELDDPEVEAKLYEEIKQCARHLESLLSVCKQIEDIFKYGTLMQIVNALVII
SSCMFVLSITPQSDPDFFVMIHYIIALFVQLFTVCYFGNEITEVADELNNSLYQSNWLSCSK
RHKQCMIIMMSRMQKKIHVMIGKFSPLTMNMFVAVVKGALSYCAVFRAVDNAEI
>CbowOR11
MGAVKVLFFYFRGDKLIKIMATLESTDLHYEKCQKRKFFPGSISTNYKKVGIKYTLLFFML
AHATLISSYIPPTIAAIQSELDNPGKSLPDRLPYYSWMPFKFDTSTTYLIALGYQAIPMFSYA

YSIVGMDTLFMNIMNCIGMNLEIIQGAFLSLRERAADKIAGPLMTQDGLHNSHELKTALN
REMKKVCRHLQIIYRLCEDLENVHTFLTLAQTVATLFILCSCLYLVSTTPASSKQFLSEIVYM
VAMGFQLILYCWFGNEVTLKADMIPFFIWQSDWISADREFKHAMIFTMIRAKRQLHLTAG
KFAPLTLTTFIAIIKASYSFYAVIKNTST
>CbowOR13
MDYGYPKNFFEANDVVKRISGIMLLQGKEDNIFFKWYQIIYIIFVYSSTVVFTVGQYIMTK
NSVNKISNLVSSLGVLLTTHVGHFKFWLLLSKKKELENLKNDIEGENYQYATIGNSNPGLL
LTNEKKFCTVCTYVYLAGCYLIGIFGNITTVVRLNGALTGNNTFESINMTCNDFASFTFYIP
FFTANKWQCIISSTVMYSGMAFESGIHAACDLLFFAMIHCLKIQLRIIADVFRTIRRRSLLKL
NVPEDYTVLHDEENSALEEELYRQLSHSTEHLNILLRVTQEIEHLFTYVLLAQTLSSLLIVA
SFLYLTSTISINDADFFLQMQFLLVILIQLALLCWSGNEITEGFQLIKTALYESDWLSCSHRF
KRSMILTMIRLQRPVLLTLGGFSPLTLATLVGVCQGSFSYFTLFKSFQ
>CbowOR14
MATEFQKAFETEKWILSLFGFYPQWNPEPLWKHARRVFCIAVTLTYIISMCTGPFLENSVMI
LCTVMGLSKMVQLLTSKRQFREIEHYISNMKPSIIRRSSLIGAFRVSVVTLVGFLGIMPLSM
KNQRMLPYKSWLPYSVEGASPYYSTFIFEVISIVMAAFTNSTIDMMYYCLVDICCAELDVL
KLNIIEIDMSDHVDIVEDELKKIVIHHHKIIRLVGIIQEIFSSVVFVQCMASVLVICFLGFQLIY
VDKLPSVKALIELSFIACMLIQIFCYCWFGHNITMKSSEVGDICYHTKWFESDLMIRKIILII
MERCKKPVELRAKIFTLNLQTLLAILRSSYSYMAILRTLYTDE
>CbowOR15
MKEVHFKNTVLNFYEFYNTDFKLLKFFGIWIPDSSNSKFHKIYFIVINFVFCAIFNLAQVSN
LLHEINNLKNLAACGYVVAIACMANVRSYYFLKNREEFLYLIRSLNDSHFQPESEDQICSA
KKSLRFYSKVKMIVSILCTITVFISMSTPVFYKKNELNLPFASWYPFDVSSYPIYQIAYVHQ
CISVIYVTSINTYVDIIMAGFNTFIGIQCDLLCSRLYNISKDHSSEENETTLLDCIRHHKLIVR
FANNTEILFNRIYLGQFIACTSALCMALFLLTLHQESRFESSFLVFYLTAIFSLLFIPCWFSSE
MQGKSENIPEAAYSCNWVTASKLFKKDLIFFILRAQKPLKFYAVGFFQISVETFVLIVRSSFS
YYTVLNNMIMKEG
>CbowOR16
MDIVVLTLISLTAIQFKLLNKGVAKIFKGITNLEQADVVIRERIQKYKNHHAFLMTFRNELN
NLFSNAILAYMGVIITTQCTELYVLFSWNSIQEGIRAVLYASTMFFELYICYCLPAQDLIDEA
EKLPISIYCSKWYQYPNHFKDVLLLLGQFQLNMLISAGGVAILDIQTGFAALKSMVSYFAF
LRTVGASSEK
>CbowOR17
MNIEHIKEIEVLENSLKFLRIFFLFPRKSEINDPKKNVYWKFILLSLSTAYFSIGAAIHLVVNV
RSGAFVNVDKDVGTIISYYGALYFISRYLGNIKYIIILYKQFSDFKTYGLPNNFEKTNKLLN
KFSKIYFVYHMFIVTGMTTSTLLTIGTCEEENLENNINDICGLVGPTWLPFEFDYFPLKQIV
YGYQVYCSFVIFQLAGHLSYTLMESVEHLIIRFEHVGHTFVEALNEKNSYTRREKFYVAIQ
YHNDVIQMGKLLNSCFAPSLIVHISLTGPVLGVAGYRFLTEIPLDSTCLFFGWMFSTFIVCR
GGQRLSEASLAVGDVIYRVNWYNLETDLQRDLKMVMLRSRKPVYLRAGPFGPMTYSTIV
TILKTCYSYITLLKQTM
>CbowOR18
LGVDVLVLSVCACVVQQYKILEYLFLNFNTPRMVEINEMFEKMGEEVNSSYDAQKKYLV
RCIELHQIVLRVTRNINKALSSIQLFQLSSSATSIGVGLFQLTKGSSTFFQYTMASAYIFANL
MQLLIFCSVGNELYYQASLLPQSIFLSNWSESSVELRKDILIVLQKSQQVPEL

>CbowOR19
MFRVGDAIAFQSTINYMTFFKVFTVQTDTRYVGTILRFWSFCLICLFNTFHLVYVKIENIDV
DTSEDLVVILGGMGILLICIFSASSSRRWTSFLHNLIDFEKYGKPDGIEHAIERGNYWAHFF
GLYYIIGTVIYGIVTYMEAPSCHRLNNEKNLHLICDTFVAIWLPFDIPLKSIRLSVFFIQFVLI
TCNVNPAAMACFLTWECTEILRCHLRHLKKHFHKMVKEGDVRKRPDGIGYWIRYHNHIL
SLSYELKSLFKISVGHTSLISGLVIGCTENQILKSKPLGASLFFLGWMAAMMLLCHAGEIL
MEETLSVADTLRDSQWYLADLETRRDMVFIMLRSQKPVHLEAMPLGVMNYALFVMILRT
SYSFMTLLNQSS
>CbowOR20
MRDVEEAEKSLMVTGWFPFDTREYFAVAYLFQLQIAIIGGLFLVALDSLIISLIMVAPLRLKV
LANYFRHFGDKKSMNSLLSLKNLISEHQGIIRYVEDLNASLKWLFLADFVVKSYNISIVLS
NAVSIYEFRNIVIIIYYVLQTRGRNKSELAFSALFLCFLLSQLYCFYFHANEILLESTNLAENI
FKSKWYEQNSQIKRSLIIVMIRSQKPLQITIGDLHAIENILFVKIVKAAYTFLLFQYLGL
>CbowOR21
MKTQFQKAFQKEKWILTSLGSYPQWEPEALWQHCRRFFCMTVSITYITLMCTGPLLENSI
MILCTVMGVVKEMQLLFSKQQFREIEEYIGSMKPCKIPISRLLGSFRNSVVTVIIFLGLMPY
TKRSLRMLPYKSWLPYDVSRAPVYYYVTFVAEVLTIIMAAFTNTTIDVLYYCLIDICCAELD
LLKMELMEIDMSESYEVVQNKLKNVVIYHQRIIRLVEVIQDVFSSVVFIQCMTSVLVICFLG
FQIVYVDEIPSGKATIEVSFIGCMLLQIFSYCWFGQSIMMKSLEVADVCYNSNWYDADLRI
RKMIFIIMERCKQPLELRAKIITINLQTLLAILRSSYSYMAILRTLYSD
>CbowOR22
MSSFSESPENEEQPFSATTKMMRLLCVYPLGFEKWQMVRFYVNVVVVKLFSFFFCCVLCL
LHLVMTKIDGAHKADLSEDVSMIMAGTGMLATNLLFAYKVKKWNSLMGKVADSPEMRN
IQNFEAIKKRCNRLARLFTMYCVIGAGIYLLSGYYESLVCIRKNEENGSNEICRTLMPVWL
PFRLSSAAELTLFALQAFAGINLSLPGANMPFLVWEITEMISLRISHLKKISESIVVEKNIKSR
QERLKHWVMSHQQIIECISLLNEQVRLCFGHISTIAALVLGCLANQAINSVHLGAMAELGG
WMVGLFLLCSSGQKITDITESVAEAIYAMEWYSTDVQTMRDIRFILMRSQKPLVLQAGPL
GALNYPLYMMMVKASYTYLTLLANTI
>CbowOR24
MMTEKDEKMPKKLEIVCMPTSIRIFRFYCAFPPSDKLLNPGKMFYIRFALIALFSSVVLVGS
TMHLIKNVKDRTYNHIELDFTYIVSNLAGYGLLCSYFTKVNAAVQLYLILSDFEEFGKPINF
DNTNKKFNKYAKYQYCYLESITVCILLGSNMFRGAQCRKDNAELDQHEVCGLFAYTWLP
FDIDFFPVKQIYLACQLFGIHYVYMMAGLASWMVLESVEHIATRLRHVSHFFNEALKEAE
QRKRREKFNFAVRYHVAVLDLESKLNQTFSVFMFTHMVMSGIIMGYGVYSYMKGKNVST
ILIATGWLIGLLMDCYSGQRIQDESTLVGTALYDADWSDADDELKRDIRFVMMRCQKPMI
VQATSFGIMDHPLFLAVLKATYSYVTLLSQSDL
>CbowOR26
MKHKKLYNFYTIFCTSVWVTFILSQLVYMFSSFSNMDEMTSIIYVAGTVTIDLVKMLAIYS
NMDRIKPLLNDLNNPLFQPKCKEHVELALAVKKFHSRLFYFCLYFGVQTYICFSAIPFILEE
NVTLTQGWFPIDWTYSPNYEIVYAFQNIVILWNTLIFLNLDTFTSGLLMQVGLQCDFLSTTF
NKIDAFHVSSGVLIENGEQMALSLKDNHEFFNRVMTENLIVCVKHYRKIRRLATEIEDIHHI
SVFILFLGGAIIICADLFQLSIVQTGGVEFVLFVSFLMCMLMEQFMYCWFGNEIIFKSDNIFA
ASYNTPWLDCDLKFRKILLNFMTQSIDPIGLKAGGLFTMSIKAFVSVLKSAYSYFTLLQRIQ
EKECSELN

>CbowOR27
MIPSEDNSLSSMWLTKLILKSIFMWPDDYSDTKRKTFYKISMTICLFIQSGLVLNLTQNYHD
WEKNLAVVSSMSTIFQTVFKMTALYQNSDHIKFVLMCMCRKFWPHNLDNENSEYIFKQS
HSRRMRLMVFLLASGFLFSLGSVISPMFTRDTPFKSDYPFNWRRSPFYELIYLIQVAANGY
LINMTVIGFDFLFMDICAALTNQYVLLGSCFERLGTENMQDFYARIRERGCQKWPPKVGG
ARRFLGICVQHHQLLTQITKVVGHIFNVVAFLQLCSSVVAICVSGFIATKDDVTTSQIATMG
SYLIGHLIQLYIYCSVGNELLFQSSTLTNHIFGSNWYNLDSTTTKKDIIFIMKKAQIPAKLNA
FKVFPLNFATFIAVVRLSFSSYTLLTSITNK
>CbowOR28
MYASNLDWCLKLNFLLGVHPAKQKSFTQTLQYLFIIFGSCAIMILTVLLLYYKEDAVSMKD
ITDVSTNFTMFPHGMIKLTTLYMKRAEILDLLRRTKEHFWQIKDDREDVKRSYKLAKLLK
NLFFNSVVLFIISAIVKPIIIGGNTLTYKCHKPELIPRWLFLIFQDAMCVAILFTLSCTDVLILT
LLILTQIQFRMLNEKIQTIHDNEVDFHDRLKECVDHQNFLMDFVDRFSKVFSKTILLFIGNII
LSLCMCMYIITTESANINVQMEALFHLIAGLNEICLCYSIPAQTLMNEADEVGKNAYFSKW
YEHPKDAKLILQIMIRDQKRMVITAGDFVRIDMEMFLTACKTIVSYCMFLRTMSMVDQ
>CbowOR29
MTCNEFASFTFYIPFFTATKWQCIISSTAMYSGMLFEAGIHAVHDGIFFGLIYCSKIQLQIIGD
VFRTIRQRSLSKLNIPEDYSVLHDEENPALEEELYRQLSHSTDHLKILLRVQDEIKKLFTYVL
LAQIISMLFTLASFLYLVSTISINDPGLFLQFFWFMTILLQLTLFCWSGNGITEGFDSINTALY
ESEWLSCSRRFKKSMILTMTRLQRPVLLSLGGFSPLTLATLVGVCQGSFSYFTLFQSYQ
>CbowOR30
LIRVSIITGFFYGGLALSQLINMFVSLGEGEIHEIVTATYLSLTNIFSVVKVLIIFVNRRKITTL
VEKINKKEFQPKTKNQREILEGYIKLSKNVTSILIGISSSACLFWAVYPFTVEGGPFLPLAAY
VPYETVSWPIFGITYVSEMIGIVISAFCCTNADSLITALIMVVCAQLELLNDSLKTIGEHSKI
ELKK
>CbowOR31
MMAFGYPKNFFHMNEATVRFLGVWLPSKKHHILIRLLHPFYFIFVYSTLIYFVIGQYMKVE
MKNVTTIISSLAVLLTTHAGHVKGSLVVFGGRRIQEIKDILQDVNYQYYPVGEVNPGSTFQ
KEKTFYTMLSYALLVGFMMPGASGTVTTASRLMIEMKGNNTFESIDKNCNDFITYTFYVPI
FIETKWECIISSSLMYSGMTMYEGIAHAAHDGLLAGLLICLKTQLLILGDIFRTLRQRVLSR
LNIPEDYSVIHDEENPALEEEMYRQLCLCTEHLKILLTARDKIEKTFTYMLLMQTIASFPVF
ASSLYAASQTPLSSTDFYTNIDFFGCVLVQLAMFCWFGNGITEAGEAIRSALYEGDWYSCS
PRFKKSMILTMTRMQRPVYLSIGRFSPLTITTLVSVCQGSFSYFTLFKSL
>CbowOR32
MEEDQKIYHFKTLKYILILMGQWKFRNRSRFFIKLYELIISRLVVAYLILSAHMFLINVAFAW
DCKARVMEMLTAYMQNLNVIIVTLIMRSQRMRNVLRYVQHYENIKLKDENIAVRDIYMQ
YVSINHKICRLLIGVVVFTALFYFATGMRNSFLISSTEECPMMKGIMFQLWYPMDTKKYFY
LVVLNDNLIIINIVITIIHAKGLIIANMIFAISQMKILQYELTLVGQIEQETDEDVELRVKKCIM
IHQEIARMMDLFVSASKDIILMQYFITSSELALYLLQMMLADSFAVFGRLLMNFIYLFVEVF
LLFWCANNVLVESMAISDVIYNESNWITLSNGAKKDMLMMLSRAQVPMAFKATFVGNIS
LETFTKLLKLCYSVVAFLSNVVRE
>CbowOR33
MIAGFTTFIGLQCDILCDGIINMGKDRENELRVEEFIEHHKLILRFASTAGKVFSEIYFLHFIS
STSTLCMTLFLLTLVDVNTSEFYYLVVYQSSVFSLLLVPCWFSSEMQRKSENLPNAIYSSP

WIDASISLKKDIAYFICKTQEPIKFKALGVFLISVDTFMAVVRSSFSYYAVLNNLNVKGE
>CbowOR34
MTFFFKIKGLSPPKTTAVKIIYLSLALPHILMCTFVLILSEWMAFALSQQTFKERMFNMSVA
TLDTILVFRTTVWAFNKAKLDEIRAIITRKSFNFRCFDLLKVGCEQVLTVGRTKEVEKERGL
SCKEIKQLWQKAKFVTEKKCNEMDVFRKELMLNTRLLCCFIHLAITIISVLTYTLSFVNDF
TNDTYEAYNPILNRTSLYRKFQYPLYLPFDTSFDGYYWLAYFYNCYAHLGNIITFLPIETTLT
CSLIHLISQTAVLKEAFKYVDENNFGYQSFGEASIIKEIRIVKCINEIQEIYRAVELLENLCNV
QLMVQYGFATFLLCSICYVIPLVENTMEGICCLIFFAASLGQIFTFSYCCHTLALELQAIGVS
VYNLDWTNYPSKLKRTLNISILRTQKPANLTAGKIIVIDLLFFIQVVQKSYSFYTLITKTN
>CbowOR35
MSHNEIVCMTTSIKILRTYGVFPSQSRELHPGMVFYIRFLVLAVVTSLTLVGSTLHLIKTIQN
NEYNYTEMDLVYIVSFVTAYALIGSFVMKVKASGEMFVFLSNFEEFGKPINFDKNNKLFN
RYSKYHYVVYLESLILIILFSSNIFKSKTCRLENELYNLKEVCGLFTYTWMPFNIDYTPVREIY
LTIQLLGNHHIYMLAGLVAWQVFETIQHIIIRIRHVKHLFVEALQEGDVKVRRKKFNFAVRY
HNAVLSAFFSLEDKLNAAFGIFMVTHMVLTAAVIGTGIYCLFRRRSLSSFLVCMGWFWGLF
MDCFSGQRLQDESLELAVALYDSPWYEMDKEFIKDIMFVLSRCQIPMKLRAYAFGVIDRA
MFLAVMKGTYSYITLLRSQ
>CbowOR36
MYFFYVSFLYGTGVIFFVCEFMIFNETIGKISKFVSHIGMLFTHVVGILKMSILIFGRYRILKI
MNVLQNEKYHYAPLEDSQPGLLVVKEKFVSSGISILVFVLYTFVGVSAHISSLITINEEIKGD
SFEGTNKTCHDYMPYFFYIPFPTETKGQCGIAFAFMDVGLGIFAWVIACHDGVFVGLLNCL
KTQLLIVCNIFTTIRARSLKAVNLPKNYKILHDEYNPALEKELYRQLSHCTEHLKLLLVVRD
DLEIMFTFVTLSQTLASLLIFASCLYVASTVPMTSPEFFAQMEYFLCVLVQLSLICWFGNEIT
RASELIRLSLYESDWLSCSRRFKSSMILTMIRMQRPVYLSIGKFSPLTLATLVAVCRGSFSYF
ALFKSVQ
>CbowOR37
MYFNTIQKVLPILYIIGADPREGFTKSQFFLYFYNIFSAIGMVYLLVLKFANAENKVTVKDIT
DAVICLFLFCHGMVKSTTMFVKKNSVQTLLAQMEKHFWPMNNYKYSYIHNGILNICKTIR
NTTNFIWFMHFCNAMGFLVGPLITKDPVLPFECYRPEWMGYYTLLLFEDVTSIITILCPVL
AMDVFFVTIIKLTQIQWKMLNSEIQSMFDLSPSGKISREDEENIMVMKIKKCVVHHNFLLN
YQQLLNDTFSIPLFFFLIVIVLCMCVEMYVISTVSDWESLRTAIVYTATGCLEFMLCYCYPC
QDLSDEADNISYSIYFSNWYRNPEYFRDTQLIMQKGQKLVAIRPGGFMIMDLKTGLSVGIF
PSP
>CbowOR38
MFAGCIFTCTFWAICPFTEDVASLPIAAWIPFKTDSSPSFELAFAYEIIATVIGGITDLNADCF
MAGFIMVVCAQMKILNDSLLNLRHFAVEELNAETGGNNDDDGIAEELQKIMNRKLVECV
HHHRYILEFAEEANSLFTTSILGQFAVSVIIICTTLFEMTLVPFASIKFISLILYQYCMLMEIFIV
CYFGNEVILESSKLTKYAYHSDWRDCSQEFKRNLLFFMTRSQRALKLYAGGFFTLSLDTY
VKILKSSWSYFVVLIQVNKDSG
>CbowOR40
MYEKEFRNVFWLLNFVGMHPLKKYVTPFIVFNAILTFYITVLIILKLLWDKELVAVESLCVF
SQIWLKIFVLTTKKRKIKQVIDDTQQFWENDPENSENKQLLKNLAKLERIFLTYISCSTCMF
LFKPLLVKGTSIYYYYKIPQIPFYVSYPIEFYVTIVTMALAIAVNLFISIVIVIGAGQFSNLNA
KMKQLDLSIAEDCQDGLRTCTLEMNKNIEYHDFLIKYVSHLDEIFSMLFVVLTGIITSLLC

MNMYVLSQPTTTAVDMIRCGTMVCAFTSEFLFLYGVPAQRLMDEAEEVANSAFYHCQWY
LPNIIPLRKSLSFMIHRSQKSVCLSAMGFIDINRQTIVAMIKTAYSFFTFLQTIETTGGAK

>DponOr10
MTIIRSADNEQFIKLAKVALISTGVWIMPITENRSVAFAFKIYSLFMKGSCVMYFSLLFAETI
RLIIFKYDMDVILASVGVLFNAAKIMLKVFIYLKYHILEHFEDVIEKERALWNSDNEELKA
LYRTKVRHCNVFVITVFTSSLMAVTALQLSGAFTAFELAEYSKANNITIEPHVMYQSLFPFS
KLDNLYWWLASQALWWWVGLTYNTMTHAVFAILLIYAATQLEILQIRLRNCIEPEFSETPS
QMLIKEKVLLLRKLTQDHKYVIDYVKHFNECTKYAILLEFLLTSLDTASVSVNIIKMKGAE
LSWLLSFLVLLVMQISLIAWTCNEIQVQSMAIADAIFASRWYCLLDKEAIAYVHFMIVRAQ
KPLLMTIGPFGPMTTASALMVFKAAYSYVSIMKE
>DponORco
MINKFKVVGLVADLMPNIRLIQASGHFMFNYYADNSGSLHILRLGYCCMHLFFVLVQYGC
IFGNLVKEKDNVSHLAANTITILFFTHCLSKFIYFAARSKLFYRTLGIWNQANSHPIFLESSN
RYHALALKKMRSLLYIILFGTIFSASAWTAITFVGESVHFIKDPDNDNETITEEIPRLLIKSW
YPFDAMSGMTYYVALVFQIYYVFFSLFQANLLDNLFCSWLIFACEQLQHLKEIMKPLMEL
SATLDTFVPKSADLFKSPGSATSQDHLIENDFNAKNDDLKGVYNIRQELGNLNFRSGALQT
FGQGGGGVGPNGLTKKQELMVRSAIKYWVERHKHVVRLVTAIGDAYGVALLLHMLTATV
MLTLLAYEATKIDGLNTYAATTLGYLLYSLAQVFHFCIFGNRLIEESSSVMEAAYSCHWYD
GSEEAKTFVQIVCQQCQKSLFISGAKFFTISLDLFASVLGATVTYFMVLVQLK
>DponOr18
MKDRVKLMHSFKFIMILAGLWRLKLTDNRFYQWLYLLYSMVLQALFTLLTFGSIRNISEAI
SNRDYTGVIDNIWLSLLTMVILFKMIMFQRKRFLDLILQSIDEESKIYTEETTCIQEIFEHNY
KIVKHIILLVCVCVSSSGFSVCVCNTITYFQYRHINQTEMKPMMAPHIQFPLNRHKYFMET
YLLSIGTTFGGVIYYTLTQVYFVVVTAFVISQLKIIQRLARDFHLCSNGGDQEKEALTVFRK
IYNKHLYIIDFVGELNVLMKYLIFVECTVISIIIASVLFQLIFVPTSLAASTLYIAVSCTVLSQIF
VLSWISNEIEVESLSISDALFESRWYEQTQKVKKIIIIIMMRSRKPLRIMIGPFYPLTIHTALNS
LRAAYSYVTLIFAMSSSGQLQI
>DponOr22
MICSVAFGCFPWEFYFPDNKSRRRAYEIYGRIMFAYYIVFIFTIFVQLVVMLKRPDFDIDAV
CANMCVTLINTVTLFRQLVFHFNPKFKKIIQKVIDMESQVYRKGDEKICSIYTKYVKGINK
SLKLYFCFIGSLLVIFCVRPLLADPVEQRVGDEVKLVRPLPQSSWFPIDTEEQYLYVYTWGC
INCMISAFFVTCSDLIMFALLTQPMGHLNILHEILQNFDEHKRNFALRNQIVNDDIAAYWTL
VDVIKHHNEIITYVNEINDCMSFVMLCDFLQSSLQVACILTQALENDIDVFLVLFMISFIGS
MFLRLILYYHYGNELLTTSQNIALSAWQSNWYDQSPQVKIMMFTVIARAQKPLKFYLGQF
GVMSLQAFISVLRASYSYMTLMYGLN
>DponOr2
MKFFKKTEENVFFGFNIMVLKACGLWPDLIDYRYDKWRLMKDCLMISSLMPCAVPIMAD
FIMQLYDGVPNLTAAVENMIALNCIIGMIYMVICFIANRRTIIKLMVNLKYFNKYGNSRKTK
EVDEKANLFSKIFMFYGILGNFVYMLMPQLSIDKCHNNRTAKMIDDGVPCGLVVRSVFPF
KFDYKPVFEIIFVHQIYTCTMVSIVVLALTMLLCGFLMHIVNQLKNLREFIAKLRHCPQDK
PGERLFFIIQYHIDVIEYSQNTAKAFSTMLLFYITLTSLVLSVLCFEVIMVDAFEDSVRFALH
LVGWLAILLSVCYNGQLMIDESVEVANDIYSLNWFNFPVGIQKKIQMIIMRSQKPLILDAA
GMGLVSLPAFLKVLSSAYSFFTLLLKLK

>DponOr38
MKPSKTKKAQLISWTKVFMIGGGFWNQPLTNSYIGEKVYFCYSIFMKCGCFMWWSMMV
GELFRLVAYGYDVEIILAQFGLVVNASKIMFKLVVYIRENLLALFKDITEKDVEIWNLDNEE
IHTVYWKNIKLIKSYVLALSVSTSLCLGMLDVSGIHVILKTVEHNKAFNDTLEAHAMYQTI
LPLNKLDNLPLLFTLQAYLAIIGFVYNCLTHLMFATLLVYAATQIQILQIRSKNFIGADQLSG
SDMRDKLLVLKEISQDHQYIIGFVENLNSRTRYIVLVEFILSSFDLASVSVNLITLDFSSSDIA
GQLIFNLSFFVLLSIQISILGWSCNEIKCESEELANALYASNWYLLNPKGQKMMQIMMARA
QKPLIMTIGPFGAMTTNSVLAILKGAYSYVSIMRK
>DponOr35
MKNDFFGFCIPLAKYIYILPDISTQDRTKPLLHKICAVIIYVLALFCYFSEVVKLYQIVTGEY
FVYDELIRNYTVSYFHFTSLIKAINIKGAISARAFKTIIGFEDNIYNGEDEDIRKVYKASVTPI
QRVRKYYMAGMVMVVICYACAPAFRDPIEIQRENETIRIRQLPVSAWSPVEEYFWLDFVW
KSLVGAYLAYFFVTTDLILYSFIAFGACQVRILQHYIHNFNRYCEEIMHTEGVPKNESARLL
QKQLIAMHQDVISYVNMINGSIKQLMMLEFIPGSVQLAGMLYQLMTNLNAIQCIFLGQFIS
CLIARIFIYTNSANDLSQLSQQLAADWFEIDWIELPKDIKMNLNICILRCQKNLCITVGDLN
AIDMTTFLTILKGSYSFLTLLTTI
>DponOr20
MLYPVRKGLPFYHNLLVLKLAGYYPKSSNYNKKLFFYCLVCWMSLWTGTLWNLIILLYLS
IQNKSSHGITEAMGYLIGNSSLALICLHFALKHQDWSHLMDALIDFQKYGKPPKFNTVQT
NASKVGITFFKVLFCAAVMYCVLQVLLEEECEKKLTFGKTSCGMLLPTWFPASHAESKLA
KRLLLIYQLLACWAIAPFTVIVSLVLQANEFIAYRIDHLKSLLRKVGSNENPDLQLHQFLAY
VQYHHHIIRLCRKLNYVAKYTTGHVALTFVTVVACFGHHSIQEKSLQSLTYQATYVISMCI
LCYAGQNMQDQMRSIGDALYSSTWYNCSLKVQKMIPLVLLRTQQPIGLDAVPLGVFNYM
LMVMVLKTTYSYMSFLSRTI
>DponOr25
MAIYPKCRLIQISMISSSLLGTFPWQFLFQDNKTFKNMYAMYSKLMLGHFTLFLFTAQLQL
WILITDEELMRNAIFANLSVTFIYNITLAKQLIIMLNSNFRATIKQIIETENCKSPIEDDEVTN
EIEFKMVQRSDKIVKCYGFLLIVLTILFFVKPFLMTPTIVSIGNTTKVIRDLPISSWLPFDEQE
HYSYAYIWQVLNALQGSTYVASTDILMFNLIVFPAVQLRKLQHLLKNFAHYKEKVKTLYNI
ADDEQAAKITLVYFISRHMEIIQYVRRFNESMEIVMMFDFLQSSLHIASILPEVLMSEFSVM
VVLMVASFLVSMIFRLILYYYHANNVMILSAELSYSMYESNWFDQTPKVQQMILIFMLRA
QEPLTLRFGGFGVMSIESMIAILKATYSYVMLMI
>DponOr24
MSVLGETTQIKKKWRSSIAITEKVLVITEIWPNDDTSLYRTMKVVFITIVCIVFNLTVIDELK
MLAIRQDYKTLSMHLSTFGLYIGFSVKIILFQFTKHGPLKNMLDSMDSPIFHAYPPEMQKH
QDNCIRVSNLIGKFFVVYLVGGTILFYLNKPFYSSYPLPITFSHPLTTTTFYLLLTLQCVCSFYL
IMIGICFDMLVMGLANVATAQLDMLIEEITTFTPTSIETLEKEEHRFIKRCAERHNAIISYVNS
IEDVFTYIFLAQCVVSVTCICNGLFQLTHVAPVFSIHFYYNCIFTFNVLFEIGICCWFATLMT
NKGNDVADACYNYNWLHSSSATRKLLLIMLCRSQKPLFITVGKIIQLSIGSFLSVLKTAYSY
YALMQHLYDKTSQ
>DponOr16
MDFIQLFAPFKLILNISGFWPQKNPKPIVEVRKVFTLLVNLFFCLSLSIQCLFLRSQIEEFLDV
LTVITPPVAYLFKQIVFFGHSGAFLTLMDFLKDDDLVSIPLQLRKQISDSLQVAKIIGVGYQA
CCTMTILFIVIWPMFTEHQLPVQFTLFDLGDFYAFMYLLQIFALANAAANSSSLDLIALTLM

CIVKGQICVLNDKIRSLGEINASKGHGAQRVKYVSGCVLHHTKIIELVALIENVYSQIVLIEY
LTSMVVICNIGFQLVIVELASFAFLLMLTFLVTMLCQLGMYCWFGNEIMLHSAAIRDACYE
SDWIHSCPQVRKMLLMIMERSKRPLYLTAGKFSILSLNSFTSVIHSAYSFFALMQRMYGKS
TTF
>DponOr30FIX
MFIRNQRAFVARTCKLAGLYPVQLLPEDENLRKLYTIYYQALIMLYFICLISFCTELFHLLR
AEKATVDDILKSISMTTLFAMTALRQWVIRSSPDVQKILRKAGNVEQRVYEENDPEVVNIF
ERAGHVALLYYIYYAVGTVFLCLGCILEPLYDNQKVFSGNATAFSRKLPLPLWFPYDIQAH
YWETFCVTILLICLLVVFQVAVDVLFFYFIRSPVIQLEILHHFFKRFNDYTGRISVEPGNVAS
NVMMRKCIDMHRKVIKFVDIFNENFSNIIVLDFVQSSFRLASISAAIIMIESFTVTSFVFTLIF
LWITLVREYYIYHAGNEIIFLSSGLVHSVYETDWYIENRQFKYMVKMFMVRAGKPLDIKIG
RFGSLGFPALLSILQASYSYVTLVRGIQKS
>DponOr45
MAKTLSFIQLSKFYLLLSGLWPFKISDNFLVDKIYRIYTLCQICYYLCVIFGLLVNLVILIIRF
DQPQRIIGDINLFIIVFECCLKVVIFQIGKVPFMLNQITQFEDSMEASGDAEVKDYYAKDAI
YCRRINVIQSIATIIACASFAQDSVVTFITSDDMSVFKDNPFMHDLWYPFNREDYIYLVICIA
FICDMQGLVCNAACQTTLLCLMIYARTRLKILQIRLRKFDKIAVEEYEGDVVRAVKDLIAE
HQYLINFVKSLNDRTQHVLLLEFMLNSLCLASGTSQFIIIDTTSGWLATVFLNLYVIVQIFIL
SWHANEISVEGLAVSDAIAASQWQKQSKEVQKLLIIMMMRAQKPIGLTAGPFFRMTNSTA
VQTMKVAYSYASIMTQNMPE
>DponOr7FIX
MANLKQALQIYDICSFLEGEHIRLGIGGFYPRRIKRTFIVNLVTVFAYIITIAQMAVVINFVLS
ITDIVTITEVLLFSMTQVGFVNKLVNFHRNSRKVATLDELISQEIFTRVTVAEMDIMKTSFQR
CQKVLNIFLLSCFGVTLLYGVVPAVNGIMTGTKMYPFPGKFPFNPDDYFVLIYGGEVATVA
VSAWNNGAMDCLFTKHTVIATTLFRILRKKIKDLHYNTNEGERPLENRIKHCVRYYNEIIK
YVSAIENIFAYGILVQFMCSAIVICLTGFQLLVVASESGQSGLLVVYLFCMMFQLVLYCWYG
HMLMEESNRITEACYAINWHEMKIGQQKMLITIMERAKKPIALKALGIFRLNLSTLMTILR
SSYSYFAVLQQIYRNDKIMALVTN
>DponOr19
MYPIRKDLPFYASLRMLESIGFYSENTNGFKKRSIVRTLIFCILCWSIILLSAVLLIYENLNDK
NYASVFFNIAVAVASTSTYCCTLLFVKYQEKWSDILTALVNYEKFGKPRRYNQLKERGDRV
AMACWGGILTGVFLYMLFAILHENDCELEKTGGGVCGLIIPTWLPAPYDNSLLARRLVLLY
DIPNAAAVSSFVLVTHLNIQVNEFNIARIDHLSLLFNDIEFCKDPQAQLNKMKHCIEYHQDI
IRVSLQFKNLSKRTMGHMTLTFTIVTASMGCQLLQTSKNFYQENAFFFEIIYVINMFIMCYC
GQRLEYKMKTVGDFLYSTHWYNLNPKLQSLIPLVILNSQKTIRMDAVPIGYLNYELFVTLL
KTTFSYFSVLTQLT
>DponOr48
MMTLHVRYPLNRNKYFFETYILSLSSTLTGAIYCTISQVYFIVLTAFVISQLKVIKRLARDFH
LSTESNFNEEEALTAIRNIYNKHLYVIRFVHEVNLDIKYLILADCMVNSIIIAFLIVQVLFVTT
SLSSLMFYVAVSCTVLSQIFIISWFANEIEVMSTSISDALFESHWWEQTEKVKRVISIIMMRS
RKPLRIMIGPFYPLTIQTALNSLRAAYSYVTLIFAISTRNTMHV
>DponOr43
KFFLKLSGLWPFKISDNVLVDKIYRFYTLCQICYYLCVILGLSINLVILIIRFDEPQRIIRDINL
FIIAFEICLKVVIFQFRNVPHMLYQITGYEDTIEGSSDAEVKAYYAKDAIYCRRINVFQFIATF

LACASFAQDSVVIFLTSDDMSVFKETPFMHDLWYPFNRADYIYLVICIAFICDTQGLICNTA
SQTTLLCVMIYARTRLKILQIRLRKFDKIAVEEYEGDVVRAVKDLIAEHQYLINFVKSLNDR
TQHVLLLEFMLNSLCLASGTSQFIIIDTTSGWLATVFLNLYVIVQIFILSWHANEISVEGLAV
SDAIAASQWQKQSKEVQKLLIIMMMRAQKPIGLTAGPFFRMTNSTAVQTMKVAYSYASIM
TQNMPE
>DponOr40
MYSKQESLLGMLKPMMMFTGTWRLDGMNSTVRWFYWLYSLIFHGFGVLFIISVVAKFVE
FVKSGADSEDIISSQVFLLSGTCIFSKFLIYQICNVSDILKAILQEEEKIWLSKDSESISAYQA
DIKHVRKWNWGILLSTMFTGVALMSAGVASLIQADISSINSEGNEKEEWSMIPMWLPYNE
REHRSTVVVLKCIFTFIYVCMFIVSGMTFVALMIYSLGLLKMEQVKIGKCNWTSYNMADL
SVDMKTLLINNRRVFRFIKHLDRSIRYVVLVDVLLNSISIAALATNITNVQRGDFVFCTGFL
LMQVTQVFVLGWFANDIIMLSRTRADVLYNLNWYYLDLKNRKLFGMMLMQCQQLSVISI
GPFGPMTIGSVISVIKAAYSYMMLMQSYK
>DponOr6
MIKQPPYEVYATDFFSVNRWILKCAGLWPPSTPNRVVRRLYQLYTIGVFLFVNLWFTGTEF
VSLFYTYKSQYELIKNVNFFLTHFMGAVKVILWYFYGHLLRDIMNALESPQLHYEGYADF
SPHRISHLHRAIGRRYSLLFLCLAHATLISSYIPPLIAVAEYLNQPQGGLQKLPSRLPYFCWM
PFSYDTPGKYLLAVAYQAGPMFSYAYSVVGMDALFMNILNCIAENMVLIQGAFKTVRERS
TLHYCVGALAPPCDAIREHPLVLRQMDLETKKIIKHLQITLRACKHLEGIYHIITLSQVTATL
FILCTSLYLISTASPFSKQFFAELVYMMAMLFELFLYCWFGNEVTLKYEQLPMHIWESQWL
ATDDCFKKQMIFTMLRTNRPVYFTAGKLARLTLPTFMSILKTSYSIFALIKNFSK
>DponOr33FIX
MKNDFFGFCIPLARFIYIMPDKTPQNVYGWRNKLWAVFMYGLAVFCHLTEIIKLFQIVTAK
YFLLGEFIRNFVITSLHFTSLGKAMFIGGKTGKKAFEKILDFEKHVYKNLGDDIRLIYKNK
VTSIQKVKKYYLIGIILVVIFYVAAPIFREPIHIQDGNQTIRFRQVPLSSWSPFEQYYWLTFIW
TGLTGIYLSIFFVTTDLICYSYVQMINEGMKNLMVLDFLPGSVQLAGMIYQMMTNLSVIQ
CILLGQFICSLIARVFIYSNSANNLSQLSKQLAVDWFEIDWTELPKDVTNNLNFCIMRSQKN
LQITVGDLSVITMESFLTILKGTYSYLMLLMTI
>DponOr39
MSDSKSKKVFAVMRIILMCAGFWNQSISKNRLINKIFHGYSVFVKLSCLVFWLLILAETSRL
IICQYEITIITASVAILLTDTKIVVKIVIFLKHNILDIVADVIENKDEIRTEHYKEIKMLYDRKA
NFLKFAISVLGGSTTGAVFLLQGSGGALVLQDRKHNKLFNDTVETHGMYQTIFPLHRNNH
IYWLFATEVFWSYLGITANIVTQLICVIILLHAASRLEVLQVRFKHLIMPDFQIKASDEDMK
AKVTELKSIIRKYQLTIRFINEFNQSTKYITMIEFCLSTFDMASCCASLTKMKGYESVWLLF
FMMVLLTQLYLIGWTANEIRVQSEAIATALYESNWYELNKEGRQLILISMIRAQRPLNINIG
PMGPMTTRSILTVLKGAYSYINIMR
>DponOr3
MMENKSYRIMQLHTNLLKCLFLWPVDTFSPGLNSLLMYGSFCISMCCGIPIISAAGYQFYV
GIEDVNILLEALIGVYDIIGNTVTYVCFLRKQRQIQEIIDDIKDFFQYCGYETVRKIDSEIMC
HTKYFLFYVTVSVILNLAWPMLSVNNCLKSRRSDFYIKHDPCGMPTQNLYPFEASEGIVF
WVLYIIEAIFCYHTCCFFSLATVIVIGFLKHITAQLKCCAYKFEHICDYMGSCKNDENVIIRE
FVHLIKYHQRIIKYAEKVFGIFDVMIIVYIGVTSFTLAIIGYQIAIPKTNLEDRIRYTMLLIGW
VLLFYSICFYGQQVRDESMKVGEAIYKSEWYQHQTLLGMKTDIMFVMRRTQKPLDFKAT
LLGEVSLIVFVAVMKRAYQLFTLLLTVTEDGP

>DponOr5
MDTLGPALTKVRLRLPSLDIPDSKGTDYPKNLYSAIDRISFLCGQAKLSKNRSWILRFAYSL
YSYTLIIIAIMFIISEIITFRKSLTELTTVLSEIGMMFTHLVGMVKFWILIHKRDEIEQVKNKLR
DVQFEYVGIDDFQPGLKMRKEKLFIIIISTFIFALYNFVGISAHISAASMMYKYTANGNFLG
NTTCETFVPYYFYYPFDVSSPSSCHYLLFYMDLSLDIYASYIATFDSVFVILLNLLATQLNIL
GDALRTIRKRCVKRLQMKVDSSSLYDADNPLLENEMYNELTHCTKHLYLLLEVGNDIESIF
TFLTLLQTIASLLIFASCLFVAARVKPTTPIFYSQLEYFSAVLSQLTVYCWFGNEITLASSAIP
YSIYSSDWFSSSERFKKSMLLTMARLQRPLYVSIGKFTPLALTTLLSVIKGSFSYFTLFQSAG
TD
>DponOr36
MKPIEDQSLFRACKILVLCGGMWRGNIPNWPIAHQKLYKVFLRGAQFAYFFCLPSLVLSLW
VNVDQDNEKAISVLKNITFVVVIFCKMIIIQSRPVTMLIEAASEKEQQAILSEDPQISEIHRR
HVVYTEFVVKSIMLCTFLAGLAYVVGDLYLANEFYKLHPNAAPTDPKPHSIYFWFPFNPD
EYYKIALTYEFVHIVQTVIYNGASHAVVNSAIIFVKVELKILEYEIRHMMSKPNLSNLTPAQ
LMKIHIRKHQELIKWVCKFNDSFKYIILLEYSVVSLTLASTLTEILQGIKMVFNGIFFLLSTLS
LFILSWNANEIIVTSVFDLSDALYHFPWYELDKEAQELVLFMMLRCKRSLNISNGPFGFLTL
RGAVSRLKLAYSVVSVLSR
>DponOr37
MFSASKWVIMSSGSWGLEVDSKYRILYKIYVLYIRFIYITSTVAVFAMFLVNLGSNNDKAIE
ALSLTLCSVSCIIRLAVCLKQKVVNLLKIVMEDQFNYAVNDPKIKVMLQEYKSYVTFLCVF
VVCYTYSLVILFNIFNGIIEFQSFRKLHPNATEYPQYLVSIWLPFNVQTHFTLALICQTVLFL
QSCVLNYSSTVLFNTLMIYVVIKLKILQHLFQNFNTYPKNLENFHMELRDVLAIDNLKHLI
RQHQDIISFVKELDKNIKIGVLIEYTITSLMLATISIQVLTGNKVASFSFYGLILIYQLFLLSW
NAAEIKTQSEKIAGAIYATDWYVYGPGVKQIIHFIIMRCSKGLSLDIGPFGPNDLGAASARL
KLAYSYVSVMGNNK
>DponOr27
LLLCVIQPYNCLKMAIRTSLFQFAKYFMTFCGLWKVPFSPKVQRFYVVFSNVSHFVYCSF
VLSLFVKALLIVVGFESSDNVFNAISVAVIMFDINFKAMIYVKFGLPRLFHQLMKEEQSIEE
NSHQEISEYYLRQCELYVNICTLQLVTTIFPTYFYILVNILQFPLDAENFMYEMWIPFPVQW
KVLAIFKIVICQYGIFMNTAVRSALQSLMMFITSQLWILQVNIRNVFSEFSEEAAREELGKLI
RKHQFLIGFVEKVNDSVKYILLLEYLLDSINMAAAMLQIATASSVTEMTFTLVYFILLLTQL
VILAWSANEINTQSVEVSNAIYQSNWMDQ
>DponOr12
MEKDKFKILSLHINVLIFLQFWPNPLFNNVVNNHIMSIVCFITVTSCIPCIWTIYKVFEGMY
DIGILFESFICFVNIMAYLTAYWTIFRNKAVIENLINDICIFLPYCPTNLIRDTDASSIRYTKYLI
VYVTLGVFVNLAWPAISPEGCMRQRQSEYLLKHDPCGMPTHNYYPFDASKPIPFWIAFAC
EALLTCNICILFSMVTAIILGLLMQITEQIKHCCDKFEHINFKGDVETARKEFLECVRYHRAI
LEYAERVFTVFAPVMSAYLVVTSFATALIGYQIVETDNTQDRFRYAMLLLAWGCLFFMICL
YAQILQDESVLIADALYNSDWTCNSIYFRHYIIRVIARAHKPLYFNISFLGKISLTRFVSVMK
TAYTVFTVLVTVVDRK
>DponOr32
MNSKVNQAEKLDPVDKRSKVFPSSDFLRLYAIFTGQMGIFPWQLMFERNKTYQNLYNLYS
KLILSYYMYVTVSMWLALVFLCLEDTLRIPEITKNITVSVICTVTIIRLFVMKLHPAFLRNIT
FIIDAEQYILSSNDAEVHRIYKNCKIISNRHTIFFIVLSYLMALFISLRPFFTDAYEINYKNESL

QITSLPLSIWVPLNEQEHFLSVYFWNVLNNLVMTSIVLSIDIITFLLLIYPVGQLQILHHILSK
FENYKNRMKLNYPGLDDDTIGAITLKACIDLHRNIIAYVDDLNACMNIFMVVDFAQSSLL
LTSVFAQLLWVEPSITFYGFVFMYVTYLNQRLFMNYYYSNEVWLLSENLNLSVWKSNWY
EQSHYVKFMIYFFIMRTRKSLKFKIGPFGFMNLSTYIAILKASYSYIALLHSTQK
>DponOr42
MAFYGFLFNCASQTPLQTLMVFSAAQLEILQLKLSRSFEETVSSEEAKLEHVKKLIREHQF
LIQFVTNLNEAIKYTILLEFAVESVHGAGALLQLISTKKAIEIPYALMYLSLLVINMSALAWS
ANEVTTQSENVTVGVYGSNWTAQSAPLKKLLLMVLMRAQKPLSIDVGPFRPMNNEAAL
MTAKGSYTYAQFMIHSRSISK
>DponOr23Fix
MAIYPKCRLIQISMISSSLVGTFPWQFMFQDNKILKNMYAMYSKLMLGYFTLFVFSQQLEL
LILITDEEVMRNAIFANISVTPIYTITLAKQLIMMLNSSFRATIKQIIDTEKCKSPIEDDEVFEI
ELRIVQRSNKLVKYYGLMMFVLGTLFCVKPILMTPNIVSSGNTTKAIGFFPLSSWFPFDEQ
KHYPYAYMWQTLSLLQGTMYVTTTDILMFNLIVFTAVQLRKLKHLLKNFVHYKERFMTL
YNIVDDEQAAKITLIYFIRRHKEIIEYVRLFNESMEIVMVFDFLQSSLHIASVLPEVLMSEIS
LMVVLTVASFLGSMLFRLSLYYYHANNVIILSAELSYSIYESNWFDQTPKVKQMILIFMLR
TQEPLTLRIGGFAVMSIESLIAILKATYSYVMLMI

>ItypOr14Fix
MEYSFHYHDGFRSHMFLYVMLQGKIAVIILIDYVCSINHMKNCSETTMKHLKIIIKKHLEII
RYNQFVLKSNQYFYLLYLFNEVFFGLLPMFEYFSPNRSIIAVSSATVYFFCLVYAVNDAAEE
YITLVEYFRTEIFNLKWYEWDVSCRKVYSILITYLNEPMKVDFVVVNINRALFCQILKLCIQ
YLISFIQPTELVDSSFCNSALYVLSLIISHYW
>ItypOr28
MGLYPASRYFKNPIMWSSILGAFPWQMIFQENAKLQQVYRWYSNFMLTWYFGMVTTEYI
QLYHILNANVIQMDEVCENVCMSLVFTCTGLRVWVMRRTNGLSEIIQTVVDAEREADGL
DDEKTRQYEDIHVKHMEKVSFIYAAFVFMSVTNGCLATLYADTKSVIIGNSTIVEKPLIIST
WFPFDKNEHYWVAYGLQVFDGYMAALTVACTDILMFNMISYPIGQLTKLQHLVRNMAVY
KTHFEAFPTFTKIVQRHKHIIKYVELFNQSMGTFAIFEFVQSSVQIASVLVQTSPDDLTLMSF
CFIVLFFTSMLTRLFMYYYSANEVIIQSINLGDSVWESSWYHQPHQLKQAMLMVLVRAQK
PVSYKIGGFGIMSMQSIVAILKATYTYISVILRN
>ItypOr32
RVKQLRLVSILCGLIGILPYKFISPDKKLYQDIYRAWSILSNLIFVLGMFLAYMKLYTLFNEE
KIRFVELSRNLAVTMLCTMTMARQIIIRVKPEVSNMLSQILETEEAILIKNDKTVAEIYTKN
ANNLYRKSVYFWFIMFNVVVSHIARPFYIFEEIQQGNVTVIHKTLTLSLWFPVDDQEYFWE
CYTISSLYAVCFASFQSYTDIFMYAMITYPVGQLQILFHTIKHFQTYKMELQRLLDSMNDSI
AAKLMMKNCVEMHRLIIKYIEDYNSCMSILTVFDFFQTSIQITSILSQIVMIEITVFLAVFIMI
FLTMVFYRLLMLYYYANEIIITSEDLCQAVWQSEWYNEPPHCKFMMQIMMMRSKTPLML
KIGPFGSMSLRAFLSILQASYSYFMLVYSQKDN
>ItypOr31
MHIFPKSDHLDFSFALCSMLGILPWKLVFQDNSFLQTLYYLYSKTLLIITVIFITTEWMEVCR
ILNXDPVNLTDLNNAIAPVLLFTVTAIRMIIFNRNPDFMKLLNYIINRQEFMAQRDDEIRKP
SQKFIKINKWTGVGYLIMYLAVIYQLLALPLVLGPIEQQTANQTTTIRILPLLSWIPFDTQQH
YWGCYLWQALNLQLASCNICHIDVLMFALILYPIEELGYIKHVLRNFDSFKSRTGIENSNFA

SITVFKDVIKIHNNVINYVGTVNDTLSFVMLLDFLQSSLHIAVILGAVVVGGPTDLASLSFV
GTHFFSMVLRPFLYYYYANQVMVLGGDLTKEVWNVDWFDESKDVKYMVRFFNMRAQK
PLQYFVGSFEVMNLQSFISILRVAYSYVMLLHTLQ
>ItypOr23
MAVYPKSEYLKVPAIYCSTIGIFPWKFMFQDNKNLQTIYRCYSIVMLAWCIGFVVTDYIQL
VILLTSKTLDMQEISFNTCITLLFTCIGLRAVIVYFSPNSANLIQSIIDSEKVTYLDDAECMKL
EKKHLRSVRLISHCYFIFIIFSTTSRCVYSFPKEPDIIQNGNETEIVKEHMLSIWFPFNQEKYY
LTVYNIELLDSFLGTFFVAYVDIYTFNMISYPKGQLKKLQHIMKHFHNYKAKYSSETNEEN
DFIVFKDLVQRHKQIIQHINAFNELMEFVAIFEFVQSSAQIACGLTQSSLENLTIGSFLFVMSF
LISMLVRLFLYYYAANDVTVESTKLAQCIWESNWYEESQKIKLSMLMVIIRAQKPLIFKIG
GFGAMSVQSIVTILKATYSYITLAYKRT
>ItypOr16
MYNIPENERKNYFLKFSRVTMLMLGIWPVRRGGDLLEKLYESYFLTTFLYYIAFNLSGLAL
AIRTWSNNYLTTASSMGIVIEYMSNAYKVWLFKTSVFKSLIKEIQDREREIFEGPDEAFKEI
YIRNAESNKKVVLFYTIMGTSGISLYFITPLVSNVLMPLGYNNVTGVYEHYFIVFNWFPFD
PNRYYWAAYLIQFTGCLIGYSYIVHCGAFYISILNFIRTQLKILRHVIVNMSEYSLLYKNTYK
LTEEQSQFVLLRAVVLEHQRIISFVTKTNHTIQLFTLINFVISSFQLALLVYQIFQVAILQQVT
VLSYFITLSTQLFLTYYAAHMILFESSNIASSIFEGNWDTYPPQTLKLLQMICMRAQKPLAM
TIGPMAAVKVTALFQIFKALYSYICLIIKF
>ItypOr34
MKFRELIHNDFLGICIQLGYYFCIIPEKAVTTDKIESRNYFFYTCIVRTLILYCHICQWVKMY
QIITADIFIFDELVRNCAITSIHFQSFVKTSIFRQNYQLFENVIDFENVLYKNNDQKVLLIYRD
TLQAIKNSRLVYVFGILIVIVFYIAAPLFRGPYYVEMGNETVTIIQLPLSAWSPTNNYFSNFA
VTGAMGAYLAMVFVQTDLLYYCFLYFSICQLNILEHYIVHFHHYSNELVNDHKCSHVMA
LSLTQKIYIKYHQNIIKNVKQLNDALKNSLLIDLVPSSIQFANQFYIIATNLNIMQCVCIGFFT
IMLMSRVMAYCYLANQISVQSQKIGSAWFQMDWSDFPNEMKKMISFCIMRAQKPLVITLG
NFGNITLMTFVGILQASYSYVMLFITL
>ItypOr2Fix
MKVLQRETEITFFKFNIWVLKTCLLWPEDLNYKYDKKRFIKDLTMVASLMPCFLPILADFL
QQLYEEVPDLTEAVENMIALNCLIGMFYMVICFVRNRRMIIQLMIDIRTFNKYGNDSITQEV
DNKANLFSKMFMFYGILGNFVYMAMPQIRVSKCHLNRTEDMIEKGVPCGLVVRSYFPFKF
DYSPVFEIVFVHQIYTCTMVSVVVLVLTMLFCGFLMHIVNQLKHLRVLIARLKNVPPEKFE
RKLIFVVRYHVAIIQYSQNTAGAFSTMLLFYITLTSVVLSVLCFEILMVDAFADSVRFTLHL
LGWLIILLSICYNAQLVLDQSQEVANDVYSLDWVSILSVDVQKKSKXVIMRSQKALVMEA
GGMGVVSLSAFLKVLSSAYSFFTLLLKFK
>ItypOr8
MSELFLLHFPRILMVICGVWRLPYFKSKKVQTVYDIFSIFLQFTFSLMCLSMFFELVNLINT
WNVLNLIEFSRVALSSYLCIIKALVLRNSSIQRIMVYMIKEERNVLRSKHQQPKALYMDTV
KLINRVSFLLLLVVLPTLLAFSADCLRKGIIDFDDAVKYVYLPLIDQKKYKTVQLTVQTIFIN
LIGFYYCMTQAFMVTAMKFAQGQLELLQLYFREFDYYAARQSTTEIAYLKTLLEYHQKIIH
FVETLNKEMRLVIIIEFFFSAVNIACSLFTLLTMATNLIDILFSVNCLVFLLAQLAILSYLGNEI
YQAGLNIASASYELKWYEKNREFQKNLLLVIKRSQKPLVLSVGPLGPLTNETFVSVLKASY
SYFNLMTRYN
>ItypOr17

MGLMTTPRFFLQLWGIWPVNTLLPPKJYVMYRFCIIGWYSFFNIFQFIASIRLILNNESFERI
SRCISVMVTLVLMLVNSLIYQKNCIPQLCSTVMEIEQXLARSNDAKITQTYHTTVAKNKYL
NLYIVGSSLFTLVAFIGLSLLDVIKAGPAFWDFDNVTFMHELYVPFNRGNHQALIITTNIFTA
CESVVVNGVIQTTFYALVMYGALRFKILQLNLKKIEQTEGDRKWRMRELIQDHQYCIRFV
GELNQATKNVLLMSFVLNSLKVASVLFPLMAIREFTDLAFPLIYSSMLVSEVVFQGWMCN
EITEQSLQVAQTIYDTFWYKESKQYNVLLQLMLMRAQRPVTMRIGPFGAMTTSTILTTMR
AAYSYATLMMNSS
>ItypOr18
MYTLSKNKPFYYALVLLRAFFWYPSSPKCSVTFILCSLVLRLLSTLAALGTLAHLVLNLTG
ETKAEISEDIGDLTGFVGCMSACLNFLWHRSRWSSFINRLLTFKQFGTPPGYVKVVRRGNL
ITLACILYTIPGMLWYSHLTHLDIPRCEALNREFGMKEACGMVNPTWIPPGYDRRNGWRF
WVLYVLQCTGIFVYLPSTFVISNIPLEAVGVIVTRIBHLKYHLKRCGSDLGRLYHCVKYHQ
DIIEVSKELSDLVQATLGTLLLTGAVVIGSLGSQVIKASTPKAVTFILGYVTTIFMVCHAGQK
LINQSLTLADQVYWMEWYEQQPKIRKDLRFVLARCQKPLGLVGPPSMGXAGYSLFLIML
KTSYSYLTLLNEVIS
>ItypOr4Fix
MDKQSQILKFHVKVLKFLMIWPFDGLDPNQNYYLMRGCFAYACFCSIPVFSGAAFQFCVG
IDNVKVLLEVLVGVGNITGYNIAYVCFLKNQEKIQFLIRDFQEFVQFSGPEIIQNTEEKTTRY
TKYLLGYASIGLVITFSWQMLSTESCVAQRGGDYYVRHDPCWLPVRNWYPFDASQPKLF
WIVFPIEAIYSIHICLFFSLATSTIIGFLMQITSQLQYCSNRFEHVFDEVDLKPFQQIKPDFLFL
IKYHKKILDYSKKLFNVFDALIVVYISLTSFIMAIICYQIVDPKISAQDRIKYAILLIAWCLLV
YLICYYGQKVQDEALKIGQSIFKSHWYGGTTAVELKPYILFTLARTQIPLEFKAQLFGTISL
LQFMKVMKWSYSGLTLLLAVTDED
>ItypOr13
MSNYQRYNIQTFLKEERLFLGITGFVSGNLKLHLPTGVTYVLTTMQVLASIYYGLSTTDLA
EITAAFMITLSHINTLNKLFGLHLKSLTQLDRILVKQIFALADDRELTILKRTLTACHNMLTM
YLVTVLGSILLYGATPLVANYTTMERNYPTLAKFPFNPDDYYWAVFAGEFFIVALSALSNG
CMDRLFAKHVAIATGLLKILRHKIKQIMDVDDQKVIEAKMKHCVLYYNEVMGYANQIEN
QFSFGIFIQFLCSCIVICLIEFQVLLATSSETIGLLLTYLTCMITQVTIYCWYGHQLMEESNSIS
MEFYDLNWIEMSVKNRKTMLTSKERAKYPIVLKASGVFPLNLATLMKILRTSYSYFAVLH
QVYTK
>ItypOr9
MFSSKNLYKYYSHFASTSIIMYTIMLTIRLVQLVIEGQTPSAKLYRCFTINIVIYMMTANLIIF
RRYGLPDLISQVMKDEEAALNSLDKDIRKTYLAQTKIYEFTSVAQVVSTFASGLMFIALNV
YMKVKGLLKHEAFMYELWFPFNRENHDGFVIFFNLYIVVLIMFCNVASRIIPQTMIIYANA
QLRVLQILLEKAFDAPCSDPLVKIQELVKKHQDLINFITFLNSALRNVIFMEYIINAINVAAG
LLQFITVRAAMDLVYAFVHFSLLVIQIFVLALNANNVSTQSEAIANAAYNSQWMDQSNNI
KKIIYIMIMRAQKPLVLNIGAFGVMNAESALTTMKAAYTYVSIGLQR
>ItypOr19
MLIFGNIISANVIVLFGSSLINGDYFLVTSSFPFALAIIVVNGSTLSFAVNHKQWSNLFKSLTD
CQKFGKPPNYDLLKKNGDRKGMICMTCYISTCTLFAIIEAVEEQRCLKNVSKHEICGFIIPV
WWPTNYHPSTFLKTMVQVYEVGTIIILSNFTIIATLQYQVCEYIAAKAAHLGLNFNAIDPN
SDAKTQFEQFKLFVDYHQHIISLCAEFDSSCKRTVGHVTFTTAAISALFSYHGMQGNYKLL
AFLALYILNLGFMCHTGQNLEDAMLGISNSIYSSKWYELNIRVRQRIPFLLARTQKRIGLDA

VPIGYLNYALFMTVLKTTCTYLNLLNHTI
>ItypOr27
MLNGDVLKMDAICSNICLTLAFTCSALRATVMRVGPNLLKIIEQVMHAEKNPASIEDQTSF
NLERKSIKTMRKLSHLYAVAITMIASSKCALAPFEKGEIVHIGNTTIIDRPLIMSAWVPFNKN
THYWAAYIIQIYFAALGAWHVAYVDMFMFNMLGYPIGQLKKLHYYIKNITTLTRNDDSLE
EFKNVIRQHQQIISYVKFYNDSMGTFAIFEFLQSSVQIASIFIQTSPSDMNLGQFGFIGGFFIG
MLFRLFLYYYTANEVMTESEKVGVSVWESDWYEQPTNLKMALLTVMMRGQRPLYYKIG
GFGLMSVQSIVAILKATYTYLTVVVRNN
>ItypOr43
MDAALALMKNITFVAVVVFKTVVVQSDAIVKLVKAASVEEEKIRNLTDQAIRKIYKSNVD
YCNRVTKVIITYLYGSGTIYVLDGLYKSYTYYENHPNVKPEDPKPHTVLFWFPFDHNRYY
KIAIAYESFHIFQTLNYNGVAQSVVSSVMVFLKIELKVLQHHIRAIQGGSRDYQKVLIKCAI
KHQQIIQWVNDFNNNFRFIILFEYSMISLTLATILIDILQGTKICFNATFFALNFTQLFVLAWN
ANQISDESSISISDALYACSWYEFDKTTQDFVLFMTLRCKKPLNISNGPFGYINMDAALSRV
KLAYTVVSVLSTSTK
>ItypOr36
MKIPHMFEHAMKHERNIFKSRDLELIICYQEQVKYGRRVNLSQFLVTTLSTSTFAVTALLD
VYWAADMSKYEKEPFMHDLWFPFRRETHMNWVIFFNLFMIVQGTCFNTATQATLINLMI
YSSSRLKLLGLKLRKFDAIASQNGRDILETVHDLIFEHQDLLSFVESLNVRIKYVLLMEFIL
NELGLASGIIQLIVIDTTSYMVSVVTIIILQLFQIFVIAWTANEITIQGAKIADSVMASNWVE
QPTNIKKLFLIMVMRAQRPLGLTAGPFFNMNANTAVSTVKAAYTYLTFMMNNYN
>ItypOr6
RYATDFFFGKPMDVKKGWFTEALKQKQGSKYSLMFLMLAHATLTSSYVFSTITTIQHMKG
NSTVALPDRLPYYSWMPFSYDTGPKYLLAIGRIKQVPMFSYAYSIVGMDSLFMNIMNCIA
ANVTIIQGAFKTIRERALPGQPNNVPHESKADMDVLRVELRKIVNHLQTIFKACDKLENV
HRMVTLCQVTATLFILCTCLYLVSIAPPLSKQFLVEFVYMLAMSFQLYLYCWFGNEVTIKF
QELPRYIWASSWLATDTQFKKALLFTIMRTKRPVFLTAGKFSRLILPTFMSILKTSYSIFALIR
NTSK
>ItypOr35
MDNQPKFLLFTKRLAFALGIFPSKLTFQDNNWRNQCYTLYTKFCLGLFNLYLLTSFVQLFV
IMSSNPIDFTELSKNLIITPLFTVTVIRQICMSQPGFIKLIQHLISHEMYLDTXTDKYVAYIIQS
SNIVFCSTFDTVGEILLTTILVYPTVRLKILKHVFENFQKYERNFTDPHSADRLMKVCLKIH
TDIIRYVEQFNSVMGTCMFLDFIQSSVHMACVLAEILTGDVSLIELVSTSAYLVILNFRLFIL
FTTTANEVIVLSQGIGVAILNTNWYKKNSSV
>ItypOr1
MWITMFCTKNRHVLISLVQGLSDFTGFPPNFDKFMQQLNFYSKIHLCYLTGGSLMYFVLF
APLHKRNCDELKREKNLTETCSLLLPLNAPFVEYQSFGKFPTLQLLNIIIFLSLMYMYMCA
GTIVWLNVELVEHIRIRIRHLKHMILRALKSNDKQFRREKFRKAVRYHEYICSMSRLADEF
FGTELFLHVVLTGAILGISAYLIGDGSLETVMIFVGWLNAIIMGSVAGQRLINESLGISDIIYE
VDWYNFETALKKDILFFLVYAARNLCLLGLGMW
>ItypOr11
MDVIKDILRNFKENSLLQAKFERHLKVAIKTKKRGDFLFLYGTFLGFGTQLFWSIYPFSQN
KKLLPVNGWYPYNPMNSPSYELTSFFQIFASAFNISHTMNIDSFTINLMMQTGMQCDFLQL
TLKNIMQFHTVDGILCQSLQQNTEPLEDILTPNLKTCIRHYIEIKRIARKLEDIYRTSMAVVF

LGGAFIFCAIFYQMLHSQRDPTEIFYLLFFLFSMLTEQFIFCWFGNEITFKSGQIHGALYTIP
WVDCSVKFRKML
>ItypOr7
VFFDQLRVCYFNVWGGTLNGQRLQWKEKLSFSRKFPFNPDDYYVAIFLGEVIAVAVSAWN
NGSMDCLFAKHAVIATTLCKILRKRISTMLQITEDDRTIEDKLKHCVIYYDEIISYVTKIQRI
YSYGVLIQFLCSAIVICLTGFQLLVVSSKNGKIGLLLVYLTCMTIQLVLYCWYGHILTEESNG
ITMACYVVDWHELKVTNQKMLIMLMERAKKPLGLQAMGVFRLNLFTLMKILRSSYSYF
AVLQQIYKN
>ItypOr3
MPTRNLYPFDASQGWSFWILFVIEAIFCYHTCCVFTLATVTLIGFLKHILAQLRYCGHEFET
IFDGVNEESGGKHLTLQHFIRVVKYHQEILRYTEKVFSTFNVMIVVYTGVTSFILAITGFQIT
SPETGGEDKIRYTMLIIGWALLFYWICYYGQQIQDEASQIADAIYNSKWYENTNTVVLVRR
DIIIIYLRTKRVLDFKVQFLGAVNMEVFVAVMRRAYQIFTLLLSVT
>ItypOr39
MHDLWFPFRRETHMNWVIFSNLFMIVQGTCFNTATQATLINLMIYSSSRLKLLGLKLRKFD
AIASQNGRXILETVHDLIFEHQDLLRFVESLNVRIKYVLLMEFILNELGLASGIIQLIVIDTTS
YMVSVVTIIILQLFQIFVIAWTANEITIQGSKIADSVMASNWVEQPTNIKKLFLIMVMRAQR
PLGLTAGPFFNMNANTAVSTVKAAYTYLTFMMNNYN
>ItypOr12
PFNPDDYYWPIFFGEYFATGLSALCNGCVDRLFAKHVSIATGLLKILRNRIKQIMDDDDHN
VIEAKIKHCVLYYNEVIKYGKVIERQFSFGILFQFLVSCLVICLTEFQFLVATSSGSFGLLFAY
LACMITQISIYCWYGHQLMEESDSVSMEFYNLNWLDMSIKNQKTMLTSKERAKNSITLKA
SGVFQLNLTTLMTILRASYSCFAVLHQIYTK

>McarOr1
MLKFKVVGLVADLMPNIRLIQASGHFMFNYHADNSGALHTLRLGYSCMNLVFVLLQYGA
IFGNLVAEKDDVNDLAANTITVLFFTHCVTKFVYFAVRSKLFYRTLGIWNQANSHPLFVES
NNRYHALALKKMRILLICVGMTTILSAAAWTGITFVGESVHTIKDPNNENETITEEIPRLLI
KSWYPWDAMSGMAYYASLVFQVYYYVFFSLSQSNLLDSLFCSWLIFACEQLQHLKEIMKPL
MELSASLDTYVPKSADLFRAPSATSQDNLIENEYNAKNEELNLKGIYNTRQELGGHFRSG
TLQTFGQGGGGVGPNGLTKKQELMVRSAIKYWVERHKHVVRLVTAIGDAYGVALLLHM
LTATVMLTLLAYQATKINGVNTYAASVIGYLVYSLAQVFHFCIFGNRLIEESSSVMEAAYSC
HWYDGSEEAKTFVQIVCQQCQKAMSISGAKFFTISLDLFASVLGAVVTYFMVLVQLK
>McarOr2
MKRNMSYDNFDYTVFFTHNILMYKIFGFWRPDDDMKREKLYNCYTLICTIIWLLFLASQY
IFIITNIQNVDEVTATSFVTITFSINLIKMLAIYRNMNRIKQLIKDMNLPMFQAKCARHRDIID
YTRIYTIFFYICLYFGNTDRHYFWTIVPFIGDERATLTHGWFPYNETKSVNYEITYVFQTTV
SVWNTMLCLNLDTFTGSLLILIGLQCDLLCVTLENLGDFHVENGVLCENSEEYQSSLVND
KVKFSKTMTENLVVCIKHHKEIMRVSKDVEDIHRVSVFILFLGGALIMCCCLFQLSVVPIGS
IEFFMLLFFLISILTEQFIYCWFGNEVIQKSSRILHSAYCTPWLDCDINFQKVLLQLMTQTYR
PITLKAGGLFTISISVYISVIRTSYSYFTLLKK
>McarOr3
MSQKVDPQYFKKHLKWLTWLGIDIIPIEKVWYAIPYKLYAFVLLVYVYLYSLLEIIDIVKSS
DFNSMTFGLSYSVTHILGAAKITILILKKKILRDMLIRLEQGYFVPNKARGGEKEQQLVNA

SVIRANLHADIFNTLVYLIIGIRCLYAIFDKGVYVEVLDEKLNVTTLKHIRTLPYKAWLPVD
LNKSPAYEFMFIIQASCLVLYGYYIGFLDSLIYGMMIHMNNQYLILRNILEHYVELAKNIVL
NRNPNSVTDDTSTDYIKLHNGIERQKTLAGPVLDVIENIAYHCAKYHLAIIDYCDDIEKEFS
NLMLLQFLSSLYILCFQLFQLSLVTNYFSFDCISMCLYLILMMYQLFCYCWYGNAVMIQSL
DISSVIYNTDWLVTNESTKKCLLLMMMRAQRPIIFTAGKFAFLSLPTYMAIVRGSASYFMV
LQQMQ
>McarOr4
MAPSYVFDLPKAFEFEKKLLLYTGLYPNTGLVNKYIYYLSGLFHIGITILIEISLIIVISIHIDNL
STITDALMFFVTQIALTWKLTNVCIKRKVFCEIEEILSQPIFYNLSQECENIIHYYVKFSHRFA
RCFRIICIMVCATNGTLPLVGGKLGHAMLLGWNPWDSEDRIKYYLNSTFQLTALCVSACIN
STIDILTVILLAIATAQIEILKNNLVNIKYGEKEAKKLFNENVRLHYEILRFVNAVDRSLSSGI
LSQIFGSVLVICVTCFQLIIVSVQSIQGAFLLIYLLCMTFQVGLYCWFGHYLIDSSDTIIQAVY
MSDWYEANNSLRKAVIIFMERCKQPIVLRIGGLFPLSLGTFTSIMRSSYSYFAVLRKWYEPE
>McarOr5
MTDKGYTPHFFRTNEIIEVYTGAWMYNENLVAPGKKWLLYIWSVLIYIGAVFFLFLEFLKL
RDTMKVSNDFIRQCGLISCHSLCVVKFVILVLRHRKIKRLMDTLQDKKYQYEPLGDFSPG
QRFDEARKLTHWCTIGVFCLYSCAAVSAHISAEVLINKDAKRERFDGNITCYEYMTFYFAI
PFPSDTKAQCEMSFIFMHFCIDIYAWFAAGHDSFYAALLNCLRVQVDILCDAFRTIRPRVLK
RLELPQDLSIFHDDDFPKLEEALYRELTHLTEHLMILLRVADDLEEVFNLITLAQTVSSLIVF
ASCLFITSTIPLSSPEFFAQVEYFTCMLIELSLFCWFGSAATRASEAISPAIYESDWYGTSKRF
KQSVLIIMCRMQNPIYLSIGKFCPLKLDTIVMVFKCSFSYYTVFKAVGE
>McarOr6
MPLSVFFIYFLIILSSMQPFAAIAYQFYVGIEDMNIISEAFIGISDLVGFLFIYICFRKHRGLIKE
TIKASAVFLKYCSPNVMEKAEEEVQTYTKGLLIYFSIGLTFNGLIPLYDYENCDQRRLSDY
YRAHDPCGMPIRIWVPFDARKPVIYYLVFFLHANACLNICYGVLCITMTLVGLLIHITAQIK
NLRQNLLQVFDELPEDGDCYSETLVLKLENKLKFCVKYHIIIINYTDQVFAAFNLMLIVHIS
LTSLIFGVLGYQIVTVEDFTEKLRYVMHLGGWIALLFLTCYYGQLILDESTTVANAAYQSK
WYNGPTYLRKNLCLIIMRSQKPLKLRAASIGVISLETFLSVIKTAYSYFALLLSIAE
>McarOr7PAR
VLLYCTTEFAFLCKLMNFVLSKKEIIELEAILESRLFTVDTPEEEAIIKTSTRQIRKLANIYKT
LCFLSVTFYALFPLADGGREAQKLPLPGWFPFNVCNHYYEVFIFEAIGIGLCAWFNSALDL
LVVIMMILGKAQFELLRHRLMNIAIYGEDGERRRRVKMCAQHYKSILRFVMLTESIYSNGI
FVQFMSSGIVICFTGFQMLIISLKSIQFVQRILYLSCMMYQIVMYCWYGQVLTDSSNKITEA
CYLADWINCNVILRKSLLIIMERAKYPAKIRAANIFTVNLETLLTILRSSYSYFALIYSIYDTK
NETK
>McarOr8
MTVPYADDFFHTNRWILYIGGLWWPDTYKSIYHKILYMSYCAANFLFCNLYFTPTEVLSLA
STYKSIYHLIKNFSLSQMHVLGFTKVLFFVFKGYKMKAIISVLEDKKLHYEDCDEVNFHP
GMLTNKYKKIGRVAGIIYLVLPLVVILLAYTLSAIAALRYVEGDSNHQLPERLPFYSWMPFS
YDTPKKHLIALVYQATPLVSYSFSVIGMDFLFANIMNCIAMNFTIIQQAFRTIRERAAIRVKE
PLKVKDELYNSEPLQRELNKEMRKIIQHLQTVYRMCDELEDVHKYLTLAQTLSQLFILCAS
FYLTSITPFGNQLVIEGIFMIMVISPIVFYCWFGDEVTHQGGEISVAIWQSDWLGATKSFKTC
MIINMIRTQKPVYLTTGKFAPLTLATLVSIFKASYSFFTVLKNTSNQ
>McarOr9

MTILGIWPVKANWFKKLRLYEVYHRISFWILFEFYYNADNKNFVQVMGKNFSETAEVLG
VVIVLLITSFKVKICTSPKIKNLLQQIEDAEKIIIETTELDTRNIYNQHIKMSTKENTVQLMIG
VLAISLYSVRPILANRGLEPESKNKMFIFASWFPFDEQTYYAPAYLIQFISGLYSTGYTISTTM
FLFNAMIFARCEIKILQNQFVNFTYYVKKDAKDNCRTYEESQKVALEDRILKHRKIINFVN
TLDTSFKTILLLDFTVTSFQFSMVVIQMVQRSQLDVAVVSMVMYLSTLALQLYLVYSNAH
EIIIESNKIAQAVFESEWYDLPNDVKKAFVIIMLRAQKPLYLSIGPLYQVRCDMLFKILHALY
SYICHFLEVNYIVTIV
>McarOr10
MSSYPKKLFFLNRWILCCVGMWPPDNQNKLFRFLYKVYAIAAFFYIMVLYNVLEIISLIYT
YNDTVSFMKNVSAVCVHLAGAAKSVIFYLRGDKVVEMMITLESEELRYEDCEARNFYPG
KISKSCKITVAKLTALCFVMVHIVLLSSFIPPILQILLCIIKRDATVLPDRLPYLIWIPFKMDTV
SRFTLALVFQIFGMFGGAYNISGMDSIFLGLMHCISQNLVIIQGAFLTIKERSVKRIKGPALA
ADRLNNSECLNAAMNSEMRKVSRNLQTIFNVCWDLEREYKYLMLLQVLITLLILCSSLYT
FSSATPNSKLFYTEIIYISAMMFEPFMYCWFGNEVTHKADEMSNSVYQCDWLGTDKKFKT
SLILNLTRSMKPIYLTAGNFVPLTLATFVAVVKGSYSLFTVIKGSN
>McarOr11
MYFNSLLGVWPFVFERPDFRLWQMMYKMYSNLMLVFGTYVICTQYTQLVMLLQEEEIW
VQEIIRNLCLTLLHSMGLAKVYAIRSDNLKELISEALKVEEDIYRRGDEDIMEIYRLYAWHS
RVSNIAFLINIAIETCFYAMHPLYVGELPHFDKATNQTKMIRALPMSAWVPFDIQEQYLEAY
LWQSVEGTVTASFVMYTDIFSFSLIIFPLGQISILSHVLRNFDHYVKKAQEKHGCDRDEASF
FIARECVVKHQDIIRYICVFNNAMKYIMVFDFLQSSMQLATIVIQLFGSELKMVEVIFHGEF
AFCMLMRLMVYYWYANEIMLKSSDITLAIWEGVWYEESQRVKHMMLMIIRRSNKPLAL
DIGPFSTMTLQALLGILKATYSYMTIMYNR
>McarOr12
MRANALLGVWPFIFEDNPKLQKIYDVYSRCTFIYYLLFIITAIIKLIFLICDEVFVIQEVIANL
CITLLYSVTIMRVWAIKTPRVKNIIREIIITEERILKSKDETVITIYNSHAMQSKVSNIIFLVNIF
LVTALYFIHPLYVEDRAKFYESKNITVIEKPLPLSSWFPFNEQEHYLVTYLWHVLDGSIGAS
FVTYTDIFTFSLIIFPLGQLKILIHIMSNFEKYVDKIQNQLDCSPEEASFTTLRECVLKHNEII
KYINDFNTAMRNIMVLDFLQSSIQLASIVLQLLVAEFTILNFAYSGQFALSMFIRLLVYYWY
ANEIMVHSSDVAFALCTSNWYEQPEKVKKMLVVILMRCNKFLCLEIGPFTTMTLGTFLGIL
KATYSYMMVIYK
>McarOr13PAR
NTVTSFKEDFFHANRVIYRICSLWLPGKEIPLQLRVMYLTYVFAWYFLFLTFLICEFLIFKDM
LQEVSKFVNYFGMLFTHLVGTLKLSVIILQYKRINNLMSILQDPEYCYESLGDFQPDVLLH
KSKIISFIVSVSTFVLYSFVGISAHISSHIVMNQVVKNPTLEKNMSCVDFVPYYFYTPFTGTT
KLQCESMFLLMDICYFIHATIIACHDGVFAGLLNCLRTKLVILGGAFKTIRPRCLKRLNMPT
NFTVLHEEENPEIEKVLYAELNHCIKNLHILLQSRDDIEHCFSYVTLAQSLASLFILASCLYN
SSTVPVTSPDFFSQLEYFVCILTQLSLICWFGNEITLASNHIILSLYEGDWFSASPRFKRSMIL
TMCRMQRPLYLSIGKFSPLTLATLVAVCRGSFSYFAVLQSI
>McarOr14
MDGGILRVQKLFMILSNKWEVNTKSVLINKLCRIQGIFFESYFILFTLYLPFNLLLHRKCML
IFYELGGYFLHHTNIIIMNILFRKNAMKKTLKYIKNYEQVEYSKETQDSKDIYGYYSLLNA
RLGKYVIVFGTCIAGISWYVSTISYSIKENTEDCAVLEGVMYQVWYPFKTRYNWLSIIFDL
SMAYIAVSMHIFNRMSPITLVLFQLAHIKILANKIRNIDTHAEELASLHDVNIEQALNIAVDE

CVKSHQEVMSLMDLLLQATKEMMLIGFFSSSMELASFIIQLFTAASKYHFIRCFIIFPIDLLQ
MLAFFWFADEIYVESTTLSNVIYNEVDWTRYTKPLRMKLIVMMIVAQKPIYFNATGIGEM
TLEKFKCILNSCFSAVTFFQTMYYN
>McarOr15
MEKLVHIKLLRRMMIICGQWNFKNYNNPALTIYRAYSRFIIYHVIFMTQMILLTIAMQWDC
RSRVIEMLMLLYSIHQHLVMIFLTKIYNLEKSLNYMMDYERVKLKQAGEDEKNVYFKYAR
VNNNMNVLIIIVCILTAIMWYMTSIRNTFTVRGNEVCPISKGLVYQIWYPFNFDNQYWLIVI
NDLIFFLNVVILLTYTKIISITVTIFMLGQIKILQEKIRNLEQDALVLQRINRTEYDESLLLSLK
LCIKRHQEIGWFMEVLQDSTSSIILTQYFSNTFEMAAFLIQMLTEKSLYLIIRSFIVFCMVILQ
VYIFYWFANEVQIESTAIPDIIYSETKWTENDQIRRYLLLMMTRSQKKLSFKSAAIGDMSLA
TFTKLIKLCYSIVAFFRTAYDL
>McarOr16
MSGQTQNIFLHAQRYIMIFVGKWYIDFGSNTKNRIYYIYSVLVELYFLFMTQQILVSMVIYR
GCTERVGELICYYIQYTNSNICSFLSKRSKIRKIFNYIMDNEAEHLKHGTADVYLKYAKVN
RKVIVLFLVLTGVAGAIWYILVVRDTFFAEENENCLILRGLNFQIWYPFDLFNRCYVITLLN
DILMYTSAVGVHIYNKISPVSFMIYILGQIKILQEMLRCIEKDAISMHELQGEKYEEAILKNI
NNCVKMHQEVIKFMGLIDKGCKEIVLIGFFTNSLELAAFVIKVLMEEDVFGALRTIGILCM
TVTQLFMFFWFANEIKVESTYISDVIYYQTNWISYDKHARRHLWLMMIRSQRPLTISAAAI
GDMSIDTFKRIIKLCYSIATFFKTVYM
>McarOr17
MVKPIETVCCRTTLKILRTCYMYPEEGKEQNPGKLFLLKWLTLMILSSVTFIGSFLHLIISLK
DEDYRHLDVDFSITLSMIATYIFTCFFFARVKFASKFYMHLSNLERLEKPLDFEKKNERLEK
FALYHYIYMELLVASLLLFSNVIKGAKCKQENLEFDLHEVCGLFTYTWMPFDIDYFPVKQI
YLFLQLFGTHYLYLIAGTMAWTVVEAIQQIVLRLRHAKYLFTEAIKEVDPVLQRQKFNRA
VRYHDAVLGLDDRLNGTFGVFMFTHLGMTAPILGTAFFAILHGGSGSSLFICLGWFIGVSM
DCFSGQHLQNESIDIARALYDTQWYNCSQDIKRDVLFVLMRCTKPMYLKATSFGIMDRV
MLLGVLKATYSYIALLTQTQ
>McarOr18
MDEIKEEEPFTHSLKMLNVMDAFPLEHNFFSNGIFFVRFWILRTMSFCISCVLPTVHMVTS
VKDGIKLIISEDLSVIVGTMVSLITTCIFVFKRNSWSKLLSDIADLKIYGSFSDFDIVKVKLN
LFSRIYFWYCVNATFVYGSVSFIDTSQCEEINKLGKEWREVCGTYLPMRLPFDANIQWMR
VSIFFTQMFFTLLSLAPSALACSIIFQSTGFIIAHIENLKKHLVGAFDSTDVQETSNILRYCISY
HNHILRLSARLQDLVGTNISHVLLMSAVVFAGIGNQILKTKPVGGTLYFIGYMIALFLLCHS
GQRLIDETASIGSAAYNSKWYKGNTSMIRDTLLIIYRSQKPCTLEVLSLGSLNYPLFLLIIKT
SYSYLTLLQQTA
>McarOr19
MFKIQKGAPFYSTLLALSLFGQIPVEFKETYSKSFLVKCILSRFIGFVLVSVAPILQYVMATK
GSIEVDISENISITISSIGALLTGTFLTCQYKKWLKFFEDITDHKAFGKPPDYEDLVKNFNRFS
AFYTIYCTGSVPVYAVTVYFNSMRCDEAALKAGFFCKSFTPIWLPIDGSSLQLRVIIYIVQM
LLGGTIVCTSAVINFMVWESTEMLISHINSLKIHFNKISEKSTDKERSEQLGFCVRYHNHILR
LSSRLNGLIKWTSGHMSLTAALIFASIGNQISNSKSVGAFLYLIGYVGALFFICHAGQRIKDE
LMSVGDAVYNADWYATDVKTIRSLRFIIARCQIPFHYEAIPLGVVDYPLFLMIIKTSYSYVT
LLSQTT
>McarOr20

MIKIRDIVCCTVSIKILQVCFLFPLKGKELEPNYLRGFIFFFLMGFSSLTVIGSFLHFIISIKNH
VYYHIDLDMAIMISMFTTYSFSIVFFFNIKSAVRLYMTLSDFDEHGKPRNFDKRTKLIDKVV
TYYYIYIEFLIIFMLSTSNVSSSGKCKKKNKKYGLNEVCGLFSYTWMPFEIDYYPVKQIYTI
CQLVGTHYLIILAGVVSCLMAETMEQIITRIHHARYLFLEAIKEKDYAKQRQMFNTAVRYH
IGVLDLEDPLNETYGFFMLTHLAMTAPIIGTALYSILYGGSGSSTFICLGWFIGVMKDCCCG
QRLQSQSNTVPIAIYDSEWYTCNEEIKKDILFVLMRCRRPMYPKAISFGVLDHVMFLGVV
KAAYSYIALLSQTT
>McarOr21
MSTDRICVPFSTSIAILNFNLLFTHGKSKSMLIEVMGWSLRLLSFINLCIFPIHHLITSIRDGIE
VDISEDISSSAGGFIAVILLCLFKFKEREWSKLINDIVHFSKIQKKSEFEKLTKKMNKISAITY
SCFTIALLIYAIVVYNSTGHCRQMNKDMGLHEFCGSFTSIRLPFEGNSLIIRLPIFLTQMFVN
ANALMSAAHITFFVYEITQYLVMHVDILKQNLLQVYDVGSSEEISQKFKSCIMYHNRILRL
MIRLNQVTKFTVGELSLTAAIIFACIGNQILKGSSMAGIAYFQGYVTELLFLCHSGQIIMDQT
ESIGASLYQSKWYEVEAKLMRNVIPVLRRCQKPMTLQALPQGNFSYALFLMMMNSAYTY
FTLLAQTT
>McarOr22
MATRYPADYFGMIKIMYGIAAIWLFNPNGSLIVKCLKYSWTLILYSIVVTFVIFEYIMIEVM
FKDIFTLIAQMGLLLCGHISLLKATVLIKNHKKLAEITDFLEDERYHYKSVGNFDPGKLVV
DEKRFTNNFLKMLLVCFGLVGVSPHLAAEKIIHQEVKGNYFSENVTCYDYLPFIFYIPFPSE
TKHMCEVAVLFMDMSISGIAIIIACHDGFLVVLLNCVRVQFVIVGEAFSTLRERVLHRLNLP
EDFEIFYDEQHPQLEAELYKELNVVTRHYALLLKISEDLEEIFNKIILVQTLLCLLVFATCIYA
GTTVPITSPTFGASVQCFSCVLAELALFCWFGNGVTTSSEGILLALYKSDWFSASKRFKSS
MILTMTRVQKPVYITLGKFGPLTLVSLVSVCKASFSYYTLLKKMNI
>McarOr28
MNEGFLKIQRICMILLGKWQFDQKNLMQNKLYKIYGWFIIIYYFAVIQSMPFVIKSRWACE
DIITKLGTIYLNHMNVFMTNLMSNTDHLKKLVSYILNYEKITYPKETDFVRKTYDFYCAM
NYKMTALYIVTPSSLAIVYYAFEVTFTFLKDENDPCAPNKGRIYQFWLPFDTNKYFYVAMP
FELLEITIVAILNTYNKLIPSSMSTFQLGQIKILQEMLRHVDDEAIKLHTNQHIEWEQAVDIT
VTKCAKKLLEILSLMDILHKATGPLMLLVFSSNALETAIFIIRMLRAKTIVESLISVGVSFIIF
VQLLSFFWQANEVYLESQNIIHVIYNETNWVDYNVSVRRKLIMMMTKCQKPLSFQGSGI
GSMTIETYKKLVKSCYSVVTFFTSSYG
>McarOr29
MGRKFLKIHRFYMLILGKWKITPRNTLQNKLYKIYGWFIHIFFIGILQTMPYINRTKINCWD
VITKRGALYMICNNMFIISLISNSVNMQKLVEFILNYEKLLYPSEKNFVQKTYDFYCKMNY
NIVMIFIVIPSFFAYVYYGFEVILTFLKDPNDPCFTTKGLIFQFWLPFDTDKYFYIAILFEFFL
MSLAICFNTYNKLIPCSMSTFQLGQIKMLQEMLRHVDEEARELNASQCVEMDEAVDAFV
TECIKKLQDILSLMNLLHKATRPVMLLAFFTNILETAFFMIRMLTAKSDAEAITALGVSSVIF
IQILCFFWQANEVQLESQNILDVIYNETNWVDYNISVRKKLLIMMTMVQKPMSFEALGIG
SMTIETFKKILKSCYSIVTFFKTAYN
>McarOr30
MKLHKLLRLQNHLLILLGRFEESFGNIFVDKLYYIYSWFISCYYILMLQSIPFIVLTKWECTE
LAMKMASIFLHHSNSFFLSRLAIKPPMKKTLSHILNYENLIYPNEAKDRQNKYNHFANLNF
IVSLLAVVLPTQYGWLYYYLEVKRSYIEQENPNCALKKGLAYQLWYPFNVEKYLYIARIF
DFLELLMATIYHSFNKSLPIGMACYQLAQIEILHLMLRNLDTDAKKLQHGNIGRDEAVETL

LNECIRRHQIIIDFMGLANKAMRTIMFIVFFTSSMEMAVFLIQMITAQTTTDRLLTCGVSCV
LALQILSFFWFANEVFLQSMKVSEIIYNEMNWVDYTIGQQKKLVIMMAQSQKSLSFRATM
VGAMTLETYKAMIKSCYTLVTYFKTVYE
>McarOr31
MGCRFLKVHRIYMLILGRWKVSSRNTLQNKLYQIYSWFVPIFFIGIIQSMPYIIRTQMNCSYI
VTKRASLYMHSTNILIISLLSNNANMQKLLKYILDYEKLIYPSEKKFVQKTYDFYCKMNY
NIVTVFILIPSIFPYIYFTLEVTLTFLKDPNDPCATTRGLMYEFWLPFDTEKYFYIAMLVEFY
ELFLAVCFNTYNKLIPCGMSTFQLAQIKMLQEMLRHVDEEARELNASQCVDMDEAVDRF
VTECAKKLQDILSLMDRLHKATKPVMLLAIFTNSLETAFFIIQMLTAKTTTEAIIAVGLSCVI
FSQTFGFFWQANEVHLESQNISDVIYNETNWVDYNVRVRKKLLIMMTMVQRPMSFEALG
IGSMTIETFKKILKSCYSIVTFFKTAYN
>McarOr32
MNDFLKIQRICMLLLGTWKLDPKNVLRNKLIKIYSWFVIIYYIGIIQSMPFVLKSKWTCIDF
IKKVGTLYLNHTNVFITSLMSNTDNLKILVAYIMNYEKLIYPTERYFIQNTYDFYCKINFYIT
AICIVAPSSFWCCLLCYRSYLHIFGGMKNDPCASMKGRIYEFWLPFDTKTYFYMTMPFELF
EILIAVVLNTNNKLIPSSMSTFQLGQIKILHEMLRQVDDEARELHESQHVEFDEAVDLLVTK
CVKKIKNEILSLMDIMHKATRPLMLLVFSSNSVETALFIIRMLTAETVGEGLVSVTVSFMIFT
QLLTFFWQANEVYLESLNIINVIYNETNWVDYNVSVRRKLIMMMTQCQKPLSFEGLGIGI
MTIETYKKLLKTCYSVVTFFETAYD
>McarOr33PAR
MDPVDKMSDYFKHNMISFKYTGIWLNLFDVRRTSLLVIFYSVIINSLFMMSPQVCHVIYM
YKARNNVQAFADEFYVSLASLLVVLKSYSLLKNFDIIKKCWKPWTLIFFKQKRLSAKANT
AGYGNMEMIYWLYATFALLYVFLLLISVLLERILQGTKVLPLVVCYPFEVNVSPVYELMFL
YQAIALSWLVIQNFNLDTFITGLLTVAAVQCDLLCNDLENLTPEKLECQKGEENDIMDEKL
VDCIKHYQEIRRFVNDISHCFSMNIFQQFSCSVITICTTLFEFSTKEPLSQEYFAIIIYQSSIFIQ
LFIFCWTGSELTEKSKRIPISAYASKWEDASKTFKSNLLIFLHNVQRPLEI
>McarOr34PAR
IVNMIMVLGDIEKMTEASFLALTHLVQVMKLFYVLRYENKLKLLINSINRKSFQPKNLEQY
VILQKYVRESNIISKTFLSAGFVTCCFWGVSPLTQSGDIVLPLAGWYPFDTDRSPAFEIIFAY
QFVASVTNALSNISLDTLMSGLIMVVCAQLNILNDSLRNIRKYAEAESDDGRAVSREELQR
RMDERLVECVVHHKHILEFSNEVTFLFTNSILGQFIVSVVIICITLFEITLLPWGSLKFFSLILY
QFCMLLEIFLLCYYGNEVILQSMQLTKFAYFSDWTDCSTKFKRNLLFFMTRSQVPLRIYAG
GFFTLSLETFVKILKSSWSYF
>McarOr40PAR
IHTVTAGEYNHIDLDLSYVLSMTSGFLLFTLFSFNITTATKMYMFLSEFKEFGKPPKFDKYN
AFLNKVAKFHVIYLNINITLFAAGSNVFKGAQCKKDNIELGYKEICGLVTNTYLPFDIDYFP
LKQIYVGLQFFSIYYVYTISGTITFMVMETMMHIGFRLDHVKQLFDEAISEKNVERSRKRF
NFAARYHARVLELEHEVNACFSYAMFSHMILTAAIIGCAAFGVMQSGSANPFAVCIGWFN
GISFVCLSDQHLINKSLEVGTAVYSSKWHQAHPSLQRDLVIVIMRCQKAMILRSAGFGVM
NRATILAAVQASYSYITLLSRSP
>McarOr48
MSKRIMLLLGLWPATSSHDWKYICYKAYFCAVRFNYGVFIITLIMGIFSKYTLNAGPLEKA
DIIQNYLLYILLLWKMILITTSQILKLIECISEREMHIMNLEEEDIKNIYAKNVNYNFKIFLIVT
ATIVVGLTQFCILVSFEIHGLKNNIERKLPLPEWFPFNVGKHFLTSYYFQVFNRIYSGMIIVA

MDSMYFSLIYFPITRLKILGHNLMFFQELCNKNYSETPDNILKALILEHQDIMRYVKDFNR
LMKWYLFMDFLVRSYHISLVLFKIMALQEFYGNGSDGVLLLTFSINYLMVLLLQMHIFYY
HSNELRLESMEISSYIFQGNWYDQSPSCKKSFLIMMMRAQKPLEIHIGNLNTITSYLIVKVL
KAGYTYVILSRK
>McarOr52
MRYEFLKIQRICMLILGKWELNSKNELINKVYKIYGWFVPIYFIGIIQSIPFILRTKLDCTDIIT
KIASLYMISTNVFVISIVSNSANMRKLVSYILHYEKFIYPTEGFLYKKTYDFYSTMNFNIITL
FIVCPSSFGIVYYGYETSVTFWRDENDPCAPKKGLLFQFWLPFDTEKYFYIAMMFELFEVC
LLVIINAYNKLIPNSMSTFQLGQIKILQEMIKHVDEEARELHVSEYFEMDKAVDILITKCAK
KLQEIISLMQILHQATRPVMFLAFFTNILETATFIIRMMTAKSAGEGIIAVGVSCIIFIQIVSFY
WQANEVPLETQNVINVIYNEINWVDYNVRSRKKLILMMTMCQKPLYFEALGIGRLTIETF
KKIVKSCYSIVTFFKAAYN
>McarOr54
MVLFPKNDHFKVTMYANALLGVWPFIFEHNPFMRKLYHIYANFTFIYFMLFIVTAYMELV
VLLMAKELRVQEIVGNLCITLLYSITIARVYAIKSDSVKNLIREVIEVEEVIYKSDDEEVIGIY
KEYTHHSHISNIIFLVNITIETIFYFTHPLYVGETIVIDEATNATKVVRALPLSSWFPFDEQEY
YHLTYGWQMADGTVGASYVMYTDIFTFSLIIFPLGQIRILMNILRNFDKYVKMTQDQYGY
ERDEASFLTARECILKHKNIIRYINEYNRVMRNIMVFDFLQSSLQLASIVIQLFVSEVRLFNV
IFHGEFALCMLIRLLVYYWYANEIMVQSSNVALAIWDGGWYEEPQKVKHMMMMMIMRS
NKPLVLDIGPFSPMTLSALLGIMKATYSYMMIIYN
>McarOr56PAR
SPHVTTVMSLQPRSKGIFKNLTCYDFMPYTFLYPLFPSQTKRRCEIVALYMDIAISGVALGI
ACYDTFFAAILNCLTAQLTVVNKAFRTIRQRVLQKLKVASNIKLFYDDENPRLEKELYAELT
FTTRHFVALLQIAEDLGKIYNLVILGQTVECLLIFAACVYIATTVPITSPEFAAASQYFAAVF
VQLTLFCWFGNGVTTASEDILLALYECEWYSASRRFKSSLLITMIRMQRPVYLTLGKFGNL
TLVSLVSVCQGSFSYYALFKKY

>TcasOr1
MMKFKVTGLVADLMPNIRLIQASGHFMLNYHADNSGALHTLRLGYCCMHLVFVLVQTFS
CNFVNLVLERGDVNDLAANTITVLFFTHCVTKFVYFAVRSKLFYRTLGIWNQPNSHPLFVE
SNNRYHGIALKKMRRLLYIIIIWTSFSAIAWTGITFVGDSVHNIKDPENENLTITEPIPRLLVK
AWYPWDAMSGMPYYITLVFQVYYYVFFSLAHANLLDSLFCSWLIFACEQLQHLKEIMKPL
MELSATLDTYVPKSADLFRAPSATSQDQLIENGTNPAKKNEDLKGVYSTRQELGGHFRGG
ALQNFGSGGVGPNGLTKKQELMVRSAIKYWVERHKHVVRLVTAIGDAYGVALLLHMLTS
TIMLTLLAYQATKITGVDKYAATVLGYLLFALAQVFHFCIFGNRLIEESSSVMEAAYSCHW
YDGSEEAKTFVQIVCQQCQKAMSISGAKFFTISLDLFASVLGAVVTYFMVLVQLK
>TcasOr4PAR
MYSENKFDFVLTIKPNILFLKIVGLWPVDNNDVYRIYTLIVTVFFMGVFDFTRIMNIFFVYT
DLKVLTATIYLTVTDITVLVKTCLFMSNIKTLKRLIVTINCDVFQPKTDHQKQLVQSGLKA
WKVSYMVFWSLVFCCLVMWTVSPIIQATPAGRKPLPLPAWYPYNTDITPFYEITYVCQVIS
MWFLATANMNMDSLIAALMIYVGAQCDILSDNLKKMKTFSKIKEEQAKFNQTLIDRIEHH
KKILQFAYDCNASYNFIILAQFFTSSLAIALSMFQLTLVDPLSMESFPLLSYAFGMALQIFLY
CWFGNEVEAK
>TcasOr207PSE

MVEFNGPFIMLRTILFIDMNTYKILKLFNVLLNVIYSLIHCLLVYHMFKNLNINLIIRYGPAV
LFLILVIVGAVFSVYLEKDILEIVTLFRKTRWSLSMIKKDARIKLEKKCKIINIFILFLVLLIIST
ITINAPYFGDQRELFICIQVFEEYFGEWFFIPYNFFFVAFPFLYYNFFKLWMTFVYGILEAQL
QFFILEEYLCGTFETDFRKNWEYLQDTRYQQEIGTSLRLCIAHHINLKKLIKMIQNVTLMV
MPFFLVLGVLILISSFSFIINFADTMTTIAKIRMVISAISMVGITILLSWIGQQVIDVTSDIFVTL
GGASWYYWNQKKYXKTLLMFLTNSIKNESIVLAGICADYGLCVALLRLSVSYALVLFNLR
KSTLVZ
>TcasOr59
MDEEFLIGTFETEKKFLRYGSFYPCGKRIKFIFLGLFMFVYSWTEFLSMITVLFVERDNLTK
LSETLLFCMTQAAFLFKLVNFLYHNKTMLRIESILKNPILNCLDQFEKNIIEKYMIRVKYLA
RLFRILCILTVSFYGLFPFIDEDPDHMLPLPGWFPFDVKTHQIELVIAQTCGIAIGAFLNSTLD
ILPTILITLGSAQFDILKIRLENITSVDTSKSWLVKKAIKKCVIYHTILLNYITQIEILFHKGIFV
QFTASVVVICLTGFQMLVISVRSIQFILLMIYFSTMTCQIALYCWYGNELMYRSMGLSDAC
YMSEWNKCDTSVCKSLAIIMERGKRPVVLKAGNIFSLKLTTLMTVLKSSYSYFAVLQRLY
ATSE
>TcasOr63
MGFMIQDYDLRNAFSLERKLMLVVGFYPKRDNKHEILYWLSAFFNLLISYGQLTTMIIQM
VFDRSDLSKLTESLLYFFTHFTFLCKLLNFQYYSKDLIEIENFLTDPIFYGYSFEQLDIIKAKI
RSCAFISNAFRICCTFTCSFYCLVPFIDESRKKILPLPGWFPYDTTNYYYSTFFVQSLSLFISA
YCNTAIDILTWKLITLASAQFEILKENLTKIDYEGGFNETKGALVRCITHHAKIVNYTERVE
AIFSKGIFLQLFGSVIVICTTGFQLIVVPIPSVQFAVLGTYLCGMTTQVATYCYYGHEVMTTS
DAIGMSLYLSNWYASHVKIRKIVMIFLEKTKKPTIVKAGNFITLSLATLTQILRSAYSYFAVL
QRLYKDSZ
>TcasOr67
MDFTIRDFDLRNSFSLERKLLLVLGFYPIRDKEKHRILHQLSAFLNLLLYYGQLLTIIIQMVI
DRNDLSKLTDSTLYFLTLFTFLCKLFNFQYYGKDLIEVEKSLTDPIFYGYSFHKLQIIKAKVR
SCTLVCLAFRISCTCSCFIYSVVPFIDRSGQKTLSIPGWFPYDTAKHFYITFFLQSLSLFISAH
CNSATDTLPCKLISLATAQFELLKDNLRTIDYENSFEETKHALVKCITHHRKIVNYTKRVETI
FSKGIFLQLFASVLVICTTGFQLVIVPFGSLKFAIHGIYLCAMTAQIAIYCYYGHDVMITSDEI
GTSLYMSNWYASHIKIRKIMVIFLEKTKKPTIVLAGNFITLSLVTLTQILRSAYSYFAVLRRL
YADDZ
>TcasOr70
MPSIIDISFKININVLCLAGLYLPDKFKSLYRVYTYLVYVFIVIPVPTLGCVYLLAQEKITFRQ
IADNLFLIAELGCFIPKYWPLVRHAERIKRCIHYFSAPIFKTDRKEHQEILDDCIKVCHQWS
AFYFASVTAGFVSWSIRPISWENHILPTDIWLPFDPHTASSAKVASVYFYLVLGKGFGLGIKI
LKNNLQHLGEYVDEELASLEPCRKAQLTYQKIRQCVIHHEHILAFVEEYEECFSQVALSQF
VGAVVIFCVSCLQLTIVEVVSLDFLAMMMYFIAMLCQVYLYCHFGTILYDESDTISDAIYLS
KWYEFDKRSKKALCILMERLKRPMTVTCGKIFTMSLVTFTMILRRAYSLLAVLENYNIELN
Z
>TcasOr27
MLETKQIVFHSFKLNVTVLSLIGLYPPKNYSILYKIYAVILFLAVHTPQLVLGLLHYFLMGDF
TSIDYSDFVTVGMMFYAFKLLPFVTSVTKIQKCINYFDTLGYKILKSEEKIIEDCVGSCRRN
TNVFFVGCCLSWMGFVAQVFLRDEPQQLPLKVWFPYSRDESPVLFYCIYILLIFGPGYSVL
ACGTIDPMIGGLAYHAAAQLQRLKRNLQYLDEYIKEKNVGKSKENKRGVIYEEIISCVQR

YQEIATFVDLFKDSFSQVVFSQFMGSVFLIGLCCFQIITATEVDINFVITANYIWVILFQIFFY
CYYGTMLIEENYTLTNAIYLSNWYEYSIPEQKALFMLMERSKKPMIVTAGKILDLSLDTFT
MILRRSYSLLCCLKZ
>TcasOr36
MKGLVEKSFRVNLLVMQVMGFYPPQKYKSLYKIYTYVVYCAFTTLIPVLATLELFLAENIN
LEQISDNAFIVCEAGCFIIKYLPFVRNADKIKKSLFLIERPMFHIYTKRQEHIIEECVAICRRN
CRLFLTFCTITVINWSITPFFLPGNNLPVEIWSPFEHKASRKFYFLSFVYIVAGVGNAAVSSG
VIDPLLAGLISHATSQLKVLKNNLQFLDEHAEERIASRNISFIERKRFKADFIYQQIKLCVNH
HIAITEFIDVYEDTYSSSVFIQFAASVVVICISCLRLSMVEPFTFTFFVMALFLWTMLCEIFLY
CYYGTILYEENHSLTNAIYMGKWYNYDIKSMKALVILMERSKRPMIVTAGKILDLSLETFT
TILRRAYSLLLVLKNYESTPTEZ
>TcasOr40
MSSLIQESLHINLRVLEFFLLYTPGEPTNFQKLRSSILFFALMFHVPVLSGINLIVGKHDNPM
KLVDNSFGFVGLSCYIAKLWPLIGNRSKIKVCINYLDKPIVELRENQKGILQACSKICRRNS
NIFLYYMIISVTGFVTKPFLFEERGFPVDVWLPTSLKDRLDVYWGFYIYVSIGVAYPVIASG
VLDPLIPSLLCLATGHLKVLNDNLEHLDEYSSEENGSKDSNLYKNIQKCIKHHIEILNFVYN
HQKCFSLMVFSQFLGSPMILCFTCWNVSMREPFSLEWFQSLAYFLGLLLQLFFYCYYGTR
LSEEFEHVTTAVYMGKWYKYDVKSRKALIILMERSKKPTIVTAGKILDLSLETFTIILKRSY
SLLAVLKNQNZ
>TcasOr32
MCLSTSEQSFSINLKIMKLCRLFPPTEGKKFYKIQAYLLQFLLLLPIPILGNLHLLLDENLDM
EKVNYNAVFLAQVTCFVIKLMAIIANSEKIKKCITELDSPKFAAVRENHKIILQHCIKVCKR
NTLIFVVFVICGASSWATKPLFWSRRNLPLDVWFPLDTTSTPVYCSLYIYLLIGVYFTSFAN
MVIDPLIAGLAYHATSQIKILKDNLQHLNNVYANEEITSSKNKIIYMKIKRCVQHYDDILSF
VKEFEECFSLAIFSQISASVFVICFSCLQLSKIKTFGYYFIQLVFYFGVILAQIYFYCFYGSTLF
EESSSIINAVYSSKWYDFDVPCRKALLILMERAQTPITVAAGKIMDLSLVTFATILRRSYSLV
AVLNNYQZ
>TcasOr52
MSQIDLKEAFKQNIVLLKAMGLWFFQNERFYKLFKCFVQGSLVFDSTSLIIYVALNIRIKNV
TDTIYSLPGSLEVVLQAILFRKNFHLIRKSLNNLKQKEFQPKNDTQEKILKDSIALSRRVFY
SFFWLVFVMIGMWMVLPLTKKGKYLPTKYWIPFDYRLPVVYELLYVFECSCIIFHAFSNVA
LDTFFSIAMIQIGAQCDVLCDTIRNMDEQEKTNTMDRILIECVHHYRLIEDFAKSIATSFKEI
LMVQFVCSSLMLCVSMYELSLSEPMSGHFFQVLLFQISATNEIFLYCWFGNEVIIKSERLFY
AMFESKWYDSAATHRKNLMIFAHQVQKPISLLVWNIFPVDLKTFGGLLQKCWSFFVAMK
NIQEIQEZ
>TcasOr48
MPHATLSKMVVQKIDLLEPFDNVTRLLKILGLWYSPNETIVYKIYKNFVMATCFLYTLTCT
VYGFKFMSFETLEIAFGAVEGVLKSLMFRLKFQKIAESWQQIRQQEFQPRNEHQRTVLKW
YIEVTKSLFLVYFFGVYIGCISALTVSSWLRHKDFPTDHWFPFNYRRPFLYQYIYVHITVGF
YLTAFLNCASDSCFYLSLLHITAQCEILADTLKNVHDLHKLNAAKKNSGQKGEDEVMNQI
LIECMKHYNLIKKYTSLVADCFKEIITLQFVPTIVMICIAMYKISTLEPSNTQFWFFAFTELG
AITQIFIYCFVGNLVTSTSQKLFYATFESQWYNASQKFKKNLITVMMAVQRPVIFYGWNIF
AINYATFKSIVQTSWSMCVAFRSTQDLZ
>TcasOr49

MVVEKINLREPFENVTRLLKILGCWYFPNESLVYKMYKNFALITCCMYTVTSIIYSFKYMS
IDYDKAYESLEIGVGTAEGVLKGIIFRMKFQKITESWQQIQQPEFQPRNEKQKMLLRRYIY
VTKFLFKVYFFVVYIVCVTGLIVSSLLRHKDLPTDHWLPFDYRKPFLHQYIYLHLTAGLYL
NSLTNCAVDSCFYLSLLHITAQCDVLADTLKNIHDLDKLNAKNAPERENKDQVMNKILVE
CMKHFNLIKKFTNQITDCFKEILTLQFVPTVAMICMGMYKISTLQASSSQFWFFVCTDLGA
TTQIFIYCFVGNLVTTTSEKLFYATFKSQWYNASQKFKKNLLTFMMAVQHPIIFYGWDVFA
INYETFKSIMRTSWSICVALKSTQDLZ
>TcasOr50
MVVEKINLLEPFENVTRLLKILGCWYSPNETAVYKIYKNFIIATCFIYTVTCNIYVFQKMFT
DSDKAYETLEIAVGSAEGVLKGIIFRTKFQKITESWQQIQQPEFQPRNEKQKSVLRRYIEVT
KTFFKVYFSLVYVGCVTGIVVSSWLRHKDLPTDHWLPFDFRRPFLYPYVYVHVTVGLYLN
SFTNCVLDSCFYLSLLHITAQCDVLADTLKNIHDLDKLNGKNVPERENVDEVMNKILTEC
MKHFKLIQKFTNVITDSFKEILTLQFVPTIAMICISMYKISTLHPSNTQFWFFIFTDIGATTQIF
IYCFVGNLVTTTSEKLFYAAFESQWYNASQKFKKNVITVMMAVQQPIIFYGWNVFAINYE
TFKSIMRTSWSICVALKSTQDLZ
>TcasOr55
MKAFSFNIKLFQIFGQWCYENESFYKIYKYTATVLLFLDWLFTMIFVLVNFQESEVVDSLYI
SPSMTTSMLKYVIFRVNFSQVERMLKIVEEQYLKIDSRRVGFLVERGTKSSVFVIKACFYLV
MATVVSLITQPLLQEDIDIPLVIWLPFDYHRSGIFELIYVYVSVSYLYFAYVNVATDCFFYIS
AIQIGVQCEIVGFMLENLNEIAKQEEENVRRLFLSCVTYYNNILECVKIISDCYREILIVQFF
CSFVALCMTMYQLSIVEPFSDVFFKMCVFQSAVICEIFLYCFFGDLVLEKSGKLFYASFSFG
WYNGSAKFQKELLIFMNQLQKPIIFHVGNVIPVTCETFKSIMQKSWSFFIALKNTQNRZ
>TcasOr54
MNLQKLDPLEGFKPTISMLKIFSVWNSSNMFYKIYKNVTTLSLAITYTCVMICVVVNFNV
SEINENFYYIPALSTAPFKLVIFQKSFKKIQNLLFLLQSQYTKIRSEKQAKMVEDSVVLSKRV
VKVFAVLVVPTCVGLFGMPLLKDEIKLPLIIWIPFDYHEPVVFGLVYFVISFSGSFTAYINIGT
DTFFYNCLIQIETQCNILSDTLRNLHEFGRFEAEIHTILIECIEQYKTILKFTKILSKTYQGILS
VQFICSLLSLCLTMYRMSLADPGSEEFLRYFVFQWGVLPEIFLYCYFGHRVLDSTKNLYYS
TYELQWYNTSAKFKTNLLIFMGQIQNPIVIYVAGIFSLDLETFKKIMQKAWSFFTALRNIHE
QZ
>TcasOr57
MSHSNPLEAFKLNTFFLKALTVWHVENPTYRLYKIFVVFSFAVTFFSAWICALVNYNVSEIS
ENFYYLPAMSTGPLKYAIFQKNFTNIVNLTHLLETQYAKIRTENQKKIFDESVIFERKVMKN
FAILIIPTCVAMFIVPYFQDRREMPLIVWFPFDYKQPVVFDLVYFILAFACISIAYTNVSTDAF
FYTCLIQIETQCEIVSDTLRNLDKIVTNGFRNVAESRKIFIECIEQYNVILRYTKIVSDTYQGI
LVVQFFCSLVALCLTMYKLSLADPGSQDFIKYFVFKLGVISEIFMYCYFGHRVLEKTEDLYF
AIYEMHWYDASKQIQNEVFIFMGQLEKPIVFYVANIFSLDLDTFKKIMQKAWSFFTALKN
MHDIRNNZ
>TcasOr23
MANFNWTKIIETNFVVLKVIGLWPEKTFFACKLYNIYTHFMVTCLLVIHLLLQTIQLALIID
EFQLFLTALPLLLQQYHLLIKLFYFMVKFPILRYILHSLNNHQVFQPQNQDQKIQMEDRLSF
MKKIYFSFYSMAGVAISFLVAFPILDILNGGERQILFVCWFPYDYMTSPFYEFTYFYQSASII
YAGVIVLQIDTLVTLLMTYLGFQCDLLCENLTQVGYNNSKENNTELEFVKCIKHHQELIKL
KNHCVDFSSGLIFVQVATSSIAIGLTLFQMTLNVSTFNVIFLVLYGLSVTFQMFQYCWFGSE

VIHKSDKIAYSAFEMNFVDAPLSVKENLVIFMACTQKPIKMPVLKVTHLSLQTFTKVLRTA
WSYFALLVQVSKZ
>TcasOr14
MLLKWSSVIEFNLFLLKWIGLWPGEDYQLNMYSFYGFSVIILILCGHTLSTGLTLILDSGDI
DTFTETMFILNIEFMTAWKALNFALNRKKFMQLLDAIDKTTFQPRNGKQVTLVLRNIDGW
KVMFKMFGISLGLSFIFTGLLPIFSKTYKDRKLPMEAWYPFDSTKSPFYQLCYVYQMAAV
AVAVMVILNVDTLVAAMNICIGLQCDLLCDNLRNLHTNTSKSMNQKLIECIKHHQNIISFAE
KFRQAFNWSIFLQFFVSTTSLGIVMFKITRFSLYVSEYYRFISYACSVLVQVFIYCWFGNEVI
VKSSKIPYALFESDWTQDSLEMKKNMIVFILRTQKTLKITVCHVFDLSLPTFLTILKTGWSY
FAFMNRVTSPHZ
>TcasOr15
MLVKWSSVIKINIFLLKWVGLWPGEKYQLNVYSFYAFTVIILILCGQTLSTGLTLILGSGDV
DTFTETLFVVNIEFMTAWKALNFALNRKKFIQLLNAIDKPMFQPRNDKQVTLVLRNIDGW
RVMFKMFAISLALSLIFTGLLPIFTKTYKQRKFPYEAWYPFDSSKFPIYQLCYMYQMASAS
TLVVVILNVDTLVAAMNICIGLQCDLLCDNLRNLHFDTSKSMNQKLIECIKHHKSIIRFAEK
FRQAFNWSIFLQFFISSTSLAIVMFKISRTTNYGSEYYRFISFACSVLVQVFIYCWFGNEVIV
KSEKIPYALFECDWTPEPLEVKRSMIIFIIRTQRILKITVSYMFDLSLPTFLSILKTGWSYFAF
MNQVTEVNTSKZ
>TcasOr11
MEKYDWMQAIKTNILILKIVGLWPDSEDYKFDFYALHASVWLSTLLVASTFFQGINIIFILD
DVKALTGTAYVLLTEILAVIKTYFVVKNMKMLKHLMQSLNNNKLFQPRSHEQIKLIQPSLK
FWKLLYNLFHSLVGGATLFWILFPIVDKKEKRLPFLGWYIVDTKVSPYYEIVYGFQFCSCC
YMSALIINIDTLIAALNVYIGNQIDILCNNLRNLKAGCSIERDLITCIKHHQEILNFVQYANK
FYRWIILLQFFVSAVSIGITMFQLTIVVPLSSEFYSFLFYANSIISQIFMYCWFGNEVQTKSNK
IPQAIFESGWTDFPLKTKKDLVFLLMKTREPIKVSAFNLFSLSLDTFMRILRTSWSYFALLN
QVTZ
>TcasOr7
MNKLQKFDWKATIRPNIAFLHYLGIWPEGEEYYKLNFYTLKTILYIIILVISTIVFQVINIFFT
LDDLTSLTANIYVLLTEILYFIKLCFLVKNMPALKLLMKTLDHKLFQPKANQIVIIQPLLNFW
KLIFLAFVITCSFTVLFWAIFPILDSSEEEKRLPLLAWYPYDTKISPNYELTYLHQVASYIYIC
YSHLNIDTFITALNTYIQCQFDILCDNLKNIKSDTKNVDTKLAKCIKHHLLILMFANTSNEF
FSWIIFFQFTSSAAITGMTLFQLTVVKPFTTEFYNFMAYVTAEVVQIFMYCWFGNEVQVKS
SNIPYAAFGSDWTEFSPNKQKSLLFLITRSQKSVKMSAFNVFDLTTDSFILKSAWSYFALLN
QVNSZ
>TcasOr12
MQKFDWRSMIKMNIVVLRCVGLWPSGEESYKPGVYTIYASTVLTLFLLGHIFFQAVNVYFI
RNNLSAVTGTIYILLIEILLVFKVYYLVKNMTVLKQLLKMLETEMFQPRNSTQINEIQADM
KFWQMLIRFLWVSVMCSNLFWAIYPLVDNAGKEKRFPFLAWYPYDAQKSPYYEITYVYQ
TISVNYMSSIHVSVDALAGALNVYNGNQFDILCDNLRNLHRLTKNGTIDAGRNFRYCLKH
HKHILDFAKKCNNYLNWILFMQFFVSTISIGITMFQLTVVRPFSNEFYSLFTYISAIIGQIFM
YCWYGNEVEVKSSKIFYATFESDWIEFSEEVKKELIFFVMRTQKPVKLSALNLFYLNLDSF
MRILKTSWSYFALLHQISNRNSZ
>TcasOr10
MEDFSWEATLSQNINFLKVCGLWPPGDEAYKFNLYGIYAGFCVLGFLCVHTGTQTFNVYF

ILDDLEAFTSSIFVTFSCVACVFKTYYLLKNMKLLKVLFININKEIFQPKNKEQQLLIQPSILF
WKRFYLVFRILCYNTCFFWCAYPILDKRIKQHKLPFLAWYPFDSSVSPLYEITYFYQAVAIW
YIVIISFNIDMLIGALNMFVGAQCDILCDNLRNLGKSDINELNPDLIKCIQHHKAILSFVSKL
NIFFNWIVLLQFFSSAVSVGFTMFELTLVAPFSGQFYSFICYGSAITTEMFIYCWFGNEIEIKS
SKIPYAAFECNWVGTPLGVQKSLIIFTIRTQRPMQVSALNLFYLSLDTFKTVLRTSWSYFTV
LNQVHSZ
>TcasOr17
MDDFNWISTVKTNLLLLHIGGIWPRGDGTHKLNLYTIYAIFITFTFTTYHCFSQIINFFFVDD
LQALTESIFISLIQSMALVKAFYILKNMRILKNILKNLETNKMLQPRNLKQIKMVQPSLTQW
RLLSQMFWISAVFAMCLFGAFPIVESTYKEFRLPYLAWYPFDTKSSPFYEIMYLHQFVSSY
TIAIVDIGADTLIAALNVFVATQCEILCDNIRNINGSVEEMDSKWKECFTHHKEILKVARHC
QKFFNWIVLMQFCASVICIGLTMFQLTLVVSFSSEFFSSLFYFGAITVQIFMYCWFGNEVEL
KSSKILYATFEANWVEAPHQVKKNILIFAIRCQNPIKMSSLNVFYLTLETFMAIFRTSWSYFA
VLRQIQNRISEEZ
>TcasOr39
MSNQHEIDLTEFVKLNIKNMHFFGYFFPRFGHNKTRKTLYTMYSTLFVGSTFVLTALSQIA
NMINSFGDMERMTEASFILFTNVVQCFKIYSFLTYGPRVWNLIDGLNRNIFKPINTDQHRIL
VNDIYMSKKISKIFLLACTLTCMSWAISPFFDKRGDVLRLPLSGWYPFNTDKSPAFELVYIY
QILTTWIGGMGNISMDTFISGIIMAISSQLSILNNALKNITKNNELVRCVFHYRIIIKFSDEVIY
LFNTCLTTQFIVGVIIVCISMFQMSLVPVLSFQFVAMLLYQMCILLEIFLWCFYGNEVMLKS
DQLTQAAYMSDWTKSPNHFKQNLLFFMTRTQFPLKLYASGYFTLSLETFKAIVKSSWSYF
AVLNQVHSRQTQZ
>TcasOr41
MDNTLDIDLTEFVRFNVNSIHFFGYFLPEFGKHPKKKIIYVIYAVIFVGTTFGLSLVSEIANMI
NAFGDIEKMTDASFLLLTNLVQCFKMYSFLTHGPRVWKLIHSMNNSDFKPKNLEQRNILV
EEIKMSKRISKTFFMACTIVCSLWGISPFIDRGNSEKLRLPLSGWYPYSTDTSPGYEITYAHQ
TLTTWIDGLADVGMDTFLSGVIMVIAAQLSLLNNSLKNLTKNCKNDGKKANTNLIECVIH
YRTIISFADEVTYLFTSCITAQFIIGVIIVCVSLFQMTLVSLRSFQFFSMFLYQGCVLMEIFLW
CYYGNEIILKSDELTRSAYMCEWIEESREFKKNLIFFMTRTQFPLKLYASRYFTLSLETFTAV
VKSSWSYFAVLNQVHTKZ
>TcasOr65
MTATKSLKEIPPIYLRVHLTVLQILGIDILPVESVPQNLFYTYTALIISTMCLFTIAEFLDMVL
NYEDIYRLTFGLCYCVTHVLGTVKMFLMLYLRKKLWGNLTTLEEGIFKPNPTRGGPEELQI
VNDAITMCNRQGYVFYTLVFLIIGARLLYASLANWPYDKHNYFDGNVTVIVNTKEMPYTT
WMPFDYNDSPLYETIFAFQIFSTTVYGFYIGAADAVICGFMMLIKAQFLIVKRELETLIERA
QKAAIAENPDNEDNFGREIERIELLDKRTQDYVAKYANECVYHHQELIALCDHAEEDFCY
LMLLQFISSLLIVCFQLFQVSTLSPDSVEFFSMVCYLLLMLFQLLCYCWHGNEVQIVSGEL
SRYAFGINWIIMRESPKKTLLLLMMRAQRPCYFTAGKFSLLSLQTFMTIVRGAGSYFMFLR
QMNIZ
>TcasOr198
MPNVTNKRQKRLFSKTRTKSEDPFVMIKDVFVDGGYHPVTKMLNYICLVIHSCSLLLELN
YFVHNYHFDLMMKYCCAMSLMGYIIATMLFAIFQEHSAIDLTKDILSLFWPIDYCGPRVKE
EIVKKATKINRIHYIVLLFAGALGITMFPIWGDQKEWFLCVQVYQHYFGKWSKIPYYVYFF
TYPMLAFSSVRLPFMTMYAIVQIRMQVYLLHQHISEISGEYVYDMKNLQILCDQNYQNEI

YDKMRLIISHHIMLKRWMRKLVHTVQISMPVFVLLGTMTSISVLFYAIYSFHNINFILKVRL
ISVSVCTVLVVYMFSEAGQALSTETTGVFDLLMTCPWYVWNIKNRRILLIFMANSLEPMT
FSLAGVTLDYRFALGMLRTSCSYSLILYKLKTGIZ
>TcasOr229
MSARPLHLRNFPYYFLKVLVFDFEQYSAGKVLSYFCAIVHSISIFLQMHYLVKNFTKETMF
QYGCVLTVLTYCVVALFFAIASGNFVEKLESEISSFVWPLDICGEDVKAAILKRAFYTSLVA
YITIIAFPIFSVIMFPVLGDQSDMFLCVRVFNEYFTKWSQIPISLYFYSFPVIAFSGIRLPGMLL
YAILITHIQMFLLNRRIEQISELSNQRRVFETLCSCIELQAKLKRLIRNVFQLVYIAMPIFILLG
AVSSVFVLFFVVNSLETASYFLVLRMGCFFGANVLVVFIFSQSGQSFSDETGRIFDTLVMCS
WYNWDKRNKKVLLMFLANSLEPMSITIAGITLDYKFALAMLRTSCSYALVLYQMKNZ
>TcasOr160
MSGKTKRITTKTIHLSNPYSSFKKVFSDFAYSKIMIFYTIATLAFHMLSLFLQIYYVATNYSV
ELICRYGPMMCLAIYVVTAKVVGVFYYKTFTMLENQCLFVLWKTCNSSPTTQRLILNKSL
KMNQKLHLALMSYFLLAIVMLPTWGDLNELFIFSQVYERYFKFWAPVLYYFYISTFLWCS
YYSFHLPGCILYLTLLLDVQIKLINDKITEIDQNFSQNEISETLRLCISHHIALKRWMSTLAK
MVNSVMPVFVLLGALSTVAVSFFVLNTLQNTTMILKIRLAILTVCNFVIVSTFAELGQIFSD
QNNSLFEHLIDCPWYLWNVKNRKILLMFMANCMKPKTFSWGGITLDYSFAISILKTSFSYA
LILFKLRGETIRNZ
>TcasOr165
MSDNTKKATTKSLDLTNPYSSLKKVFINFAYSKIMIVYTSATLIFHILSLMLEIYYLATNFSV
ELICRYGCMMCLITYMVTAKFFGMLFSNQFKFLEEQCLLDFWKAFNSGPTTQRLILKESSK
MNRKIHLALTFYVILAIIMLPIWEDVNDFFMFSQVYENYFANWAPVLYYFYISTFVWCSYY
SFHFAGVIMYLTLLLDLQFRLINDKITEIDQNSTQNEICGTLRLCISHHIALKRWMNKLANS
VDTAMPVFILLGALSTIAVSFFVLNTLQSTSVILKIRLATITVCNLIVVATFAELGQIFSDQNN
SLLEHLMDSPWYLWDVENRKTLLMFMANCMKPKTFSWGGITLDYSFALSIFKTSFSYALV
LYQLRGNTFZ
>TcasOr173
MSNVTFDEPFMFFKKVFFDFGYCTSIRFYHLFCFTFHICCQIIENYFFLTEYLSADFVTRYGC
PMIVIGYTIVCEFFLMKWEEPIKELLDERETIFWEIDSNSKPQILKYSSKVNRIYKFFLFWVV
ILAIFLLPFWGDLDETFFIIRIQKIYFGKWSTLFYTLYVSTLPFMVYSGIRFPIVTLYLIMQSH
LQILILSQKIGQISQNNNHMDDVSKFHDVGYQKKIRTSLHVCMCRHVTLKQWISKILQIVQ
KAIPVYFSLAIIVLVTVMFCILYNVESASTTTIFKIRLVLVGICGAVVLFTFSETGQLLSDDTS
QVFDTLAASPWHEWDPKNRKTLLMFLLNSLKPVKIYWGGFALDYQLGGSVIKTTFSYAL
VLFNLRKDZ
>TcasOr175
MRNFQDSDDPFIFIRKVFVGFGCSTIIMYYSRLIFIFHTLSLLLESYHVITNFSLDIITQYGSA
MSLMLYSITSQFLLICEQNLITEVVEECKSFFWTMDFLSFIKQTQILKDMTKIKRKMYLSWI
WFVVFGIALLPVWGDYNEMFLFPFIYQTYFGNWSPLFYYFHASSFPFLAYIAIRIPAFILYLT
LALHFQTLLLNQKILQIPQNKSGNQEDIFRNLCSCISHHVALKKFVTKTQQSIQKMIPVYFV
LAILCLVAVMYSCLNSLAMSTSNHFKVRGFFGGVCGVVVLYTFAEAGQLQADTTGEVFNT
LMQCSWYNWNNRNQKILLLFMVNSLKPSYIDWGGVIVGYSFGSSVIKTCYSYALVLYKL
KISKEQNVTFZ
>TcasOr199
MSMTRSKYFQDSDDPFSFIRKIFIDYGYSKKINYYNRVTFTFNTCSILLESYYMITNFSLDLF

VRYGGALSLMLYHVVTQFLVIAKQKSLEQLLEESKSYFWKADIFNSSVKNQILKSCNHMQ
RKFCLLWTPFVACGIVLLPVWGDFTESHIFPQVYKAYFGHWSPIFYYFCISSYPFAVYTSIRL
PAIALYLFLQAHFQIVLLNQKILQISKNNDLDETTIFENMEYQKTIYRNLRSCISQHVALQK
YITRILVSIQKAIPVYFCLAVLCLIAVIFFVLNNLNMSASNHFKARIFVSGVCGSLILYTFTEA
GQLLADTTGDIFNTLMQCPWYYWNIKNRTVFMIFMLHSLNPLKIDWGGFTLGYSFGGAVI
RTCCSYAVGLYNLRESKYZ
>TcasOr223
MTDFKDPFIMLRRIFIDVNSYKITKLCDFTVITFHSLVLCLQLYYMIRHFDVNLSIKYGPITA
FFLFMTVSAVLSGALSQDIFRAVAFFEKISWSLDVIRKEARIKLERKCQVINTCISCILLFSST
TMVINLPFCENQRYFFISNQVFEEYFGKWSVLLNVFYYSGVPYLGYHSVKPCFVFVYAILE
IQLQFSLIEEYLLQTYETDYLESGEHLEDTQYQREIGEALRRCITHHVQLKKLIDMMVDIV
LMYMPFFLVLGVFLLITCFAFIINFADTTTNTVKVQAFMFVVTALCNTVLFCWNGQQLIDV
TNSIFLTLGGAPWYHWNVENIKILLMFITNCTKNDSIVLAGICLDYKMFVSVFRISVSYALV
LFNLRKRSLVZ
>TcasOr209
MMPEFSDPFIMLRKMIFIKNHKIAKFCDFLIIAIYSSAFCLQIYYLCKNFSISLLIQYSPTLLC
YIFVIDAAVLFFYVEKNILEAITYYDEIGWSLSMIPKDAQTKLRKKCLIINICVSFILLLILSTL
TINLPYFGSQRELFICIQIYEEYFGNWAFVPHHFYFGVFPFIYYNSVKMWISFVYTILEAQL
QFILVEEYLLESNIINDFKGWKHLHDIRYQQEIGKSLRLCITQHIALKKLVKMIVNITMAAM
PSFLVLGVLLLISSFAFILNFADTMTNILKIRVLMFVACIVCITTLLCWTGQQVIETTSDLFDS
LVGAPWYLWNRENIQIFLMFLVNCTKNESLVLAGICLDYRLFVSMLRISVSYALVLFNLRK
SSITZ
>TcasOr214
MAEFIDPFLMLRALVSVKFNDYTSLKLCNILLITIYSLIHCLLIHYMFKNLDINLAVRYVPMI
MFLTLVIVGAIFSVAIEKDILEAQVFLFKANWSLEMIRKDAQLKLERKCRIINICILCVLLLIF
ATITINAPLFGSQRELFICIQVFEEYFGKWSFIPYYFYFAAFPFLYYDFLKLWMSFVYAVLEV
QLQLTLVEEYLFETYQINSSKEWKNLQDTHYQQQIKKSLRLCITHHIALKKFVKMTVDLTI
KVMPFYLTIGVLILISFFSFIINFADSMSNILKIRIFMFSASIVSITVLLSWIGQQLVDVTSGIFW
SLVGAPWYFWNLENVKTLLIFLMNCTKNESIVLAGICIDYSLGISILRLSVSYALGLFNLRK
SSLDZ
>TcasOr238
MMSEFNDPFIVWRMIFMINFKKHKITKFCEIVLIVIFSLIHCLLLYYMFTNFSVNLLIRYGPT
TLFGIFIIAVTIFSVALEKELSGGIDILDEICWPFNMIGKEAQLKLERKCRMRNMCTAFVVLII
LTTIIVSYPCFGDQRDFFICIKVFEEYFGEWWSIPYYFYFITIPFFCYNYHKLCFTFVYAVLET
ELQFFLIEEYLLETFKMGYLKRWKYLENTQYQQELGKSLRFTIAHHNALKKMVKAIVNV
TVNGMPLFLLLGFLLYISCFTFVINLADSMTNILKIRIFVCGASCVSVTVLLCWNGQQIIDV
TNSIFSTLTGAPWYFWDVDNVKILLIFITNCTKNDSITMAGICLDYKLFASLLRISFSYALVL
FNLRKSSLSZ
>TcasOr259
MMPEFNDPFLVWRMIFTINFKKFKITKFCEIVLIVIYALVHCLLLYYTFTNFSANLLIRYGPV
MIFYIFMIAATTFSIALEAELSEVITFLDEICWPLNMIAEDAQVKLQRKCRIINMCIAFLVLII
LSAIIVNYPFFGDQRDFFICVRVFEEYFGEWSFIPYYFYFAASPFFYYNYFKLCFTFVYAVLE
AGLQFFLIEGYLLQTYKVDYLKRWKCLKDNRYQQELGKSLRLCIVHHIALKKLVKMIVNL
TVNGMPIFLLLGSLLYISCFTFTINLVNSLTNILKTRIILMGASCVGVTVLLCWNGQQIIDVT

SSIFTTLVGAPWYFWNLDNIKILLMFITNCTKNDKIVLAGICLDYKLFASILRISFSYALVLF
NLRKASVSZ
>TcasOr202
MVEFKDPVIMLKTIFLVNVKEMTKFSQVFLAIFTFYSLVHCVQMYYLYKNFDVNLLIKYAP
ATTATLFVSNTKLLSSSLNIMPIFSVVSETKLLRITTFIDKTFWPLDSIRKEARIKLERKCRAI
NISIYCILLLLSVAVFSNFPCFGRQDDFFLCIKIFKEYFGQWSSIPNYIYFTLFPIFCYPYFRIAF
SFVYAILETQLQFSLIEEYLFEVYQMVDLNWKYLQDPRYQQEIGKSLQLCIEHHTALKKLI
HSIVNITLTGMPIFLLFGIGLFVSCFVFIINFGDTMTMILKLKTPLLLYVATMLSMTLLMCWN
GQQVIDVTSRIFYTLVRAPFYFWNLYNMKVLLMFITNCTRNENIVLAGICLDYTLSVSILRI
SVFYTLGLLELRNHSFDZ
>TcasOr224FIX
MTEAGDPFIMLRWILLMDVSNNKITKYCNVFLTTIYSVVLCLQIYYIFKNYDTNLLIKYGPI
TISLLFMITVAVISLIMQKEIFKTVTFIRETCWPLNIIQKSGQIKAERKCRTINFYITSTFLLFLS
AIIIHYPCFGSQRDFIICIEMFEEYFGEWSSVLYYLYLIGVHFLYYRLFQTCYMFVYGMLEA
HLQFFFLGEYLLETYQTDCLKRCKYLQDTRYQQEIGQSLRFCIKHHIALKKLVKMALNLA
FIGMPFFLVFGVLLLISCFTFIINFADTMSTILKIRIFMFASNTVCIAILLCWIGQQLIDVTSDIF
VTLCGAPWYNWNLDNIKLLLMFIMNCTKNESIVLAGIRADYQLFVSLLRISASYALVLLKL
RKCSLVZ
>TcasOr217
MVEFKDPFIVLRKIFFIKFNNCKLTLFLNISIIVFFSLVLCLQICYLMKNFNLNLLFRYGPVTV
LFTLVTVTAVLSLTLEREIFMAITFFFKFCWSLNIIRNDAQITLKRKCRCVNIGLLCILLIILIAI
VIGFPCFGSQKDFFICLEVFEEYFGEWSFIPYYFYFAASPFLCYHFLRICFTFVYAILEAQLQ
YLIIAEYLFEIYQTNPSKRWKYLQDTRYQQQIGKSLRLSIVHHVVLKKFLKRTLHLTKIGMP
FFLVLGILLLTSSFAFIMNLGDTMSNILKIRIFLFTTSVLCITILLCWIGQQLIDVTSQIFVSLSG
APWYFWNLENIKILLMFLTNCTKNESIILAGICLDYKLFVSVARLTVSYAVVLFKLHKSSLV
Z
>TcasOr254FIX
MDKLDDPFIVLQRLIFIEAKNcklaRFFDVLLIVLDSLAHCLQIYYMCQNFAFSLLIRYGPVFI
FFLLVIVTAVISVGLEKEIIETYAVYCKTCWPLNIVKKQTQIKLKKKCQIINRGILCSLVLFLA
AVISLFTCFGSQREFSICVEVFDEYFGEWSFFIQHFYFTVAPLLYYHFFRVCYLFVYALLQA
HLQYFLIEEYLFETYQTNDLKGWRHLKDTRYQQKIGKSLLLCITHHIALKKFVKMTLDLV
LIGMPFFLVLGVLLLISSFAFIINFADTMSTILKIRILFFAASSVCLTMTFCWIGQQLINATSEIF
WSLGGAPFYFWNRENSKILLMFLMNCTNNDSVVLAGICLNYRLFLSVVRLTVSYALVLFN
LHKSGLVZ
>TcasOr222
MDKRDDPFIILRKMIFIEAKNCKIAKFCDAFLILFYSLVQLLDIYYMSKNFSISLLIRYSPITI
MYLLIIIAAVISVGLDKEIIEAYTVCCKIRWPMNVVKKQTQIKLKKKCQIINAGLSCTVPLFL
VTIISTFPYFGSERDLFICVEVFEAYFGEWSFIPYYFCFAASPFFYYHFFRITFVLVYAFLHAQ
LQYLLIEEYLFETYETDEAKGWKYLQDTRYQQEIGKSLQLCISQHIALKQFVKKTVDLVLI
GMPFFLVFGVLLLTSLLAFITNFEDITSNILKIRILLAAGCSLCITIVFCWIGQQLINVTSDIFFS
LGGASWYFWNRDNMKTLLMFLINCTENESVVFAGICLNYELFLSVVRLTVSYTLVLYNLQ
KQZ
>TcasOr208
MEKLDDPFITLRKMVFIEAKNCKIARFCDVLLIVLYSLAQCLHLYYMCQNFNLNLLIRYGP

ILISCLLVIVTAVISVGLDKEIFEVYTVCWKISWPLNFLRKDAQTKLRRKCQIINRGILCSAL
LFLTTVISTFPCFGSVRDFFICVEVYEKYFGEWSFIPYYFYFAAAPFLYYHFFRVCYVFAYAF
LHAQLQYFLIEEYLLETYQTNDLKGWKYLQDTRYQQEIGKSLLLCITHHIALKKYVKISQN
LVLIGMPFFLVLGVLLLINSFGFITNFGDTMSNILKIRILIFVACGVSITIVMCWIGQQLIDVTS
EIFVTLGGAPWYFWNRDNNNILLMFLTNCTKNESFILAGICVNYQLFFSIVRLTVSYTLVLY
NLRESGFIZ
>TcasOr206FIX
MWNNNPFIVIRTIFLDINNYKIVKFCYVSLTVFYSLVHCLQFYYIIKNFNLNLIIRYGFITSLL
SYVLAAGILSLVVEKRIRKTQIFFDEIGWSLNIVGKDAEMKLEKKCKLINISIYAIMLLLIITL
LVNLPFVGSQRDLFLSIQVFEEYFGKWSEILDRLYFALAPFLSYHGARLSFTCIYAIMQVQV
QFSLIGEYLFETYQVDDSKSWKYLQDTRYQHDIGESLRLCVEHHVALKKSIKMMVDIALT
CLPFLVLLGLCTLISCLAFIMNFWDTMDNILKLRIFMWAAWIVLITIMFCRSGQQLIDATSDI
FFTLGGAPWYYWNLDNIKILLTFMANSTKNDSISLAGICLDYPLFVSVANTTVSYALVLYN
LRESSLDSSNKKZ
>TcasOr263
MWNNNPFIVIRTIFLDINNYKIVKFCYVSLTVFYSLVHCLQFYYIIKNFNLNLIIRYGFITSLL
SYVLAAGILSLVVEKRIRKTQIFFDEIGWSLNIVGKDAEMKLEKKCKLINISIYAIMLFLVIT
LLVNLPFVGSQRDLFLSIQVFEEYFGKWSEILDRLYFTLAPFLSYHGARLSFTCIYAILQVQV
QFSLIGEYLFETYQVDDSKSWKYLQDTRYQHDIGESLRLCVEHHVALKKSIKMMVDVALT
CLPFLVLLGLSTLISCLAFIMNFWDTMDNILKLRIFMWAAWIVLITIMFCRSGQQLIDATSDI
FFTLGGAPWYYWNLDNIKILLTFMANSTKNDSISLAGICLDYPLFVSVANTTVSYALVLYN
LRESSLDSSNKKZ
>TcasOr200FIX
MSIQNTKKTLNGKEKHPDFIKFCFTDSNDSFLILKRVYIDFCYHKITKTCNFLLVISQLFFYL
IQIHFLLSRFSLELLARYSTIMMITTVALFGLILSFYLEEDIHELYKILTEIAWPLDKASKKDQ
QDLRQKSRRINSLNLYFLGFLAFMIVIFLPIFGDEENLFLCIQVFDEYFGDRAFIFYNLYFIGF
PFLIYFSVQLCFMFLYAILHLHVQINLINHHICEMGASFELLSDWKKLHSVVYQSAISQLLC
QCIRQDIALKRFIVKLNETVQLGLPFFLPVSGLCGISVIFFLLNYMCTMSLVLWLRVSAFFIC
LVFVALIFSVSGQLLIDETGKIFDTLVKCPWHIWNVRNRKIYLICLTHCVRPNCISYAGITLN
RIFLITVFYKTVSNAFILYQVRNSZ
>TcasOr231
MEVTGKKVTSGDPFITLKKLYIDLGYHKVTKLVNVFFIVFYGFVYLLQIYYLIVHFNFEIIA
KYSTILLLSTYLFNVMIFSIIYEKCILDAYKTFSQIAWPCDNASKPLQIIILQRSKTIKYLNYFF
LGFIFFMACINWPWLGDQNDFLLCIQVFKKYFGSWSPLFLLFYYLGFPIIGYSAARIFFIILY
GVLHLELQIRLITELFCKISRNATLEDVRNAKYQRDVYWTLREGIRHDTALKKVLFDLNKE
VKHGIPVFLIVTLLCSVSIFFFAITYLESMGFGIQIRTIAFVGAVIFVLFSYSLLGQHLINQTSL
LFDQLYECPWYTWNVKNRAIYLNFMLNTVRPIKITYAGICIDSRFFLSITRIILSNAFMLYQL
RNSZ
>TcasOr315
MTLVRKLQAAATNAFEIRIKDDILAELFNWPFLVLDSKWSTKFAVFLTVYCVFETLACALV
YSTLDVNMMGTYAIVIARFATTFCSFFSFFTKRKQYFEIINENFPHFWPLQSLGKSTFNRIK
MRASSVKFYSFLNVVVMLIGAVILISFTQDESEVYLSVKIYKDYVNKWTTGFVMFFYVSFI
YIGLVVAAISFVLTYTAFHLIFQCFLLNQKLKQINDSIVENEQKQAKFDEKYQSFIYKELISC
VKLHQRLILFGKRINHLVYAPLLVYIFGGIVVGVALIYYLKSSVQHIFTSLILLLIALINSTTF

VINGQMLENEAENIYISLTNLPWYSLNVQNRRVVYVMLMQSQKIIHMSASGLVSLNYQLTI
VFFRCIYTGMTFLVNVGLZ
>TcasOr316
MTLMRKLQTAIRNLFEIQIKDDILAELLDWPTLVLFSKWPKNFAIFSTIYCVFDTLVCTLVY
STLDVEMLGKYAIFIAKSTIALCSFFSFFAKRKQYHKIINENFPHFWQLQSMGESTFDQMK
KIATTVKFYSCLSVVAMLIGAVILILFTEDESEIYLSVKIYKDYVNKWTTGYIMFFYASFLYI
GIVTAAVVFGLTYIVFHLIFQCFLLNQKLKLINSYIVKNGQKLVKLEERNQNFIYKELISCVK
LHQRLIYFSNQINDLLYAPIFMYTFSGIVVGVALIYFLKTSIQYILTSLVLSIVSLIITTTFVING
QLLEDETENIIISLTNLPWYSLNVQNRRVVYVMLMQSQKIIHMSASGIVSLNYQLTIVLFRC
IYTAMTFLVNMGLZ
>TcasOr272
MTRTPTDDTTFDLDNFMKNDSMKLVRVIAYDTLKFKITKLILFITFLVHFSTTLIQVYFVCV
DFNVYFFVKYAPAMFGSLFVMVSIIALFVTAETDMVVRVFRKAQLRKLTVEDGPSFHVFQ
KECKIFTVFFVLNLIIALFSGYLHALPDDDDREIFYAFAFFEDYCSEWKDFCSFLYRITFLPV
AYVMYVPINVFVYAAIHLKSQIYYLKEHLIQINEGYDISNNNDLFYDENYQRIIREKMIYLY
KIHVKLFLAALDIRKLIRGFIALFAIVGCLLGISILYFVMLFQGNLFDKFGRLSTLTVVAFNSF
AAVIISGQMIESSSSDDVDAIIYNCNWYDWNEENKRFFLLIRMATMHPFKLQFSQNYAVNY
QLGVAILKAMYSAFSLLKAIKNDFZ
>TcasOr302
MDFSEPAPFHADALWIVRLLAVDILNKPFVRFLMFTVLLFHVTVLLIQIYFICFVQTFGEFV
KYSAIFCAMFYVNLSMITLLYEKNMVKHILEKCKLWALNSIDDKIYQDIRREALFGTVFVI
TNLILVFVTTITFIIPAPLDRDVFFVVYFFESYLDQTWGKILTLVYRATFLLLGFIVVTCAHQL
FYTIQQFKFQIYLLKEHVENITNIEFFDEDDSLLLKNPSYQREVKRRVRFCIKRHIQILNGAD
VGMKLVKKWIPLYSIAGILFFVSIVFSCISFDGSLEDVYLRVGSLGVVSVTTFCSIIFVGESIA
TQSDVLAQVNTFIRWCSLNRENLQLFAIMGIMSKEPYYVKFSQNLAINYALGIKVVKLVYS
FLCFLIQCKGVLYZ
>TcasOr300
MIGLTNGDYSPRPSMEGDCLKILKFFAVDIFNPKIVRFFLWIMLLYHVVFTLVTAYFMLYVL
SNSEIIGYTPAFLGNFYPMLCVWSVLFISRLIYVKEDMPLWAIDTAGAKVQASIKRKIFLYT
AFGIFNLVLSLSAGSFYLKNVSEDVNVFLALRIFRDYFPNYYQVLDLIYRLIYFCFSYLMVA
PSYLLIYYILHVRIQAIIFAAYVAHIDGHSDYGTDIDLFDNEEFQSEVERRFKFCIKRQIEFLL
MESKKLSQISNLIAAFSLAGCLFGISIIFHLFTGQLIQEYYFRIGLTSLAAIATFSAFIYTGQST
EVQIELVDNAIDNLCWYNFNRSNKLLYLIAKADLARVRKIKFSGQWAVNYDLGFAIVKGIY
SIISVVVSMWZ
>TcasOr276
MTMQFIVKRATRGIFHDLRVLKFISSDIFDIKIMKLCLFITFLIHLTACAITIHAFMFNNFSRR
EFISCAPVLFGCFYGLLGLGTILFKPSMTRTLMLELKAWDITAADDAVSSRIKFEINVITVFC
LVNYLLALVASFFYYMSFYGDEEIFYLIRFLEDHCPNHKRVLIKLYKISFVLLGYVMVVHA
CQVLYATQHVRFQLILCAHFMANVTKQAKNIKDEHLPDDNNYQNMIRERLKFCIIRHQEIR
RFYFDKLEEMGNLIGGFALLGCFLGISFAMHMLTSEFLRYHFARTVSSIIAGVTTFATVIAA
GQSVETEVDISTRVVKEVKWYTFNESNKRSYMLMLLNSMQTYKIKFSENYSINYELGLSI
VRGVFSIVSVVVQLDYZ
>TcasOr281
MDYSEKSLIQGDCLKLLKVISSDIFQPKLVKLILLIVFGVHLVVDLLTLRALLVNELDFKEFI

FYGPVFFGSFYGMMALLTLVLKDDFISNLKQEFRLWPLDCAGDEIYSQIKFENKIIKIFVVF
NCIVTFIGSYLYFLPLDSDNETFYAVRFIEENYPDHRNLLHGLYRSTFLIFGYAMTVHVYQV
IYNSQHLRYQIIIFTEYVASIGNPDKRKENELFYDKGFQKVVYERLKFCIMRHQEFLVISNK
KVGDMRVFIVGYSLCGCLLGISLTFYIFSGKFYREHFPRVSVACVGAVTTFWAVITAGQAIE
SEYDSLLSTLLGKIEWYYFNDSNKKNYLIMLINLMQPWKIKFSEEYAVNYELGLAIVRAIY
SIVSVIASMHFEAZ
>TcasOr283
MLKFEPKPTTKDELLWVVRTIYVDLFRNKLIQFALKMLFYGSIIMAIYQGVLFLYEFEIHYF
VKYSSMYCFTCFILLAAYSVPIIAEVATTAFTTIKCWKIDSGGALVENKIKQEAHFTNIITAIN
CIFGLMVLVLFIVPFEDDNDFYFLFIAFEKYFPQWQQLLKWGFKAFFPCITILLQAPFYIVIY
ACLRIKFELYMWMEFLKNLNIVYEKSDICELVHDSEYQTEISKRLRFCIERQEHIYRSLLYG
KKYVQQLDIYIFAYAILGSLGGISIIFVCISFEGNFFQGTYLRLSALTFVTLTTFMHVIWAGQS
VETTSSDSYDILKQCDWFLWNLKNRKTYLMCLNYTQRPLKAQFTQNVSINYVLGFSVVR
TVYSTLTALNSLRKASKZ
>TcasOr286
MSVQKVSPDNLWLSAKICLHIFQYKAIKIILKILSLSIIILTCIQTFLYLKRFDSAYFMKYLPV
YAGSLFILASIFCIEHISHVILSTVEEFEFWDYADSKPEIRNWIKWEALYINTFMVVDAFVAY
LSGIFHAIPLDEDYEIFYPLPIFQEFFPDWTNVLGWLYRSSFLIVPVVMTAPSLMIIYFTSRLR
FQMFLFMDILENISEGYDISEANDLIENSTYQKEIKERLKTCIKRHNEFLSAGGQVMKNGQ
LFILIMSAAGVILGVSIIFFLFSFEGSFEKRYPRLVTLVISTGLTFTHVIIAGQLVENIATRLYEI
LHFMDWHSWNQENRKILLIFMHNAQQELQIKFLDEVAVNYQLGISIGKAVYSMISVLSSFK
NLEESYNZ
>TcasOr289FIX
MLNFEEKLDENIFKNDVLWLSRKLCYDYYNTKIVKIILFVLSIRVAILTLLQTLLFLQRFDG
RYIIKYAPVYAGSFLIFLSVKHIPLSLMLINSFKTITLWRIDSCGPEIERKIKKHAMWTNICLIS
CTVVGLVSGIFHAMPLEDDEELFYPLAMFEEFVPQWKNLLSWMYRLSFLTVPFSMPIPVYI
AIYVTTKSYFQILLFGHFLENLNTGFDTTLNHLLIYNNKYQNTIKKRLVFCIKRHAYFSRA
MNELIKKTYVTIATFSIMGVILSISVIAFLFSFQGNFENRYIRVTTLVFTIVTVSTHILYAGQLI
EDAAFQVYTTLKTVDWNSWNLENRKLYLVCLQNAQIIFSIKFTQDVSINYRLGFSMAKDI
YSMISVMSKLRNVDYSKIZ
>TcasOr293
MISFEENIDHDIYKDDVLWIMRKISIDYFHYKVVKILLTLLSIGITILTVIQTFLFLERFEGRYF
VKYAPAYIATFLMVVAMQYISFSIRLAALIKRITFWTINSARVETERKIKKHAMYTNIFFLGT
VIMGVISALFHIMPLDDDNELFFPLILFEEFVPNWKNFFSWMYRLNFLAVPFTLPIPIYITTY
HLIKSYYQILLYLDFLKNINTGFDTTSSNNIESAEYQQVTRDRLVFCIKRHSYFYTQMREVN
RKMSKFIAIFALISVLLGGSVLTFLFSFQGTFENRYPRIVTLILTAGCIFAHVIYAGQLIEEAAT
QVCENLKVLDWYHWNCHNRKLYLIFLQNTQKPYKTQFSQNVSINYELGLSIIKTVYSLISV
LRNLQDINZ
>TcasOr294
MIVYEEKIDQDVNGNDILWVMRKVCIDCFQYKIVKILLLLLAIFIAILTLVQGFRFLERFDG
PYFIKYAPAFIRTFVILVAIAFISFGMETAIYVKDTTFWLIDSAGLESEKRIKKHAMYTNIFFV
SYIVVGVISAIFHIIPLDDDNDVLYPLALFEEYVPDWKNLFSSIYRFTFLTVPFTLGIPLYTAI
YIITTVYYQILLFVVYVKNINTDLDVENVKYQETIEKRLIFCIQRHSSFLKRMKESNRKMSA
TVLVFSIVGILLGASVLMFLFSFHMSFKIWYYRIITMVLPTGAIFIHIIFIGQSLENAISQLEAN

LKMVEWYHWNIPNRKLYLIFLINTQEQKGVKFSQNVSVNYKLGVSIAKAVYSLISLMSNL
RSIDZ
>TcasOr287
MISFEEKHLTNVRESDVLWLGRIMSLEIVQYKPMRFILNIIAVSIIALTLIQTYLFLQKFDGLY
LIKYASVYTASLFILFSIIAAPFLTKFSTEALNNLEYWPIESAGAQIEKQIQREAIYINTFFVVN
MVVSLISGVAHMIPLDDDKELFYPLAIFEEFAPKWKNWLEWGYRLSFLVVPVVMLNSSYV
GIYTLSNFRFQISLFNHLLKNINFPLNDNEQTIELMDDQKYQNEINKRLKFCIKRQTHLYKV
AHYVTGKVKHLSFFVAILTILLLIAVIVFLFSFQGTFENRYFRIITLVLTAGNTFIHVIIMGNRI
EEETEKIFENLKSLNWSSWNLQNRQVYLIFLHNNEEHFKVPISENASVNYELGISMAKTIC
SMVSVMSQLKNIDYSKNZ
>TcasOr292
MQPNLQKNDILWLIRKLTFDLFQLKITKMFLIITSVSIILLTIIQTFLFLKKFNGYYFIMYSAV
YTGSLFILVSSLSVLPISKLIKTAWVKFSFWEINSATPKIERKIRKEIFYINCVVFFNTIVAIISGI
FHAIPLQDDEELFYPLAIFETYTPEWKDWFSGIYRASFLPMPIIMVAPAYTVVYLCAHMRF
QFCLLLHFLENINPDNENISDKKYQAQIKERLHFCIKRHIHLFSKSRPVLEDLKKFVFVLTL
CGTIFCISIIIFHFSFQGTYEGRYPRIITIIIAASITFFLSILPGQLIENTSSEIFEVLRNTNWVSW
NEQNKKLFIILLLNTRQIYKIKITENVSLNYELGVTMAKAMYSMISVMKQLZ
>TcasOr304
MRCYNHDVLWLLRFWCCDIFQYKLLKFVIFVMLVANTVLMVLQGYHFLINFNSLYFISYS
PYWFGSFFIILSLTTCLTISNIGPEGIKSATLWQIKSTDPKLINRIKFQVKLITAYIIVNTVIALIA
GLAHTFPSKNAEEICYVVYKIIEIYVPKWKTELCWMYKASYIVMALALPATCNQVVYGATH
IRFQFYLVLDWIRNNIIESGCDDLKLPNDKDFQHKITKNIIVIVKRYTEFHRTFQAVNQRIAI
YILLYAVIGGLLGISILMFYFKFSDTLVISDYLHAGTLTVAAITTFMATVTSGQKLEDIFEELL
YTWCSVPWYLFNKWNKQIYLMIMVNIKPINFRFTENSSVNYNLGAAIAKTIYSMLSLLTQ
MSDKDVSTLZ
>TcasOr153
MAPNKSSKKDFLDMCRKYMHGSGLGPTSPKIKRFISLWLLFPVSLLLDVLVIYDFHFLDN
DIFKTAELLESVSSFGQLPIRKFILTYHSKLIQNLLEDRKKFWSYEMFGETYGKFLRRKMVL
ATRLIQTMIFFGASVATLMFVSTLADDRKTVPLECWIPEFKHSTHVVLVMQFCSLCEIIYLV
GAVDCLYVLTCVDIKIQFLLLQKKLKTIQVGVKPMEECLNELTICVKHHNLLLRSHKSLNR
IFSEYFFVQYFVSVLAACVQLYILMYITASLEDIMKSIVYLSAVVFQVAIFFMPASDIEEEAE
QFAVEIYNVNWECTSGTKFRKQLLFMLMKAQKPLYMLGGGMIHANRNEYIVLFRLAFSIS
TLLGGMNENGRTDKZ
>TcasOr133
MEYLKYCQTYIKGTGLASNSSLFRNILAKYFFLPPCFLIIAFSIHELWDTNNNDVSLVTEVL
ECVASYTQLIIRKYIVFTQSDLMVEIINDCGKLWPFDMFGSELGKKFKQQMKTCWTLVKF
LVVCGFATFFLMCISARAAERDNSLPFLCWVPDFPYATELLFFLQFMLLLELLYYVLATDAF
YILICMDIQIQFEMMGKMLKSIKFGEISEKECWDKLVELAKQHDRMLHQKLNQVYSKYY
VVQYFMTVGSMTVQAYNLKYRMVNIQTALKSIVYTFSLMFQCGYYFFPASNIEIEAENFS
TEIYFLNWQNIGNIKIRKHILFMLMKSQENLAMMGEGMVHVNRNECLMMFRLAFTIATL
LDGLNQVZ
>TcasOr136
MEYLKYCQSYIIGSGLASNSPVFRQFLARYLFFPLSLILVSLSIYIIKDANNDIYFLTEVMESL
ASYTQLLIRKYMIFTQSKLMVEIINDCENLWSLELFGPELGKKFKQQMKNCWTFVNVLVT

SGFSTVLLICITTLTDKEKSLPFVCWIPGFPHATELIFLTQFVLLMNGLYYIKLTDAFYLLVC
MDIQIQFKMMGKMLKTIHFGLLSEKESWEKLVELAKHHNKILHKKLNKVYSKYYVVQY
VMSVTAMTAQAYTLKYIEVNIQLALKSIMYTCSLLLQGALYFFPASNIEIEAENFSTEIYFLN
WQDHGDVKIRKHILFMLLKSQENLEMMGEGMMHINRNEYLMMFRLSFTIATLLGGLNQ
LZ
>TcasOr138
MGASIHYFNYFYGHSQSSKFPTSSKSLKARIFNYVLYPISCTILAFMLYNFRYMNDNIFKIA
RNCQSITSFGQIFTRHFIYAQHSFLLENLLKNTAKNWKYDPKNPLFGHKTWTRVILVKSLKI
MSFFVVTFIIILPFFCQDIDLPQACWVPGSGHKMQLLIYFLQSVCLLEPMILLDMVDSVFLL
TGVELEIQFILLRKAIKNVQISEQENQNFCMGKLKLYASYHQFLLDQHTVLKQAFSSFFFL
QYLVSVQGLCVEIFVIKQASTIEQIFFSATYIVANAMFLSFAFLAASYLEIEAEALPHAIYSID
WYNGDEKLGKHVLFMLMRAQKPLILTGAGMFAVTRNALLQVYRLAFSISTLLKQVZ
>TcasOr139
MSDHFRYVSTIFKLSGYLPSLKTLKTKILKYVFSPIMFTLITFIIYNFRYMHHDIFEIARTCEA
LSTHGHVFGRKLAVLKHANLIEQVINDRNFFWSYEKFGEKLGQRFRQKLFFRDYVMKSLS
AMSVLMLAYFYLTPVFVRSVNLPQASWVPQFSHATALVYFCQVVCLSESIPVAVIIDGTFLL
MGAELEIQFNLLKKTLKAVEIGQNSGQKHEEKCLKQLKICASYHDFLLKEHVKMKTIFSEF
FLLQYLLSIEGLCIELFVVNKAQTWGQFALGAIYVVGIIMQSSFTFLSASNLEIEAESLADSI
YDVDWYNAKDPRIRKHILFMLMKAQEPLRLTGAGLFDVNRNMLLQIMRTGFSISTIMRQF
Z
>TcasOr144
MAFNESQDNFKLCFIAFNLSGLGPSSKPFLKILSYVLYPWLCLLFVLVCVNIVFKHSNIWDI
GEVTSSISIAVMMVVRKTILIKYSSVFAEIIELHSRFWDYGLFGKATETKIRKKVDFFKFILK
CYIVSGITATSTRSIVPIFDKNLTMPQDCWIPGNNSIVKHIIYAFQVIFYAESISYFTFFDGFLLI
VTANLQAQFILLQKATGSINFETDSEETAWKKLVKCCEHHKFLISVHKKLNTLYSYFYLVT
YFLVITMGCVSLFVIFDKSSTFAQLLESAITMVVLNVMIAMICICSSEIEIEAEKLLTQIYEVN
WYETPNLKIRKFILFWLMQAQVSVETKGAGLLVVNRSLMLQVQRFSYSVSTLLKGMNEZ
>TcasOr146
MKYIREVENIKAGIFSSCGIVAEEQRVEMSEKNQQDYFKIPDKCYNWSGVRVSSNALTKFV
SLYILYPLMLILYFMIIYNIRFKKNISDITEVFISISTFTIITFRKTLLIRNGSIYEDILKKQSHYW
KYYMFGKPTEMKLRKSMEFCVMVIKFLIASTTGSIIFHSIISPIIEGKIVLPQPCWVPNNDPV
ANDIIFALENIFYMEGTNYLVVFDGLYLLMTANLKTQMILLRKAVASINFREDEKTTWAKL
KEYCEYHKFLLRIHGKINKIYSAFFLMTYVFTIMGTCTPLFVIFYEEADMVLLGKSVFIALI
LNTLLVMTFIPAGELEIEAEKLSFEIYSINWYETKNLKIRKFVLFWIMQTQIPVQMSGGGML
IVNRPLVLQVQRIAYSLTSFLAGLSZ
>TcasOr154
MSNNDETDYFKIPLKFYNWSGVRITSKKIPKIISVYILYPMMIVLYGLMIINIRFNIDSLAKFI
EVGISVSTMTIIALRKTLLIKNGSVYEDLLETQKLYWAYNRFGQAFESTQRKRMYFCLLVT
KFIIGYSCVSLSYHSIIAPIIMHEMILPQPCWNPGNTTIGRNIIFFVENIFYIEGTSNFVIFDCLY
FLLATNLRIQFALLRKELNSIDFKEDSEEKCFARLVKCSQYHKLLLSVHRKINTIYSAFFLLT
YVFTITSTCTLMFVVFYMEADAALLGKSIFIVIILNTLLVMTFIPAGELEIEGEKLAMDIYFM
NWYETSSLKIRKFILFWLMRAQIPLQMTGGGMLVVNRPLVLQAERVSYSLASFLANLZ
>TcasOr113
MLDAWERLTFYPFKWISLGGLHPQNDRLVAKFLFLYNFAGFGTILGLAITQIYLSYENIYYT

IDSILTIVLYLHIASKYVNLHLHKDTLAMLIQERSKFWPIDTFEMTVRQKCVRILTKSLTIIKS
YLGYSLLVVISFLIQPIITGQLPVFMYVPRGTYYIFFVIFMTITPGIMSSIWGVDTLFFSITTPV
SIQFKLLAHKFETIDLKMDSKRVRHEFRKLVDYHNFLINYCQNINRMSSGIFLTQYLVAIAT
SCMQLFITSQPEFGLLNKIKCLTYFIMQIIETGIYCFTAQLISESSENVGNAVYKAPWYDFNC
GTRRDIALVIVRSQKKVVFNGLGLVWIKMETFTKIFKTALSFYTYLNTMVYQNZ
>TcasOr115
MSNKQLDPTYYILKIFKAAGVHPDVKVTPFLVFFFWMNCTIVSAVIVLATIGAIFGALDNDI
NTVVECLQSTFIYIHILGKHFVLFYSKPILSHLLAQRTHFLQLETFDLSTIEQFQQLLKKTSK
FVNTFMFCTSCVVFSFYMQPYLTHGDLPVSVYVPDGWYYYIHFGFWPLAPCIVASIYGSD
ALFCAISVPVIVQFRLLARKIQNWKIENTKLNNQKSRKIFKKNLKELVDHQNFLFEYCNEM
NKFNNGIFLNQFLLSVGIICVQLFVVSQKGFKLPNKIKCVGYSFMEIIETAIFCFNAELISDA
SEDVGNAAYDSLWYESDDPEVRHAITLIIARSQNRIVFSGFGFVWINLKTFTQIFKTALSFY
SYLHNVVLNZ
>TcasOr116
MPETLSFYPELMLKSAGLHPYTKLKIVKFFYHHYLNLFFFIFLLFLAILEVGVSVKCDIYRA
IEALSSVLFMTLTLFRYVVNYRNKPSLAWLLEKRSNFWLLEHFEGQIRTDCAKIMHTSSNFI
RNYKNYAIVLAAVFYVQPFIFHELQMKIYVPEGWFYYLYLVYWYMTPPLFVSVYGVTSM
FCAICIPVTIQFKLLAHRIQNLDFKSEKFQRDLKHLVDYHNFLIDYCTRINRYSNGVLLFEFF
ITISVCCILIFIAANDYPFVDKIKYAGFIVSQFLDTAIFCYNCELISDASENVGKAAYDSLWYE
SESKIRRSLILIIVRAQKKVTFSGYGLVRINMMTFTQVFKATLSFTSYLNTVTVDEKMLNNZ
>TcasOr126FIX
MYNNKDSLLYDPLRFFGFIGFHPIFNSLILKISFYSTTTLGLFIYTMAIIGIVKPEETNSFFTLE
CLQTCILLSHTIGKQVNYYMNSNKIVKFLQMTTEFWEFETFQGTIHPESNFLFHTVRKMIR
YYFLVTTFGFIFFLINPPVCYVPEGWELFLRIVRALTFWSYYTSTMATDAFFVACGTLLLIQF
KLLGHKFKNLDTQSQEKWNNLRQNVKHQIFLHSCKLLNQIFAVVFLIQFLNSIAALAISIFIF
SKPGSWNNRFKTLFYLVVVLFENAFYCVPAELVSAEALKICDQLFASKWYESNVAQFRKS
LVIVLCCTQKVIKFSSFGLVEMNLQTFVLISKTALSFYAFLNQLKRZ
>TcasOr127
MSPRTLEMIADLPKDIITDSLKMLRLGRHHPTGSIWWTIFFIPVNTFFCSLLIILSIVGIVRYH
EDDVFLAVDCLGTCTLMLHAISKQIFLHAQKNAINVLLKMKSQFWNLDDFDGEISKECEM
ILTSGKIAVRIYFSFTCAAATFYFLQPFTAHHLPSDCYVPEGWFPFLAISYMYLIPTLVPSVV
GLDALFWALGLSLAVQFKLLAQKFKLLGTCHENETAILWNQLKELINYHRFLIDFCKKLN
KLFSFIFFVQSFITITSASVAVFIVMQPGNLSTRVKCLLTFVSYILEMAFYCLPAEMSVNAAID
VADSVYNSKWFRIKSTEFKKCLILIIGRAQIPFTFSGFGLIHINIRMFQLVCKTTFTFYTYLNT
VQNRQZ
>TcasOr117
MDKNLDPDDLSAYPLKFLWYGRLHPGLNPWWTKILVPVNVSVAFLYLVLAIKGIFSSYNH
DTFFTAECVQTCILVVHAIGKFSNFLVHKNSLLRLVAKKSQFWKLESFDGDLYNECVWIST
FVKKITRFYYFLNLFVLISFDLQPFTTGYLPTGCYVPEGWFNFLTGLLWYLSCAVLFGLPGT
DGFFCSLATSLIIQFKLLGYKFKNTKLYKNEPDITLWNNLKQLVDYHNYLLSYSKELDATF
KTIFLLQFMISIGSASVSVFIFMQPGDWSNRIKFLLYFVATMVQTAFYCIPLEFVVSSAKQIG
DFVYESNWYQVKDIKFEKCFTLILARTQKNVVFSAYGLIWINLGTFLVICKTVFSFYTYLN
SVNKITSZ
>TcasOr120

MAKKFLLDISADSLRLLWLGQMHPLSPFRRFVTFLILNLAACWLMIALAIKGITISYKSDIF
FVAECLQTCNLMFHGVGKFLNLYFQKNNLKSLLENRSKFWQLDDFRSEKLYSQMQGITF
VIKKVLRYYYLLVLCVVFLFDLQPFATGLLPTGCYLPEGWFKGLTLTLWFLSVSFFLNIQGT
NGFFYSQSVSLIVQFKLLSHRFKTTQFDKKELKELVDYHNFLTSYCKQLNQAFAAIFLLQF
FTSITSASLSIFIFMQPGAWTNRIKFILYYSYTLVETSFYCIPAEILVNAASEIGNSVYDLDWH
KIRINRVKKCIVIILARTQKTMVFTGYGLVNMNLQTFVVYVKTVFSFYTYLNSVRKIZ
>TcasOr123
MAKKFRSDDISADPLRLLWLGQMHPFSPFRRSVAFLVMNVSACWLLAALAIKGIITSYKN
DIFFVAECLQTCNLMFHGIGKFVNLFLHRDNLKKLLKNRSKFWKIDDFQSEEIYQELSEITS
TVKKGLRYYYCGVVVVMLLFDLQPFATGSLPSGCYVPEGWFKSLTVMTWLLSLSFLNGV
QGMDGFFCSISISIVIQFKMLTHRFKNMRLFHNESERKMWKELKELVDYHNFLTRYCKLL
NTIFASIFLLQFLVSIISASVSIFIFMQPGAWSNRIKFILYYLAVVAETSFYCVPAEIIVNSASEI
GYAVSELDWYKIRINQIKKCFVIILARTQRTMVFTGYGLVNMNLQTFVVYVKTVFSFYTYI
NSVRKIEKZ
>TcasOr105
MKPALKLANVLGLDPLRNDNYTQLKKMFCALCIVSLFVSAYLEFFSNFTTFETYETAPESL
IPHFQTMFKMYSLIFSRTEIVELIQMAEQFYKFSQCDERKKLTKLYKRVDLFFYVYASLVAA
ACVLFAIVTLIFKPGKPIFLCYGGLHGLESPEFEIYLVVDLIGIVIISVTVPAFDGLFFYFALYI
YTEFKLLKIAFKTMSGQELREAVKHHDFLLKYIKKLNSVYSPIFLYQFFCNLLAICFCLFML
SRSGIPPEMVSFSKYFLCLLAFLVQSYTFCSIGDLITELSEDVSNAIFYTDWLDDEAYENKT
ARLIIMSRAQNPVMLTIGKFANMNLRTFILIVRNAYSFLAFVNHALNZ
>TcasOr106
MESALKLIDIIGLHPLKSDKYSTMRKTISFLSLVVILISAQLEFLSHLSVFEVYNSGPHSTIPP
LQSLLKMATLHFYKNELIDLMEKSKSFWKLDKFGDLYKQELSKLHRLVTIIVYIYIALLTAT
CVQLAVLTLIFRRGKPIFLCYGGLYGLESPHYEIYSILDAIGIGVISIAVSGYDAMFFFFALDI
YTEFKMIKSAFKRHSDQTVSSYNKQFIEAVKHHDFLLQYINQVNDIFSPMFLFQFFSGLLGI
CFSLFMISRSGLQDINTLSIYSAGLLGFTAQSYTFCLVGEVISELSEDISNEIFYTDWLDDEV
YRNKTAILIVMNRAQESPKLTIGKFADMNLRTFIMIVRNAYSFLAFINNALDZ
>TcasOr107
MENPLKLLHIIGLDPRQSDKYSTIKKVISFLIVLAVLLSALIEFFLHHNESQVYDTAPQSTVP
NLQALLKMFALIIYKKELIDLFTKGNHFWKLDKFGDCHKQKLTKLHKYVDLFFYVYAVIIT
GAFLQLALLILIFEPGKPIFLCYGGLYGLESPQFEFYAVLDFLAIGVIAISVTAYDSIFFYFALY
IYTEFKMIKIAFKRENCAQFIEAVKHHDFLLQYISKVNEVFSVIFLTQFFSGLLGICFNLFMIS
TQGTRDMKSFSTYFVGLVGYTAQSFTFCLIGELISELSEDISNEIFYTDWLDDEVYRNTTAR
LIVMNRAQESPKLTIGKFADMNLRTFIIILRNAYSFLAFINEVLDZ
>TcasOr112
MITRLMAQFAIKGRVGTGGYIMDKVKLAQPLAHLNIIGLDPLKNDRFSKIRTVITVAVFALC
NVFSFSELFLHYNNPHVIVRSSEVVFPFFQNDWKIAIMLVYKKNLAQLIQNTSRFWQIDAF
GKNYQYSMGIKHKYVRIFYLVYRLMLMFSCSQYILLTIGSDRPMILSFGETGGLGSGALLF
YLIFHIVYLLIIFNVINGFDGLFFFLVAHVLSELQMVKVAFSSSKVITFWNHKRRFKSAIQHH
RFVLDYINRLNSIYSILLLNQHISCLFGICFGLYLFISDGFPPDYEHISKYVPYVIYYITQVWV
FCFAGQLIIDWSVNISDEIFYHDWTLNRTYENKTDKLIIIQRAQHAARLSLAGYGNLDLQSF
NLVLKNGLSFFTFVNAVIHKZ
>TcasOr109

MGKVKFTEPLEFLNVVGLNPENCSNFSLFRRVISLGFFLVVITLGLLELLLHFEGLETCSRA
SEAMIVQYQLFIKIAVLLKHRKNLVVLMQKTRKFWPLDKFGQDAKIERPHKLLKAFFFAY
KLIMILMALQYILRKFVSKNGKPLAIAFGESKGLSPKVDHLYFVLHSTSTFVVLHAVTGFD
RLFFFLIGHVLTELKLVKKSYRLTQNRREKFLETVQHHAFALEFVRKLNRIYSQVLLNQHL
SCLFGICFGLFLVSKDGIPPDLGHVTKYVPYVISFITQTFTFCFIGSLLITWSLQVPDAIFYND
WGKNQAYKYKTDKIIAMIRGQRAAKLTLGGFGDLDLESFNLVVKNAFSFFTFVNAMNQK
Z
>TcasOr110
MDKVEFSDPLFFLNVIGMHPFKADKFSKFRLAFSIAVYFAVIFSGVLELIVNSQGLETYARA
SDTLIPQCQLVCKIFVLAKYKKQIARLLNGSQRFWDLGQFGARYGNSFGKTHKYLKSFFL
LYKVMLTFTCLQFLAVKIIFKIPKPIAISFGETKGLEPLYDHLYLVLHAMITLVTINLVNGFDG
LFFYFIGHVLTELKMVKVAFGDSPIETNWSEEKRFKFAVRHHRFVLDFIEQFNIVYCTMLLV
QHLTCLFGICFGVFLMTKDGVPPDLDRASKYLPYIVTFIFQTFTFCFAGNLLLSWSLEIPNEI
FYHDWAKKTTYENKLAKIISMKRGQRAARLTLGGFANLDLDSFRMVLKNALSFFTFVNA
MMNKKAVTSVZ
>TcasOr73FIX
MTRKHIFLNFTVTILKLSFLWPSNDNYDQWRLVKDASLIVSLMPCALPILAHFVLQITGDV
YNMVTITENLIALICIIGMIYMTICFVKNRKLVKTLVKNLPAFTKYSKTTDIILTDKKANLYT
KIFVFYGVIGNVVYMIMPYLNIEKCQQRQNNDVPCGLVTRCWFPFKFDYSPVFEIVFVHQ
FYTCLMVSVIILDLTMLICGFLMHITNQLKHLRGFIKRFDCSSQKIAEDVIYCVKFHTAIITY
SEKTNEAFGTMMMLHITLTSLVISALGFEILIVDNFNDSLRFTLHLLGWLVLLLLICYYGQL
LIDESIAVAEDIYYVPWHLAPVDVQKDIYMILMRSQKPLTLNAANIGVMSFPTFLRVISSAY
SYFTLLLNIKSZ
>TcasOr102
MQNQSKPCQLDMMDETYLQFFVKSFTYLNMLPEKTTFCTTIQQYYVSVIITITTFPILADL
VSQFYEESISFTSVNENFVALSALFAVIYVSVCFINRKHKIRALIADLALFETFSSKAVITETD
KSVKFYTKLFIVYGIVGNLCYGLLPILGYKKCHESKSVHMTRYGIPCGLVVRFLFPFKFDY
SPLAELVALYEILVCILGTSVVIVVTTLICGVLIHITVQLQCLRKIILDLSQVNDLEILEHKMK
FCVKYHTAILDYGIRTDLAFNQMMLLHITWTGFIISVLGFEISTTDDYVEAFRFFMHLLGW
LGMLFVVCYYGQKILDESLAIADAVYTFLWYKKSVIVQRYVLLILLRSQKPLTLRACGVK
VMSLATFLGVLYSAYSYFTLLLKLKPZ
>TcasOr76
MMESTVTRLKRMYLWPTASVTSRKPAFFLITFSCFLLYGSVMHLIVNDISMEEVHVIETTA
GQFGVLYYLTLFTIYRKGILEIYADLSNFTKFGKPYNFDKRNKQLNQWSRWFSVVLYFFVI
SVFAWPGIFTQSCEDLNVALNKTEVCGVVSPVWLPFRFDYKPMKQFVYFWQSFCCLYSN
GGAGTISFAMSETIEHLILRVEDLKILFPKIVAERSPEVRRKMLAKWVDYHLWLLSIGKLM
NDTYRYSFSVIVLCAGTLFGCIGYTVMKNASTNFNSSFIFFGWMESVFVICVCGQRLMDA
FHSVGTTVYNSEWCDTDVDFQKGVILITIRAQKPVRIYAGPFSYVSHLLILTVFQTSYSYIN
LLNASSZ
>TcasOr77
MKYILMKKTIAFLSVTGFWPKTKESTKTRAFCILFSSSFLLFGSLGYLIVYRKFGSDDIDSIE
TATSHFGVLYFMFFWILKRDGLVHIVNLLSDFSKFGEPRFFNDRNRQLDYLLQYCIFVLSVA
TGGVFLCPIIFVKNCEMVKQEKNLTKVCGLVSNVWAPFDYSEYPMKRVVSLWESYCCFIN
FGCGGIMSFTMIKTMEHLHIRVEQLKDMFPDVVNEKNLAVRKQKLEKWVKYHLHLYDIG

ELMNNTYRYCLSVIVLCVGILFGCIGISTMQPGSSHNSLFLFMGWFQSICILCMVGQRLLD
VFLSVGVMAYDSAWYEKDVDFQKAVLMIMIRARRPVLIYAGPFTNLSHLLILGVLQTSYS
YINLLNAKZ
>TcasOr78
MGHAIMTEILTYLTLMGFWPRSPKSSKASAFLIILSTSFLFFGILFYLIVNRQFGSSEIDSIETI
TSQFGVLYYLILFTWKRNDIVEIVELLSDFSKFGKPPFFDQRSTRLNYRLSCIVLILIVANIVV
AALPVIYIDSCHKANEQLNLTKTCGLIAPVWLPFDYNEYPRKHLVFAWEVYCCVMNYVG
SGIGALTMVGTMEHVIIRIEQLKYIFPKILDQPNPRIREQMLKNWVRYHLALFEIGRLMND
AYKWSLSVIVLCVGALFACIGISMLQSTASQINSICLFFGWFPSIAFLCMWGQRLLDSSLSV
GTAVYSSRWYDMDVAFQKSVLMILIRSQKPIRISVGPFTHLSMLLLLGVFQSAYSYINLLNA
TSZ
>TcasOr79
MGHVIMNEILTYVTLLGLWPRSRKSTKTISYLIILSSSFLFFGSLLYLVVHRKFGSNEIDSIET
VTSQFAVLYYMTFFTLKREGTVRIIDQMSDFSKFGKPPLFDQHNKRLNYLLSYFVICLFVAI
VGVVALPAIYTGSCHKANEQLNLTKTCGLVAPVWLPFDYNGYPLKFLVFAWEGYCCIITYA
CSGISSLVLVGTMEHLIIRIEQLKLMFPEILNEANRHIREQKLKNWVQYHLALFGIGKLMTA
TYTYCLSVIVLCVGILFGCIGVSTMQSASSNNSVFLFLGWFQSLIVLSVCGQRLIDTCLSVG
IAVYNSRWYDMDVSFQKSVHMILIRSQKPILIYTGPFSYLSHLLILSVLQTAYSYINLLSARG
Z
>TcasOr87
MKHVIMDELLIFLTFLGLWPRTPTSPKIISYLMIYSTSFLFFGSSIYLILHRKFGSDEIDTIEIIT
SQFGVLYYLTLLVVKRDGITKIVNLLSDFSKFGKPPLFDQRSRRLNLLLRLFVTVLLAATVA
IVSVPVVFINSCNKQNLQLNATKICGLAAPVWLPFDYTQNPRKYFVSAMEIYCATMNYAG
SGSGAFLVIGTMEHLVIRIEHLKNMFPEILNEPDKQIREKRLKKWIEYHLSIFEIGELMNETY
KWPLSVIVLCVGILFGCIGVSTMQSVSFQNSSVFLFFGWFQSIFVLCFWGQRLLDSCLSIRK
AVYNSKWHEMDVSFQKSVLMILIRSERPVLIHAGPFSYLSNLLVLGVLQTAYSYINLLNAR
SZ
>TcasOr89
MKEAVLQQSKKEMHLLNLWPKGHVKHFRFRYVITLIIVSPFTLGTLTHFINVLKENLDVDL
SGDISVIAVVTGLHFMLITFVWGHKKIAYLWENLGPHEYFGKPDNFEKRCKQLNFYSRLYA
YYCYLGLTVYIIMKNRGGIECRRLNVERNLTEICGLVTTFWAPFDIDFFPFRQILFVDQVFAT
YFIVKGGAAISFTTLEVGEYIILKIKHLKRLVKEVFDDPREEVQRKKLVFCIKYHQYIISIQE
LYDGRYKHCNGCYILMVGIIIASLSNEIMKNHNIEALLHLVGWVFSFYICCFSGQSLLSESL
TIPDAAFESKWYEAPVYMQKDLLLMMLRSQKPLMLHATPIGVMSLSLFITLVKTSYSYFT
LLNQSTZ
>TcasOr90
MAKDTSPVLRESIEVMKYLQLWPQNERTNLRRRYFIVIFLCSPLHLGLATHLVVCLKDNLD
VDLSANIAVLSAVTGLTYMLIVFVWSQDKLVHLLAKLDTHEIFGTPDNLTKRSRRLNFYAK
LYSYYCYFGIVIYSLVQIIEMPQCRKMNEEKGLSEICGMIVPFWAPFDIDWFPLKQIFWLNQ
LLGIYIIIKGGAAVSITTFEVAQYICLKIKHLNRLLREAFDDPCDVVVEQKLLHCIRYQQHIIR
TNELFNVCFKHCNGCYVVMVGIIIASLLNQILKEKSVGALVHFAGWICSFFICCHAGQAVIS
ESLTIPEAALDSHWYEAPVKYKKVLLLLLVRSQKAFNLQATPIGIMSFDLFIALLKTSYSYF
TLLHKSTZ
>TcasOr84

MTEEKELRLCLWSCYYLKLSLMWPLKREEFKSSKGLYLRLLVFVIISGSTFTAMIFMHLYK
SLKVGSYDVSEDLAILASNIGYVLMMTMYVSRQKDLELLLLDLSDFKTYGKPPNFDKVR
KRMDLYAHLIFFYSMFGSFVYNMDKIILIDKCKEARRINEVCGSAIPFWTPFETEDLFTLTL
VITYVLINIFVVVKVAMTVSVQVLEISSHINLRIEQLKIFIAGCFDRDFKASRERLDFCIRYH
NVIIDFSERFSRCFSYVMFIHLAITGIIIGCLENQIVQEHQPEAMLHMGGWSTATFIACYGGQ
LLMDASTSIADEFYNCPWYEADVKMRKDLILIILRAQKALFVSTGPFNVLSFALFVSIMKL
SYSIFTVLSZ
>TcasOr92
MKNQEIKICRATLTVLKYSLIWPSEADEMNPGKWYYIRVVTFILFTCPWVLSVFMHLIVSIR
NNADIHLSEDVALMVAFTGVYYMTIIYVKKQPKVAFLLRDLSYFQFGKPPGFDETERILGF
LSKLTFCYSVMAVVIYNYIKYRQKPECERMNKLKGLKENCGMLTPTWWPFEINYSPAFQL
IFLYIFTSTQVMMKLSLMISFNVLEMAHHIILRINHLKTMILESLDEQDYEASKRKIKTCILY
HLEILGFAERMDDCFSNGMFAHLTITAAICGCLEKQFVDGDNQLGSLLHIFGWILALFLAC
LGGQHLINASETISDAIWSSKWYDADLRLRKDLIFMMARSQVGLYLNVGGFGILSYALFLS
VIKMSYSILAMLTSZ
>TcasOr94
MAIKICKFTRKNMQISLIWPREFEEINPGKWYYIRIVIFLITYGVFPFCTFLHAVVVIHNNLDI
RISEDIGAVVSNIGISYMAIIYVQQQNQIAYLLKDLSDFKDFGKPPFFEEENKRLNFWSICTF
IYPTCGASLYNLSKILEKSECNKINEENGLPATCGFIFPIWVPFNINYFPLFHIMLISTWFCTT
MFVRLHLSISYNAFEIAHHIILRIKHLNGMIITCFDCQDYKISRQKFTTCVLYYKQILDLSNR
LNQSFSSIMFVHFTMTSAVCGCLEKQFVDGEYVGGFIHLVGWIISLFIASVGGQDLVNASQ
SISEAIWSSKWYLADIRLKKDVLFMLMRSQKDLHMSVGSFGVLSYAFFVSVLKMSYSILA
MLTSZ
>TcasOr97
MNNQKIQISNMTRKVLRYSLLWPKTNEELNPGIEYQFSVLGFFLVTGVLVLCITIRFFITIKA
VHEVDAEVLAILIASYGSYYMICAHLKNQHKVALLMRDLSVFNNFGKPPNFDKRNNQLN
FVAKLLALYSFLATIFYNGEQLINKTECKRINKEKGLSDHYCGLLAPCWLPFEIDYFPVFHL
ILIYAFTSGYLLIKMAIHISYNAFEIVSNIVLRIEHLKAMILETFENRNKQVCHKKFLQCILYH
IEILDFAARLDDSFFNSMFGHLALTGGICACLEKQIVSGVNVVAGTLHFIGWILALFIGCVA
GQYLINASEILPSAIWTAKWYDADLELKKKVLFMLARSQKSLFIRAGPFGILCYPLFVTVL
KTSYSILCMLTSZ
>TcasOr98FIX
MVKKESEIKISRVTRKLLQYSLLWPTEGEELNPGKWFYFRIFAFLSFTSLWCIAICMHFIFV
MKDKPDWDPTEEIAIIIAIYGTYYIVLAYVKNQRKAAGILRDLSNFDKFGVPPGFEEEEQRL
RVYIICVFIYGFITITFYNFYKMSQKKSCERFNIEHNLHENCGLLSPVWIPFRIDKFPRYELVF
LYLLTCCHLLMKLPLIVSYNALEMVHHIILRINHLKIMITECFDDPDYEISRRKLTQCILYHT
EILEFATRVDDCFSNCMFAHLTLTGTICACLEKQIVAGFSRFGAILHFFGWILALFIACLGGQ
QFINASDTIPEALWASKWYNADLRLRGDLLLMMMRSQRDLHITAGPFGVVSYALFVSVLK
ASYSILCVLTSZ

IR sequences

>CbowIR6
MNLRTKMGLYKNILLFQLLLGYCEGQTTQNINVLFVNEEGNEVAEKALDVALTYLKKNN
KLGISVDIRKVVGNRTDSNAFLESLCSTYSSMLDAQAYPHLVLDTTMTGLGSETVKTFTQ
ALALPTISASFGQEGDLRQWRNIDDNEKDFLIQISPPADIIPEIVRTLVLNQNITNAAILFDKS
FVMDHKYKSLLQNVATRHIITAIKDGNQVVDQLSQLRKLDLVNFFVLASLKNIKRVLDAA
DSVGFFNRKFAWHVITQDDGEIKCVCRNATIMFVKPLPNAAYQDRLGTMKRTYQLNVEPI
ISSAFYFDLTLHSFLAIKEMISDGVWKSVTNYITCDDYNTENVPKRNGLNLKKYFNKESTE
SPTYGPITVLSNGLSYMEFQMQLTSVGVRDGASDKSTILGTWSAGFYNNLTIVEQQVMVN
LTADVVYRVVTVEQKPFMFRDESSPRGYSGYCIDLIEKIADILKFDYEIATVDCFGTMDEN
GKWNGVVKELMEKRADIGLGSMSVMAERENVIDFTVPYYDLVGITVLMKLPETQSSLFK
FLTVLENEVWLCILAAYFFTSFLMWVFDRWSPYSYQNNREKYKDDEEKREFNLKECLWF
CMTSLTPQGGGEAPKNLSGRLVAATWWLFGFIIIASYTANLAAFLTVSRLDTPIESLDDLSK
QYKIQYAPLNGSSTQTYFERMANIEERFYQIWKDMSLNDSLSEVERAKLAVWDYPVSDK
YTKMWQAMKEAGLPNTMDEAVAKVRASKSSTEGFAFLGDATDIKYMELTNCDLTVVGE
EFSRKPYAIAVQQGSPLKDQFNTAILQLLNRRELERLKEKWWNKNPEKKDCEKADDQSD
GISIQNIGGVFIVIFVGIGLACVTLAIEYWWYKYRKGSKIIDVREVAHNPTKPPTFPKQKFSE
HNPDNTKPKLPKRSKF
>CbowIR75q
MLNSLTLFMILFLNFSSAMKNYTEIINIVDELLTKQNIPSEVTAYLCWSKVLKANLFKRLSA
SNILTKIIATDDIVDLFPSEYQIYLVDLDCEGSNEILKKAQRKKLFSRPFRWVFCGNIEQPLF
NDLYFGVDSRIFFIDNAGSEYHIKMPYKREKNSKKFTVNDLAEWNSLQGFTRFDEFAAAR
NRTDLFGMNINISCVYTDSDTLNHLEDYRNIHIDPLTKLSWILVHHLMSILNASATVIFRNT
WGYRDSNTSLFSGMIGDLQTGEAELGGTASFFTIDRIDVVEFYASSAPTYMKFIFRAPPLSY
VTNVFTLPFHTYVWYCSFLLLVLIFFAIYVIVKWEWKDVVFREKLERMHDGSISPLRPTFF
SVLLMEIGAITQQGTDSEPKSNAGRIATIFTFIALMFMYTSYSANIVALLQSTTESIRTLEDLL
NYRISLGVQDIVYAHHYFEVRIQLSIDSYPNQTPGLKWLNTQDLGKLWR
>CbowIR8a
MRNVKIILLENEGQDTILTWYRSIVQAFKSPIKFEEFLISVDGEEFDRERICQAFSNGAMMIL
DLTWTGNDLARTVSMEMDVPYLRIDVSLSPFFDLLHEYLNFRNSTDVALIFDDPSRIDQAI
YYWIDNVQIAMSISESLDAMAAKKLRDFRPTPNSFAIFAETKNMEKMFKIALEENLVTLPE
RWNLVFLDFHHKSFDRGLLKKMPVNLLTLDAGLCCQLLNLNSYCECPSRFNTSKMFLKIA
LNMLVTAIEELFKDDFKFHDNIDCDSNFTKDNEESVRKTFEEVLNKAVGNDNLIRLDNSSN
LRLKTTGSIEIGTDVGTEVFAKYENEAITALRNKIVKPIKAFYRVGITHALPWSYQIKDPVT
KKLVWTGYCVDFTAKLAEKMNFDYELVEPKKGTFGKKHNGVWDGVVGDLASGQTDLA
ITALIMTADKEEVIDFVAPYFEQTGITIVMRKPVRKTSLFKFMTVLKLEVWLSIVAALIVTG
FMVWFLDKYSPYSARNNKKAYPYPCRKFTLKESFWFALTSFTPQGGGEAPKALSGRTLVA
AYWLFVVLMLATFTANLAAFLTVERMQAPVQSLEQLARQSRINYTVVSNSQTHKYFINM
KFAEDTLYRMWKELTLNASTDDSRYRVWDYPIREQYGHILLAINDSNPVANAEEGFKNVN
EHLDADYAFIHDSSEIKYEISRNCNLTEVGEVFAEKPYAVAVQQGSHLQDGISKMILLLQKD
RFFEGLQAKYWNNSVKGDCPNTDDNEGITLESLGGVFIATLFGLALAMITLAGEVLYYRR
KRKTKELNIKQSKVFPEKPLDVFPPKPLLLGNNQITIGNTFKPVNLKEKIRKEREAMKISHI
SLYPRARKPINPFEIK
>CbowIR41a

LFLPGDNLLENLSHIYSMQELKYIADIVIVNREYKDEDSQGLYMSDNVFSLWTHSYRGMD
ENAKRMFLDLWFSKNQSFMLDENLYPDKLVNQMGRKLEMATFQYEPYSIIGSSETESKGS
EMVTCLTFARHYNMTPVLVVNDEGYWGDIFDNWTGYGLLGNLVEDKADIGFSALYTWE
SDYYFLDLSKPLVRTGITCLVPAPSLAAGWTTPLYSFSTTMWAAVGSMFFVCIFVQFFMHY
FHAKIYDDTNQSLKLLDRSILCVLKLFVQQVVTTRETPPGRSGKYFMGLLFTFSLFLSSSYS
SGLSSIMTIPRYGRPINTVEEFAESKISWGATQDAWTMSLKGVEDP
>CbowIR5
MGLIEFVVASLCLNATCEPEDAVVPGVSTHLLKLNELAEELKEETLTVTTFENGQLSGYISQ
NGSFLGTGVAFDIFHILQEKFGFNYTIVLPDADIFMDGFNKKGAKSLLEAKQADIAVSFLPV
IESFRNDVVYSRVFDIAEWNVLMNRPKESATGSGLLAPFTTAVWILIIFSVLVVGPIMYLMI
LIRAKMCKDDNNKIFSLPSCMWFVYGALLKQGSTLNPKSDSSRILFSTWWLFILILTAFYT
ANLTAFLTLSKFTLPITDPTDISRKNYHWVTNKANGLRDYIEYEKHDRLSNGRTLVQDIGK
DRYYADMKDLDILEEYVKKRNMMFIREKTLIKNVMYRDYQEKTKRGVDEEKRCTFVMA
DFPITMFSRGFAYTHDFKYAELFDRTFQYLIEAGIIQFKLRENLPDAEICPLNLGSIERKLRN
TDLMLTYVIVASGLGIAASVFLLEILWRMSKAKYKRTRKRKATTWLEKNNNLMKAKCLH
LHTNSSPPPPYQALFRPPFYYSDRDGGQKKTINGRDYWVIDKSDGLREIIPIRTPSALLFQIS
N
>CbowIR21a
MDYIIRANYTSKSKRTVLHVSSQCFALFKVAVIFPKKSIYGETLSQGVKLMIQAGLLSKIRS
DVEWDMIRSPNGKLLAANSRTTGLKIISYEDRALTLDDTQGMFLLLGAGFLIGAAALTSE
WFGGCLKLFKRIRPPSSDSSIASNPRVHTGRMPRKK
>CbowIR2
MVTEFSCDKLGSFEFLKKLITQGIPTRILLIDKDELSYLFPPNCQIFIVNLQCENSTNILKKA
NSLKLFSFPFRWIIYHHEPINETIFEESFLSLDILVDSDVTLLEENKNKSVSATKIYKRHRNHP
LVIEKMGYWTKTAGLRDDREEKIMVRRRKNLQQIPLNTCIVITHNDSLKHLTDKRDKHID
SIAKVNYVLVEHLSDIVNVTLNYSIQNTWGYKNNKSEWSGMIGELTKNEADIGGTPLFFII
DRVDIIDYIAMTTPTRSKFVFREPKLSYVTNVFTLPFDDYVWASTIALVCIISMVLFIILKWE
WKKKDLPSEKDSSNVPELKDSLTDVILFSFGAFCQQGAPSIPFSVPGRITTIILFVSLMFLYT
SYSANIVALLQSSSTSIQTLEDLLKSRLQVGVDDTVFNRFYFPNASEAVRRAIYLQKVAPPG
KKENFMSIEEGVKRMRQGLFAFHMETGPGYKLVGEMFHESEKCGLKEIQYLQVIDPWLAI
QKNSSYKELLKIGLRQIQESGLQTREVSLIYTKKPICTSRGSSFISVGLVDCYPAAVVSAGG
AILALIVWILELGLYYRPYMWISVKKVFAKSHEKIPGSIEQWPEWPYLK
>CbowIR64a
MDPPRTIFTLKDLAESQLRIGIEDILIDRNYFVQTTDPDAITLYEKKIKGQSNSSGFYSPSEGI
ALVRNGGFAFHVETSTAYPIIEEIFTNQEICELDEIQMYRTQPMHTNLQKNSPFREMMNFC
MLKLVENGNMDRLRKHWDARRPNCIESAKKQEIHVSLSEFCCSPIALTLGVCFSLIFLLVE
CSINYKERLKKVWTFKNHSKSQYPFME
>CbowIR68a
LYTYGMLLLVSIPKLPTGWSLRMLTGWHWLYCLLVVTSYRASMTAILAKPAPKVKIDTLQ
ELVSSQLTCGGWGEMNSEFFKSSDDPLVTTISQNF

>TcasIR40a
MRRDHGGDLVSASFDIVAGFLFEEICICFDKNTNINFLQHLLVRFVSNNIAIKLFNITTVEVQ
DKYFAFLNYQVTNHLGANTIFFSSHKFYEHVLLEINERDFIRRNLIYIFNWGRRPFSRYFVR

NIINVMKVFVITNPRNDTFRIFYNQAVPYKKHHLEMVNWWQHGVGLFNHPTLPAKYNNV
FKDFKENVFKIPVIHKPPWHFVQYGNDSIKVTGGRDDRILSLLSKKLNFRYDYFDPPERIQ
GSSASENGTFKGVLGLIWKRQAEFFIGDVALSHERANYVEFSFITLADSGAFITHAPSKLNE
ALALLRPFQWQVWPAIGVTFVVVGPVLYAIIALPNAWRPRFRVRSHARLFFDCTWFTTTV
LLKQTGKEPSSSHKARFFIIILSISSTYVINDMYSANLTSLLAKPGREKAINNLNQLEKAMAT
RGYDLYVERHSSSYSLFENGTGIYSRLWQMMNRRQTHFLLESVEEGVQLVRDSTNKAVIA
GRETLFFDIQRFGASNFHLSEKLNTAYSAIALQLGCPYIEEINKILMAIFEAGIITKMTENEY
EQLGKKKQTTSETEKELIPGVKKENRRVAKVSEDNEKLQPISIKMLQGTFYLLCIGNIFSGF
ILLAEILVYKHRKTYKHKKRRHRFVYLRKIRHSVASKFGAVVDAVRRVYRRAMHDAFVAT
LEYLE
>TcasIR21a

MQRGLIVLKLCLTALALKSLDKRALQKSHEKSQLEKWEDKFLNRDPSFDQTASLVNLISK
VALDELSGCSATILYDKFTETSSDLLLEKLFRTFPIPYLHGQITDKYHMKVPKLQTSQDTCT
GYILFLKDVMRSKDVVGPQTNNKVVLVSRSSQWRVYEFLASEQSQSFMNLLVIAKSEKIV
SSSIARLICLALHLKFGTALAIYAPNGGKSAVYPSVIANVPKLGFRSAESVTSVITQNGANL
GIGGLYITDTRLKATDMSHIHSQDCAAFISLASTALPRYRAIMGPFHWTVWLSLTLVYLFAI
FPLAFSDKHTLRHLLDKPEEVENMFWYVFGTFTNAFSFFGKDSWSKTDKFATRLLIGFYW
IFTIIVTACYTGSIIAFVTLPVFPATVDTPEQLVRGKYTVGTLDKGGWQYWFENSTDPITQK
LLTRIDFVPDIESGLKNTTKAFFWPYAFLGSRAQLDYIVRTNFTTINKRSLLHISSECFVPFG
VSIIYNKNALYSKIIDQGVLQAVQSGIVDKIKNDVEWETMRSASGKLLAANSYGKSLKALT
VDDRALTLDDTQGMFLLLGIGFLLGGASLLSEWMGGCLHLCKGNRNQSATSIQSNYRSHE
VPTPREKLDSMQFNSFENHKIEEEIVEERNCIIHRQDDDDIEEHINRLFDFEGVFGEANPDS
RTGPEEELSFKNTTKAFFSLYAFLDSRAQLDYIVRTYFTSMNKRSLLHISSECFVPFGVSIIY
NKNALYSKIIDQGVLQAVQSGIVDKIKNDVEWETMRSASGKLLAANSYGKSLKALTVDD
RALTLDDTQGMFLLLGIGFLLGGASLLSEWMGGCLHLCKGKRNQSATSIQSNYRSHEVPT
PREKLDSMQFNSFENHKIEEEIVEERNCIIHRQDDDDIEEHINRLFDFEGVFGEANPDSRTG
PEEELSEENGKK
>TcasIR76b
MGLFEIALAALCLNATCPGEEEPPEFPEVQYLAPDSNDRKTLFAQLTEQLKNENLIITTLKN
DRLSGTEKRNNTILGKGIAFDLLNILQDKFQFNYTLIEPKANVWGAEKFGVLDLLKDKKA
NLSAAFLPVLTQYSNHISYSPSLDTGEWVVLMKRPKESATGSGLLAPFNLPVWLLILLSLV
VVGPVIYFIIYLQAKLCKDDNNKVFPLPACIWFVYGALLKQGTTLNPMTDSSRLLFATWWI
FITILTAFYTANLTAFLTLSKFTLPITEPKDIGEKRYKWVTTKGNALEDTVTVNESLTELGKI
LGQPQRYLYVSDSDILRNYVHKRNWMFIREKPIVEYVMYDDYKEKTRNQIEEAKRCTYVI
TKFSVVSFSRAFAYSKDFKYKPLFDSTLVQIVKCHKCFSLLSRIQYLVESGIIKFKLREELPD
TEICPHNLGNKERQLRNSDLLMTYEIVGGGFIISAIVFIIEVIIRRQKKPKTKSLPLQNPNKH
TFEINLNNNYEKFGHFPYSSKFVTPPPPYHTLFNPPHKSDNMKKRNFNGREYWVYDSISG
ETKMIPMRTPSALLFQYTN
>TcasIR93a
MLLELVLSSAFVCVIRGDSFPSLLTTNATLAVIIDREFLSNEYEVIKHAIESYLVFAKREILKH
GGVNVQYYSWTTINIKKDVTAIFSIASCPDTWRLFRQARDANLLHMAISESDCPRLPPDEA
ITVPLITRGEELPQLLLDLRTRQTYNWNSAFILYDDTLSRDQVTRVVKSITAQYSNLRVNAA
AISFVKLETRLPMDEIRRQVKEILSSVSIKTVGGNFLAIIGYELVELLMEYAKMFGLVNTRT

QWLYIISNTHFRHKDINRFRQLLSEGDNIAFLYNNTVNNDTCTGGIQCHCEEILSGFTRALD
EAILFEWETSSQVSDEEWEAIRPSKLDRRNSLLQGIKTFLLQRGQCDNCTSWLMKTGDTW
GREYQQNGTDSGGLISVGNWRPSDGPSMSDELFPHIVHGFRKRNLPIVTFHNPPWQIIRSN
ESGAVSEYAGVIFELIKELSKNLNFTYTVELAKIGQEFSANLTKNEAQVVTNFIPDSILDMIR
NKSVAFGACAFTVTEESKRLINFTSPISTQTYTFLVSRPRELSRALLFMSPFTGDTWLCLSA
SIVSMGPILYYIHKYSPVYEYKGLSKRGLSSVQNCIWYMYGALLQQGGMHLPQADSARII
VGAWWLVVLVLATTYCGNLVAFLTFPKIDIPITTIDELLAHSGTVTWSMPKGSYLERTLKY
TTEPRFRYLFDKKVEVGNFKNMIEDIENGKHVHIDWKIKLQYIMKQQYLDSDRCDLALGL
DEFLNEQLAMVVSQDTPYLEIINDEIKKLHQVGLIQKWLTDYLPKKDRCWKNNRHIVEVN
NHTVNMDDMQGSFFVLFLGFLLSFFITIGEKLWHKYVTKKKMKIIQPFTT
>TcasIR64a.1
NKISLILVILSKTETYIIKSCLSNAIVDFAILANVAFSLRISCYKLFMHIKLIANVFYNQLDQV
LNRNHYHLAVIIDSGCIDYADFAIQDKKYFYETYHWLVPTTPQNLNNSLNFLQKSPLNINS
DVNVAILNGEGTKWSILDVYNPASSHHGQFTVTKLGLCDETNGYQAKIAGNKYWSRKN
MTGVQFKSAVVVPDPSIKLNDYLTSDKNRQLHSMHRFQSVTVNYCREMYNFSLEIQRTNS
WGYLTPNGHFDGLVGLLERRLVDFGSSPLIYKLDRMPVIDYSYGNWVLRSTFIYRRPKIIE
ASYKIFLRPLSRTVWICIVLMMVLLMLFLKVVFSREKRLLQKRNLVDSSWSFLFLFTLGAF
CQQGATCHPQLLSSRTLSIFVFLFCILTYQFYSASIVSYLLIDPPRKINNLKDLSDSNLRAGIE
DILIDRNYFVQTTDPVAIELFNKKIKFSNNNSGFYEPWDGLDLVKQGGFAFHVETSTAYPIIE
ETFTNEEICELEEVQMYRTQPMHTNLQKNSPFREMMNYCMLHLVENGLMYRLRKYWDA
RKPMCIESAKKFTFNVGLKEFSSGLIVLSYGILISLGLLLREVIVHKK
>TcasIR64a.2
MSPPLPFMILLSVLTQTHALLDINLIENYFTEKSIKSATVFGCFRKTEQLNLVKIFSRGSSPIS
VLNLNQAGVYQSIKSNHQQIGVVLDGDCPESESFLITVSPGFTHIAPNVVFISVRSTETXFD
VKHHWLILSKSIQFLEKIKNAVVNINADIHVAVQSGTNWTIFDVYNPASEHGGSLKYTRVG
FYSRGRGYNAQTNEAKYWRRKDMTGVTFKTMVVLLVPFEGPLEDYLHNDDNRNINTFN
RFQNKLLRFCRDYYNYSMIVELGSSWGYPFPNGSFDGMVGAMEKKLIDFGSSPIFVREDR
ARVIDYGRNTWSWKAGFLFRSPKSRTSIEIFLKPLSTSIWLITGVLATASIVILKMVTTFERN
RYHSTSETSWSLSFLFTLGALCQQGSPWVPKMACGRITAISIFLLSLIIYQFYSASIVSHLLM
KPTNKIRNLKDLTDSSLKVGCEDIIYNKDLFAHTTDKVLKDLYAKKIYGKGNTSHFFPPEK
GLDLVRQGGYAFHIEVARAYPIIETTFPDNAICELREVKLFKNTDLYNTMQKGTPFRDMLE
SCFQRLAEQGILDREKKHWHPRKPECIQSSQAFVTFHVGLDEFYPALLVLLIGIVISLTVLV
VEKQIHIAREKMEREKGVVF
>TcasIR64a.3
FQLRVLMERLFFLSVLAVIIYTTNCTDNHDIITSYIKEKSVKYATVFGCFTKKEKINLVKIISH
ICPISVFDINRLNIENRMESRHFHTGIILDGDCPSAEKFLINCGRSYLFDVKHHWLIVASSEKI
REKFNNVILNINADINVIIPEKPSNWSIIDVYNPASQHGGVLNFTRVGFYNKHDGYKIKYTG
VKYWNRKNLTGVTFKSMVVVTYSKTXKNSAYTIFQLPVPFEGTLQHYLDSDDNRDVNTF
NRFHSRLISFCRDYYNFSLDIEVSKSWGYTNEDGTFDGMVGALERKIIDFGSSPLFLREDR
ARVIDYGRNTWILRSLIKQQFRIISNWGFSAAFIFRNPKVRTSLEIFLRPLPSSVWLITGLLAI
VSIIILKLATSFERRRYVYDVETSWSISVIFTLGAFCQQGSPSTPKMACGRIATFFIFLLSVLIY
QFYSASLVSHLLNKPLTKIKNVRDLLLSPLKAGCEDILYDRDYFLHTTDKVAKELYAKKIL
GKSNSSNFHTPEAGLKLVAEGGYAFHVETATAYPIIESTFQDQAVCELREVPLFRTQPMHAN
FQKKSPFRDMFDTCFQRLAEHGLLVRERKHWHPRKPECIQSSKSIRFNVGLDDFYPALVIL

LVGIVASLLLILVIEKEFRILTENPA
>TcasIR75q.1
SFLGTILTVYKQLAEKKIVLNVLTNHWKINQTKLSQHTFLVGDTLCPQFNSLLSHVSKFFC
YQNSQQTLGQIITSSXKWLVFDQNSTVNTNDLLLDSNFAVASQISNGRFHLKLCYKRAPNE
TIKFNEIGVFSNGFEYYNHFIPTRNRSDLSGVNITVSYVVTKPDYPFDVEDYRFRHLEAFSK
LSYAMVYPMLEMLNCTKKFIQRSSWGYKGANETQFVGGMFGDIQNGTAEIGGTVSFYTV
DRMSVVDYLSVTTPSDLKFILRAPPLSYVNNLFTLPFDTKVWYCLYFIVGVTVLILYVIVR
CESTYENALERRNNIDNIKPKFFDVVMLQIEAITQQGSENEPKTMSGRIAVFIVFLVLMFLY
TSYSANIVVLLQSTSANINTLQDLLNSKITLGVEDVVYSHHYFETQTEFTRKSIYEKKVAPK
NQKSNFMTTEMGIEKMKDEFFAFHVETTAGYKQIMDTFQEHEKCGLIEIDYLNVLYPSITI
RKNSPYKEIVKVNFRKIYESGIRHRQLNRIYYKKPHCVGKGGSFKSVGIVDIYFSVEIFAIG
CFMALWLLLLEVLFKKKIKFLVQ
>TcasIR75q.2
MKILIVFICLLINETTQNNFTDNLIVNTFNFIKILNVPVKISAHICWTRGKFDSLLMKLYXTV
LANTIHFIKSISDKYNTNLIKNVSPKYANPEHQLFIIDLKCNDSLSVLQQAEKFKLFKSPFK
WLLLGNSESLPNLYFGTDSQIFVTEPRSQLDDIKTIYKYSPMVPRFVQHSFDRFYTNTKRT
NLMGTTIKISYVITNLDSLNHLWDYRLQELKKKLYHFLICRNSHIDAINKLNYILVHNLMD
FLNASRQFTMQPTWGYKNSTTGLYSGMAGDLQKGLADLGGTPLFFTPDRIDIIDYIAATTP
TYMKFIFRAPPLSYVTNVFTLPFDSAVWHYCFVMVAVVVVCIYVIVVWEWKETKFEEKD
THSHIDTLRPNIFDVVMFEIGAITQQGTNAEPKSNSGRIITIFSFLTLMFLYTSYSANIVALLQ
STSDSIKNLEDLLNSRIKLGVEDIVYAHYYFENAQEPVRKAIYQQKVAPKGQKPNFMTAEE
GIRKVQQGFFAFHVELSTGYKIIGEVFQEGEKCGLKEIEYVNLIEPWLATQKKSPYKEVMK
IGMRKMHETGVQNREIRKIYTRKPQCHSGGSNFGSVGLIDCYSAFLTFGVGIAFAFLLFVM
ELIVRRYFIRREKERLK
>TcasIR75s
IVLPMINDLIEHFNKTQIILAYLCDKNGTNLLLIRNNNNTNFRRLSGSEPLFXKKLYQVNVL
SPNSRDMPYPTPPAFLTYVLDAGCSNTKQLLLLVPVITHXLIFGNNILKASEQKQFATPFKW
IVYYNNPVELSFFIDEYFTKTNILVDSDVTLATINPTSGTFDLNKIYKRKINGSIIIENIGIWGR
GLGVTDTGYEKITYKRRRNLTKTVLKSCIVITNNDSLNHLTDKRDIHIDSIAKVNYVLVQH
LSDTINASLEYSVRGTWGYKDNKSQWSGMIGELTRNEADIGGTALFLTSDRIRVIDYIAMT
TPTRSKFIFRQPKLSYVANVFTLPFDASVWASVCGLLVIIAGLLYVVVRWEWKKKDYVQV
VVFFAFWVDFPSSVFCRTNRTSRKFTILGSXVFITFGALCQQGSSSVPFSIPGRITLIFLLVSL
MFLYTSYSANIVALLQSSSSSIQTLQDILNSRLDVGVDNTVFNHFYFPNATEPIRRAIYQQK
VAPPGQKPKFYPIEEGIRKMRQGLFAFHVETGPGYKFVSEIFREDEKCGLQEIQYLQVPDP
WLAIQKNSSYKKMLKVGLRLLQENGIQEREVGLIYTKKPQCLARGSSFISVGLVDCYPAA
VVLAGGIGAALAVLILEIYVHQRFVGFLL
>TcasIR8a
MVISENLDKTTANRLKAIRPIPNNFAIVATSSNMEELLQTALDENLVTLPERWNLVFLDFQY
QQFDKKRLKNMPINLLHMDEEICCRFLQSEKCECPHDFNLQENFLSLATNTLAKILKTLTM
ENLLRADLNCDDSRYSEATRTRFYELLQQEVDSNDLVFKENFGLHVNINGVIETGDEKVAE
YNYKTGVTVLDGKKVEPITPFFRIGITHALPWSYKETDSSGNTYWTGYCVDFTEELSKLM
GFGYEFVEPKSGTFGKKRDGVWDGVVGDLATGETDLAITALIMTADREEVIDYVAPYFEQ
TGITIVMRKPVRKTSLFKFMTVLKLEVWLSIVGALIVTGFMVWFLDKYSPYSARNNKKAY
PYPTREFTLKESFWFALTSFTPQGGGEAPKALSGRTLVAAYWLFVVLMLATFTANLAAFLT

VERMQTPVQSLEQLAKQSRINYTVVKDSDTHKYFINMKHAEDTLYRMWKELTLNASTDD
TQYRVWDYPIREQYGHILLAINDSNPVANASEGFRIVNEHTDADFAFIHDSSEIKYEISKNC
NLTEVGEVFAERPYAVAVQQGSHLQDEISKTILNLQKDRFFEQLQAKYWNHSGKGSCPTT
DDNEGITLESLGGVFIATLFGLALAMITLVGEVLYYRRKSKIQNSETKKPKTVQTSENWKT
DTLMPVSLINKDKQSVTIGTEFKPVNRNRDLSEFGHITLYPRARNRITQTSNE
>TcasIR25a
MASSSAIIYRIAIYSRIATAHLNYSDFLNNVLTETHKMLKLVAFILYCTNLANGQTTQNINVL
FVNEEGNLVAEKAVDVATNYIKKNNKLGVNADPVKVVGNRTDASGLLDSLCSSYNEMIA
NSMNPHLVLDTTMTGLASETVKSFTAALGLPTISASFGQEGDLRQWRNIDENEKEYLVQIS
PPADVIPEIIRSLVLSKNVTNAAILFDDSFVMDHKYKSLLQNVATRHVIAPIKEADKIGDQL
RQLRKLDIVNFFILGSFENIKRVLDAADSVGFFNRKFSWHAITQDKGELKCNCRNATITLA
KPLIDAQYQDRLGLIKTSYQLNAEPEIAAAFYFDLALYSFLAVKEMIADGVWKRNNATNYI
TCDDFDGKNTPRRAGLNLKKYFSKEVSETPTYGPISIVSNGYSFMEFTMQISAVGVRESSS
DKSVPLGSWKAGYDNNLTLVDPQIMKNYTADVVYRVVTVEQKPFIIKDETAPKGYKGYCI
DLIQRISEILNFDYEITPVGDQKFGNMDENGKWNGVVRELMEKRADIGLGSMSVMAERE
NVIDFTVPYYDLVGITILMKLPKTPTSLFKFLTVLENEVWLCILAAYFFTSFLMWVFDRWS
PYSYQNNREKYKDDEEKREFNLKECLWFCMTSLTPQGGGEAPKNLSGRLVAATWWLFGF
IIIASYTANLAAFLTVSRLDTPIESLDDLSKQYKIQYAPLNGSSTMTYFERMANIEAKFYEIW
KDMSLNDSLSEVERAKLAVWDYPVSDKYTKMWQAMKEAGLPNTLDEAVKRVKDSRSSS
EGFAYLGDATDIRYLEITSCDLQMVGEEFSRKPYAIAVQQGSPLKDQFNTAILQLLNRRELE
RLKEKWWSKNPEAKKCDKQEDQSDGISIQNIGGVFIVIFVGIGLACITLAFEYWWYKYRK
GGKVVDVQAKHSDVATKINDGFHAKINKLYPRSRF
>TcasIR144
MQVSKILLLSSLLLNRDETSKCLDAIFKQPVVVLRGVPKNLQNFDAWKPETYLILAPNATV
LEQMLEKWSTIESFNPRAKFWLLTHWHEIKPKTLTILAKFYIVNVAIVTRTGQVFTYYPYK
YENIAQPDTKPVLLGQCDNVPSFPDKLPKFWRNTTVQVLTKCLLPYVDCSDLDQGLETQI
FDLVQEFLKFKVRRIFDKSFKFGLAKINGSYSASFRFLQEREVDMAMGSFRSVGSTQFRDF
EFSTNHMEDKLVWVVPKARPMVHWVRLVKIFEPSFWGLLVVLTVAMARVFEKMARFTD
EPMGIYRKSGFRVAVLILIGSYLKKTPKRFEMRIIFIFWIYFCMVLNIVFNSNLTNVFFGTFN
TFQVNSFDDIIKSNLEMGLTDDVMHILSQEQNWPEITSTKVISSCAFGPACLNRTIFQRNLV
CCWGERSIKFRMAKFYTTQVHYVDDHLLFFYLLFYFVKGYPIVPQISKMIVQLKSAGFVQ
FIKSKVDKLEPRQGNELTTKILTLKRLEGPFYFLLVGWVGGIMIFGYEVVTYERKRRKKVR
QEVTKILKKKKMRQNEKVKILEI
>TcasIR41a.1
TKMLFNNFCINILVNFIINNYHKNSRCLLIFTDGDFDYKGEIPTVRIKATNGSFNSYLIFNYH
GCQSVIIYTSNVTALLIKFETEIRLKMERFNERKFLIVPQNPSEDFDKFFNLKQLYFISDLLLV
LPTHNDTIFDLKTHKYVGVIDNNEPVLLDRWFSQNQSFLFGKNLYPNKLQNQLGRPLKM
ATFTYEPYSIIGNVFEQFFENDFILQGKSVGEHHGSELMSAVQFALKYNMTPVPVINEKDY
WGDIFPNWSGNGLLGNLVDDKADVGFSALYTWEFCYHFLELSKPLVRTGITCLVPAPKLS
ERWLTPLFSYSSYLWFCIILTLVIAIFVLSLVLFCYNHNKTLNLNYPLKRKTTYIHFLESAVTI
VLKPVFQQSLTLRELPIEIASKLLMGLVLLLALFLTSSYGSGLATVMTIPTYENAINTVEDFA
NSGLDWGATQDAWIMSIQNAEEQRYVKIVSKFHPISEEELFQFSKSGKFGFSIERLPFEDYA
IGDYIKEDVIDNFHLMKEDLYWEQCVIMLRKNSVLLPALDLFILKIFEAGLISHWQNEAVD
LYMNPKVQRAVKFYRQGQEHTVVKLQWSHVKGPFALLLIGLCISFIIFILELTLKKKRNQF

>TcasIR41a.2
TLGCLTMTNLNVLLQILLKTYFLNTRCIFLFTDSTIDLQVETPIVYFKVSNTLNPSLIFQHHG
CQNILIHHENASDIFVQFENLIRLNNERFNERKYIVTGHNSLKILLTKQLEYVSDLLLVVPK
QTGHYELITHVYRHQNRSKINEPVLLDVWYSQNHSFRQENDLFPNKLTNQNQRVLKIGTL
SYEPYSVIGKLTVNXSPYYLNLGKDDYSFDGTETSLVYEFVHKYNLTPSFTIMGDDLWGD
VYANWTGIGLFGSVLNDEIDIGYAAVYTWEEYYKFMDYTKTLIRSGVTCLVPAPQLAAGW
VTPLRSFSLGMWIALVIVLLSNTIVLNLLFYRNQKYHXNQLFQILLFNAFSKRFFIDSLTTAI
KLYVQQPLTLTLKRGLLKYFIVTNMIMVLFISSSYSSGLSSVMTVPRYGKSIQTVKDLASSH
LNWTGTTDAWIFSLRQVEEANYENIKNRFVVKTQNDLVTASKQYNFGFSVERLPYGHYA
VGPYIQRDVICNYRIMQEDLYWGQCTFLLRKNSVLLPLLDKLILRVFEAGLEAYWENQVK
CFGRKNMNLRDFLGCLPIHGHVCPKRHYVLYTTYXEHDTIKLTWEHVEGAFAVLVLGYA
ASIFTFVIELILDKVRS
>TcasIR68a
MIKNLLPYKCVVLISDDIYGGTFTKSWYRRFGPFITFVVIRVDEYEDLLSPFEETQACLDTA
KNEGCQMYLILLSNALQVSRLLRFGDKYRVINTRAKFVLLYDNRLFDKPLFYLWKRIINVI
FIRRYSGQKSDTKKNMPWYEITTVPFPTQITSILIPRRLDIWTKSKFRKGIDLFRDKTSDLRN
QTLKVAAFSHIPGTTKSLQEKTARTVIGNFSGTEVEILQTVSAAMNFHCELYEPVNVDVDL
WGGKQSSGKYTGLVGEMVSTNADIALGDLYYTPYILDLMDLSIPYNTECLTFLTPESLTDN
SWKTLILPFKYFRPAMWAAVLVCLLICGAVFHALARFHETISQNKSQVLEIHTKRKKIIILSI
CPEIEKLDSNLKYTKMREQYKPPRFEGQSIGLYQFSEPFNSVLYTYSMLLLVSLPKLPTGWS
LRMLTGWYWLYCLLLVVAYRASMTAILARPTPRVTIDTLQELVNSRLKCGGWGEINRQFF
KSSLDPITKLIGENFELVNDSNEAVDRVAQGVFAFYENSYYLKEALVKRQLRFQIARTTQN
QSEREMRDIAREDRNLHIMTDCVIKMPISIGLQKNSPIKPRVDKYIRRVLEAGLIKKWLQD
VMASILNAEVQSTQEEMKAIMNMKKFFGAIVALFIGYFISVVVLIVENVYFHFFVKRNPHY
NKYTRSIHHVKKAE
>TcasIR100l
MPRKLFLWIFFLLVSCYGNLSETHLQFLKRYFVSANSVAISMLQTHHQEVKIRDLAEVISR
KLNSIGTPVVVHENHKSGSLNIIMIVWSLKILRQFLDSLVVPEEKGTYYIIILEQDCATVHSD
FAQILEQFWCEHNVLNVVVQNPCSGGTFYLFLPFEHRDNFWGSCKSWDFNEQMPNKLRN
LNQFPLKISLFLYNPTLIAKLPKGLKTNPRYHNLSASKGYGGLDGFLLRELVDYFNFDPVIV
ENLEEYGRVLPNGTAFGSLGDVVNQRVHFSINSRFLMDYGTKEIEYTFPYISDEICMLVPKS
LKVPTWKTLLKCFNTLSWVLIFVSCLCSTFAWYFVGPSKNLHKLIWQIYCFIVGIPQKIEPS
FSQFVFLLSCFFFNVTIFGIIQGSYFTEFATTSFYPDIDTLEELYESNLPVATHFWFLLDGDTS
DLMTKLKTHKIEATGDCLEQTARQRNIATLGRKSESDLIIRTKYTSRDGTPLVHIVEECHTS
LYLCGIVPKGSHFLAPFNQIITRLFEGGFTTKWYRDVFDGIISEEKPQLDETVSFNSLNMND
LQTAFHILTIGHLFSIMVLIGEVVIKGKHNKKLLT
>TcasIR100
KVTIIILIMMCLSLPKIQTCPIKINHLKEHFKQVKSARIMILQNEIIVTDWLIMELIKDNKITV
TVQKAIRNFEPFNTSNLTRFEALEFNDTIPTLQTDSTCGHLIIVKNEERLYQYLKSDPGFLIL
NPRHFYAIVAMELFKTNVLREFWSLQVSNILLLDCDTSYTVLPFNGTTIRINAYTQRKLLR
NFHNYFLQVSMQPKPPTAIVKFPKPLRENPIYKDLVPFKDYAGLDGCLLKVLTQRLNMKY
VIVGNGQKYGTVLKNGTTTGTLAWIASNKVQISTNGRFLMTYGTNKLEFTVPYSSDQVC
AVVPKALKIPKIIMLAKSLTPSSWFMIFLIYVICVLIYTLMGSTGSTWTLYAIFHGFPVKIVPT
SRQSFFLTSCMLFSIIIMTIIEGSFFKTFTTTTYYKDINTLEELDESELPIAETFFSFTNDKSRIM

TSLKRKKLVINRDDILEQVARKRNIAKLERKRDIKVRLKTEFLDEEGESRLHVVEECFTTF
YIGFIVPKNSIFLPTFNNVIRRIFESGLTQKWYGDVEFSIFLEKIFKLENNIKHHSFSFDNIVSA
LCVLFIGLSLALLVFFWEVTKXKQITLIYVSLIYCIISRH
>TcasIR100j
LTLVQVVICLLEVSHYDNEKFVNVYQHFTLVRYLTLTFLNDGVHRIDLNNLVVDLMSRLN
FSMMIKEKRLGKNSTTFQESDPFQGHIMVVYDVKVLLAFLEESTEVVPKARGSFAILFTSL
KCPTHYETNHALKQLWTNHGTANLIAFCDNIYVYHPFSKNDSTWGATLDYSPATETPNLF
RNFNGYLLRVSLFKRPPTALKQVPSYISNNPIYRDLKPGDFAGLDGTLLRFLSNYLNFTVVI
DESHPTHGRVLKNGTITGSLSDVVSHRVDFSANDWFLIDYQTPEIEPTVPFSYDQVCPVVS
KALKVPQWKAFFFIFDLTSWVLIFFMWLCCVFVWHVLNPFRDLSTIIWEICSVLFGNPVNV
VPLSNQHMFLGSCMVLNIIIMGIIQGSVFTDFTTTTFHKDINTLEELDEAGLKIASSAWYLD
FDTTDLIKRLKTKQIRNYIGSYKDTAFKRGMAVLGRKQDVEHMVKVEFVAEDGSPLLHVT
SECLQTFLLVSLFPKGSPFLPTFNNVITRLFEAGLTVKWYQDVTSTGTMLQQMKNFANRRP
TGLFSLNDAKLAFYALFVGYIASFVTFLTEILTKNHHNNVHNHVDVLKAQHHGQVQVDQ
>TcasIR100n
DTFWIVYQTHFLLTDYLTLHILETEDHKFELRQFTQNILKRVNKYGYFLSVRITKSSLNKRN
KSYHFPSTAYAPSQNLAKLSDDQEFYKAKRLSTDSKHGFALIVWDLTTLHLFLDQDYRTIV
PEGRGTYAIQVVSKQCDVKNEIAFTLQRLWTEYQVINVVAQTPCSCDKTHIFIYHPFVKRE
GFWGLATSHTLDQIKGDSRLISNTLSDFNGFPLRISIFPRTPTAMQTLPKLLHYNPIYRNLTW
SKGFAGLDGLVLATLAEYFNFEVVLVGSLLEDDFGKVLPNGTTVGSLADITERRAVYNAN
ERLVAYFNLDQIDFTVPYTREDICLVVPKAAKIPKWKILFQSLDPQSWCFTLFAYVSCFMF
WYNIGPSRSLPKVSWQMFSFFLGIPTKSFARKLDQVLFLIPCMIFSVVMLGVVQGSFFTKL
TLFSFYQDVNTLEEMADLELPIGAFIWNLIRDDSDVIRRLKSKSVKPPDNIFDMIAAHRNIA
TIETRARAQLLIGSKYVDDDGFPLLHIVNECLTTFLNANIVPKGSALLTVFNAVLGKLFESG
LTRKWNNDVVDSLIAEKMISVNRKRVRTKSFSLYDAQGAFFVILVGYACSVFVFLCEIVLK
XDKICYLALIINKT
>TcasIR100e
DDFWRVTKNHFLLVNSLTIQVLQTEEHQYDLNQYTVTLLKRLNSLNLLVALRMQEKFLSG
RNFPKHSVTNHTFSTTKPKFDPIGGEELTQLKRLSSDSSKGYFIVIWDVESLHNFLDEDFQV
VVPEARAXYMIHFAFTYSTEACKIVKLQVSSVLTRLWIDNNVFNIIAQTSCLCDLEVYVHR
PFVKRGGFWGLTNSYQMSEIVENPRIIANPLINFNQFPLKIGIFPRPPTVIETLPKLLTDSPIY
KNLSFSKGFAGVDGLVLGTLAECLNFDTTVITSKPNSYGYIYKNGTATGAIADVIDRRMVF
SANSRFLLIYNTDQLEFTVPYTAEKMCLAVPKALKVYKWSSMFRCFNKLTWVSIICSFGIC
TIFWYLLKWQKLVTALATIAQFLLGVPANVRPNVPQMLFLNSCMGFNIVIMGIIQGFLFQS
FTTTSFYPDINTIEEMVDSELPLRSSIFYFLRIDNSSLIHKLKSRTMAAPPNVYDLVAFHRNIA
TTDIKSHVDFMVRSRYLDEDGWPLIHTVDECFETFLIANIVPKGSAFLTVFNNVITKLLEGG
LTQKWYEDVINSLILENWINLNRNKSKTHAFSLYDLQVAFYVIIMGCAVAILVFVAEIVHKR
RNXNNCCNNHHKNIIFAA
>TcasIR100f
DDFWVIFSTHFLLATSLTFITVQTNSKQYDLRLLAQAIIQSMDKDQVMTTRHVILHNYAEN
INFNVVFKTGTKKNARDFVTDLLAKTKKLASDSREGFVIITWNVNVLQKFLAQHISEINPR
TRATYLFILISSSDSLRKIKHCLHFLWHKYDILNIVVHVLGCGTTTTLIYRPFCKTKNSWGEI
TAHQIEEIVQQPLLLTNSLQDLNQYPLQVSLFARDPTALTQLPKLLQNNPIYKNLASFYGLD
GSMLSTMAKILNFEVVIVENHDRLPFGRVWPNGTASGTLGDVVNRRVALSSNSRILADYN

TQEIEFTVAYNGDSICVAVPKSLKVPKWRVLFECFDAASWLLTLSVFIVCLCFWYCVALKN
FARILWDVYSFLMGIPTRIVPSRQYFFLSSCMVFNVIILQLLQGWLFTAFTKTVFYPDLDTL
EVLEKTNLPVATNMWFLFKDNSEVIQKLSSRGIGKTPNSLDLVAYSRNICVLDKRQDLELY
SQAKFVGPDGLSLLHIVNQCLTSVLLVNIVPKGSPFLPVFNDIMSRLFESGFTKKWYSDVV
TSRVTEKMVSLGRKERNFSFKIKDLQAAFYVMMAGCVFSLFVFVGELVTHXVFVMNKSS
QSKSHRFLLNCNYGV
>TcasIR100g
TLFKIAEVTFFMVTMHEEFLSLLFGNYYHTNLYQTVKIQEKFARTNNKTGAWYENVALDQ
KLDPPIDQNWQRVKLRTSDSFEGFIIIVWDPQTLDQFLNQNFSLVVPRARATYFLLFVFSIY
ENCKLVNHILKRFWSEFSVLNIIAQTPYCCNKVYIHRPFVKTTNSWGVTQSYTLTEVTQNL
ALITNPLLDLNQFPLRIALFEKNPTAIRKLPKALQNNPIYRNLSRSKGFAGSDGFLLSAMVE
YLNFDPLIDETLEPMNFGHVLPNGTVCGVLAEVVHKRTDYAGNCRLMTYFGTDGYEFTA
PYSSEKIAMVVPKAGKVPRWRSLFNCFNALSWSLIFSIAIVSTVFWCFLRRSQHLKRASWE
MFAHFVGIPCRVVPSRGQFMFLTACMMFNIIILGIIQGSFFTDFTTTSYYPDLNTLEQVLDSN
LPIMAFAWRLLRTNSSPILQKLEQRSIPYEDNVYELVALYRNVAALDRRLDLELEIKTKYSG
RDGVSPLHIVDESLVTFLTTSLVPKGSPFLVVFNHVIRSMFEAGLTAKWYDDVVTSLIIEHK
HKTPSFGVKYRPFTLQDVQAAFYVIAFGYSCSVFVFWCEIIVKFSGKIKHFHYYFVLI

>DmelCG3822
MRSSGVLVLPLLLLQLILNCRKAQSLPDIIKIGGLFHPADDHQELAFRQAVDRINADRSIPRS
KLVAQIERISPFDSFHAGKRVCGLLNIGVAAIFGPQSSHTASHVQSICDNMEIPHLENRWDY
RLRRESCLVNLYPHPNTLSKAYVDIVRHWGWKTFTIIYENNDGIVRLQELLKAHGMTPFPI
TVRQLSDSGDYRPLLKQIKNSAEAHIVLDCSTERIHEVLKQAQQIGMMSDYHSYLVTSLD
LHTVNLDEFRYGGTNITGFRLINEKIVSDVVRQWSIDEKGLLRSANLTTVRSETALMYDAV
HLFAKALHDLDTSQQIDIHPISCDGQSTWQHGFSLINYMKIVEMKGLTNVIKFDHQGFRTD
FMLDIVELTPAGIRKIGTWNSTLPDGINFTRTFSQKQQEIEANLKNKTLVVTTILSNPYCMR
KESAIPLSGNDQFEGYAVDLIHEISKSLGFNYKIQLVPDGSYGSLNKLTGEWNGMIRELLEQ
RADLAIADLTITFEREQAVDFTTPFMNLGVSILYRKPIKQPPNLFSFLSPLSLDVWIYMATAY
LGVSVLLFILAKFTPYEWPAYTDAHGEKVESQFTLLNCMWFAIGSLMQQGCDFLPKALST
RMVAGIWWFFTLIMISSYTANLAAFLTVERMDSPIESAEDLAKQTRIKYGALKGGSTAAFF
RDSKISTYQRMWSFMESARPSVFTASNGEGVERVAKGKGSYAFLMESTSIEYVTERNCELT
QVGGMLDTKSYGIATPPNSPYRTAINSVILKLQEEGKLHILKTKWWKEKRGGGKCRVETS
KSSSAANELGLANVGGVFVVLMGGMGVACVIAVCEFVWKSRKVAVEERLSAILNE
>DmelCG5621
MISTEASFPLGFILTSLLLAFPGCRGERTNVGLVYENTDPDLEKIFHLAISKANEENEDLQLH
GVSVSIEPGNSFETSKKLCKMLRQNLVAVFGPTSNLAARHAMSICDAKELPFLDTRWDFG
AQLPTINLHPHPATLGVALRDMVVALGWESFTIIYESGEYLPTVRELLQMYGTAGPTVTVR
RYELDLNGNYRNVLRRIRNADDFSFVVVGSMATLPEFFKQAQQVGLVTSDYRYIIGNLDW
HTMDLEPYQHAGTNITGLRLVSPDSEQVQEVAKALYESEEPFQNVSCPLTNSMALVYDGV
QLLAETYKHVNFRPVALSCNDDSAWDKGYTLVNYMKSLTLNGLTGPIRFDYEGLRTDFKL
EVIELAVSGMQKIGQWSGEDGFQENRPAPAHSLEPDMRSLVNKSFVVITAISEPYGMLKET
SEKLEGNDQFEGFGIELIDELSKKLGFSYTWRLQEDNKYGGIDPKTGEWNGMLREIIDSRA
DMGITDLTMTSERESGVDFTIPFMSLGIGILFRKPMKEPPKLFSFMSPFSGEVWLWLGLAY
MGVSISMFVLGRLSPAEWDNPYPCIEEPTELENQFSFANCLWFSIGALLQQGSELAPKAYS

TRAVAASWWFFTLILVSSYTANLAAFLTVESLVTPINDADDLSKNKGGVNYGAKIGGATFN
FFKESNYPTYQRMYEFMRDNPQYMTNTNQEGVDRVENSNYAFLMESTTIEYITERRCTLT
QVGALLDEKGYGIAMRKNWPYRDTLSQAVLEMQEQGLLTKMKTKWWQEKRGGGACS
DADEDSGAVALEISNLGGVFLVMGVGSFFGIFVSLLEMVLGVKERSDENQEAPDSDASSL
GFANLGGVYLVMFVGSCFGSIYGLVNCVVSVYLRARENKVSFKTELLDEIRFILQCSGNTK
AVKYPKNSSRSNASSKSKGSSMSVDSLPEDTSEADASGKHNHGKK
>DmelCG9935
MLIASGFLLFQFLSYGLGVPPLVRIGAIFSNQPGMYNSELAFRYAIHRLNMDKSLLPETTVD
YYVEYVNRFDSFETVQKVCKLIRVGVQAVFSPTDSVLATHINSICDALDIPNIGRSAHDFSI
NVYPSKQLVNYAFNDVIQYLNWTRFGILHEKENGIINLHQLSRSFHGEVHMRQVSRDSYV
SALNEFKGKEIHNIIIDTNSNGISILLKNILQQQMNEYKYHYLFTSFDLETYDLEDFKYNFV
NITSFRLVDTADVGVKQILKDIGLYSHHIFKKPYLNLHIKKSTILESEPALMFDSVYVFAIGL
QTLEQSHSLTLLNISCEEENSWDGGLSLINYLNAVEWKGLTGPIQFKDGQRVQFKLDLIKL
KQHSIVKVGEWTPHGHLNITEPSMFFDAGSMNVTLVVITILETPYVMMHYGKNFTGNERF
YGFCVDILETISREVGFDYILDLVPDRKYGAKDPETGEWNGMVAQLMKYKADLAVGSMT
ITYARESVIDFTKPFMNLGISILFKVPTSEPTRLFSFMNPLAIEIWIYVLIAYFLVSLCIYIVGK
LSPIEWKCINACDLENISIGNQFSLTDSFWFTIGTFMQQSPDIYPRAMSTRIISSTWGFFSLIIV
ASYTANLAAFLTTERMINPIENAEDLASQTEISYGTLDSGSTMTFFRDSVIETYKKIWRSMD
NKKPSAFTTTYEDGIKRVNQGNYAFLMESTMLDYIVQRDCNLTQIGGLLDTKGYGIATPK
GSPWRDKISLAILELQERGDIQMLYDKWWKNTDETCTRKNTSKQSKANSLGLESIGGVFV
VLIAGIIVAAVVAFFEFWYNFRYNYEATPSQSVVNNKYNQDGILESERNYTPPDRSFWIEIA
EELRYASWCMNKQKRPALTRTCSKCTIPKGQRINKL
>DmelCG11155
MVRKKREIVIKENIQGRSYLKKICCSYIILSILVISNALPPVIRVGAIFTEDERESSIESAFKYAI
YRINKEKTLLPNTQLVYDIEYVPRDDSFRTTKKVCSQLEAGVQAIFGPTDALLASHVQSIC
EAYDIPHIEGRIDLEYNSKEFSINLYPSHTLLTLAYRDIMVYLNWTKVAIIYEEDYGLFNLM
HSSTETKAEMYIRQASPDSYRQVLRAIRQKEIYKIIVDTNPSHIKSFFRSILQLQMNDHRYH
YMFTTFDLETYDLEDFRYNSVNITAFRLVDVDSKRYLEVINQMQKLQHNGLDTINGSPYIQ
TESALMFDSVYAFANGLHFLNLDNHQNFYIKNLSCTSDQTWNDGISLYNQINAAITDGLTG
TVQFVEGRRNIFKLDILKLKQEKIQKVGYWHPDDGVNISDPTAFYDSNIANITLVVMTREE
RPYVMVKEDKNLTGNLRFEGFCIDLLKAIATQVGFQYKIELVPDNMYGVYIPETNSWNGI
VQELMERRADLAVASMTINYARESVIDFTKPFMNLGIGILFKVPTSQPTRLFSFMNPLAIEI
WLYVLAAYILVSFALFVMARFSPYEWKNPHPCYKETDIVENQFSISNSFWFITGTFLRQGS
GLNPKATSTRIVGGCWFFFCLIIISSYTANLAAFLTVERMISPIESASDLAEQTEISYGTLEGG
STMTFFRDSKIGIYQKMWRYMENRKTAVFVKTYEDGIKRVMEGSYAFLMESTMLDYAVQ
RDCNLTQIGGLLDSKGYGIATPKGSPWRDKISLAILELQEKGIIQILYDKWWKNTGDVCNR
DDKSKESKANALGVENIGGVFVVLLCGLALAVVVAIFEFCWNSRKNLNTENQSLCSEMA
EELRFAMHCHGSKSRHRPRKRSCLNCSSVPTYVPSNVSTSNVGVYYNYFN
>DmelIR8a
MELPLLVLLLALRFAGSEVLKITFWIEPVQRAEFDTDIAMVLKELDALRLDVKVDDTTLTL
TRSEDGLDMQRFCEILSTVGASAVIDLTYSHWEEGYNLVRSLGIGYVRLERIMRPFLDMFG
DFMRQKRANNVAMVFMNARDAVEAMQQMLVGYPFRTLIMDASQTDPGQHFLERIRSLR
PAPTYIALFARAAAMNGIFEKVQKADLFQRPLEWHFVFLDTRDRVFKYRRQAELCTRFTL
NPRAICRSMPMPDLYCGSGFTMQRAMLLNVLRSLINAAQVSPGYPLAIYQDCNATASSSE

VSDPLEKDDYNWLDMVHWSNFLAYAPPLPHIQDQFQSPVPGLTFAVNISAGYYSSEHEAK
TDLAAWSSVGEMRLLNETISPARRFFRIGTAESIPWSYLRREEGTGELIRDRSGLPIWEGYCI
DFIIRLSQKLNFEFEIVAPEVGHMGELNELGEWDGVVGDLVRGETDFAIAALKMYSEREEV
IDFLPPYYEQTGISIAIRKPVRRTSLFKFMTVLRLEVWLSIVAALVGTAIMIWFMDKYSPYSS
RNNRQAYPYACREFTLRESFWFALTSFTPQGGGEAPKAISGRMLVAAYWLFVVLMLATFT
ANLAAFLTVERMQTPVQSLEQLARQSRINYTVVKDSDTHQYFVNMKFAEDTLYRMWKE
LALNASKDFKKFRIWDYPIKEQYGHILLAINSSQPVADAKEGFANVDAHENADYAFIHDSA
EIKYEITRNCNLTEVGEVFAEQPYAVAVQQGSHLGDELSYAILELQKDRFFEELKAKYWNQ
SNLPNCPLSEDQEGITLESLGGVFIATLFGLVLAMMTLGMEVLYYKKKQNALEITQVRPVN
DSSGSGGNSSTAPPTATSTTKQAWHIPVLEAEEKPAKVSPPPSFETATFRGKKLPARITLGDG
KFKPRHGLYARRNLGASDSHSGYME
>DmelIR25a

MILMNPKTSKILWLLGFLSLLSSFSLEIAAQTTQNINVLFINEVDNEPAAKAVEVVLTYLKK
NIRYGLSVQLDSIEANKSDAKVLLEAICNKYATSIEKKQTPHLILDTTKSGIASETVKSFTQA
LGLPTISASYGQQGDLRQWRDLDEAKQKYLLQVMPPADIIPEAIRSIVIHMNITNAAILYDD
SFVMDHKYKSLLQNIQTRHVITAIAKDGKREREEQIEKLRNLDINNFFILGTLQSIRMVLES
VKPAYFERNFAWHAITQNEGEISSQRDNATIMFMKPMAYTQYRDRLGLLRTTYNLNEEPQ
LSSAFYFDLALRSFLTIKEMLQSGAWPKDMEYLNCDDFQGGNTPQRNLDLRDYFTKITEP
TSYGTFDLVTQSTQPFNGHSFMKFEMDINVLQIRGGSSVNSKSIGKWISGLNSELIVKDEE
QMKNLTADTVYRIFTVVQAPFIMRDETAPKGYKGYCIDLINEIAAIVHFDYTIQEVEDGKF
GNMDENGQWNGIVKKLMDKQADIGLGSMSVMAEREIVIDFTVPYYDLVGITIMMQRPSS
PSSLFKFLTVLETNVWLCILAAYFFTSFLMWIFDRWSPYSYQNNREKYKDDEEKREFNLKE
CLWFCMTSLTPQGGGEAPKNLSGRLVAATWWLFGFIIIASYTANLAAFLTVSRLDTPVESL
DDLAKQYKILYAPLNGSSAMTYFERMSNIEQMFYEIWKDLSLNDSLTAVERSKLAVWDYP
VSDKYTKMWQAMQEAKLPATLDEAVARVRNSTAATGFAFLGDATDIRYLQLTNCDLQVV
GEEFSRKPYAIAVQQGSHLKDQFNNAILTLLNKRQLEKLKEKWWKNDEALAKCDKPEDQ
SDGISIQNIGGVFIVIFVGIGMACITLVFEYWWYRYRKNPRIIDVAEANAERSNAADHPGKL
VDGVILGHSGEKFEKSKAALRPRFNQYPATFKPRF
>DmelIR21a
MSYYWVALVLFTAQAFSIEGDRSASYQEKCISRRLINHYQLNKEIFGVGMCDGNNENEFR
QKRRIVPTFQGNPRPRGELLASKFHVNSYNFEQTNSLVGLVNKIAQEYLNKCPPVIYYDSF
VEKSDGLILENLFKTIPITFYHGEINADYEAKNKRFTSHIDCNCKSYILFLSDPLMTRKILGP
QTESRVVLVSRSTQWRLRDFLSSELSSNIVNLLVIGESLMADPMRERPYVLYTHKLYADGL
GSNTPVVLTSWIKGALSRPHINLFPSKFQFGFAGHRFQISAANQPPFIFRIRTLDSSGMGQLR
WDGVEFRLLTMISKRLNFSIDITETPTRSNTRGVVDTIQEQIIERTVDIGMSGIYITQERLMD
SAMSVGHSPDCAAFITLASKALPKYRAIMGPFQWPVWVALICVYLGGIFPIVFTDRLTLSH
LMGNWGEVENMFWYVFGMFTNAFSFTGKYSWSNTRKNSTRLLIGAYWLFTIIITSCYTGS
IIAFVTLPAFPDTVDSVLDLLGLFFRVGTLNNGGWETWFQNSTHIPTSRLYKKMEFVGSVD
EGIGNVTQSFFWNYAFLGSKAQLEYLVQSNFSDENISRRSALHLSEECFALFQIGFLFPRES
VYKIKIDSMILLAQQSGLIAKINNEVSWVMQRSSSGRLLQASSSNSLREIIQEERQLTTADT
EGMFLLMALGYFLGATALVSEIVGGITNKCRQIIKRSRKSAASSWSSASSGSMLRTNAEQL
SHDKRKANRREAAEVAQKMSFGMRELNLTRATLREIYGSYGAPETDHGQLDIVHTEFPNS
SAKLNNIEDEESREALESLQRLDEFMDQMDNDGNPSSHTFRIDN

>DmelIR31a
MNLLISMFILILAAGEGEIIPSMEESVVTNFVKSLVKTKQAIVFSCLFKDFKEISLALMRINQ
FVSVVNLNQSYSLTSILTRENYARTSVMVNARCSGSSELLFEASENRYFNKTYQWFLWGV
DLEVQSLFPLNLNYVGPNAQITYVNETADGYAYWDIHSKGRHLKSNLEINLIATLINDTLNI
ARDIFHLQSIDFRGQFNGLTLRGASVIDKEDIISNEQIESILSRPTKDAGVAAFIKYHYELLG
LLRERFNFTVNFRNSRGWAGRLGNTTFRLGLLGIVMRNEADIAASGAFNRINRFAEFDTIH
QSWKFETAFLYRYTSDLDTHGKSGNFLSPFSDRVWLFCLLTLGAFSIIWVLFEIIDYKILRIR
VNSQKLEHLNQKSSVICIKTTCIERILQTFGACCQQGLDPNPVDRSVRFLVMTLFLFSLVM
YNYYTSSVVGGLLSSSDQGPSTVDEITASPLKISFEDIGYYKVLFRESQNRSITRLIEKKLSS
SRSLNELPIFSHIEDAVPYLKAGGFAFHCEVVDAYPVISEYFDANEICDLREVSGLMEVEIL
NWILHKNSQYTEIFKTAMCNAQEKGFVERILRRRQIKKPACQSLYTVYPVSLSGVLPGFVI
LICKSINKFS
>DmelIR40a
MHKFLALGLLPYLLGLLNSTRLTFIGNDESDTAIALTQIVRGLQQSSLAILALPSLALSDGV
CQKERNVYLDDFLQRLHRSNYKSVVFSQTELFFQHIEENLQGANECISLILDEPNQLLNSL
HDRHLGHRLSLFIFYWGARWPPSSRVIRFREPLRVVVVTRPRKKAFRIYYNQARPCSDSQL
QLVNWYDGDNLGLQRIPLLPTALSVYANFKGRTFRVPVFHSPPWFWVTYCNNSFEEDEEF
NSLDSIEKRKVRVTGGRDHRLLMLLSKHMNFRFKYIEAPGRTQGSMRSEDGKDSNDSFTG
GIGLLQSGQQADFFLGDVGLSWERRKAIEFSFFTLADSGAFATHAPRRLNEALAIMRPFKQ
DIWPHLILTIIFSGPIFYGIIALPYIWRRRWANSDVEHLGELYIHMTYLKEITPRLLKLKPRTV
LSAHQMPHQLFQKCIWFTLRLFLKQSCNELHNGYRAKFLTIVYWIAATYVLADVYSAQLT
SQFARPAREPPINTLQRLQAAMIHDGYRLYVEKESSSLEMLENGTELFRQLYALMRQQVIN
DPQGFFIDSVEAGIKLIAEGGEDKAVLGGRETLFFNVQQYGSNNFQLSQKLYTRYSAVAVQI
GCPFLGSLNNVLMQLFESGILDKMTAAEYAKQYQEVEATRIYKGSVQAKNSEAYSRTESY
DSTVISPLNLRMLQGAFIALGVGSLAAAALNNTINVRSLNSRDKFICGGPVKIWYYLVLLL
WYYFNRGLVGIYQLWHKTSIRNTGKGMPFLGE
>DmelIR64a
MHWWLLVFLPLSCQGLPEHELLELELDYGLAEPQRTSLLQSSLILQFSQDYKHIPRITYFTC
QKPHLQTPNQIPNAAEHRDAFAAKNFQLIKSLYESELFVRIVLLDVLAQSPTSGRPNRPGN
GPTGGFSQTPSQAQSNSEWLEGVLRMEALRQIAVVDLACGAVSRRFLELASAKMLYSEKF
HWLLIEDFAWHGRTQTAEGSGKRDDGEMEEEEPPGQQIQATDDEDLPSIESFLGGMNLYM
NTELTLAKRMSEAAHYTLFDVWNPGLNYGGHVNLTEIGSFTPTEGIQLHTWFRTTSTVRR
RMDMQHARVRCMVVVTNKNMTGTLMYYLTHTMSGHIDTMNRFNFNLLMAVRDMFNW
TFVLSRTTSWGYVKNGRFDGMIGALIRNETDIGGAPIFYWLERHKWIDVAGRSWSSRPCFI
FRHPRSTQKDRIVFLQPFTNDVWILIVGCGVLTVFILWFLTTIEWKLVPHDGSALIKPKGGA
PPRHHYQQQQQQEQVEAPVRPITAVSVVVSKEKVEEKQEEYEDSTPIDAGTLWQRCYQKL
NKYIKDRKAKQKKAPERVGLFLESVLFFVGIICQQGLGFSTSFVSGRCIVITSLLFSFCIYQF
YSASIVGTLLMEKPKTIKTLSDLVHSSLKVGMEDILYNRDYFLHTKDPVSMELYAKKITSV
PTTKENEADEDEPVDPNPVSTDPAKSYRDIVHSHETGAHAKDNAASNWLDPETGLLRVK
HERFAFHVDVAAAYKIIAETFSEQDICDLTEVSMFPPQKTVSIMQKNSPMRKVISYGLRRVT
ETGILTYHFNVWHSRKPPCVKKIETSDLHVDMDTVSSALLILLFSYAITLMILGTEILYSKW
HNRIQLKWVGAT
>DmelIR75a
MQLVQLANFVLDNLVQSRIGFIVLFHCWQSDESLKFAQQFMKPIHPILVYHQFVQMRGVL

NWSHLELSYMGHTQPTLAIYVDIKCDQTQDLLEEASREQIYNQHYHWLLVGNQSKLEFY
DLFGLFNISIDADVSYVKEQIQDNNDSVAYAVHDVYNNGKIIGGQLNVTGSHEMSCDPFVC
RRTRHLSSLQKRSKYGNREQLTDVVLRVATVVTQRPLTLSDDELIRFLSQENDTHIDSLARF
GFHLTLILRDLLHCKMKFIFSDSWSKSDVVGGSVGAVVDQTADLTATPSLATEGRLKYLSA
IIETGFFRSVCIFRTPHNAGLRGDVFLQPFSPLVWYLFGGVLSLIGVLLWITFYMECKRMQK
RWRLDYLPSLLSTFLISFGAACIQSSSLIPRSAGGRLIYFALFLISFIMYNYYTSVVVSSLLSS
PVKSKIKTMRQLAESSLTVGLEPLPFTKSYLNYSRLPEIHLFIKRKIESQTQNPELWLPAEQG
VLRVRDNPGYVYVFETSSGYAYVERYFTAQEICDLNEVLFRPEQLFYTHLHRNSTYKELFR
LRFLRILETGVYRKQRSYWVHMKLHCVAQNFVITVGMEYVAPLLLMLICADILVVVILLV
ELAWKRFFTRHLTFHP
>DmelIR75b
MNFSVLESHFKEAQIFVDADVTYVTHDPFSKNFLLYDVYNKGRQLGGELNITADREIFCN
KTNCRVERYLSELYTRSALQHRKSFTGLTMRATAVVTALPLNVSIKEIFDFMNSKYRIQLDT
YARLGYQARQPLRDMLDCKFKYIFRDRWSDGNATGGMIGDLILDKADLAIAPFIYSFDRA
LFLQPITKFSVFREICMFRNPRSVSAGLSATEFLQPFSGGVWLTFALLLLLAGCLLWVTFILE
RRKQWKPSLLTSCLLSFGAGCIQGAWLTPRSMGGRMAFFALMVTSYLMYNYYTSIVVSK
LLGQPIKSNIRTLQQLADSNLDVGIEPTVYTRIYVETSEEPDVRDLYRKKVLGSKRSPDKIW
IPTEAGVLSVRDQEGFVYITGVATGYEFVRKHFLAHQICELNEIPLRDASHTHTVLAKRSPY
AELIKLSELRMLETGVHFKHERSWMETKLHCYQHNHTVAVGLEYAAPLFIILLGAIILCMG
ILGLEVIWHRHCTLH

>DmelIR75c
MTSWPLYRLIVFNLLEINLSNLMVFHCWSIKEAFPLVEMLNQNGIFSQYIDVQNPDNLANV
HKEYLDSDLVSLNADVTYVSREDEERFILHDVYNKGSHLGGKLNITVDQTLQCNRSHCQ
VKEYLSELHLRPRLQHRMDLSSVTFRLAALVSVLPINSSEEELLEFLNSDRDSHMDSISRIG
NRLIMHTQEILGFNVQDAFGGAIGMLTNESAELCTTPFVPSWNRLHYLHPMTEQAQFRAV
CMFRTPHNAGIKAAVFLEPFMPSVWFAFAGLLIFAGVLLWMIFHLERHWMQRCLDFIPSLL
SSCLISFGAACIQGSYLMPKSAGGRLAFIAVMLTSFLMYNYYTSIVVSTLLGSPVRSNIRTIQ
QLADSSLDVGFDTVPFTKTYLVSSPRPDIRSLYKQKVESKRDPNSVWLSPEEGVIRVRDQP
GFVYTSEASFMYHFVEKHYLPREISDLNEIILRPESAVYGMVHLNSTYRQLLTQLQVRMLE
TGITSKQSRFFSKTKLHTFSNSFVIQVGMEYAAPLFISLLVAYFLALLILILEICWARYAKKKF
STIIPQNQ
>DmelIR75d
MKVQVAHWLPLIFFLLVSGTPRVAGSWRSEYSRQDPDPKTRWGNQLPDMLVAYYRHHGV
HSLMLVVCHTDIADFRLWKLWQHFNLNNFYVQVSTESSLRDLQHVDALDEHKDAPPPKS
FHANNSTHWETSFLLPALPYKMGILLLEFSSECALNLLRWSAASEHNYFTTNRFWLLLTED
PGDIDLLEDPEIFIPPDSELRVLHYENVGNFSCSLIDLYKVAAWKPLKRTLVGHNIRNSRHVI
HALQHFGSAITYRQDLEGIVFNSAIVIAFPDLFTNIEDLSLRHIDTISKVNHRLMLELANRL
NMSYNTYQTVNYGWRQPNGSFDGLMGRFQRYELDLAQLAIFMRLDRIALVDFVAETYRV
RAGIMFRQPPLSAVANIFAMPFENDVWVSILMLLIITTVVLVLELFFSPHNHDMSYMDTLN
FVWGAMCQQGFYVEVRNRSARIIVFTTFVAALFLFTSFSANIVALLQSPSDAIQSLSDLGQS
PLEIGVQDTQYNKIYFTESTDPVTKNLYHKKIASKGENIYMRPLLGMEKMRTGLFAYQVE
LQAGYQIVSDTFSEPEKCGLMELEPFQLPMLAIPTRKNFPYKELIRRQLRWQREVSLVNRE
ERKWIPQKPKCEGGVGGFVSIGITECRYALGIFGCGAAVSFVLFLFEFIFRHFKQVYRIIKGY

REVQR
>DmelIR76a
MENLLVESYYFSTVLSFFAQQFFADSHATCIFWHPAFDFRLETVHPMPLIIMDWHRWANRS
DQDVYDYKIKEDEFEGKGIPYNDWTLRLTVAIERSHCETFIAFQEQIPEFARYFYHASIYSI
WRSLRNRFMFVYTKEFEDKKDSYLSGYIFQDQPNILVITSQYLNSSTFEIKTNRFVGPRNFN
KNPEPVEFYILQRFDAKGTKATWETQSAMSSKMRNLKGREVVIGIFDYKPFMLLDYEKPP
LYYDRFMNTTDVTIDGTDIQLMLIFCELYNCTIQVDTSEPYDWGDIYLNASGYGLVGMILD
RRNDYGVGGMYLWYEAYEYMDMTHFLGRSGVTCLVPAPNRLISWTLLLRPFQFVLWMC
VMLCLLLESLALGITRRWEHSSVAAGNSWISSLRFGCISTLKLFVNQSTNYVTSSYALRTVL
VASYMIDIILTTVYSGGLAAILTLPTLEEAADSRQRLFDHKLIWTGTSQAWITTIDERSADPV
LLGLMEHYRVYDANLISAFSHTEQMGFVVERLQFGHLGNTELIENDALKRLKLMVDDIYF
AFTVAFVPRLWPHLNAYNDFILAWHSSGFDKFWEWKIAAEYMNAHRQNRIVASEKTNLDI
GPVKLGIDNFIGLILLWCFGMICSLLTFLGELWRGQG
>DmelIR76b
MATGIELLVAAALCVACPPLNDSPPTNLIQMGENGTLSPVTELPMDVDASEAGFDADAPV
ETLETINRKKPKLREMLDWIGGKHLRIATLEDFPLSYTEVLENGTRVGHGVSFQIIDFLKKK
FNFTYEVVVPQDNIIGSPSDFDRSLIEMVNSSTVDLAAAFIPSLSDQRSFVYYSTTTLDEGE
WIMVMQRPRESASGSGLLAPFEFWVWILILVSLLAVGPIIYALIILRNRLTGDGQQTPYSLG
HCAWFVYGALMKQGSTLSPIADSTRLLFATWWIFITILTSFYTANLTAFLTLSKFTLPYNTV
NDILTKNKHFVSMRGGGVEYAIRTTNESLSMLNRMIQNNYAVFSDETNDTYNLQNYVEKN
GYVFVRDRPAINIMLYRDYLYRKTVSFSDEKVHCPFAMAKEPFLKKKRTFAYPIGSNLSQL
FDPELLHLVESGIVKHLSKRNLPSAEICPQDLGGTERQLRNGDLMMTYYIMLAGFATALAV
FSTELMFRYVNSRQEANKWARHGIGRTPNGQSVAPSRWLRGWRRLNSGHGQLLGASTHG
QNVTPPPPYQSIFNGGSHGDPLNRWRRPLANGNALGNGVLLGGDSEGGVRRLINGRDYM
VFRNPNGQSQLVPVRSPSAALFQYSYTE
>DmelIR84a
MIKLQVKVISWPLIILTAFLRVLQIESINTNFLELAAFEDFLRSEHLSHVLVVRGDDADGDW
KIECHQKLLANYRVQFYRPEMSANFEDLMFYGSPRTAVLVLNSEHVLVRRQVFGVASEAG
YFNNSLAWFILGSGRESLPVEQLIDQLLSGYRMGIDADITVALRGPDNASMLFYDVYRISR
QANTPLIIEKKGLWTHSGGYQKFGNFKNTWVIRRRNFLNVTLIGSTVLTEKPPGFGDMEY
LADDKQLQQLDPMQRKTYQLFQLVERMFNLSLAISLTDKWGELLDNGSWSGVMGQVTS
READFAVCPIRFVLDRQPYVQYSAVLHTQNIHFLFRHPRRSHIKNIFFEPLSNQVWWCVLA
LVTGSTILLLFHVRLERMLSNMENRFSFVWFTMLETYLQQGPANEIFRLFSTRLLISLSCIFS
FMLMQFYGAFIVGSLLSESARSIVNLQALYDSNLAIGMENISYNFPIFTNTSNQLVRDVYV
KKICKSGEHNIMSLQQGAERIIQGRFAFHTAIDRMYRLLLELQMDEAEFCDLQEVMFNLP
YDSGSVMPKGSPWREHLAHALLHFRATGLLQYNDKKWMVRRPDCSLFKTSQAEVDLEH
FAPALFALALAMVASALVFLLELFLHWLPDFRRRLGTMST
>DmelIR92a
MLLQPLVMHLSQLLRIIVGQYFAEFPSILIVYNNSASTTPLQLEYLSALELVLRELSKPIRLQ
WINVAFLKDLNDLEDQVMGALNSSVTEGFITILSQTHHFIHARYYATRNANVRLKDKRYL
FLCEDESPAELLCMDILQFYPHHLMVRPGTETAPTGPTGPHPDPRRGGGASVSTKNKDDG
EGGAGNKTTSPYRDINFELWTQKFVGAVGNLDALLLDAFLPNETFANRVELYPNKLLNLQ
RRSLLVGSITYVPYTITNYVPAGQGDVDPIHPQWPNRSLTFDGAEANVMKTFCQVHNCHL
RVEAYGADNWGGIYDNESSDGMLGDIYEQRVEMAIGCIYNWYDGITETSHTIARSSVTIL

GPAPAPLPSWRTNIMPFNNRAWLVLISTLVICGTFLYFMKYVSYRLRYSGTQVKFHHSRKL
EKSMLDIFALFIQQPSAPLSFDRFAPRFFLATILCATITLENIYSGQLKSMLTFPFYSAPVDTIE
KWAQSGWKWSAPSIIWVHTVQSSDLETEQILARNFEVHDYSYLSNVSFMPNYGFGIERLS
SGSLSVGDYVSTEALENRIVLHDDLYFDYTRAVSIRGWILMPELNKHIRTCQETGLYFHWE
LEFIDKYMDKKKQEVLMDLANGHKVKGAPQALDVRNIAGALFVLAFGVAFAGCALVAE
LLIHRMDLSK
>DmelIR93a
MNPGEMRPSACLLLLAGLQLSILVPTEANDFSSFLSANASLAVVVDHEYMTVHGENILAH
FEKILSDVIRENLRNGGINVKYFSWNAVRLKKDFLAAITVTDCENTWNFYKNTQETSILLI
AITDSDCPRLPLNRALMTVECRINAVVFVDQTILEENALLVKSIVHESITNHITPISLILYEIN
DSLRGQQKRVALRQALSQFAPKKHEEMRQQFLVISAFHEDIIEIAETLNMFHVGNQWMIFV
LDMVARDFDAGTVTINLDEGANIAFALNETDPNCQDSLNCTISEISLALVNAISKITVEEESI
YGEISDEEWEAIRFTKQEKQAEILEYMKEFLKTNAKCSSCARWRVETAITWGKSQENRKF
RSTPQRDAKNRNFEFINIGYWTPVLGFVCQELAFPHIEHHFRNITMDILTVHNPPWQILTKN
SNGVIVEHKGIVMEIVKELSRALNFSYYLHEASAWKEEDSLSTSAGGNESDELVGSMTFRI
PYRVVEMVQGNQFFIAAVAATVEDPDQKPFNYTQPISVQKYSFITRKPDEVSRIYLFTAPFT
VETWFCLMGIILLTAPTLYAINRLAPLKEMRIVGLSTVKSCFWYIFGALLQQGGMYLPTAD
SGRLVVGFWWIVVIVLVTTYCGNLVAFLTFPKFQPGVDYLNQLEDHKDIVQYGLRNGTFF
ERYVQSTTREDFKHYLERAKIYGSAQEEDIEAVKRGERINIDWRINLQLIVQRHFEREKEC
HFALGRESFVDEQIAMIVPAQSAYLHLVNRHIKSMFRMGFIERWHQMNLPSAGKCNGKSA
QRQVTNHKVNMDDMQGCFLVLLLGFTLALLIVCGEFWYRRFRASRKRRQFTN
>DmelIR7a
MFHHLWLLMGLRSLAMGALHPPQPEAMTPLVAAALEILAEQVSPSQSTLAVMDLTQDAE
HRDERQEQLMTIILRSVGSEMALRTFQKPPAEVPASFVVFLVNSAQAFNTLGFHFTDIHSTR
EFNFLILLTHRMSSRAERLQVLRDISRTCVRFHTSNVILLTEKRDGVVLVYAYRLLNMDCD
LSVNLELIDIYKNGLFRHGHEARSFNRVLSLSGCPLQVSWYPLPPFVSFIGNSSDPEERAQI
WRLTGIDGELIKLLASIFDFRILLEEPCNKCLSPDIKDDCSGCFDQVIISNSSILIGAMSGSHQ
HRSHFSFTSSYHQSSLVFIMHMSSQFGAVAQLAVPFTVIVWLALVVSSLLLVLVLWMRNRL
VCGRSDLASHALQVLTTLMGNPLEARSLPRSSRLRILYAGWLLLVLVLRVVYQGKLFDSFR
LPYHKPLPTEISELIRSNYTLINQEYLDYYPRELTVLTRNGSKDRFDYIQGLGKEGKFTTTSL
IATMEYYNMMHWSTSRLTHIKEHIFLYQMVIYLRRHSLLKFAFDRKIKQLLSAGIIGYFVRE
FDACQYRKPFEEDYEVTPIPLDSFCGLYYISLIWLSAAVVAFILELLSQRIVWLRRIFE
>DmelIR7b
MKYWLYILSCCSLVASTMESSSDWDLAEALAQVVANSEMGRFKTLYIYTHTNSQSTGGHL
EELLDQVLMIVPNNLQARRLLLQQSMEYKPYVHAVLALVDGLPSLSAIYARIRATQDLSHT
LIYMSMPTDAYGEEMQATLRFLWRLSVLNVGVVLRPPGDHILMVSYFPFSALHGCQVISA
NVVNRYQVGTKRWASQDYFPSKLGNFYGCLLTCATWEDMPYLVWRPDGSGSFVGIEGAL
LQFMAENLNFTVGLYWMNKEEVLATFDESGRIFDEIFGHHADFSLGGFHFKPSAGSEIPYS
QSTYYFMSHIMLVTNLQSAYSAYEKLSFPFTPLLWRAIGLVLILACLLLMLLVRWRHHHEL
PRNPYYELLVLTMGGNLEDRWVPQRFPSRLVLLTWLFATLVLRSGYQSGMYQLLRQDTQR
NPPQTISEVLAQHFTIQLAEVNEARILASLPELRPEQLVYLEGSELQSFPALAQQSGSSARVA
ILTPYEYFGYFRKVHPMSRRLHLVRERIYTQQLAFYVRRHSHLVGVLNKQIQHAHTHGFL
EHWTRQYVSAVDEKDESVARIASTSYSTLDGIDGDPSLSESEEDQQVAPVRQNVLSMREL
AALFWLILWANLGAVVVFVLELLLPRIKLRKILRKMKKSTRASATTTSTLSSPSTTKDIPFS

CKDGFQDSWPKCSLLVS
>DmelIR7c
MLHSAVHNVSLVYALVWAIDNYYGMATSTPLAVVQFPTSRESRRLHNDLIDAALGRSSGT
GRIQFLLEDDRVEMTETDTDPPPPSGLTGRPIAIWFLDSLRSYFRLEMYLNQLGSPYKRNG
FFLVIYTGLEDQPMESLKIMFRRLLNMYVLNVNVFLQRDGTVHLYTYYPYGPHHCQSSLP
VYYTAFQDLAAPANGFGLTKPLFPRKLTNMHGCEMVVATFEHRPYVIIEDDPKTPGGRSIH
GIEGLIFRSLAERMNFTIKLVEQKDKNRGEILPDGNFTGILKMMVDGEVNLTFVCFMYSKA
RSDLMLPSTSYTSFPIVLVVPSGGSISPMGRLTRPFRYIIWSCILVSLIFGFVLICLLKITALPGL
RNLVLGRRNRLPFMGMWASLLGGLALYNPQRNFARYILVMWLLQTLILRAAYTGQLYLL
LQDVEMRSPIKSLSEVLAKDYEFRILPALRTIFKDSMPTTNFHAVLSLEESLYRLRDEDDPGI
TVALLQPTVNQFDFRSGPNKRHLTVLPDPLMTAPLTFYMRPHSYFKRRIDRLIMAMMSSGI
VARYRKMYMDRIKRVSKRRNLEPKPLSIWRLSGIFVCCAGLYLVALIVFILEILTTNHRRLR
RAFNVINRYAA
>DmelIR7d
MDIRCVVALLLGLCKVQAVVWPHQHLLEEQLASQISATLQKIFINGLAVYNFGVFISTSYE
EMDRDRVILVHQVLNRNLYPPNFPVAVVLASKMNRKITAQVFTQLLFVQNAEQAIAIAEG
VNRNGLCVIVLLTSQPERPIMTKIFTYFMQERYNINVVILVPRLHGVQAFNVRPYTPTSCSS
LEPVEIDIKDGDLWDVFPRRLKNLHGCPLSVIVWDIPPYMRINWKSSDPMDGLDGLDGLL
LRIVARKMNFTLKLIPNEPNGLIGGSSFMNGTFTGAYKMLRERRANITIGCAACTPERSTFL
EATSPYSQMSYIIVLQARGGYSIYEVMLFPFEKYTWLLLSTILGLHWIVGSRWRMPSPILA
GWMLWIFVIRASYEASVFNFIQNSPVKPSPRTLDQALSGGFRFITDHASYRMTLKIPSFQGK
TLISAGQPVDVFDALLKAPWKTGAFTSRAFLADHLVRHRKHRNQLVILAEKIVDNMLCM
YFPHGSYFAWEINKLLFNMRSFGIFQHHSQILAWDNLPTTTDTDTPGKRIHSSTESVATGFA
ESMSFVVAALNCLMGALCISIVVFGLELLSRRRHWTGLEWLFERV
>DmelIR7e
MNHINEFVARAVLHVVHHYILSVTPSLVLTLCCRSNHTCNFYNKMMSTLFREWGLAPLQI
VNVLRGVPWHPVPGRRHFNVIFTDSFAAFEEIRMEYYSREYNYNEHYFIFLQARDRLLQG
EMRLIFDYCWRYRLIHCSIQVQKSNGDILFYSYYPFGEHGCSDMEPQLINRYNGSMLVEPD
LFPRKLRNFFGCPLRCALWDVPPFLTLDEDQEEVLRVNGGYEGRLLLALAEKMNFTIAVR
KVHVNMRDEALEMLRRDEVDLTLGGIRQTVARGMVATSSHNYHQTREVFGVLASSYELS
SFDILFYPYRLQIWMGILGVVALSALIQLIVGRMLRERMGSRFWLNLELVFVGMPLLECPR
SHTARLYCVMLMMYTLIIRTIYQGLLYHLIRTHQLNRWPQTIESLVQKNFTVVLTPIVQEVL
DEIPSVQHMRFRLLEANSELDPLYFLEANHQLRQHVTASALDIFIHFNRLSADKVHQRGEQ
GSGAHFEIVPEDIISMQLTMYLAKHSFLIDQLNEEIMWMRSVGLLSVWSRWELSESYLRNE
QSFQVLGTMELYAIFLMVLVGLIVGLLVFILELVSMRSIYLRKLFT
>DmelIR7f
MQGEDANLYVARALRLVIENVLAQLSTTLVVTISTRHLGTAHWFEYMMNILMDSWRMVA
VQLLRIRPDLVVNPVPGRKRVSLLMVDSYQGLLDTNITASNANFDDPDYYFIFLQARDHLI
PKELQLILDHCLAHFWLHCNVMIQTAQVEVLVYTYYPYTADACQKAYPIPVNTFDGRKW
KASQMFPDKLSQMHGCPLTVLTWHQPPFVELVWDPKHNRSRGSGFEIQLVEHLARRMNF
SLELVNIALLRPNAYRLAEGSSEGPIEKLLQRNVNISMGYFRKTARRNQLLTTPMSYYSAN
LVAVLQLERYRIGSLALLVFPFELSVWMLLLLALLIHLGIHLPSARRGNEEDGGGGLQVVA
LLLGAALARLPRSWRHRFIAAHWLWASIPLRISYQSLLFHLIRLQLYNTPSFSLDQLLAEGF
QGICTANTQRLLLEMPQLARDPDSIQSVDTPFDWDVLNVLTRNRNRKIFAVANQDVTLSFL

HSSAHPNAFHVVKQPVNVEYAGMYMPKHSFLYEKMDDDIRRLDASGFIHAWRRASFASV
HRKEQVHMTSRRYINHAKLSGIYMVMAGLYLLAGLLFAGEVLLRQRN
>DmelIR7g
MNVTSLLNFESMKYIGAQTQAASINHHVAQALRVFIEDFYQRIAPAFIVVLSCRRPSPMNF
YRNIMQLLYESVDTMIVQLVLVELGRPRRIAGPRTHNLLLVDSLDALLDIEIHTYTAQSDTS
EYYFIFLQQRDALIPHDMQGVFAYCWRHQLINCNVMTQSSGGQVLLHTYFPYAPGQCND
SQPTRINMFLGESWKHRDYFPSKLHNLNGCPLIVLARKVSPFLDLDEGQRELRGLEGRLL
QELSRRMNFSIQFSGLQDQLKNRTTWTEKQLLQKLVQERIAHLAIGYVRKRIQYATNLTPV
FPHYSNRVVGCLLLNAHNLTSLEIWSFPFQALTWICLVAGDRLALVLAVYAASLGLPIDPPE
RPSLQLLFASWLIFGLIVRSMYSALLFFILRYHLHQRLPGNLQDLTHGDYAAVMGRTTLQD
LREVPSLQDLLGLKSVIVTSEREEEVLRTLDRCTLREGAGSHPLFFGLISQDALLHLTQRGH
RAGAYHIIPQDVLEQQLAIYLQKHSHLASHLDHLVMSIRSVGLVHHWAGQMASERYFRSR
FLYREKRIRQPDLWAVYILTAGLYLLSLVVFICELLASRRAGL
>DmelIR10a
MAVLGTVFLLFMLDLKTLNLTRLNGLLVEPTRDLPQLELWLRAGSDHQDAENPYVQWFL
LRTEIPLSIVTYQENRYWMDDPFGRRNLVLVMSLDQLLTNRGAAAPIQKASTFFYILADQD
KDLSADEQLRLEGSCRQLWTQHKVYNRFFLTRDGVWIYDPFKRRDSAFGRLVRYYGSET
LDKLLFRDMAGYPLRIQMFRSVYTRPEFDKETGLLTRVTGVDFLVAQMLRERLNFTMLLQ
QPEKKYFGERSANGSYNGAIGSIIKDGLDICLTGFFVKDYLVQQYMDFTVAVYDDELCIYV
PKASRIPQSILPIFAVGYDIWLGFVLTAFACALIWLTLRVINLKLRIVSLGNQHIVGQALGIM
VDTWVVWVRLNLSHLPASYAERMFIGTLCLVSVIFGAIFESSLATVYIHPLYYKDINTMQE
LDESGLKVVYKYSSMADDLFFSETSPXWNRDLRADVIDEVARFRNKAGVSRYTSLILESS
HFTLLRKIWVVPECPKYYTISYVMPRDSPWEDAVNALLLRFLNAGLIVKWIQDEKSWVDI
KMRSNILEADAESELVRVLTIGDLQLAFYVVIGGNLLAFLGFLAEHFRWKLQKKGV
>DmelIR11a
MRFAILWLFSGCLLPGIQVGIWVVVRAQPTGRDVLLSRLGNQQNELNTRRLANASSYLTR
NYIANRINTLVVREICVECPYELSERQRQLVDQILASLAPELSVLLHKGTAEETTWEYTLFV
VNDHTAFTGQVFIFPDELLEREFFCIVVVSEIQSRQFVRQTVGSIVKSNLQMHFVNVVVVA
QLEDGTVGTYSYKLFKANCTPGITVRQINHFDRITGKPQQSMPDLYPVRNGHLGDCPFNV
GAAHMPPHLIYKRHKDPPPASNVSIPAEDLAGIDWDLLQLLAKALKFRIQLYMPQEPSQIF
GEGNVSGCFRQLADGTVSIAIGGLSGSDKRRSLFSKSTVYHQSNFVMVVRRDRYLGRLGP
LILPFRGKLWGVIIVILLLAVLSTCWLRSRLGLSHPIEDLLTVIVGNPIPDHRLPGKGFLRYLL
ASWMLLTLVLRCAYQARLFDVLRLSRHRPLPKDLSGLIKDNYTMVANGYHDFYPLELTCR
QPLDFSARFERVQRAAPDERLTTIALISNLAYWNHKHPNISRLTFVRQPIYMYHLVIYFPRR
FFLRPAIDRKIKQLLSAGVMAHIERRYMQYENKRKVASNDPVLLRRITKSIMNGAYRIHGL
VIVLATGMFILELLAGRSNGRLRRWMEWVHQ
>DmelIR20a
MLASLNRSTGLSAELLDLYGLVVHFLLSGEHTTLVYFNPAGLDCSWGVLWQRNLTAHPQI
VWQRNYSYPDLYYQFNAKLLVLACLPMDSRAAIQLEILANSLSHLRTVVRLLIEVAGPDQ
VTLARQYLSFCLRRSMLHVELYFRDYHHSLILYSFRAFPSFELVMRWISVGQGVKLFLHKL
DDLRGHRLRVIPDLSPPNTFFYRDARGDNQVTGYLWDFLATFAGRLNAGLEVVRPSWRA
GSASDSSYMLEYSAKGLIDVGLTTTLITKWNLWAIHQYTYPLLVSSWCTMLPVEKPLATPD
LFGRIVCPTLAMTLLLIILVTWLVFRQLRCLTRLKNSRPARIVPHLLTLLLLTTCSAQLLSLLI
FPPYHVRIASFEDLLRGDQKILGMRNEFYNFDGAFRARYAGVFYLIDDPNELYDLRNHFNT

TWAYTMPYIKWLVIKTQQRHFSKPLFRWSKDLCFFDFMPTSVIVAPDSIYWESIKDFTFRIH
QAGLMKHWIRKSFYDMIKAGKMSIKDYSDLETLKPLNIGDLEIVWRVCGAAIAVASAIFIM
ELLYFYINVFFNSL
>DmelIR41a
MFIDLSWSLVLSAIVGKYLNESTICIFWNDKFEFQLLHKSDYISFVGINIKSFDDNGGHYIID
TGLKKKELQNKHLFLDELVIKIIISIEVTHCETFVVFDKDIDRFVNAFNKASVYSIWRSLHN
KFVFAHIANESPESRNHFFEDQPNILFVVRDHSSASSFDIKTNKFVGRKAENPSQMILVDRY
LASEQRFQFGKSLFADKLNNLQGREVIIAGFDYPPYTVIKHNMSTNAQDMGVSGESDFKN
VYIDGTETRIVLNFCEQFNCTIQIDSSAANDWGKVYPNMSGDGALGMLINRKADICIGAM
YSWYEDYTYLDLSMYLVRSGITCLVPAPLRLTSWYLPLEPFKETLWAAILLCLCAEATGLV
LAYKSEQALYVLPGYREGWWTCTSFGVCTTFKLFISQSGNSKAYSLTVRVLLFACFLNDLII
TSIYGGGLASILTIPSMDEAADTVTRLRFHRLQWAANSEAWVSAIRASDEALVKDILYNFHI
YSDDELLRLAQDQHMRIGFTVERLPFGHFAIGNYLGPQAIDQLVIMKDDIYFQYTVAFVPR
LWPLLDKLNTLIYSWHSSGFDKYWEYRVVADNLNLKIQQQVQETMTGTKDIGPVPLGMS
NFAGFIIVWILGSAIATLTFLLELSLTYILKQSNLK
>DmelIR47a
MRQIKLLVWLLVVGVVSSTEQLQFLKNFLEAVHKERSISTILLIQRKVHKNDFLHGLYPIF
WPIICLDETKRVELVNNFNKDFLALVYMESEADTLLLSALAADLNHIRDARIMIWLQMSPS
ENFLDRIVFQASKQKFLNLVVIENTLKTRRFYPFPQPKVQVIDKPFEEKEIYPALWRNFMG
KNAIAVPDLVPPRSFNSFDPKTGHRRESGSIYNVFKAFTQRYNITMLLKWPLIRNTTQEEIIG
KSVRGEIDLPITGQLISFRHPNGSRSQPLLGMTALSIAVPCGPELPMFDRFFLFYGLATPITIT
GYYVLLNTIEIILGTLSDRIKRHPRRKKILNLVLNLRVFSCILSLPTPQGNRLRSVKGQLTMV
MSITGLILSCIVAAQTSTILTMKPQYRHIKNFQELSDSNITVVCNHLNYLTIKQQMDPKFMA
KFMQNIWIVNSIEQMKMIFDLNTSYAYQTFSYKKDPFTLLQMHTTRKAFCRTPGLDLVSG
LAYTAVLEKNSIYALALQDYTLKAFSAGLVYYWAEESIRDLISTVGRTQFEKLPIVIGYQSL
KLQDYNVCWKILLIGGALAFCVFIVEVVVGLINRRI
>DmelIR47b
MREAQIIIFLLTSAAAVTLKQYEFLXSFLKAGEQEQTITTLLMMQKHVHTKNLLQGLYPXP
WPIIHFVETQRIKFIALLYMSSEKDIFLSSLAANLKFERLDKPFGKSNIFPVLWRNYMGXIAL
TLDHLVEPRSFYWTDPRTNIKRRTGYIYMLITNFAEQHNITLQLXSPPNEDMSQMVIIERTH
KGPRSTHNWADDQLETFERXQDSLLPWHGSMAIVVPCGQEMSAYERFHAAHAFRAPIIFF
GFHIFLSLIDFLLRTISDRIRCNPRRIQLLQTVLSLCVLRCILSTSLPNSNXLRSRLRDNSPXX
XVLQAXSYSALWXLTGTAXQXHNRDFQSHKLHDYXTTDGSXHSIEVPGLLKARNXXIXL
FHIFSSLGTKFDLRIGSAGSHTSGVEFRYYELLDRXSSLENNIVSQVFTILKLPYSRFRVLKL
EDCRGCWQTLFVGFSIATFVFIVNVLMGFFRNINQKK
>DmelIR48a
MHLLITETYMIIGKTLHDILNELNERLIISTNIIFCKQFDNLIHFEAQTSRFVYSSLEAFNITSL
WNHVGNDNKLFVIVGNVPPYELFAKLELSSPENCTQFILNNTVDMCADALVKNSKAFSVS
RELRIAPANVIVPHGKPLLSYRYLAAPFNTKVWIALGTYVFLISGFLCLIHWLRSGKWDFS
QNLLEVYSSLLFTVFHLKATNGIERYILFGVLFISGFVYSTSYLRLLKSMLIAETFEKQIQTF
EELAESNIPLLINPYDRMIFQHHHIPKSLWTAVRTVSSETLLNHRSHGYVRLCPAILTASKIPS
HTHRHLFSVCRFSHEQEVVPKGSSXXSLVPCIRKRNREXNHLGCLSGVSWPGISXFFHYG
ALGGEAFGSILLHDANYFPSPRLFRRLAELHYGSY
>DmelIR48b

MILQQSSNLLKLLLLLAISSVRTQGLNDIIIELNQRLLISNNFLYCNQSDKLNEYEIKYLQHM
PPISLMIFTSIESMNFTQVEYNLGADNKLFLIMGNEEPPYDFLHALNLHFQFAEYIIVIDEPV
DLKKSTKWLDFVNHLWQQGYVQLLIYTSYDEKLYHKIIFPETVIEETLVEQYISIRGSFNNL
YGYPVRVAAYNNAPRSMLYVNRWGKHIFAGFYMRFLRAFIDARNGSFVPVLTPSNSPGNC
TLNLVNETVDVCADALAANPAAFSLTHGFRIASANVLVTHAKPLHSYRYLTAPFQWSVWA
CLVIYVLLVVNFLSFIGWLRSGKWEFSKYLLEVFSSLLFSGFYLKEIRGRERYILFGVLFIAG
FVYSTEYLGLLKSMLISEVFEKQIDTFEALVESNITLMVDPYDKILFAKYNMPEILSPIMELV
SFETLLKHRNRFDQDYAYILFSDRMALYDYAQQFLKHPKLLRIPIDFSFLYTGIPMRKRWFL
KHHLGRAWYWAFESGLTRKLALDADFEAVRVGYLSFLITEHVEAQPLNVDYFVMPAIALA
IGYILALLSFVIEMTAWRIREFLGCRKATMTSTGCSEGGHVDVD
>DmelIR48c
MSLLRIILIIIFLRIVSSIPDTIISHLSAELQIKIQIYFGLGNDLYDFSRLDGNYQKIIISHNISEEF
KTYHDEPVLIIIRLERDLNLNLATLDVLRSYLTDRQYNDILLIDNDEENLNSYVDIRKAYW
NAGFSQVLIYNSQQRTWSIKPYPYLQIRPTSLKEYIENRNTRNLMGYPLRVLVTNDPPHCF
VDKDELPGSPNRYKGSIVTMLKIFADQLNATFQANPFREFRRYSTADCVQMVSDDEIDAC
GSIFIRTYTYATSQPVRLNRVVIMAPFGNPIEKFYYFFRPFDLYVWIGTGIIVVYIAVMGSLL
HRWHFKEWNVGQYLLLAVQTLLNRELSLPQSSSGSKFMLLLLLFAIGFILSNLYVALLSMM
LTTKLYQRPIENLADLKAANVNILLQTHNIRPNSVYGSSEELRERFLLVEESQHLEKRNGLD
PSYAYVDSEDRMDFYLYQQKFLRRRRMKKLSNPVGYTWAVQVIKQNWVLEKHYNDHVQ
RFFETGLQNKLVDDVHELAVKAGFLHFFPTQTQTIEPLRLEDIVMAAMVLGGGHALAVIC
FLVELFA
>DmelIR51a
MYNVLVLFLLLFTRAQMEPHRRGHNMTLLRSVLTVIRGRENWKNTPIFLGGHCNSDDLN
NLMSWLQNTMEVTCHTVDTSTSAKNENALGHFNINADNSLGLLFCQSSHELIWFNMDKR
LRRLRGIRLIVILSDKRSSSSKAIMSTFKRLWHFQFQXNFQGYVVSTPVENDIPRVFFVKDK
KTGRKQIRGFGYRTFVEYLHRYNASLHVSNSQQEHAINSSVNMGRIINQIVDGQLEISLHP
YVDVPENMGDNSYPLLIASNCLIVPVRNEISRYMYLLLPLNQSSWILLLGSVIYISGVLYYI
QPGLLHRTWDQRIGLNILDSISRIINICSPSRIYNPSLRYFIVSVHLSILGFVVTNLYSIMLGSF
FTTLVVGEQVDSMQQLIQXQQKVLVKYYEVSTFLRHVEPDLVDGVAQLLVGVNASEQVS
ALLGFNRSYAYPFTLERWEFFSLQQQYAFKPIFRFSSACLGSPIIGYPMKSDCHLQSSLNMFI
MRIQAAGLLRHWVVSDFNDAMRAGYVRLLENFLGFHSLDVDSLRLRWAVLLCGWLLST
LIFLCER
>DmelIR51b
MCKVLTLLVVILLLALTNAAYNVTLLKSVLSLISTREPWINTPIFVGHNTQGGDLNDLIIWL
HQTMGVTSLTMNLFLQPEHIRPLGHFKITRYNGIALFFCHDKHDIMWLTLDRNLRKLRRIR
LIIILRNQRSGSQGAIKSIFNALWQYQFLNVLVLQRDQLYSYTPYPAMRFFKLDIHTEPLFPH
AARNFHGYVVSTPAENDIPRVFHVHDPLTKSRKVLGYAYRTFVEYLDHYNASLRLTNPDE
NLDPTTSVNMNHIVQLIIDGQLEISLHPYVFTPPTATKSYPLLIYPNCLIVPMRNEIPRHMYL
LRPFQLYSWYILLFAVFYITGILYCISPKLNKSSWPQRLGLNFLDAISKILFISPPITIYRPTWR
HLIIFLQLSVLGFMSTSWYNIELDSFFTTIVVGEQVNSMDQLVHQQQRVLVKEYEINTFLR
HVEPRLVEKVSRLLVPVNASEQVSALLSFNRSFAYPFTEERWQFFAMQQQYAFKPIFRFSSA
CLGSPHIGYPMRVDSHLETSLNHFILKIQDTGLLNHWVVSDFNDAMRAGYVRFVDNVLG
YQSIDVDTLRLGWCVLGIGWILSALVFSCEYWHLYPWRFIA
>DmelIR52a

MALGWSVIILGFIGQLSAQILNYTQSRDLELLEGSLFRVLSRLNLEEEYNTLLIYGKECVFH
SLLRKLEISAVTVPSGSTDYDWSFSTAILILSCGYDAENEENSYTLMKLQRTRRLIYLEDNS
EPESVCMRYSLKEQHNIAMVKSDFDQSDTFYSCRLFQTPNYVEGHFFKDQPIYIENFQNM
RGATIRTVADSLVPRTILYRDEKSGETKMMGYLGHMINTYAQKLNAKLHFIDTSKLGAKK
PSVLDIMNWVNEDIVDIGTALASSLQFKNMDSVWYPYLLTGYCLMVPVPAKMPYNLVYS
MIVDPLVLSIIFVMLCLFSVLIIYTQHLSWKNLTLANILLNDKSLRGLLGQSFPFPPNPSKHL
KLIIFVLCFASVMITTMYEAYLQSYFTQPPSEPYIRSFRDIGNSSLKMAISRLEVNVLTSLNN
SHFREISEDHLLIFDDLSEYLVLRDSFNTSFIFPVSVDRWNGYEEQQKLFAEPAFYLATNLCF
NQFMLFSPPLRRYLPHRHLFEDHMMRQHEFGLVTFWKSQSFIEMVRLGLASMEDLSRKR
NEEVSLLLDDISWILKLYLGAMFISSFCFILEILRCGERCKRLWRCRW
>DmelIR52b
MTWLVILLCFLGYMAAHIADISVQNQSLMDNELINLLLKLRNEEFYDTLLVYGKDCEFHS
VIKNVDVAVVLVSDSMNFEWNFSSLTLILSCGPDIDNGGPNSTSIKLQRNRRLVLLKEDFQP
SNICNIYTQKEQYNIALVRENFTKSKSIYTCRYFQDPNVDEVNLSGTKPIFIEQFQNMKGKA
IRIVPDLLPPRVMLYQDANDGELKMIGYVANLITNFAQKVNATLQLDFLKPSTSITEISRMA
KDDELDMGITLEASLNTSNLETSSYPYLLTSYCLMVQVPAKFPYNLVYALIVDPLVLGIIFV
LFLLLSVLLIYSQKMSWQDLSVANILLNDKSLRGLLGQSFPFPLNASKKLRLIFTILCFASIM
LTTMYEAYLQSFFTNPPSEPEICSFQDVGSYNRRIAMSALEVNGLIKTNNSHFREIRMDDLE
IFDNMPECYELRDAFNLSYNYVVTGDRWRSYAEQQTLFKEPVFYFARDLCFSRLIFLSVPL
RRHLPYRHLFDEHMMQQHEFGFVNYWMSHSFFDMVRLGLTSLKDLSRPLAYTPSLLMD
DISWIMKIYLAAIVLCVFCFLLEIGVDKWKRWMKFRNLQILNTC
>DmelIR52c
MVWLIIILFCLGNSSSQILDVTNNSHLDFDYRLFGLLQRLQVEKSYDTLLVYGEDCAIPSLF
ERLQVPAVLVSSGSTNFDWNFSSLTLILSCNFQDEREENYRTLMKLQTSRRLILLKGHIKPE
SVCDFYSKKEQHNVAMVKENFYQLEVVYSCRLFQDQNYEKLNLFDGKSIYKDQFRNMH
GAPIRTLSDKEPPRTIPYIDSKTGEEKFKGYVGMLISQFVKKVNATMQIREDLIKDDEEVSF
VDITNFTSNDILDIGICEARTLEMSNYDAISYPYLMSSYCFMAPLPDSLPFSDVYMAIVAPSI
LIMFLIIFCICSVLIIYIQERSYRSLTIRSVLMNDICLRGFLAQPFPFPRQYNRKLKLIFMLVCF
SSLISTTMYTAYLQAFLWGPPIEPRLTSFDDVKKSRYTMAINIYEREFLEALNVSLEDVEIYD
YGKFSKLRSTFNTNYLFPVTALQWFTINEEQKLFKYKIFYYCDAFCLNQFDILSIPLRRHLP
YRDIFEEHMLLQKEFGLTKYWIDQSYRDMIRANLTTFKDFSPLLENDYIEVHNLYWVFTM
YFVGMGMGLCFFILEILRPLRYWRNCKIKCEYCYAFLKNFAK
>DmelIR52d
MVRIIIILLCLGYTKARILDATNTNHTDLEERLLSLLLRLQQEQFFNTLLIYGEDCAFSSLSR
RLQVPTILVSSGSTSFEWNYSSLALILTCEFKAEREENYQTLKKLQMNRRLILLNGNIKPDS
VCDFYSKKDQYNIAMVNNNFHQVGIIYACRLFQERNYEKVYLSEGNPIYVDQFRNMQGA
LLKSITFNLIPGSMAYRDPKTGQEKHIGYVANLLNNFVEKVNATLDMQVKLHKAGKKTSF
YNITKWASEDLVDIGMSYAAYFEMTNFDTISYPYLMTSTCFMVPLPDMMPNSEIYMGIVD
PPVLVVLIAIFCIFSVMLNYIKQRSWRSLSLVNVLLNDICLRGFLAQPFPFPRQSNRKLKLIS
MLVCFFSVITTTMYTSYLQSFMWGPPIDPKMCSFADLENSRYKLAIRRYDIEMLRPFNVSM
DHVVVFDESSQLEYLRDSFDDNYMYPMSALSWSAFKEQQKLFAFPLFYYSEKLCLKPISF
FSFPIRRHLPYRDLFEEHMLQQNEFGLSTYWIDRSFSDMVRLKLATMNDFSPPRLEDYIEV
SDLSWVFGMYFTGLGISCCCFGLELLGLPSWTRRLRLTNWLRVRN
>DmelIR54a

MWTVITGIVLWAPVLVAGSAVDFIFRAAAEHSLSVIMIRIDYCPYNWAKDIFENQTIPVVVL
SDSETFINIRMFSRPLHVACLPGHELQKDLALLENFTSSLMDFPSQKKIVYISNNFSDPTRM
DYIFETCYHRRIWNIVGLLASDEHRYFYRYHLYPSFRTEYRSLESSTIFDKDFPNMHGHPLT
VMPDQWLPRSVLYVDRRTGKQILAGSVGRFFHVLSWKLNATLQLSKKVTTGRFLNATAL
KELSESFSVDVPASLTIMERVEQLASTSYPMEVTHVCLMVPVARRIPIKDIYFILSSASNMFL
AIVIVSSYGLALNLLRNMTHRDVRLVDFVLNDKALRGILGQSFNLPLSRSFSTRLIFLMLGI
VGLNVSSIFGAGLDTLMAHPPRQFQARSFAGLRRTKIPLVTTEEDFPTWMKLRVPMLVVN
VSEYNHLRNGRNTSNAYFASRLYWNLFSEQQKRFTRELFIYSTDDCLWSLALLSFQWPQN
SLFTEPVSQLILEVNANGLYDFWVGMHYYDMTAAGLSGLEDPSLQLKEREHPTSLRIVDF
QWMWQAYGTFMVIAILVFLLEVSWHRITSLFVSLVY

>DmelIR56a

MGSRFFIRNLILFGLLASSNMQIPFGELEKKFELDVDFLLGVTELVGHIQGLYSITVYADCID
IHPSIQQRIMDKFMVPVNTIGSNLSRPNYHKLDNSRIRIVLFTGLNDTILVNLNKTDVPYSD
NFYMLAYASAIKNKCIELDFIEEVFTLLWKMSIQNAILLIRGEFMMEMWSYLYMGKIHKIK
LTKPNSYLESLRKYNYRFSLEVINDPPAIFWYNSSEQADVTGGGNLSVSGPLGLIIINFLRHL
NVTIDIVPIPGKQTSQYELFQQPDNLRAENGVNMVGSALLKYSPMVTQSRMCLLVSNRR
MIPFSRFLDRLVSPGVHKLTFVSSIGIFVIKYFSHRPRSFVDAIFCTIRFFFAIPLPSIILNRLPV
VDRFIEVFIIIFVQILLSSNISITTSALTTGFWEPPIINVETMRASGLHILTEDPTILQAFKENILP
SSLADLVILVDEDTYFHHVTTLNNSYVYVVQAHNWQIFRLYQQQMTNEPFEIASEELCSK
WRILGIPLNPKSPLRFMFKDYFYRILESGLREQWVHSGFKKFCEFNNLKKLPVDSVDSWQ
PLSIEFYSNVIRAYIIGLVIATLAFVAELLHNGYRRKNVKKT

>DmelIR56b

MLLDTDLASGVIRSPYSFDIPHAFIFNETQFVVPKFCGPYMEIVKHFAEVYHYQLFLDSLES
LPKKSVVEQDIISGKYNLSLHGVIIRPEETSDFFNATQHSYPLELMTNCVMVPLAPELPKW
MYMVWPLGKYIWTCLFLGTFYVALLLRYVHWREPGNATRSYTRNVLHAMALLMFSAN
MNMSVKLKHASIRVIIFYTLLYIFGFILTNYHLSHMTAFDMKPVFLRPIDTWSDLIHSRLRIV
IHDSLLEELRWLPVEYQALLASPSRSYAYVVTQDAWLFFNRQQKVLIQPYFHLSKVCFGG
LFNALPMASNASFADSLNKFILNVWQAGLWNYWEELAFRYAEQAGYAKVFLDTYPVEPL
NLEFFTTAWIVLSAGIPISSLAFCLELFIHRRKQRRPQYERFECYDY

>DmelIR56c

MRSSFRICLFLLTTYHPSHGWNMQHLLNLLAPFGRMNVFQEIVWFVSPHQRLDQLDEFIM
RIDEAFGKSATQTVVNNNTEMRMIYSSARRNHMSFVFTTGAEDPIMKVFSKVLLGRHFYV
SMVIYVDKVGDMHPIYDLLTFAYNQQFFNSMVHFESMEGVNQLFGVSKFPVMSFENRTD
FLKYMGKIWKQVQNARSDVGGFGFTTPLRQDLPHLFQSQGHYDGSTYRIIETFVRFINGSF
KELIMPPDSLGGQVINMKDALQLIRERKMEFCAHAYALFMSDEELEKSYPLLVVQWCLM
VPLYNSVSTYFYPLQPFDWNVWFFALGALLALVLLELMWLRMFGGWSGYRGAVLNSFC
YIINVPIEGQLQQPCLLRFLLLATVFFHGFFLSAYYTSNLGSILTVNLFHAQINTMNDIVSAQ
LPVMIIDYEMEFLLNLNKELPQEFLELLRPVDSAVFSEHQTSFNSSFAYFVTEDHWEFLDEQ
QKHLKQRLFKLSSICFGSYHLAFPLQMDSSLWRDIEYFTFRIHSSGLLNFYARSSFGSALHA
GLVQRMPDTQEYTSAGLQHLAIAFILLLVMSFLAGIVFVLETLSR

>DmelIR56d

MDNRAAELILRERNIFPTNGSDNITLLNNMFVLEMFYRITQLYHFKNFIFYISERLDLNNKD
SQEFFHNFWTYFPMAPNLIITREHHLGIPMMQFISTPSLVMVFTTGKDDPIMELASHNQQGI
HWLKTIFVLFPSLQSRDFETNPESLAQFTAEIKDVYDWVWRKQFINTFLITIKDNVFILDPY

PTPSIVNKTGVWQAEEFFHKYAKNMKGYLVRTPILYDMPRVFKSDRPTNRYEKNFIHGTS
GNLFLGFLEFVNATLMDTSANVTADYLNMTNLLDLVSQGVYETLIHSFTEITTKFVVSYSY
PIGINDCCIMVPYRNQSPADQYMHEALQENVWVLISLFTLYITVAIYLCSPLRPRDLSAAFL
QSICTLTYSVPTFIIRTPTLRMRYLYILLAIWGIVTSNLYISRMTSYFTTAPPVRQINTVQDVV
EANLRIKMLAIEYERMAKSPLQYPESYLNQVDLVDKHMLDLHRDPFNTSFGYTVSSDRW
RFLNLQQLHLRKPIFRLTEICEGPFYHVFPLHKDSHMRSVMTEYIMIAQQAGLMNHWERE
TFWEAVHLHRIHVHLFDDEPMALSLDFFSSLLRTWTLGLILAGLAFAAEMKWHEHVTFKR
RPVIRITRKPRSFLRRFMKL
>DmelIR60a
MWCNNPGLIIIIFLGQILNLCQGIVNLSNETANTVIFMLPEKDLGPDVWKAGVGCLDSFAQI
FFFRNPKERFTRAYNLMLVHAFHLSSPADQIQEGFSKLINEAVTNPGPPDREELFQMRVASD
YNITNGTEDKGELILADNYVIVVDSVDRLKELMKKKIVEMRSWNPGARFLVLFHNATCR
NRPLGVASNIFKDLMEMFYVHRVALLYANSTMNYNLLVNDYYSNVNCRILNVQSVGQCH
DGKLYPNNAVVKASMQDYVSGFSPRNCTFFACSSISAPFVEADCILGLEMRILGFMKNRLK
FDVNQTCSLESRGEMDGPANWTGLLGKVQNNECDFVFGGYYPDNEVADHFWGSDTYLQ
DAHTWYIKMADRRPAWQALVGIFEAYTWIGFILILIISWLFWFTLVMILPEPKYYQQLSLTA
INALAVTISIAVQERPICETTRLFFMALTLYGLNVVATYTSKMIATFQDPGYLHQLDELTEVV
AAGIPFGGHEESRDWFENDDDMWIFNGYNISPEFIPQSKNLEAVKWGQRCILSNRMYTMQ
SPLADVIYAFPNNVFSSPVQMIMKAGFPFLFEMNSIIRLMRDVGIFQKIDADFRYNNTYLN
RINKMRPQFPETAIVLTTEHLKGPFFILVVGSCWAALTFIGELIIHRWRTQLVSTSEQQDRRS
DKRRRRRRRRKPEKDNRWQRQVQVAPVVRFTPVKRRKVFQGQTSQK
>DmelIR60b
MRRSLYLIIAIGLVDVHCVSLRYILNALENELQYRAILLVESASEIESCWEQKYIQGAVPILN
FNANQSLYLKDALNTNILALVCLNENVESTMQALYENLEDMRDTPTILFVLSDSKVQDVF
LECLRRKMLNVLAFKGLDRGFVYSFRAFPTFRVIERNVMDILQYFEQQLEDLGGHTLTTLP
DNIIPRTVVYKSPDGSRQLAGYLYPFLRNYVSTINATLKVCWHLVPEDGMIQLGEVVRLSE
IHDVDFPLGMHGIEHGSTSQNVPLEVSSWFLMLPMEPSLSRAQFFIMLGFEKVTPVLLLLTI
LLSTAHRIEMGLRPSWRCYVLGDRVLQGTLGQAFFLPRRLSVKLMLVYSLILLNGFTFSNY
SITSLETWLVHPPSGHPIHSWEQMRTLNLKVLIVPSELDSMTKALGKQFTESNSDLFELSKS
GNFQDKRLAMDQSYAYPVTCTLWPLLEHAQIRLPKPEFRRSREMVLIPLLIMAMPLPKNS
MFHKSLNRYRALTHQSGLYEFWFKRSFNELVALRKIHYKVNGDHQIYRDFEWQDFSYVW
LGFVGGTIASILVLLAEIGYHRWQLNQN
>DmelIR60c
MEMRLALFFTFACLAGAHDGSLRNMLKSLEDELGYRTILLLEGFVYSFKAFPTLRVVKRR
VKDVRRYFEPQLEDLGGCVLKVVPDGIMPRTMVYQGEDGELQMGGYLSHFIRNYVSTIN
ASLQIRWDLFPEDGDFDMDSLTGSNHVDFPLGLGSLSFQTLHKDVAMEISSWFLMLPMEP
SLPRARFFIRFGISLYLIPLIILLAIVLSNAHRFEAGLTPSWRCCSMGNTVLRGVLAQAFVLP
KGLSPKLMFVYWLLLVSGFFVSNYVIVYLTAWLIQPPTSDPVTDFDQMRRAKLKILMVPT
DMDYLKSIRGAEYVDAHSDVFQTADSTDFQTQRMSMELHFAFSVTGTLWPLLRQAQVKL
HRPIFRRSKEMVFLPFVIMGMTMPNNSIFLSSLKQYRLRTSEAGLYLLWFKKSFSELVAIHK
ISYKEDWVHDSYSDLKWEDFLFAWLGFLGGTTVSCLALLAEIGYHRWLWKRTHQ
>DmelIR60d
MRLAIYVAFLSSIGNRSGFLSSLLMSLGKELHYKTILLVGGSSTCWSLEPFETGVPILNLRG
ENNAYPQDTFNSQMLALACLQTESEDAVKLLYRSLKDMRDTPTLLFASSEEHIHDTLFLGC

FRENMLNVLALTASSKEFIYSYQAFPTFRVIKRKLVEIHRYFEPQLKDLGGHIVSALPGNIM
PRTMCYRNAEGERQLAGYLNTFIRNYVESINGTLRISWGLVPEDDMRHLTISRLSKIQHVD
FPLGIIPLYNKTDKQHVYMEISSWFLMLPMETSVPRAHLFVKLGLERLLPIIVVVGAVLGN
AHRIEVGLGPSWRCYYLADKVLRGALAQPIVLPRRLSPKLMLIYSLLLLSGFFLSNYYMA
SLTTWLVHPPASDRILEWDQLRYLHLKVLTIPEEFKYMSLILGTDFMTAYGSIFQLTNSTDF
QRRRISMDPSYAYPVTTSLWPFLELSQVRLRRPLFRRSYDMVLQPFQVMSLPLPRNSIFHK
SLLRYAALTRETGLYYYWFRRSYYELVALGKISYKEEEGNPYCDLKWNDFRIVWLAFLGG
TIISCLALLLEVAHYRWHLGNSSL
>DmelIR60e
MVIKMISFLLVSVLLCLVGASDSESMQVQVLQDLNLALQTELNVFIDFECCATSEILHKLD
SPRILLSSNSREARDLRIRGNFTESTLIIVSVMDSDLNPLVASLLPRLLDELHELHIVFLSNEE
PGFPKQDLYTYCFKEGFVNVILMSGKGLYSYLPYPSIQPISLSNVSEYFDRARIIRNFQGFPV
RILRSTLAPRDFEYSNEQGGLVRAGYLFTAVKELTYRYNATIESVPIPDLPEYDVYLAVAEM
LHTKKIDIVCYFKDFSLEVAYTAPLSIIREYFMAPHARPISSYLYYSKPFGWTLWAVVISTVL
YGTVMLHLAARGARVEIGKCLLYSLSHILYNCHQKIRVAGWRDVAIHGILTIGGFILTNVYL
ATLSSILTSGLYDEEYNTLEDLARAPYPSLHDEYYRSQMKAKTFLPERLRRNSLSLNATLL
KAYRDGLNQSYIYILYEDRLELILMQQYLLKTPRFNMIRQAVGFTLESYCVSNSLPYLAMT
SEFMRRLQEHGISIKMKADTFRELIHQGIYTLMRDDEPPAKAFDLDYYFFAFVLXTVGLISS
LLVFFAELVSGHL
>DmelIR62a
MYLQFLFALFLSRYQIVATENFDRAFELALFLDRIGRVHRLHAITIVNSLGSVDPSYLDDLH
RGLMCNSSNHFYMLPQMTATDKDSSHVHFSSLQDEETIYLVFARDSKDAVIYLQAERARG
RRYTRTMFLLRKQESQKDIKYFFELLWKLQFRSALVVVAARNFYQMDPYPTVRVIRMRRL
SSYDPHHVFPPANRKNFRGYRMRLPVQQDVPNTFWYKNRRTKAWELAGLGGILINQLM
MHLNVTMDLFRFEVNGSSLLNMAALTDLIVKGKVELSPHLYDTLQSNTSVDYSYPTQVAP
RCFMIPLDNEISRSLYVFLPFSLTMWLCLLFVLLVVHFVYVRRLIPDGHFWAILGVPGAGQ
VRYGNRKPVRRFSTFLILFGIFILGQTYSTKLTSSLTVTLIRRPDNSLEELFLLPYRILVLPTD
VYAIVDSLGHAEQFSTKFSCTDAENFSQKRISMHPEYIYPISTIRWRFFDMQQRFLRKKRFY
FSKICHGSFPYQYQLRVDSHLKDALHRFLLHVQQAGLHDLWLDTCYRKAHRMGYLKDFS
TLAELEEKLRLRPLALNLLVPAFSLFLCGMLGSGIAFLVEIRHSFGCRQKPPSINRNPGD
>DmelIR67a
MLPILVPVLLLFNETSWINPILTSIYKDRHHETVLLLQHSQHGNASGLERFPWPVFSFNEQM
DFYVRGKYNSEMLVLIWQTGNSDWDLDLWQALDRSLLNMRKVRVLLLRKWEKIPTADV
AATAEHLLFLHVAVIGQGNRIYRLQPYAPQSWLQVDPIESPIFIKIRNYFGRYIVTLPDQFPP
RSIVYRNPKTDEIQMTGYVYKFLLEFIRIYNFTFRWQRPIVQGERMNLILLRNMTLNGTINL
AISLCGFETPSXLGVFSDVYDMEEWYIMVPRAQEISIADVYVVMVSGNFLIVLIIFYFIFTIL
DTCFGPLLLKERVDWSNLMLNERMISGIMGQSFNMSARNTISSKVTNATLFLLGLVLSTLY
AAHLKTLLTKRPTSQQISNFKQLRDSPVTVFFEEAERFYLKHAWDRPIRYIKDQLNFRETIE
YNALRMGLNRSNAFSALTSEWMIVAKRQELFKQPIFTVQPELRVIQTSVLLSLVMQSNSIY
EDHINDLIHRVQSAGIVEYWKHQTLREMITMGMISQKDPFPYVAFREFKVGDLFWIWLLW
VSFLFMSFVIFLCELLVDCFISKTLIRNKRPH
>DmelIR67b
MELLYLNTLQSLSLLEGNRLVQTVQELNNIYQTELNVFLEFGNGADILESAQGTFVPTLWI
KNPQNQKVMKGNFTSCTLTILYLEDEHLDRGLYYLANWLWEYHHLEVLIFFNGGSYDKLI

QIFSRCFNEGFVNVLVMLPGSDELYTFMPYQDLKILNLKSIKEFYSLSRKKMDLNGYNITS
GLVIAGAPRWFSFRDRQNRLILTGYMLRMIVDFTNHFNGSVRLMNVLTVNDGLELLANRT
IDFFPFLIRPLKSFSMSNILYLENCGLIVPTSRPLPNWVYLLRPYAFDTWIAWLIMLIYCSLA
LRILSKGQISISAAFLKVLRLVMYLSGSRDMGTRPTTRRLFLFVILTTSGFILTNLYVAQLSS
NSAAGLYEKQINTWEDLDKSDSIWPLIDVDIKTMEKLIPDRTKLLKKIVPTLEADVDTYRR
NLNTSCIHSGFFDRIDFALYQQKFLRFPIFRKFPHLLYQQPLQISAAFGRPYLQLFNWFVRKI
FESGIYLKMKDDAYRHGIQSGLLNLAFRDRHLEVKSNDVEYYYLIAGLWFGGLTLATVCF
LLELLIGYAKIKVTISCKMNIM
>DmelIR67c
MFCWLIFLNIILLSDRSESWSAREVIHQFNHDQQLQLNIYLDCNDVELQIGQEVSNLFVNS
TADKMKILGRFSSHSLIIACFKDSTRNRTLNGVKELLWGLQYLPILFVVDSNMDFYFQQAL
RHGFIHVLALNFMNGSLYTYKPYPKVEVHQIKDMQKFYKLTKLRNLQGQAVRTTVETMT
PRCFRYRNRHGQLVYAGYMYRMVKEFISTYNGTEEHVFGNVDTVPYKEGLAALKNGEID
MMPRIIHALEWYYFYRSHILYNIKTYIMVPWAEPLPKSLYFIQPFRGTVWITIMVSFVYASI
VIWWIRYRQQGNSSLTQSFMDVLQLLFQLPLSKIWHFNMGTHQVVSFIVLFVFGFMLTNL
YTAQLSSYLTTGLFKSQINTFDDLFREKRTLLVESFDAEVLHNMTKEKIIQKEFESIILITSIE
EVFKHRKSLNTSYAYEAYEDRIAFELSQQRYLRVPIFKILKEVYDQRPVFVALRHGLPYVEL
FNNYLRRIFESGIWIKLQEDSFLEGIASGEISFRKSKSREIKIFDKDFYFFAYILLGMGWCVS
TIALFLELWSFKYSVTNVLHEG
>DmelIR68a
MRCLWILIVAFISLAMATSIPIPIANPAPLSGYEMQLKILLQKILWVANVKRCFAVITDDLHY
PIYDRIFFESVGRRVIPFFVMRTNESDDLQRPSRQVELFVKAIKSSDCELNVITILNGWQVQ
RFLGYIYDNRSLNMQKKFVLLHDLRLFESDMIHLWSVFIDAIFLKRQLDNKYTISTIAFPGI
LSGVLVMKNIANWELGKGLNGRILFADKTSNLFGTSLPVAISEHVPMVLWANATKSFQGV
EVEIMNALGKALNFKPVYYKPNQTENMDWTELDGGASVAYGSGNPDGYAQNGTHIDSM
LVDEVAAHSARFAIGDLHLFQVYLKLVELSAPHNFECLTFLTPESSTDNSWQTFILPFSAGM
WVGVLLSLFVVGTVFYAISFLNAIINGNVSSEFFRCLRPNRNVPMDPKIYRRISFRIAISRYR
SSKGDRMPRDLFDGYTNCILLTYSMLLYVALPRMPRNWPLRVLTGWYWIYCILLVATYRA
SFTAILANPAARVTIDTLEDLLRSHIPPSTGATENRQFFLEANDEVARKVGEKMEVFGYSDD
LTSRIAKGQCAYYDNEFYLRYLRVADESGSALHIMKECVLYMPVVLAMEKNSALKPRVD
ASIQHLAEGGLIAKWLKDAIEHLPAEALAQQEALMNIQKFWSSFVALLIGYVISMLTLLAE
RWHFKHIVMKHPMYDVYNPSLYYNFKRIYPQH
>DmelIR68b
MKFLVGLLLQWYLPGIYALAEIACRIAVEQNVQVTYLYRCASCPASFDADYSALELDLYRC
VGSRLPVITRNMEAHELEPFRRTDSLSIFQIPAAEKGDSLVRRILDMLNPHQRRKHMHKYL
FVWPNAGRHQLLRLFRGSWAKKLLYGLAITGRENGTFDFDPFAWGGLQVIQRLDGEVPY
ARKVKDLRGYPLRFSMFTDPLMAMPRSPVETAGYQAVDGVAARVVGEMLNASVTYVFP
EDNESYGRCLPNGNYTGVVSDIVGGHTHFAPNSRFVLDCIWPAVEVLYPYTRRNLHLVVP
ASAIQPEYLIFVRVFRRTVWYLLLVTLLVVVLVFWVMQRLQRRIPRRGVIQFQATWYEILE
MFGKTHVGEPAGRLSSFSSMRTFLMGWILFSYVLSTIYFAKLESGFVRPSYEEQVDRVDDL
VHLDVHIYAVTTMYDAVRSALTEHQYGLLENRSRQLPLGIATSYYQPVVRRRDRRAAFIM
RDFHARDFLAITYDSQAERPAYHIAREYLRSMICTYILPRGSPFLHRLESLYSGFLEHGFFEH
WRQMDLITRVGASPDAEEFLEDLGDQTDTDSGSNELAIRNKKVVLTLDILQGAFYLWSVG
IGISCLGFAVEHAHWFWRRQTLRNAVEARTS

>DmelIR85a
MSIQWLKHILLLAILVNLAGTRENHIPLDLKKSSIVMVKMSQILCKARIKVLFVYFENQTS
HEHTGQILKEVTKCDISNQNTPLEAVKDDGILMYMVMITTNISQPLELSLIRKKSAAKHRS
HVFLLVRDADTVSDAWMRASFRQFWKIWLLNIVILYWRDGRLNAYRYNPFMDNYLIPVD
NKPNEVPTLEQLFPKTIPNMQRKPLRMCIYKDDVRAIFWRQGTILGTDGLLAAYVAERLN
ATMMITRPHSYNNNHNLSSDICFLEVAKEYVDVAMNIRFLVPDTFRKQAESTVSHTRDDLC
VIVPKAKTAPTFWNIFRSFGSLVWALILVSVLVANVFCYILKSEVGRVPMQLFAGALTMPM
TQIPPNHSIRLFLIFWLYFGLLICSAFKGNLTSMMVFQPYLPDINQLGALARSHYHIIIRPRH
VKHIQHFLTLGHKHESRIREQMLEVSDTQMYEMMRNNDIRFAYLEKYHIARFQVNSRVH
MHLGRPLFHLMNSCLVPFHAVYIVPYGSPYLGFLDSLIRSSHEFGFERYWDRIMNSAFIKS
GVKVVNRRRGSGNDEPVVLKLQHFHAVFALWLVGIGMACIVLAWEHLTHNYNLAVTKRR
D
>DmelIR87a
MSTPEQRFWLAALLFLLSQHSEVRGFGINLMKVQTEDKGQEACILALLRKYFDSGDGLSG
SVLCINRNYQLPNIEEQLLRGVNNYENYPWSLLITNSREGPSPAKFLMNEKPQCYFLIVDN
LEDEDLDEVFEHWKGMVNWNPLAQFVVYLASLEETDEEMNDLMVELLLTFINKKIFNVN
VIGQSEENQFYYGKTVFPYHPDNNCGNRVISVELLDACDYPSEETDSEDENDEDEGDGAQ
EEDDGPQEEGDGEQEEEDGPQEQEDGDQAKGDEGQENDDGGLENKVENEFRIGASDDD
ELENDLSSNSSEPEAIIEEFFRAKFEDKFPRDLSGCPLTASFRPWEPYIFRNSEEQPVDDYYY
GLQGDEDDYNDTSPNYGESDDESYADPGEDGDGAIPDTETQSGGKLKLSGIEYEMVQTIA
ERLHVSIEMQGENSNLYHLFQQLIDGEIEMIVGGIDEDPSISQFVSSSIPYHQDELTWCVAR
AKRRHGFFNFVATFNADAGFLIGIFVVTCSLVVWLAQRVSGFQLRNLNGYFPTCLRVLGIL
LNQAIPAQDFPITLRQLFALSFLMGFFFSNTYQSFLISTLTTPRSSYQIHTLQEIYSNKMTVM
GTSEHVRHLNKDGEIFKYIREKFQMCYNLVDCLNDAAQNEHIAVAVSRQHSFYNPRIQRD
RLYCFDRRESLYVYLVTMLLPKKYHLLHQINPVIQHIIESGHMQKWARDLDMRRMIHEEIT
RVREDPFKALTFDQFRGAIAFSGGLLLVASCVFAFELCYVKYVYRTEKRERKTKKITKKVH
NIKIQHD
>DmelIR94a
MALPKQLKFINIFLVLLIIYGSSDGTENQHEIFLNRLLQAVHNERSVETLFLLHHSNLANCS
LQDWNPPRIPTIRSNELTVFNVEKTFNHNALALVCLMKNSYREILNTLAKSFDCMRQERIIL
MIHRKSDSKFIEDITHEVKNLQFLHLIVLIVQEKYNGQVFASTLRLQSFPEPHFKRIRNVFAI
QRIFYRPINFHGKVLNAIPNDIPILFVALNEMFTEYARRYNSTLRIQNRTIKEDIEITEDNYDI
DMKIQLHNSQNFLHHMNIAMDIGSNSLIILVPCATELRGLDIFKELGVRTLTWLALLFYIIFV
LVEMLFVFISNRFNGRNFTMRYTNPLINLRAVRAILGQTSPISNRYSLSIQHFFVFMSLFGTL
FGGFFDCKLRSFLTKRPYYSQIENFSELRKSGVTVVVDHTTRQFIEQEINANFFRDEVPNVR
TTTIQELINHVYSYDRKFAFVANSIPWRTFREEMKSINQKILCDSKNLTILENVPLTFSIRRN
AIFSHHLRNFIINAADSGMITCWFKMAGKVIRKHIKTTLRESEQQPSHLPLSFDHFKWLWA
VLCIAYVMSFMVFVMEILWSKYQRRTRSVSIV
>DmelIR94b
MSLIFNLLFILILSQAVSQETEFLQLKYLNNIVRSMIKLHKMETLVIVKHHLDNNCSLQNW
NAHGMGIIRTNDQGKLIMKDTFNSRTLAIICIGQNSHITLLRNVFETFGKVQQKKIILWTQM
ELKEKFFQEISKKSRDLKLLNLLVLKAVTKDKLLIYRLNPFPSPHFKRIENIWTPNDTLFMD
TKFNFHGMTAVVKHDYNWTIQMGNIRKFPISRIEDKEVIEFALKYNLTLQFFNDVERFDIEL
RKRIILKSNSTQPIDSGIPMVFSSLLIVVPCGNYLSIQDVIKVSGIEKWIFYIILVYVIFVLIEIT

FLGVTILISRQSRHQMIPNTLVNLCAFRAILGLPFPETRRTSLSLRQLFLAIALFGMIFSIFINC
KLSSMLTNPCPRPQVNNFEELKTSGLTVVMDHDAENFIEKEIGVDFFNQYMPRKVTLTFTE
RAKLLFSLKGNHAFTLFSESFAIIESYQRSKGLRAHCTSEDLIVAERVPRIYILENNSILDRPL
RRFIRQMQESGITNHWLKNIPSSLEKNLMQITIPYDRERVHPLSIEHLTWLWCILILGYSISM
IVFFVEMSLKRRKKNLENRAPNICIC
>DmelIR94c
MSKVFKLLVLPLIYLSLTKGSKNPQLKFLRELINVIEEGREIRTIMVIKHSRDEYCHLDQWN
PRGSPILRTNEMGSIRISGYFNDQAVILACMGENSDYGLLKSLANAMDNMRQERIILWSER
EPTKMLMDYISQQADRYNFAQIIIVTMNEDVDAVPSLHQLNPYPTPRFRQITNISNIRRTSFF
GCGLSFQGKTAILKESVVSNIRFKVWSPSGPIPLSELKDYEIVQFAVKYNLSLKLYDQNESK
SDHFDIQLGPLFITKDFPTQMAFVSPNTACSLIVIVPCSPKWRFMDVLHKLGVLKLIGCLLI
AYAVFVLIETLILWLTHRISGREVRLTSLNQLLNPRAFRGILGLPFPEFRRSSISLRQLFLVISV
FGLVYSNFVSCTLSALLTKPAQNPQVRNFKELRDSGLITIMDKYTHSFIEKHIDPEFFDHVL
PHYLILQKKEALRMIWNFNDSYSYVMYTTTWKSLNTVQKSFDERVFCESESLTIAWNLPR
MYVLGNNSVLKWMLSRYITYMPQTGIPDSWTEQLPKVLKLLYNVTSPRRIKEGAVPLSIQ
HLSWIWHLLFIGESIATLVFIVEILLQKSNQHTSNMRERSSEDDDFV
>DmelIR94d
MGQLHLLLVALVLLSPGGDSFYHSLIHHLNRELKIEYVLLLGNFDTTWLDILWQLPVSVLQ
IKEHSRETYSLLENPSHNVLTIAFVNDSPEDILEILYRNLRMLNTQPVLLVIRKSTIRVNSLLE
WCWHHQLLKVVAIAQDFMESLIVYSYNPFPVLQFIERRLDNSTVIFEKRLENLHGYEVPIA
LGGSSPRLIVYRDLEGKLIFSGPVGNFMKSFEQRYNCRLVQPYPFDESAISPARDLIASVQN
GSVQIALGAIYPQVPYTGYSYPIELMSWCLMMPVPEEVPHSQLYSMVFSPMAFGITIVAM
VLISLTLSMALRLHGYRVSFSEYFLHDSCLRGVLSQSFYEVLRAPALIKAMYLVICLLGLLI
TSWYNSYFSTFVTSAPRFPQLTSYESIRHSNIKIVIWKPEYEMLLFFSENMEKYSSIFQLQED
YKEFLHLRDSFDTRYGYMMPMEKWSLMKEQQRVFSSPLFSLQDDLCVFHTVPIVFPMVK
NSIFKEPFDRLILDVTATGLLSRWRDMSFTEMIKAGQLGLEDRGHPKEFRAMKVGDLIQIW
RFVGWMLGLATIVFLLELICFWRHKMWQNMKYMFCRNKNI
>DmelIR94e
MDCPKWILSGLCLISLVSGATVIELLGTLKLELDFEYVLLMKNRNFSLSDQVWNGTSLTKD
VMDEVQVPVLQFNENVSYFLHNSISRRLVTLGFMSDANLDEHRGLLTALVANLRHMTTSR
VIFLVQSKASTDFLYELFRNCWRKKLLNVIVIFQDFETTSTFYSYSNFPILQIEERIYETSLQT
LPIFPDRLRNLHGYEMPVILGGTAPRMIAYRNKKGNVVYDGTVGHFMTAFQQKYNVKFV
QPLQAKNPLDFAPSMQTVGAVRNETVEISISLTFPTIPPFGFSYPYEQMNWCVMLPVEADV
PPFEYYTRVFELAAFLLTLGTLVLISCLLASALSLHGYATNISEFLLHDSCLRGVLGQSFVEV
FRAPTLVRGIYLEICVLGILITAWYNSYFSSYVTSAPKQPPFRTYDDILASKLKVVAWKPEY
AELVGRLLEFRKYETMFLVEPDFNRYLALRDTLDTRYGYMITTNRWVLINEQQKVFSRPL
FQKRDDFCFFNNIPFGFPLHENSVFMEPVQKLIMELAETGLYYHWITTGFSELIDAGEMHF
VDLSPHREFRAMQIQDLQYVWYGYAFMVVLSSLVWLLENLAYTVKSKTIFPTHFMQRNK
K
>DmelIR94f
MSGMWQQVLLAETSNWFRSDVLQRFWTHLRVEIRFRTMLNYRLESCDCWFDNVLGSDN
STALLWNDQTYPHYLRRRQDTDILVVSCLRFHQYQEVLLALSLMLDQMRSMPVVLQLCG
DEDSMQELNSARLLLKHSQDLKMPNVVLLSSTFFTSATLYSYEMFPEFNVQKLVYQAYLT
LFPYKLGNLKGHPIRTVPDNSEPLTIVRKTLNGSIAIDGLVWQFMIEFAKHINATLQLPIEPH

PEKSIKLVQILDLVRNQTVDIAASLRPYSLNVQRSSTHIYGSPMMVGNWCMMLPTERVIGS
HEALTRLMKSPWTWLILLLFYSVHRFLAQKTRLRSSLIHLIKLLINLSLICFLQAQLSAYFIG
PQKVNHISNMQQVEESGLKIRGMRGEFMEYPIDMRSRYASSFLLHDLFFDLAQYRNSLNT
SYGYTVTSVKWELYKEAQRHFRRPLFRYSEEICVQKLSLFSLIQQSNCIYCYRSRIFILRMH
EAGLIRLWYRRSYYVMVTAGRFPIGDLSTVHRAQPIRWTEWQNVVLLHGVGLLFSVVVF
VIELTVHYANVCLNNL
>DmelIR94g
MSTAVNSVHSKLVSLISRGQELTSIFFYAPAKEKCHLEDTISSATWGLPLVIWRTDRTVILNG
FIGEGLLVLACLPGFHWRALLGSLARSLKYLRQARILIELMQDRDEFLVSEVLQFCLSQDM
INVNAIFDDFPETENLSSFEAYPSFEVVNQTFTPDTQVSDLYPNKMLNLRGGVIRTMPDYSE
PNTILYQDKEGNKEILGYLWDLLEAYAHKHNAQLQVVNKYADDRPLNFIELLDAAQSGII
DVGASIQPMSMGSLSRMHEMSYPVNQASWCTMLPVERQLHVSELLTRVIPYPTLALLLLL
WIFYEVLRGRWRRHSRLQSIGWLVLATLVSSNYVGKLLNLFTDPPSLPPVNSLAALMESPV
RIISIRSEYSAIEFTQRTKYSAAFHLALHASILIGLRNAFNTSYGYTITSEKWKIYEEQQKRSS
KPVFRYSKDLCFYEMIPFGLVIPENSPHRAPLHSYTLLLRQAGLHDFWVNRGFSYMVKAG
KINFTAVGERYEAKTLTITDLRNVFIIYVSVLLISLILFTCELFVSWVNYWLGF
>DmelIR94h
MLSNISFSSAPELVDLYGLVLKFLVSSETTLFYFNPTGQKCSWETLPRTILSNHPQIIWFREE
TYPGLYKRHSSNLFVMACLSSTSYDGQLQLLAESLTRYRSVRVLIEVQDKEGSFLASQILL
LCQQHSMLNVVLYFSRWTRTLNVFSYLAFPYFKLLKQRLSGSLRPKIFINQLKDLQGYKIR
VQPDLSPPNSFSYRDRHGECQVGGFLWRIVENFSKSLKGDTQVLYPTWAKAKVSAAEYMI
QFTRNGSSDIGVTTTMITFKHEERYRDYSYPMYDISWCTMLPVEKPLSVEILFSHVLSPGS
ALLLILAFILFFLIVPQLIKCLGITFRGRLIGMASRIFALVMLCSSSAQLLSLLMSPPLHTRIKS
FDDLLTSGLKIFGIRSELYFLDGGFRAKYASAFHLTENPNELYDNRNYFNTSWAYTITSVKW
NVIEAQQRHFAHPVFRYSTDLCFSSETPWGLLIAPESFYREPLQHFTLKINQAGLITQWMT
QSFHEMVRAGRMTIKDYSRTNLMKPLRIQDLRKCWVIFAVGLGTSTVVFTIELLLIYTNVF
LNSL
>DmelIR100a
MATTLQLIMLALVGGTLGQANNTDHKQVLTSIVKQLEGGLELHLRTSEDGGNDLVQFLM
QEKSSIIISAKQEEVPSRAKIMRHHFFIFDGVHQMQEIRTSLFNTDGFYILALENNTIEDDVL
LMEFAADVWLQHGHSRIYYVQLSKKSVLLFNPFLQRLVVVQDSKTYSRIYKDLEGYHLRI
YIFDSVYSSVIGDGENKVLSVTGADAKLAKTVARQLNFTADFVWPDDEFFGGRLANGEYS
GGVGRAHRGEVDIIFAGFFIKDYLTTHIQFSAAVYMDELCLYVKKAQRIPQSILPLFAVHMD
VWLCFLLVGLLGALVWLILRAVNLILGIEGVPDGSRATRISYFGAARRIFVDTWVIWVRVN
VGRFPPFHSERIFVASLCLVSVIFGALLESSLATVVYIRPLYYRDVNTLRELDESGQPIYIKHPA
FKDDLFYGHNSEVYRRLDAKMMLVAEGEERLIEMVSKRGGFAGVTRSASLQLSDIRYVM
TKKVHKIPECPKNYHIAYVLPRPSPYLEEVNRIVLRLVAGGIVGLWTGEAKERAKWSIQRF
PEYLAELDVGRWKVLTLSDVQLAFYALTIGCLLSAIVCMAEILLGRQRRLHSPK
>DponIR56e.1
MAYNYNQGPYAGQQQPGYGPQPGVYPPPPQGYPQGGYPQPGGYPSAPQPGFQPPPYGDP
YGGANQGYGGYDAEDPEVKGFDFSDQSIRRGFIRKVYSILMVQLAITMGFIALLCYEPKT
KAFVHNTPSLFIVALVVMIVAMITLACCGEVRRKAPINYVMLFIFTIAEGFLLGVSASTYKQ
DAVLMAVGITAAVCLALTLFAFQTKYDFTMMGGVLLVAVIILLVFGIVAMFVHNKIVQLVY
ASLGALIFSIYLVYDTQLMMGGKHKYSISPEEYVFAALNLYLDIVNIFMYILAIIGHARD

>DponIR75x
PAAKSIRNVIYSRRRAFLDKVRLHKAILEENLNMRVVFAKARVDAFENINTKRYEKFTYFL
DMQCPDSADILDKASNVNRFGKFEFIYSWYLLSKVNQELEDIFHIFTRFKTRMDMDVKVL
AVDDFGRFEVSEIFNPGINVGLTTRRIGKLQNGSVIIDKNWSYYESRMNMTGVLIRSANVI
RYPFTTSFDEYMTDPKLMKYDIYSKFHYQLFQGLVHIHGFEYNTSLPFSWFGNTSSGEDG
GLAKMLWDDTIDISSAGCILRLLDSDRIDFYDYIMPYYKFRSCFFPQSRSRKAELLRSIKAV
>DponIR76b
MGLMEVVLTTLATLCFNSTCVDQDLINASKQRLAHLKEELKHETLTVTTLKNGPLSGYEI
VNNTVIGTGVAFEILNIVQREYGFKYNVIVPDHDSFEPVNGGEGGVRNMLLNETIDVAVAF
LPQQYTDVVSYSRSLDTAQWVVLMKRPKESASGSGLLAPFTATVWSLIIISLLGVGPILWL
TILLRARMCKEDHDIVFSLPSCMWFVYGALLKQGSTLNPRTDSSRILFSTWWIFITILTAFY
TANLTAFLTLSKFTLPISEPKDISRKHNKWITNRGNGIVEQLYLSKKYANGDGNSLFEEIGM
PQWEPDVDEDTMLSTYVIKQNMMYIREKTVLESIMYEDYKVKTKADVEESKRCTYVITK
FAVCVFPRAFAFRPGFKYKELFDFTIQHLSESGITDFQQRKSLPDTTICPLDLGSKERRLRNS
DLAMTYMIVGGGLIISTIIFAVELIIYYAKMHCFNKKSHVNNNNTLVTQSNNGLFVKNHQH
QGNFRASKQFVSPPPSYHTLFHPPNLTNGEYKNKTINGRQYWVFNDKQGMTSLIPQRTPS
ALLFQFTN
>DponIR75sFIX
LVVLYFLRKWEFAKIKQQELLHKTAYPDVVFITIGAVCQQGAATLPHSVPGRIATLWLFVSL
MFIYVSYSANIVALLQTSSNSIRTLEDLLKSRIPIGVDDTIFNHFFFTTTQEPTRRALYEKKVA
PPGKRPNFLSIEEGVRRMRQGLFAFHMETGAGYKIVGETFTEDEKCGLQEIQFLQVVDPW
LAIQKNSSYQELLKIGFHLLRETGIQQRENNLIYTKKPSCSSKSSTFFSVGLVDCYPAVLIFA
EGLAAGLALFLLELYIHKKFSNQ
>DponIR75p.2
MANFRQHHNFKGQTRLFPHQQLRSGYQRINRQNLEDSSPVPAKELLELFGKLHSRLVRRS
GLYLLQPANFDKNRTNFHKMPLKVTYVVSDNHSLDHGIYDNRNKHIDKATKINYIMYEYF
VDIWNMSHEVVVTNEWGQEFHPSKLYYKGLLGDLCHEKADAAGTVMFTPTERLKYFKF
LVSTTKEMSLHFIFRAPPLPYSHNLFALPFDTNIWISCAIVLSLCGLVIWIIMSWEAKVASFA
ANRQMHNENAASFLDIVMMQIGVVCQMDYFHEPRSTAGKIATFSLLLGFSYLYNAFCARI
VVLLQATANNLHDYKALYEAKMDMGVEATSYNIYYFSHPNSRTNEDYRKLIYQKKIAPN
NKFLPATDGMRLVQNSYFAFHVELTTASDLILATFNNQEKCAVRKVNSIFKEDKPYLASPIN
STVTEYLMIGFHRLFETGVHSRESRRRFNKLPPCRGRNSAFISVGLIECYFAIEIFLIGFTLCL
AFFLLELASGVYRKNQST
>DponIR75p.1FIX
MSWTILCFLKMFSVCLSSSPAHADTDISFLFDFLQQTNRPNYAVLENVCWSKEETQKLHR
NLTMQNFKCKSVDGNSTIMTERYYEHSYIILVHVDCDFDTIWKQAAKGSLTFYPHIWILLG
DFEKIKKKNVHIPMNSLLLSLVRTANYTKLETAYKIKKTVDWYIERTVGNWSPKVAMFDL
EKVNIFKDRGNFMKVPLRVTYIITDNSTVNHFIDYRNKHVDNLTKINYVLYILIFDILNATQI
RLFSRGWGFESRENNGSFASGMFQDLANDRSDIAGTLAFTPSSRLKYFRYIYPPAKDMDIC
LVFRAPSLAYYTNVFGLPFNNWVWVALGLQLLCGCVLIFIIFKWEWKVAQGRKENGPSFL
DTAMMQIAIACQQDFFHEPKSISGKMATLSILIFFTFIYTAFSAKIVLFLQLSTNKINDVGSV
YAAGFDFAVEDQPFNQYYFKGPSDRAEEQLRKQIYEKKIRRSHGDQFVSAEKGIQLVRDTF
CAFHVERTVAHYLVDKTFSNNQKCSLRFVRSIFKSDMPYLSIPWNSSYIKYFIISFRRLAET
GLQDRECKRCYAKKPSCEGKANTFVSVGLIEAYFPLLIFGVGISLCISILILEKLVHKYIKLH

>DponIR93a.1FIX
TKWTIEAGETWGREYQMLDEATNAELLAVGTWRPSDGPNMIDALFPHVAHGFRRKLLPL
VTFHNPPWQILKTNSTGDVVEYGGIVFNIIKELSKNLNFTFNVATVKPQSLLNASTLQSPKG
DTDSSANFNGNSYITTYRVPHSILEMVHNKSAALGACAFTVTEENQRVINFTDPISIQAYTF
LAARPRELSRALLFISPFRGDTWLCLSATIISMGPVLFYIHKLSPVYEYKGVRCKGGLATIQ
NCIWYMYGALLQQGGMHLPYADSARIIVGSWWLVVLVIGTTYCGNLVAYLTFPKIEVPMT
TIDDVLAHKEMVSWSYAKNTLFEARLHNSVDKSFNIIFKDAKNIWDRKAMMGEIKSGKH
VYIDWKIKLQYMIKEHFIDSGECSFALGVEEFCEEQIALIVAPDTPYLHKINEEIKKLHQVG
LIQKWLSDYLPKKDKCWKKKRTIEVNNHTVNLDDMQGSFFVLFIGFLIAVIVISLEMLWSR
KVTNNRKRKVVHQFVT
>DponIR64aFIX
MDNGIDVQNHRERTSDTSWSLSTICTFGVFCQQGIISVPRCLGGRTSAIVALWCGLVIYQFY
SASLVSFLLNVPVNVLSTVQDILDNGFDVGYERVLYAMSLLKAATNPAAQEVYRRVSNKN
QTGYLRREQGLELVKNGRYAFHVELVTGYPFIEQHFDESMICELKEISLFPAMYMYSGYQ
KWSPFREFLDACLHRLEENGVVSRELYFWHPRKPQCMRSRSTI
>DponIR21a.1
RKVDYVPDVESGLRNVTKAFFWPYALLGSREELQFIVKTNFSLGSKKSMLHISQQCFVPF
KVGIALPHHLVYSEILAGGIQMILQSGLNIKMKNDIEWEMLRSSTGKLLAANSRSGTLTILS
RDDRALTLDDTQGMFLLLAIGFLAGGGVLISEIFGGCFNLCKKIDNSRATSSNSSIPSNPRFH
ERQTIRERNRSISLASFQQRHNSIQSEIAFEKAQAEEHHQGGLVECQIHGTTPDQNSQGVVL
EETNADIDYNEQISKLFEQALGEETCGSRPLRA
>DponIR93a.2
MWVRLVICLGVFCKVTNSDIFPSLLTTNASIAIVIDRNYVVEEYEPIKSKIEDYLVYAKREIL
KHGGVNTHLFAWSAINLKRDLTFLLSITSCTETWKLFESADTESLLHIAISEQDCPRLPQHS
AITIPIIDRGQDTPQLLLDLRTVGIYKWKQVVIIYDNTITNDLLTRVIKSMTKQVNRIDASGV
SLVQLAKKVSTTRDVIIANLRTELSKIDPRIMGNNFLVIVSYELAATIMKCAKELNMVNTQ
TQWLYVISDTNSSTKSMNRFKTFLNEGDNIAFIYNTTDVKNVCLGGTICHTEESITGLMKA
LDSAIMEEFQMASQISEEEWEAIRPTKNERRKYLLRKNSEYLSDYGTCDNCT
>DponIR75q
MNTLYFGFFMWFALICGGHCGVHADDVLLLIEDLLDFYRFSKKVYTHVCWDKELQLHLP
RFALHAKYHEFQSTKSQVVILMDMSCKGADFFLENMQKRNLLTPRVILLLINPAALTPHYF
PVNSNALFLKPSGEGFAISKIYSGVSNVTIGTWTKTHRYIERPIAKKKLRATNLKVCYLVGD
KHEGLESSEYQPVAEATTKLNRVLLEDGIRMINSTKTDAFQIGSGEPELVNDLIAGKCDIGG
TPLALTAEKIGRLDVLAKTLREDKTFVFRAAPHSYISNVFTLPFDSYVWCSCFGLMGVIFLI
VHLVVCWEWKDPVFKLNLQPNISLRPNPVDILLMEVGSAAQQGFEAEPRSNSGRIVFMST
LISFMFLYTSFSASIVALLQSTTDSLNTFDNLFQSRINVGFKQNISLDFFNDLKHQPNRADY
HGKLKEPQFFTLEEGVKRLQNDFFAFYAETSEVYRYINRWFQESEKCSLREIPFKNTHINH
WLFMGKNSQYRDALRIGMNSIQERGIRSREYKRLFPLKPFCDSIGGNFESVGLVDSYGAFL
IMVYGVALSSLLFLLEHMSLQYNLSTKAGRIRNRIFNSIHRGDE
>DponIR41aFIX
MGSEMKILETFSQYVNASISPVINQADYWGEIWNNWSGSGLMGNLVEDKADIGAAALYT
WEFAYEYLDLSKPTVRTGITCLVPAPKLSAGWLTPFRVYSLEAWMALIGTLALSFLALYAL
NKLQISVKPQLKSKHHINQLKGKLLSKTLMSVSKPFVMQSITNKEMAQGNLAKYLMGLV
FLSTLVLSTTFDSGLATIMTVPRYDNPINTIEELAESGLPWGGTQDAWILSINNSLEPNLMK

LVARFVAHSEANLRKYSLGDQFAFGVERLPNDNYAIG
>DponIR56e.2
RADLTSITSARNMWKKVVLLAAMLTITSQVDFSSIEFIEAVGRNRRATVFGCSRKDLVETS
KLLSEKNFESNWISEWSSSDGRIFHKSRHIFLVNLNCPETMRFLKQAQQWKLFASPFRWIA
FHQATKTPLAIEKTFRNLSILVDSDFNLVTHQNNREISYRKIYKKRIDESTFEVEDFGLWRQ
NSYFDRQTSSLNDRKNLGTVLKACIVITHNDSLNHLEDKRDKHIDSIAKVNYVLVLTMASI
YNISINFSVTNTWGYKDNNSQ
>DponIR25a
MKNNNIVAGGFFSIFLLNVADICGQTTQNINVIFANEEGNFVADKAVTVALNYIKKTSKLGL
SVDLRRVVGNKTDSQNVLDSLCAAYQQMLDDNNPPHLVLDATRAGLASETVKSFTAALG
IPTVSASYGQQGDLRQWRNLQPNEEEYLVQISPPGDIIPEMVRTLVLNQNITNAAILFDDSF
VMDHKYKALLQNVATRHLIDEINEDVNKIPDHLESLVKLDLKNFFVLGSLQTIKNVLEAA
EKKSLFNRMFAWHVLTKDPDDLKASIKNATIIFAKPIVNNLYQDRLRNIQTTYQLSSVTPEI
EAAFYFDVALKGFLAVKEMLLDGSWKKNNVTNYVTCDDYEPKYSPKRFNLNLRSYLQK
ESSEPPTYGPFAIESNGMSFMEFSMALSAVYVRSGASDKSLPLGTWHGGFNNNMTLLTPK
DMKNYTADVVYKVVTVVQKPFIYRDDTAPKGFKGYCIDLIDEIAKILHFDYEIDAVADGM
FGNMDENGKWNGIIKDLIEKRADIGLGSLSVMAERENVIDFTVPYYDLVGITILMKMPETP
TSLFKFLTVLENEVWLCILAAYFFTSFLMWVFDRWSPYSYQNNREKYKDDEEKREFNLKE
CLWFCMTSLTPQGGGEAPKNLSGRLVAATWWLFGFIIIASYTANLAAFLTVSRLDTPIESLD
DLSKQYKIQYAPVNGSSTMTYFQRMADIEAQFYEIWKDMSLNDSLSDVERAKLAVWDYP
VSDKYTKMWQAMKEAGLPPDLDTAIERVKKSKSSSEGFAYLGDATDIKYLEITNCDMAIV
GEEFSRKPYAIAVQQGSPLKDQFNTAILQLLNRRELERLKERWWNKNPEKKQCEKADDQ
ADGISIQNIGGVFIVIFVGIGLACITLAFEYWWYKYRKNTRVTNVAEAPNSRHHKVGGVQ
KGFPRQFEGESDMKITKLYPKTKF
>DponIR8a
MHVLGLFGAVACVLSVRGQQFKIVTLHQPDQAAEVKYFENAFLKVNKDEEIAFLDVLLN
EDESGHYKQICDALSTGFSLILDFAWSGTEVAQDLTSNMSLPYLHVDVSVAPFLVLLDSYL
DSRNSTDVVVVFDKEEYIDQSLYYWLDSVRLRLVMADALNRSTASKIESIRPIPHSFAIVAS
AKNMNKLVSQALNEDLMSLSDRWNLVFTDFETGIFDKSLFQNQTPSLMYLKPELCLDLSI
QSRCPSNFVLKEQFLYWLAWGLSRLAKMAAEESLEFPEKEFQCGKTTFSEDTKERLGDML
DSIVADNSNVLSLTGRSVKVAVRGNVEKMINGSFQTIAQYTNGKLTPEPGKQIDPIRAFYRI
GITHAIPWSFKAQNLQTGEFYWTGYCADFAQKISEVMNFDYVFVEPATGTFGEKVNGTW
DGIVGDLAVGETDIAITAVIMTADKEEVIDFVAPYYEQTGITIVMRKPVRKTSLFKFMTVLK
LEVWLSIVGALIVTGFMIWFLDKYSPYSARNNRKAYPYPCREFTLKESFWFALTSFTPQGG
GEAPKALSGRTLVAAYWLFVVLMLATFTANLAAFLTVERMQAPVQSLEQLARQSRINYTV
VQDSETHMYFINMKFAEDTLYRMWKELTLNASTDDTRYRVWDYPIREQYGHILLAINDSN
PVANASEGFRITNEHLDADFAFIHDSSEIKYEISKNCNLTEVGEVFAEKPYAVAVQQGSHLQ
DDLSKVILDLQKDRFFEQLQAKYWNDSAKGDCPSTDDNEGITLESLGGVFIATLFGLALA
MITLAGEVLYYRRKGKKIKPKKRKAKSKNLPLNTAGLKLSKLDGQSVDMFNVNKTVTIG
TTFKPVNLKENLTKEMETVHISHISLYPKARNRIPRVE

>ItypIR75s.2
STGGAKCLSVTLQCAWTICHVDFIHHINVYVRVLLGQYSGIIADIVKQYKNSGRPFKLQNS
FRSRRHGFQSLFLYYYYRTNQEGNLPKKGGATGQKPNFMPIEDGIKRMRQGLFAFHMETG

SGYKLVGETFEEAEKCGLQEIQFLQVVDPWLAIQKNSSYEEHLKIGLRLLHETGIQQRENN
LIYTKKTDVL
>ItypIR75p
QSRFIAEREMYEENAPDFLDIVLMQIGVVCQISYSFKPRSTAAKIATLSLLVGFVVYIYNAFC
ARIVILLQSTANNLNNYKDLYYSKIDMGVEEAPYNKYYFSNPNNRANEAWRKLIYETKIM
SKNKHPVFYSTAEGLKLVKQSYFALHVEYTTATDVILATFTNEEMCAVRVIESIYKEDVPYI
SCPVNSTFTEYLLIGFHRLFETGLHSREARRKFSKLPKCIGRNSIFVSVGIIGCYFAVEVFLV
GVILSLVIFAVELV
>ItypIR25a
RGNREKYKDDEEKREFNLKECLWFCMTSLTPQGGGEAPKNLSGRLVAATWWLFGFIIIAS
YTANLAAFLTVSRLDTPIESLDDLSKQYKIQYAPVNGSSTMTYFQRMADIEARFYEIWKD
MSLNDSLTDVERAKLAVWDYPVSDKYTKMWQAMKEATLPPDLETAVERVRKSKSSSEGF
AYLGDATDIKYIHMTSCDFVVVGEEVSRKPYAIAVQQGSPLKDQFNSAILQLLNRRELERL
KEQWWNRNEESKQCETSDDQQDGISIQNIGGVFIVIFVGIGLACVTLAFEYWWYKYRKNS
NITNVIVSDPKHRRVAGFPKDVGGKANEGELALRPGKLYVKPKY

SNMP sequences

>CbowSNMP1
MRFPVKLAIGSISAFIFIILVGFVLFPRMITSKVKGMVNLAPGNEIRDMFIKVPFALSFKIYLF
NVTNPMEIQSGEKPIVKEVGPFCYEEWKEKMNIEDKEEDDTISYNQKDTYLKKWWPGCR
NGQEEVTIPHPLILGIVNTVARQKPGALSLINKAIKSIYSDPSSIFLTAKVDDILFDGVVINCN
VSDFAGKALCGQLRTAEALTKVGEVEKFSLFSSKNATLQKRIKAYRGKKNHRDVGRIVEY
NSSKMMDVWPTEECNSIEGTDGTIFPPLTKPGEGLFMFSPDLCRSLIAFFVRKSTYDGIPCG
EFTADLGDMSKNEKEKCYCSTPETCLKKGMMDLYKCSGIPIYASFPHFYNSDTSYLKGVG
GLSPNKTKHEIKILFESITGSPLYARKRLQFSMPLESTQKVELFKNFTGTVLPIFWIEEGVGL
NRTYTGQLKSLFTLTKVVKVSKWLILIGSLGGLAAAGYLFFKVDGRADITPVHEIRRHESK
SGSTVNGAGGHVLSGNGLEKY
>CbowSNMP2
MKMFGASRFCNVKILFVTTVVATVVLIGVLLLSFVGMPLIVNDQLAKKLRLENNTEQWD
RFVELPVPLNLNVFVFNVTNSDEVTNNKATPILQEIGPYCYEERITRKILSANSTEDSITYEQ
SFNITFDEKRSGQWKESDKIVMVNPLFLILSQITNVIERFVVMGCIDKLFPPKYSTMFFEVD
IKTIMLEGIEFGVASDDIGPACNIVRNKLLEKTLPMKNVERIPSPTDPSVINSLKFAFLQYKIR
GPDGQYTTNRGIDDITQLGHIMRWDHSAEIDVWGRGESTNNATCKEVKGSDSTIYPPHVT
KSTKLDIFSTDICRTVQIRYKGTGTYQGDSGYYFGIDENTFRPATPSPENDCYCIQQTMAPD
GEPSCFLDGVVDVYPCFGAPILLSFPHFLYADESYLDGVIGIDPPNSSIHEIFLLIEPNTGTPL
QGMKRIQLNVVLRPVEFVEYTANLPSTVLPLIWIEEGVNLSQDLLDKLDKMYFNVIKAAD
AAKYAAIGVLTAFVLISGGFFVRKRYFK
>CbowSNMP3
MKFYSVLFVVKDRANMLNKFNITVSGKIIVILGVFGLFCIFAGFYVGFKAVPDVITDKIWD
MKVLKENTEQWGMFMKTPFPFTFKVYLFDVQNPQEILQGAKPVLRETGPFVYKVYKWK
SEVEWDTPDDISYFSYMRFEFDRKASGIFSEDMKVTLFNTAYYGMLQKIDETQPEVLSTVE
GVLPSIFGENHGLFIKVKVKDYLFDGLKICENEGKDGGFVAGMVCKQMIARLPESKNLRL
EDNSILFSNMHYKNNTHQGRFTVKSGGQNRTETATLTLFNGKSYISSWTGEKSMCNKIRG

ATTVFPVNIEKNMTFEAYSEDICRTIPLEYSAEETVKDIVGYKFSAMNDSFSSTKKENFCYC
TNTTRTLDGEYGCLKDGVTDLKTCIGSSILVSFPHLLYGDEEYLDSVIGLNPEKSKHETTVI
LEPISGFPLSVTQRIQFNTFLRPIDNVISLENVSKSLFPLLWVEESLILDDQYTDMLKNELFR
TIKIVDIVKWVTIGSGAACVLIALILRMSSKTT
>CbowSNMP4
MRLPLKLGVAGFLLILLSVIVGFIALNPIVRFGIRQQTALKRKSEIRNIYLKLPFPLDFRVYFF
NISNPMEVQKGATPILTEIGPYCYDEFKEKIDVLDNDAEDSLTYYPYDIYKFNAEKSGKLSD
TDYVTILHPALVGMVNQATRDSPALLSIVNKAIGPIFRDPESIYLTAKVKDILFDGVELNCK
VTEFAAKAVCTQIKSQIPGIKSDPEKSIFLFSLLGVKNATVGKSIKVSRGISNSRDLGKVLEF
DGKKVLKLWYEEQCNHFKGTDGWIIPPLLKPEEGLWSFSADLCRNVVAEYVEDSVTKGV
KTRRYEATLADMQNNEEDKCYCPTPKTCLRKGVFDLSKCMGVPILATLPHFLEADEIYLQ
QVKGLNPILDKHIIRIQLEPMTGTPIEARKRLQFNLPVSASEKITLMRNVSTSLHPIFWIEEG
VELDGALLEKVTEVFTFLGVFQVFRWLGLLIGFVSIAYAVYHHMKHSRSVHITPISGSSSSD
HVDINRSTNELVGKMKEVFQSDKGHTNPVMTGHEFDRYS

>DvalSNMP1
MSQHQTPLFKGMVNLVLRDSPVFLPIVSKAIPSIFDNPQTIFLTAKVKDILFDGVELNCSGK
EFGTTAVCSQMKSQIPGLKFKKDNENIFLFSLLGSRNGTLTRRLKVHRGITHAKDLGRLVE
LDGKKEINIWREAECNRFHGTDGWIFPALTTPEEGLQSFSTDLCRSITLQYINDTVLKKVPV
RVYETDLGDQMTDENEKCYCRSANSCLKKGVFDLTKCMGVPIYATLPHFLRTDPSYINLV
DGLAPSELLHAIRVYFEPMTGTPLFAAKRMQFNLDLQPTNKIPLFSHLPIALFPMFWLEESV
DLDGYLLKKVQ
>DvalSNMP1a
MPKLKTLSFVFFPKLHLLALFTKFAYRCGKKQSPEKNMNFPMRLAIGSACSVVFIILVGFV
GFPKMIKGKVKDMVNLKPGMEIREMFLKVPFPLSFNVYIFSVLNPAEVQGGAKPHLKEM
GPFCYNEWKTKINVADNEGDDTISYEPVDTFKNAKRPKCLSVDTQVTIPHPMILGMVNTIL
RQKPGALTLANKAIKSIWSNPSSLFITVKAQDLLFNGVVIHCGVSDFAGKAICTNLKAEPSL
THLGGDDLGFSLMGPKNGTAGKRIKAFRGTQDFHKVGRIIEFEGKPKLDVWNNSKCDAIV
GTDGTIFPPMLKKEEGLASFAPDLCRSLIAQFEKHDKYDGIPVSSYFATLGDQSRNPTEKCF
CTTPETCLKKGLMDLYKCAKIPLYVSLPHFYDSHESYLKGVKGLKPDVEKHGIRIMFESLT
GSPVSARKRLQFNMPLEPNPKVELFHNFTPTVLPIFWVEEGVDLNNTFTKPLKTLFLTKKL
VNVVKYLVLMMSIGGFCAAGYLYFKSDNSMNVTSVQKVQPDQNGHRNIISTVFNGNHTA
GQDNEAYEDKH-
>DvalSNMP2
MKVPFPFQFKVYLFIVENAEEIQAGVKPLVKEQGPYVYQLTRWKDQVAWNHSTDEISYHE
YEAYKFDAESSGGLSEQDLVTVLNPAFLSFLYTAEMDPATRDFLPLIDESLDAIFGSYNSPFF
TNITVREFLFDGLRICKNGCNDDGFVAKMACNKIKERMAVTKQMRADGTDILYASFHYRN
NSHQGYITVAAGQKNETTIGEITQLNHQSTLNVWTKDQFECNRVSGLTTVFPTNVGVDTT
FQSFSEDICRTVSFEYSRREMVGLIKGNRYEALKSTFNTSKNACFCTDRTRNFDGNVGCLY
NGVLDLTTCQGAPVLVSFPHLLYADSRYLDKVEGLNPDPSKHAMFVTLEPTSGTPLKVAK
RVQFNLLLRAVRNITSLETVGNSIVPMFWIEESTALPEKYQDFIKNKIYRTLLIMDIVRYTVL
ALALAVIVCCIVLFIYAT
>DvalSNMP3
LLLHAVDIMQYLMIIIGCGCVTIAMYFRLKNRKSVTITPATGTRKSAPPKPNEKMDVSNLSI

AGILGDRAEKKP

>DponSNMP2
MFRNCCSPRLVFLYNLLAVLLLIASLVLAFWGLPQIISKQIHKQTELTENTDQWDRFKELPF
PMEFNIRFFLVTNPADVLNGSMPILKESEPYKYKSTIKRTDIRFDDIEEDSVTYRRSFSFEFD
GSGTTREDDSITVINPLLMASFQLTNDIQRLAMAGCRKYILEPAGLDQVFLTTTVRKLLFD
GIYFGFQNATGKGVACEMVRKELGKIVANVRVVEHLNDTDCYRLAIFNYKTDNFLKNSPD
GIYTINRGRNNATALGSIMRWNGATTSTTYGTSTSINNLTCHSIKGTDSTIYSPELKAGENL
MIFNTDLCRTIQLVQVSSNEVFNGINAFRYSTGYTLFRPETILKENDCYCSHGTKGADGKP
SCFLDGLLDFRPCLGAPVLISQPHFLHADVKYIRAVSGLSPDEDKHDIYLLLEPNTGTPLEG
RKRVQMNSVLRRQPLLSMITPPNMYEAVVPLLWLDEGFTLPQKYLDDLNAKYFKTVRIAT
GFKFGFIAVALALLVGCLFVACRKMYFRNAK
>DponSNMP1a
MNFPMRLAIGSACSLLFIILVGFVGFPKMIKGKVKDMVNLKPGMEIREMFVKVPFPLSFNV
YIFSVLNPAEVQGGAKPHLKEMGPFCYNEWKTKINVEDNEGDDTISYDPVDTFENAKRPK
CLSVDTLVTIPHPMILGMVNTILRQKPGALTLANKAIKSIWSNPSSLFITVKAQDLLFDGVV
IHCGVSDFAGKAICTNLKAEPSLTHLGEDDLGFSLMGPKNGTAGKRIKAFRGTQDFHKVG
RIIEFDGKSKLDVWNNSKCDTIVGTDGTIFPPMLKKEEGLASFAPDLCRSLIAQFDKHDKY
DGIPVSSFFASLGDQSKNPAEKCFCTTPETCLKRGLMDLYRCAKIPLYVSLPHFYDSHESYL
KGVKGLKPDVEKHGIRIMFELLTGSPLSARKRLQFNMPLEPNPKVELFHNFTPTVLPIFWV
EEAVDLNSTFTKPLKTLFLTKKLVNIVKYLVLLMSIGGFCAAVYLYFKSDDSMNVTSVQKV
QPDQNGHRNIISTVFNGNHTAGQDNEAYEDKY
>ItypSNMP1Fix
MPHPKNIAWAGGALAFGGVLFKVWLFDVLVRFGVKDQTALRYRNEVRGIYLKIPFPLNFK
IYFFNVTNPEEIQNGAKPVLNEVGPYWYDEYKERVDVIDNDTEDSLTYTPYDLFKFNPNM
STPLSDNDYVTIIHPVIVGMVNLLLRDSPMLLKVVSKAIPFIFNDPKTIFLTGRVKDILFDGV
VLNCTSKEFASTAVCGQMKGQVPGLKPTPGQPNLLLFSLLGPRNATRTGSLKVLRGIKHFQ
DLGRLLEVNGRKSIGIWAGDQCNRYDGTDSWIFPPLIQPESGLKSFSTDLCRNIKMKLVNE
TVVKKIPVGVFEPTWGVKVVTRRKSATVPTLPVXXXXXVFDLTKCMGVPLYATLPHFLDT
DPNYLKLVDGLKPDHEKHRIVVFFETMTGTPLKAAKRMQFNLELQQTNKLELFSKLPAAL
FPIFWLEEGMELEGYFLKKIQTVFMLLLFADVTIYVTIATGLSVCGAGFYQYWKNTKSLSI
TPLTKNNNGLSEPKLN
>ItypSNMP1aFix
DLFKCAGVPLYASAPHFYDCHVSYLKGVRGLHPDEQKHAIKILFESLTGSPVYAKKRLQFN
MPLEPNQKIDMFKNITPTVLPLFWIEEGVKLNNTYTKPLKSLFMMKKIVGVVKYLILLGSI
AGVTVGVYLYFKSGDTVNVQ
>ItypSNMP2Fix
MRFLQRVKFNLKTVFLCGISGVSLLVVALFLGFIIFPKVVNDQLLETKILREDTEQWAIFKKI
PFAFTFNVYLFTVENPEEILKGAKPVVKEKGPYVYKLYKWKEDIIWNYTTDEISYYEYEK
YVFDQEASGSLTEHDKVTLLNLPYLTFLYTAEANEATSGFLPLIDEALEFIFSGHNSPFLVNV
TVRDYLFEGVEICKNGCEDDGFVAKMACGKIKDNLKVAKQMRLHHKDILFATFHYRNNT
HQKYLTVNSGRQNHLEIGAITQLDNSSTMNVWNQFGCNQVSGLTGIFPINLGFKT
TFQSFSAEICRPVKLHFSTIKPFGSIKGYKYVALNTTFNTSMVENQCYCTGKIPNLDGNLGC
LYDGVLDLSTCLGAPIVVSFPHFLYADWRYVNNVKGLSPNETNHQIFVNLEPISGTPLEAA

TRIQFNLFLRPVRNITSLDSVADALVPLFWIEELTYLPQKYQDVITGKLYRSIFILNAIKYVLL
AIALVIITVCILIFLYTD

>DmelSNMP1
MQVPRVKLLMGSGAMFVFAIIYGWVIFPKILKFMISKQVTLKPGSDVRELWSNTPFPLHFY
IYVFNVTNPDEVSEGAKPRLQEVGPFVFDEWKDKYDLEDDVVEDTVSFTMRNTFIFNPKE
SLPLTGEEEIILPHPIMLPGGISVQREKAAMMELVSKGLSIVFPDAKAFLKAKFMDLFFRGI
NVDCSSEEFSAKALCTVFYTGEIKQAKQVNQTHFLFSFMGQANHSDSGRFTVCRGVKNN
KKLGKVVKFADEPEQDIWPDGECNTFVGTDSTVFAPGLKKEDGLWAFTPDLCRSLGAYY
QHKSSYHGMPSMRYTLDLGDIRADEKLHCFCEDPEDLDTCPPKGTMNLAACVGGPLMAS
MPHFYLGDPKLVADVDGLNPNEKDHAVYIDFELMSGTPFQAAKRLQFNLDMEPVEGIEP
MKNLPKLILPMFWVEEGVQLNKTYTNLVKYTLFLGLKINSVLRWSLITFSLVGLMFSAYLF
YHKSDSLDINSILKDNNKVDDVASTKEPLPSANPKQSSTVHPVQLPNTLIPGTNPATNPATH
HKMEHRERY
>DmelSNMP2
MIHWSLIVSALGVCVAVLGGYCGWILFPNMVHKKVEQSVVIQDGSEQFKRFVNLPQPLNF
KVYIFNVTNSDRIQQGAIPIVEEIGPYVYKQFRQKKVKHFSRDGSKISYVQNVHFDFDAVA
SAPYTQDDRIVALNMHMNAFLQVFEREITDIFQGFANRLNSRLNQTPGVRVLKRLMERIR
GKRKSVLQISENDPGLALLLVHLNANLKAVFNDPRSMFVSTSVREYLFDGVRFCINPQGIA
KAICNQIKESGSKTIREKSDGSLAFSFFGHKNGSGHEVYEVHTGKGDPMRVLEIQKLDDSH
NLQVWLNASSEGETSVCNQINGTDASAYPPFRQRGDSMYIFSADICRSVQLFYQTDIQYQ
GIPGYRYSIGENFINDIGPEHDNECFCVDKLANVIKRKNGCLYAGALDLTTCLDAPVILTLP
HMLGASNEYRKMIRGLKPDAKKHQTFVDVQSLTGTPLQGGKRVQFNMFLKSINRIGITEN
LPTVLMPAIWVEEGIQLNGEMVAFFKKKLINTLKTLNIVHWATLCGGIGVAVACLIYYIYQR
GRVVEPPVK

>TcasSNMP2
MGCSCCTIKVLLVCVVISVALLIVSLALAFKVFPDLLESEVNKAVRLEDGTKQYDRFVELP
FPVDFKVYLFNVSNPQQVLDGTEKPKLEEIGPFVYKQYRKKTILGKNEEEDTISYTQKETF
EFDAEASKPLTEESVVTVLNPALMSIYQLAEDLHLAGAADTCIKQTFENNQGKVFIEANVR
KLLFDGFSFCKNTSPGICGLVNDLICAIAATKRNSDLVLPDYSLIFSYLNYKRKPDDGKYTV
KRGLTNIEKLGHIVAWNDSLYTKFWGEGTTCSEVKGTDSTLYPPRVTTDSAFYIYSTDICRF
VKINYKGEESYKGIDGYLFETSEDTLRSSAPEEDCYCSKLSRDMEGKKSCFLDGVIDMQT
CFGVPVLFSFPHFLWADNKYLSAVEGLNPVEEKHKTYLVVEPNTGTPLKGMKRIQLNGVI
RPIVGIKSMLQTKRALLPLLWIEEGVSLPQKYVDELKSSYFDKVQIVDGVRYALIVISAILV
GAFGIIILRKRSHAKHHV
>TcasSNMP1a
MRLPVKIAIGCAIGLVVIIVFGFIAFPKMIKGKVKSMINLNKGSEIRQMFVKVPFALDFKIY
MFNVTNPMDVQKGALPVLKEVGPFCFEEWKEKVDLDDNDDEDVMFYNPKDTFYKANG
PGCLDGSQMITMAHPLILGMVNTVVRTKPGAISLISKAINSIYGNPDSIFMTASAMDILFDG
VVIKCGVKDFAGKAVCSQLKEAPDLRHVDENDLAFSFIGPKNATPGKRFKVLRGVKESHD
VGRILEYDNKKEMEVWPTKECNQYKGTDGTVFPPYLTKEEGLASYAPDLCRSLVAVYSG
DTKYDGIPVRIYTATLGDMSKNADEKCYCPTPDTCLKKGMMDLFKCAGVPVYVSLPHFY
ESDESYVKGVVGLNPNKKDHGIQILFESTTGGPVKAAKRLQFNMPLEPNPKLPIFANLPNT

VLPLFWVEEGVALNNTFTKPLKDLFKIMKIVKIAKWLIMLGCLGGLGAAGYLYFSKKGEA
NITPVHKVKPAENGVSTLGGEVNHAMSDNEIEKY
>TcasSNMP1b
MVKWQRQLKPGNEVRDFYIKLPIPLDFRVYFFNISNPEEVKQGEKPILKQIGPYCYDAYKE
KINVEDDKDNDTLTYNPYDTYFFNQMRTGDLSQDDYVTILHPLTVGIVNAVATQKPQYLS
AVNKALPVIFKENSSIYLTAKVREILFDGVLINCNVKDFSANAVCSQFKGQPAMVEVEKNI
YSFSLLGSRNGSIPTRITIHRGVKNAADIGRVVTIDNKTDLDVWPEPECNAFRGTDGWVFP
SFLEKEDGIWTVASDLCRSFKAQYVEDLKFHGVVVRKYFADLGDMSSNPAEKCFCPAPEK
CLPKGVMDLTKCMKVPLYCTLPHFLRADEKLLQQVEGLSPELERHIIKIYFEPLTGTPMLG
QRRIQFNLQLMPIPKVAMMKTVPEALHPILWIEEGVELEGFLLKKVTSVFTLLKLMTFVRY
IMLGLSIQGILYGGYKLYQESKSKKVSPVQNGTTESKNHNQGKTGGIELPSMNKRNKENT
KNA
>TcasSNMP1c
MSYKKITIISACCVVTIIGVAYIYAIRDISHRRNVRYKYIDRVNNVSNDVNGGVVSVGYCYD
YKRIDVDNADSTYTYDIYNRSGNSDDYVTIIHVVSVNYVSVKTHYNDAGKSITAKVRDID
GMINCTSRDTAMAVCTIRTKIGISKDYKYAGNGTTRITVRGIKSNGKVAVDNVTKSDWSNC
NYKGTDGWISGRKTIWMHATTCNIHADVGATSNGAVNKYYSDNICTNCSCGIDVTKCTAI
YISHRSDSIRGVKGNDTSHITRIGTSMAIRNVVKKITIMNVSVIHVWVMGVVNGWRMIKT
YTAVMKYISVASGTAYGGYHYKNKKYSKNIVSSK
>TcasSNMP1
MTSTARRRNIMKKVYKIMDRVYNITNSVNGVVKVGYCYDAKKIDVNGDSTYTYTYNDK
SGRTADDYVTVHIVGIVNTVSRDSIVDRAIKSIKDNIYITTKVRDDGMTINCKVDSATAVCT
KAIGIIKNVYKSIGRNGTNRYKVRGMKKWHGRVVNHKSTVWSTKKCNRRGTDGWIIDK
VGWTYSSDCRNMHVVTSHGVAKYYADGDMSSNDKCYCKTCKGMMDTRCMGVIYATHR
VDKVRRTVRGKITDHIVRVIIGTAKRMNIVKKISMKTAHIWIAIVGKMIKVVVAKVDVVK
YCAVCAVAGSYCYKRKKKAVTVSKTAKA
>AplaSNMP1
MYMKTPFALDFKVYMFNITNAEAVLNGESPVLDEIGPYCYDLWKEKVDPIDNEVNDTLT
YKGKMTWIFNKAKSAPLTGDEMVTIPHPLILGIAVAVARDKPAMLSLVSKALNSIFNNPPSP
FITATTNEILFEGLTVYCNVTDFAGKAACAQIKSEAKNVIYISDKIFKLSFFGDKNGTVDERP
FTVKRGLKNYKDIGRVVEFDNKPNMNVWPTKECNEYHGTDSTIFPPLLQKEEGIVAFSPDI
CRSLAAVFEKETFVKEVKVNKYTATLGDMSADDSLKCYCPEPNKCLKKGLMEITKCVGA
PLYASLPHFYASDESYVHGVRGLHPNEEEHGIYMYFEPMTGTPLGARKRLQFSMPLEPIPK
ISFMKNLPTTILPVFWVEEGADLGDEYVDQIKSAFKMIQIVFTAKWILFVVSAVTGAIAGL
MHFKNSKNILVTPVKNDESKGRSSVINTIDSLNTYVNGSNNKY
